# The Underlying Roles of Exosome-Associated PIGR in Fatty Acid Metabolism and Immune Signaling in Colorectal Cancer

**DOI:** 10.1155/2022/4675683

**Published:** 2022-09-15

**Authors:** Ying Liu, Yongbin Hu, Langmei Deng

**Affiliations:** ^1^Department of Pathology, Xiangya Changde Hospital, Changde, China; ^2^Department of Pathology, Basic Medical School, Central South University, Changsha, China; ^3^Department of Pathology, Xiangya Hospital, Central South University, Changsha, China; ^4^Department of Emergency, The Third Xiangya Hospital, Central South University, Changsha, China

## Abstract

The polymeric immunoglobulin receptor (PIGR), an exosome-associated glycoprotein, plays an important role in the occurrence and development of different tumors. This study aimed to investigate whether PIGR is essential for colorectal cancer (CRC). Comprehensive bioinformatics analysis and immunohistochemistry (IHC) revealed that expression of PIGR was significantly decreased in CRC patients. Upregulated PIGR displayed favorable prognostic values in CRC patients. Several algorithms, such as TISIDB and TIMER, were used to evaluate the roles of PIGR expression in the regulation of immune response in CRC. Moreover, GSEA enrichment analysis indicated the underlying role of PIGR in the regulation of fatty acid metabolism in CRC. Taken together, our findings might provide a new potential prognostic and immune-associated biomarker for CRC and supply a new destination for PIGR-related immunotherapy in clinical treatment.

## 1. Introduction

Colorectal cancer (CRC) is one of the most common cancers worldwide, with a high incidence and mortality rate [1, 2]. In recent years, various types of clinical treatments have been applied to CRC patients, including systemic chemotherapy and radiation. However, the average 5-year survival rate of CRC patients with positive regional lymph nodes is only 40%, while less than 5% of patients with distant metastases survive beyond 5 years [3, 4]. Therefore, it is significantly important to explore a novel biomarker to improve the overall survival rate of CRC patients.

The tumor immune microenvironment (TIME) has an important role in mediating cytotoxic drug response and tumor progression [5]. Exploring the underlying mechanisms of TIME displays an important role in the occurrence and development of CRC [6, 7]. Immune cells combined with signaling biomarkers could play a crucial role in the prognostic prediction of CRC patients [8, 9]. Therefore, it is very important to further study the tumor microenvironment to improve the patients' overall survival. Exosomes, the nano-sized vesicles, have the inherent potential to shuttle diverse biomolecules like proteins, lipids, and nucleic acids to the recipient cells [10, 11]. Employing exosomes as vehicles for the delivery of products to initiate antitumor immune responses shows striking therapeutic effects [12, 13]. Thus, exosomes could be considered as potential therapeutic targets and valuable biomarkers for the treatment of malignancies.

The polymeric immunoglobulin receptor (PIGR), an exosome-associated glycoprotein, picks up its cargo on the basolateral surface and carries it by the process of transcytosis to the apical face. The function and regulation of PIGR may be closely related to the immune defense of organisms [14]. Numerous studies have recently demonstrated the important roles of aberrant PIGR in different tumors' tumorigenesis. Qi et al. considered that the PIGR may be a tumor suppressor in nasopharyngeal carcinoma [15]. Increased expression of PIGR was correlated with hepatic metastasis and poor prognosis in colon carcinoma patients [16]. Whereas, more studies are still required to investigate the relationship between PIGR expression and the prognosis of CRC patients.

In this article, we explored the underlying mechanism of PIGR in CRC. Based on bioinformatics analysis and immunohistochemical technology, it was found that the exosome-related gene PIGR was significantly downregulated in CRC tissues. Survival analysis showed that high expression of PIGR was associated with a good prognosis in CRC patients. Furthermore, we analyzed the relationship between PIGR and tumor-infiltrating immune cells (TIICs) in CRC. These findings indicate that PIGR could be a novel prognostic and immune-related biomarker in CRC patients.

## 2. Materials and Methods

### 2.1. Data Acquisition

Three CRC datasets, GSE20842 [17], GSE23878 [18] and GSE25070 [19], were downloaded from gene expression omnibus (GEO) database [20] (Table 1). Then, we explored the codifferently expressed genes (co-DEGs) between CRC tissues and normal colorectal tissues. The screening criteria was shown as follows: |log FC| ≥1.5 and *p* value <0.05. Next, we used Venn plots to explore the overlapping molecules between the exosome-associated dataset and three GEO datasets. Moreover, we employed the Cancer Genome Atlas (TCGA) database [21] to evaluate the effects of co-DEGs on the clinical characteristics of CRC patients.

### 2.2. Bioinformatics Platforms

The profiles of co-DEGs were analyzed by comprehensive bioinformatic technologies (Table 2). The Kaplan–Meier plotter [22] was used to evaluate the prognostic values of the overlapping molecules, including overall survival (OS) and recurrence-free survival (RFS). In addition, several databases, such as TNMplot [23], GEPIA2.0 24 and TCGA-CRC, were used to confirm the downregulated expression level of PIGR. Subsequently, we used the Linked-Omics platform [25] to evaluate the interaction between PIGR and its coexpressed genes. Meanwhile, the Linked-Omics platform was used to analyze the gene enrichment. We employed the TISIDB [26], TIMER [27] and single-sample GSEA (ssGSEA) to evaluate the roles of PIGR expression in the regulation of immune response in CRC. We also evaluated the probable relationships between PIGR expression and several immune checkpoints, such as indoleamine 2,3-dioxygenase 1 (IDO1), CD274, programmed cell death 1 (PDCD1), cytotoxic T-lymphocyte associated protein 4 (CTLA4), and lymphocyte activating 3 (LAG3).

### 2.3. Immunohistochemistry (IHC)

Tissue sections were deparaffinized in xylene and rehydrated with ethanol, and then preincubated with 10% normal goat serum in pharmaceutical benefits scheme (PBS) (pH 7.5). Then, the tissue slides were incubated with the primary antibody overnight at 4°C and then stained with a biotinylated secondary antibody (SAB4600042, Sigma-Aldrich) for 1 h at room temperature. The peroxidase reaction was visualized with a 3, 3-diaminobenzidine chromogenic kit (ZLI-9019, ORIGENE). After that, the tissues were photographed under a conventional microscope (DMI3000 B, Leica). The formalin-fixed, paraffin-embedded specimens of CRC and adjacent tissues were obtained from the Department of Pathology, Xiangya Hospital, Central South University. The ethics for this study (202205114) was approved by the Ethical Committee of Xiangya Hospital, Central South University.

### 2.4. Statistical Analysis

In this report, the statistical difference was investigated by *t*-test assay. And the data were mainly depicted as the mean ± standard deviation (SD). *P* values <0.05 was considered to demonstrate statistically significant differences.

## 3. Results

### 3.1. Identification of the Co-DEGs in Colorectal Cancer

**Figure 1:**
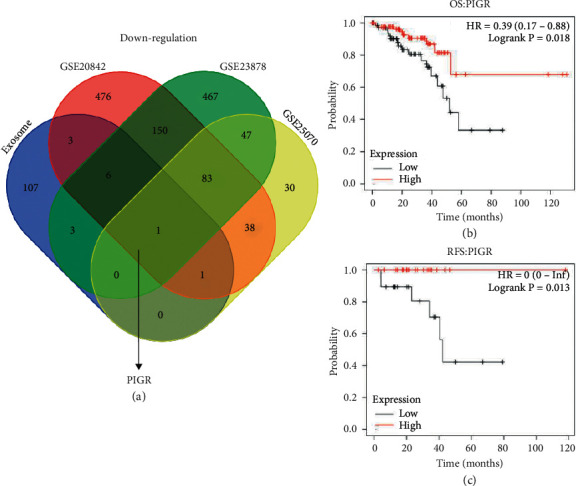
Identification of downregulated exosome-related PIGR in CRC. (a) The Venn plot showed one downregulated exosome-correlated gene (PIGR) in CRC progression. (b-c) The prognostic values of PIGR in CRC patients. Abbreviations: OS, overall survival; RFS, recurrence-free survival.

We explored the co-DEGs between CRC tissues and normal colorectal tissues from three GEO-CRC datasets. And we found 1262 upregulated genes and 883 downregulated genes in GSE20842, 258 upregulated genes and 1011 downregulated genes in GSE23878, and 86 upregulated genes and 222 downregulated genes in GSE25070 (Supplementary Table S1). A Venn analysis (http://bioinformatics.psb.ugent.be/webtools/Venn/) was used to explore the potential differently-expressed exosome-related genes in CRC. Accordingly, one downregulated exosome-related gene, PIGR, was identified in CRC tissues (Figure 1(a)). Then, the Kaplan–Meier plotter database was used to analyze the effects of PIGR expression on the prognosis in CRC patients. As shown in Figures 1(b) and 1(c), high expression level of PIGR was related with good OS (HR = 0.39, 95% CI = 0.17–0.88, *p*=0.018) and RFS (HR = 0, 95% CI = 0-Inf, *p*=0.013) in CRC patients. These results collectively suggest that PIGR overexpression could be significantly associated with a favorable prognosis in CRC patients.

### 3.2. Downregulated Expression of PIGR in Colorectal Cancer

**Figure 2:**
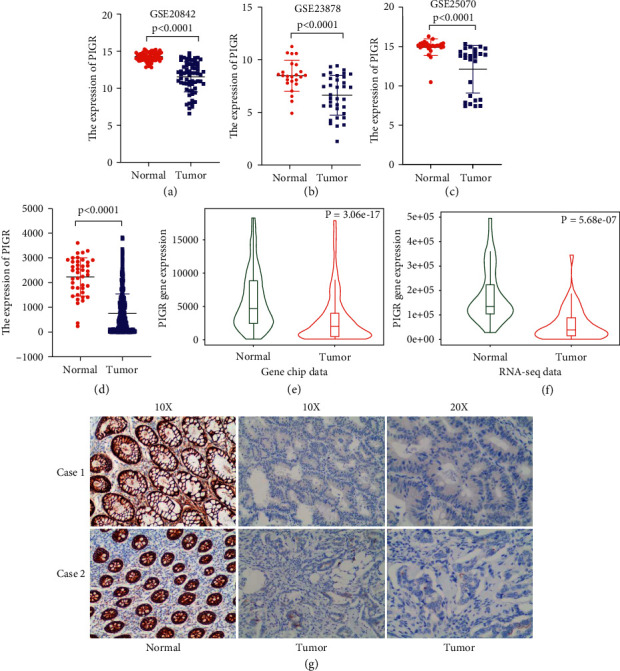
PIGR was downregulated in CRC patients. (a-c) The expression level of PIGR was lower in the three GEO-CRC datasets. (d) The expression level of PIGR was lower in the TCGA-CRC. (e-f) TNMplot database depicting the downregulated PIGR expression in CRC tissues from gene chip data and RNA-seq data. (g) Representative IHC results showing the downregulated PIGR expression in CRC tissues.

By comprehensively analyzing the expression levels of PIGR in the three GEO-CRC datasets, we found that PIGR was lowly expressed in CRC tissues (*p* < 0.0001) (Figures 2(a)–2(c)). Besides, the data results from TCGA-CRC further verified that the expression level of PIGR was significantly different between the normal group and the CRC group (*p* < 0.0001) (Figure 2(d)). What's more, the TNMplot database indicated that PIGR mRNA expression was both lower in CRC tissues from gene chip data (*p*=3.06*e* − 17) and RNA-seq data (*p*=5.68*e* − 07) (Figures 2(e) and 2(f)). In addition, the IHC results confirmed that PIGR was downregulated in CRC tissues. Together, these results proved the lower expression of PIGR at mRNA and protein levels in CRC.

### 3.3. The Coexpression Network of PIGR in Colorectal Cancer

**Figure 3:**
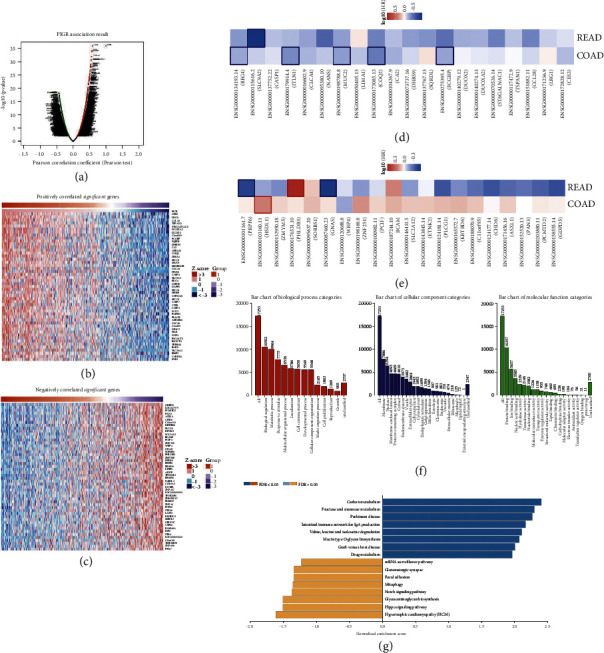
The coexpression network of PIGR in CRC. (a) The Linked-Omics platform portraying the crucially associated genes with PIGR in CRC patients. (b-c) Heatmaps showing the top genes that were positively and negatively correlated with PIGR in CRC. (d-e) Survival heatmaps downloaded from the GEPIA2.0 database displayed that the top genes that were positively and negatively associated with PIGR in CRC. (f-g) GO and KEGG enrichment of PIGR-coexpressed genes in CRC patients.

We explored the coexpression network and biological functions of PIGR in the TCGA-Colorectal adenocarcinoma (COADREAD) cohort. In Figure 3(a) and Supplementary Table S2, we presented the PIGR coexpressed genes. Meanwhile, Figures 3(b) and 3(c) and Supplementary Tables S3 and S4 have demonstrated these candidate genes that are positively and negatively correlated with PIGR in CRC patients. Notably, the top 20 positively-related genes were highly likely to be low-risk factors for CRC patients (Figure 3(d)). Furthermore, 1 of top 20 negatively-related genes might be the high-risk factor in CRC (Figure 3(e)). In addition, the Gene Ontology showed that the genes coexpressed with PIGR were mainly involved in multiple biological process categories, such as biological regulation, metabolic processes, and response to stimulus. In the category of cellular component, these genes mainly took part in the membrane, nucleus and membrane-enclosed lumen. Then, in the molecular function categories, these coexpressed genes are involved in the protein binding, ion binding, and nucleic acid binding (Figure 3(f)). Moreover, the KEGG analysis indicated that the most likely enriched pathways were carbon metabolism, fructose and mannose metabolism, hippo signaling pathway, and Notch signaling pathway (Figure 3(g)).

**Figure 4:**
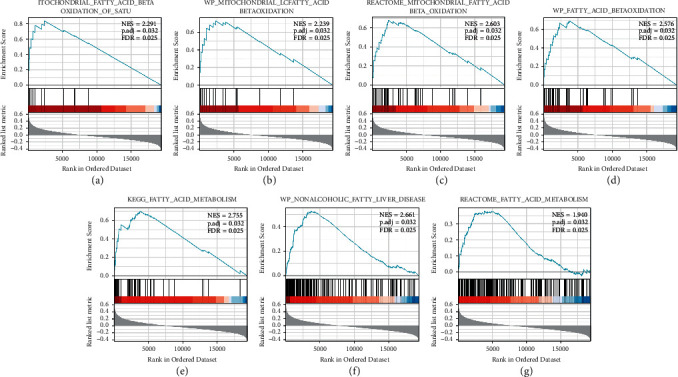
The GSEA enrichment analysis of PIGR-related genes in CRC. (a-g) PIGR-related genes were involved in several fatty acid metabolism pathways in CRC, such as mitochondrial fatty acid beta oxidation.

Next, we performed GSEA enrichment analysis of PIGR-related genes in CRC. As shown in Figures 4(a)–4(g), aberrantly expressed PIGR might involve in the regulation of fatty acid metabolism-related signaling pathways, such as mitochondrial fatty acid beta oxidation, mitochondrial fatty acid beta oxidation, fatty acid metabolism, nonalcoholic fatty acids. All the statistical significance was*p* < 0.05. Overall, our results suggest that PIGR may be involved in the cellular metabolism in CRC.

### 3.4. The Link between PIGR with Immune Regulation

**Figure 5:**
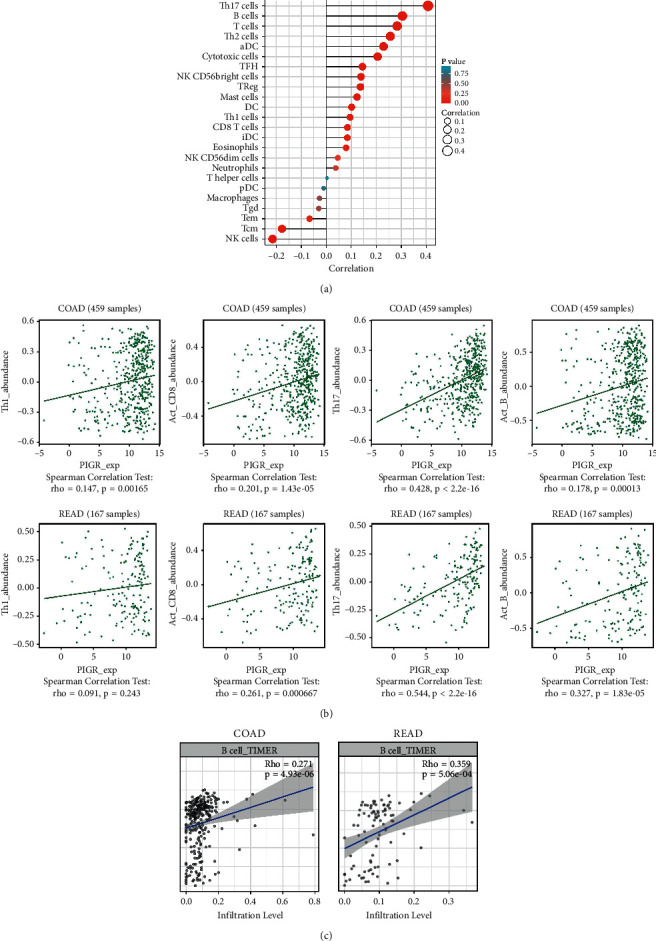
The relationship between the expression level of PIGR and immune cells in CRC patients. (a) The diagraph showing the relation between PIGR expression and 24 types of immune cells. (b) TISIDB database showed the relationship between PIGR and immune infiltration cells, such as T helper type 17 (Th17) cells, B cells, and so on. (c) TIMER database showed the relationship between the expression level of PIGR and immune infiltration cells.

We used ssGSEA to analyze the effects of PIGR on immune regulation in the TCGA-COADREAD cohort. The expression of PIGR was significantly positively correlated with T helper type 17 (Th17) cells, B cells, and so on (Figure 5(a)). The results obtained from TISIDB database also confirmed the similar findings (Figure 5(b)). The scatter plot from TIMER database further demonstrated that the expression level of PIGR was strongly positively correlated with B cell in colon adenocarcinoma (COAD) and rectum adenocarcinoma (READ) patients (Figure 5(c)).

**Figure 6:**
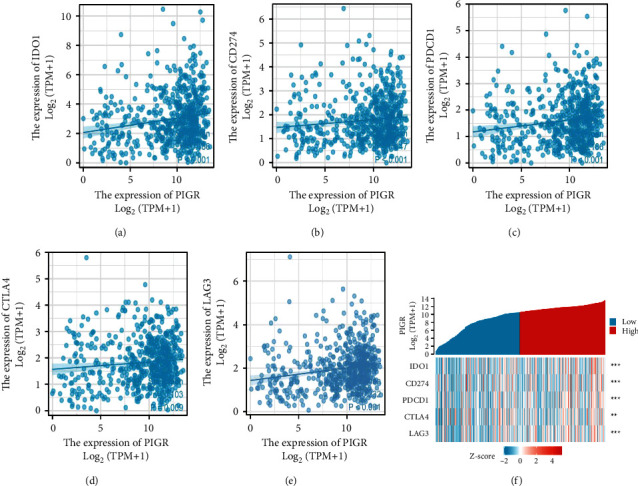
The relationship between PIGR expression and several immune checkpoints. (a-f) The scatterplot and heatmap depicted that PIGR expression was positively correlated to IDO1, CD274, PDCD1, CTLA4, and LAG3.

**Figure 7:**
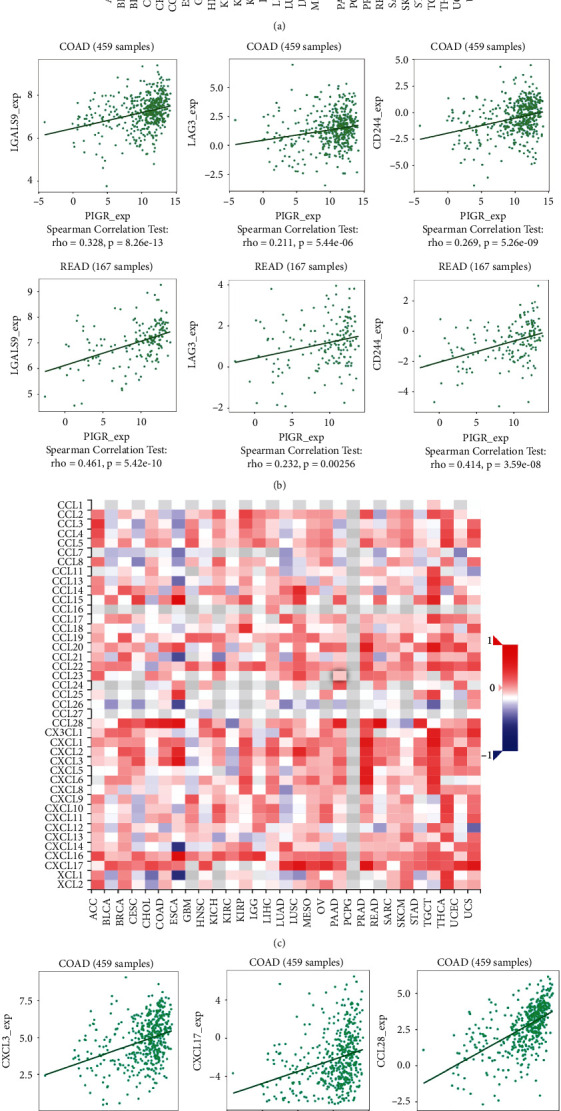
The roles of PIGR in the regulation of immunoinhibitors and cytokine receptors in CRC patients. (a) The diagraph showing the correlation between PIGR expression and immunoinhibitors. (b) The scatter plots depicting the top three immunoinhibitors positively-related with PIGR expression. (c) The picture showing the connection between PIGR expression and cytokine receptors. (d) The scatter plots portraying the top three cytokine receptors positively-related with PIGR expression.

Next, we explored the relationship between PIGR expression and several immune checkpoints and found that the expression level of PIGR was positively correlated with IDO1 (Spearman *r* = 0.186, *p* < 0.001), CD274 (Spearman *r* = 0.147, *p* < 0.001), PDCD1 (Spearman *r* = 0.186, *p* < 0.001), CTLA4 (Spearman *r* = 0.103, *p*=0.009), and LAG3 (Spearman *r* = 0.232, *p* < 0.001) (Figures 6(a)–6(c)). The patients with high level of PIGR displayed overexpressed IDO1, CD274, PDCD1, CTLA4, and LAG3 (Figure 6(f)). Additionally, the results from the TISIDB platform showed the relationship between PIGR levels and other immune-associated signatures, including immuno inhibitors and cytokine receptors.  Figure 7(a) conveyed the correlation between the immuno inhibitors and the expression of PIGR in CRC patients. The top three immuno inhibitors closely related to PIGR were galectin 9 (LGALS9), LAG3, and CD244 (Figure 7(b)). In addition, the correlation between PIGR and cytokine receptors has been displayed in Figure 7(c). And the top three receptors positively associated with PIGR expression were C-X-C motif chemokine ligand 3 (CXCL3), C-X-C motif chemokine ligand 17 (CXCL17), and C–C motif chemokine ligand 28 (CCL28) (Figure 7(d)). Taken together, these findings suggest that aberrantly expressed PIGR is involved in the immune regulation of CRC patients.

## 4. Discussion

Numerous studies have indicated the important role of exosomes in the occurrence and development of human cancers [28]. In this article, we elucidated the downregulated exosome-associated gene, PIGR, in the prognosis and immune regulation of CRC patients. Using the Kaplan–Meier plotter database, we found that high expression of PIGR was associated with a better prognosis in CRC patients. Gene enrichment analysis indicated that the coexpressed genes of PIGR were involved in the regulation of the immune microenvironment and fatty acid metabolism in CRC.

Exosomes are small extracellular vesicles secreted by almost all types of cells, including tumor cells. As important mediators of intercellular communication, exosomes provide an alternative cargo-handling mechanism to maintain homeostasis and cell survival [29]. Exosomes enhanced or inhibited certain important mediators and changed the tumor microenvironment, thereby altering the occurrence and development of different types of tumors [30–32]. Some differentially expressed RNAs and proteins in exosomes have been identified as potential biomarkers linked to CRC initiation and progression. Wang et al. considered that CRC cell-derived exosomal miR-146a-5p and miR-155-5p could activate the JAK2-STAT3/NF-*κ*B signaling pathways, thereby enhancing the invasive ability of CRC cells [33]. Studies have found that in human CRC cells, exosomal Nrf2 plays a pivotal role in oxaliplatin resistance [34]. Thus, further studies into the underlying mechanisms of exosomes might be beneficial for the treatment management of CRC patients. Accordingly, our study aimed to explore the prognostic values of PIGR in CRC patients, and we concluded that a high expression level of PIGR was associated with a good prognosis.

Nowadays, the roles of aberrant PIGR in tumors remain controversial, which might be due to the tumor heterogeneity or the different underlying mechanisms. In hepatocellular carcinoma, PIGR-loaded extracellular vesicles could activate Akt/GSK3*β*/*β*-catenin signaling cascades, driving cancer stemness, tumorigenesis, and metastasis [35]. Ohkuma et al. have assessed the prognostic value of PIGR in pancreatic cancer patients after surgical resection and determined that the overexpression of PIGR was correlated with poor prognosis in pancreatic cancer [36]. Through transcriptomic sequencing analysis, Bao et al. demonstrated that PIGR was downregulated in breast cancer. PIGR overexpression could suppress cell proliferation and adhesion in breast cancer cells [37]. In this paper, the bioinformatics and IHC results revealed the downregulated exosome-related PIGR in CRC tissues.

Immunotherapy is a novel anticancer method in the clinic. The combination of traditional treatment methods with immune checkpoint inhibitors could provide promising treatment strategies for cancer patients, including CRC [38]. Numerous studies have shown that immunotherapy can inhibit the growth of colorectal cancer cells and prolong the survival period of patients [39–41]. In this paper, the correlation between PIGR and immune-associated signatures was explored by comprehensive bioinformatic technologies. PIGR was positive with tumor-infiltrating of B cells, Th17 cells, T cells, and Th2 cells. Meanwhile, PIGR had a negative correlation with tumor-infiltrating NK cells and Tcm cells. Studies have demonstrated that immune checkpoint inhibitors have been regarded as potential strategies for enhancing immune responses in patients with CRC [42]. We found that the expression of PIGR had a positive relationship with several immune checkpoints, including IDO1, CD274, PDCD1, CTLA4, and LAG3. These above results implied that PIGR was strongly associated with immune responses and immune regulation, implying that PIGR could be a novel prognostic and immune-related biomarker of CRC patients.

## 5. Conclusion

Overall, this paper reported that the downregulated exosome-associated gene PIGR was significantly associated with a good prognosis in CRC patients. Aberrant PIGR expression might participate in the regulation of immune response and fatty acid metabolism. Therefore, we identified PIGR as a novel, valuable prognostic and immune-related biomarker for patients with CRC.

## Figures and Tables

**Table 1:** The upregulated genes and downregulated genes in the three GEO datasets.

GEO datasets	Platform	Sample size		DEGs	References
		Cancer	Normal		
GSE20842	GPL4133	65	65	1263 upregulated genes and 884 downregulated genes	[17]
GSE23878	GPL570	35	24	258 upregulated genes and1012 downregulated genes	[18]
GSE25070	GPL6883	26	26	87 upregulated genes and 223 downregulated genes	[19]

**Table 2:** Bioinformatics platforms that are employed to analyze the role of PIGR in colorectal cancer.

Database	URL	References
GEO	https://www.ncbi.nlm.nih.gov/gds/?term=	[20]
TCGA	https://portal.gdc.cancer.gov/	[21]
Kaplan–Meier plotter	https://kmplot.com/analysis/	[22]
TNMplot	https://www.tnmplot.com	[23]
GEPIA2.0	https://gepia.cancer-pku.cn/	[24]
Linked-Omics	https://www.linkedomics.org/admin.php	[25]
TISIDB	https://cis.hku.hk/TISIDB/	[26]
TIMER	https://cistrome.shinyapps.io/timer/	[27]

## Data Availability

The original contributions presented in the study are included in the article/Supplementary Material and are available upon request to the corresponding authors.

## Supplementary Materials

Supplementary Materials Supplementary Table 1. The upregulated genes and downregulated genes in the three GEO datasets. Supplementary Table 2. The coexpressed genes possess a positive and negative relationships with PIGR. Supplementary Table 3. The top 20 genes positively correlated with PIGR in colorectal cancer. Supplementary Table 4. The top 20 genes negatively correlated with PIGR in colorectal cancer.
