# Supplementary material for: The Underlying Roles of Exosome-Associated PIGR in Fatty Acid Metabolism and Immune Signaling in Colorectal Cancer
Source: J Oncol. 2022 Sep 15;2022:4675683. doi: 10.1155/2022/4675683 (PMC9499750; doi:10.1155/2022/4675683)
Supplement: Supplementary Materials — Supplementary Table 1. The upregulated genes and downregulated genes in the three GEO datasets. Supplementary Table 2. The coexpressed genes possess a positive and negative relationships with PIGR. Supplementary Table 3. The top 20 genes positively correlated with PIGR in colorectal cancer. Supplementary Table 4. The top 20 genes negatively correlated with PIGR in colorectal cancer. [file 4675683.f1.zip › 4675683.f1/Supplementary Table 1.pdf]

Supplementary Table 1. The upregulated genes and downregulated genes in the three GEO

GSE20842	GSE20842	GSE23878	GSE23878	GSD25070	GSD25070+DOWN
FOXQ1	MYOT	CDH3	MYOT	CEMIP	SCARA5
CLDN1	SEMA3D	ETV4	MAMDC2	CLDN1	ADH1B
CDH3	ROR1	FOXQ1	SCN7A	ESM1	GUCA2B
FABP6	NAP1L2	ESM1	NRXN1	FOXQ1	SCGN
KRT80	SCN9A	CEMIP	TMEM100	CDH3	GREM2
ETV4	ANGPTL1	HILPDA	LYVE1	SALL4	CLEC3B
AJUBA	DSCAML1	EPHX4	LYVE1	MMP7	TMIGD1
IFITM1	CPM	GTF2IRD1	ABI3BP	LRP8	TSPAN7
CEMIP	TPH1	CLDN1	SCGN	COL11A1	CA7
TRIB3	CPM	TRIB3	BCHE	EVA1A	PI16
CEMIP	ROR1	AJUBA	CA7	TGFBI	PCOLCE2
INHBA	NEDD4L	AJUBA	GREM2	SLC7A5	BEST4
ETV4	ABCA8	LOC10192	CXCL12	KRT80	CFD
IFITM3	SLC4A4	SOX4	PRIMA1	NFE2L3	HPGD
IFITM4P	ASPA	INHBA	DPT	ETV4	PLAC9
SLC6A6	OR51E2	UBE2C	ABCA6	ETV4	NR3C2
ASCL2	XKR4	SOX4	NAP1L2	TRIB3	PKIB
EPHX4	SCGN	SLCO4A1	LIFR	TMPRSS3	AQP8
KRT23	SCG2	MIR7112//	C7	CBX2	SFRP1
SLC7A5	ENDOD1	TEAD4	TSPAN7	PDPN	CXCL12
TGFBI	CA12	WNT2	VSTM2A	CXCL8	GUCA2A
KIAA1549	RNF112	ZAK	ANGPTL1	CTHRC1	XKR4
CEP72	PLP1	NFE2L3	ATP1A2	TIMP1	OTOP2
S100A2	RNF152	EGFL6	SSBP2	CDC25B	MYOT
PITX2	GLP2R	SAPCD2	FHL1	PHLDA1	KRT24
NKD2	UGP2	C2CD4A	ADH1B	BGN	ABI3BP
FUT1	CNTN3	TEAD4	SFRP1	COL1A2	C2orf88
PRR7	ZZEF1	C6orf223	DPT	MMP11	FAM107A
ACAN	ETFDH	PPM1H	RCAN2	OLR1	MAMDC2
NUDT1	PKIB	SCGB1D2	ADH1B	FAP	CA1
NFE2L3	SMPDL3A	NFE2L3	ANK2	PLAU	CWH43
CLDN1	PADI2	TGFBI	F13A1	MSX1	CHP2
TESC	PADI2	KIF18B	FOLR2	GZMB	PRPH
TGIF2	AHCYL2	STC2	SORCS1	CST1	ADH1A
MDFI	NEDD4L	RNF43	CFD	STC2	HSD17B2
TPX2	IL1R2	SLC5A6	NEGR1	UBD	SLC51B
SLC29A1	RFX6	INHBA	SCARA5	SULF1	KLF4
MRGBP	CASD1	HILPDA	SCN9A	SLCO4A1	ACADS
HILPDA	ANO5	LRRC8E	HPGDS	MMP3	MATN2
PDPN	MPC1	CRNDE	SPIB	TESC	CLCA4
MTHFD1L	GCG	MMP7	MMRN1		SLC25A34
C2	CA12		CD36	COL1A1	PYY
SLC22A3	EDIL3	MMP11	AFF3	EPSTI1	VIP
CSE1L	VSTM2A	AXIN2	SORBS2	COL10A1	CNTFR
SLCO4A1	IL1R2	CSE1L	JAM2	PSAT1	UGT1A10
IMPDH1	SMIM14	CBX2	SDPR	NKD2	UGDH
IFITM2	CDH19	IMPDH1	NRXN1	CXCL10	GPAT3
RIPK2	HIGD1A	CLDN1	KIAA2022	STC1	OGN
DPEP1	SCG3	TESC	SGK1	COL8A1	CA4
MTHFD1L	SPIB	TRIP13	NR3C2	MMP1	BEST2
SALL4	PYY	FOXM1	SCARA5	KRT17	KCNIP4
PDPN	MAMDC2	E2F1	FXYD1	TNFRSF6B	MAL
GTF2IRD1	LOC28345	NEK2	RGS13	UBE2C	AKR1B15
INHBA	ADAMDEC	GRIN2D	GREM2	TACSTD2	DHRS11
RGS16	GLDN	C11orf84	CHRDL1	COL5A2	LDHD
RAE1	INSM1	STC2	PTN	COMP	OGN

APLN	ADH1A	CDCA5	P2RY12	CXCL1	SCNN1B
C2	SCN9A	CDC25B	SCN7A	OSM	MT1M
MIR4435-	CA12	TOP1MT	CHGB	IFITM1	PDK4
XPOT	HIGD1A	SNHG17	PCSK2	CXCL9	HSD11B2
MMP7	CA2	LMNB2	LIFR	TMEM158	MAOA
CENPP	ABHD3	ZAK	DCLK1	COL7A1	PDE9A
GPT2	ENTPD5	FOXP4-AS	CNR1	CXCL2	SST
TEAD4	CNTN3	KRT23	FHL1	KLK6	ADAMDEC1
TRIB3	ARHGAP2	CRNDE	MEF2C	SPP1	CXCL12
SLC6A6	CDKL1	TBC1D16	IL6R	KRT6B	MS4A12
RGS16	CRYBA2	IER5L	GUCA2B	MMP10	MMP28
MYL6B	STYK1	ANLN	SDPR	SLCO1B3	SCIN
SHMT2	TEX11	BIRC5	EDIL3	MMP12	EDN3
ANGPT2	IQGAP2	SNHG17	ARHGAP2	TCN1	GPM6B
C2	CDKL1	SNORD88	PGM5-AS	SPP1	SLC16A9
PAFAH1B	SLC4A4	SLC7A5	RBMS3	MMP9	GPT
C4orf48	GFRA2	ANXA9	OMD	GDF15	SELENBP1
MEX3A	ENTPD5	WDR4	SYNPO2	ASCL2	GPX3
DIAPH3	LINC0034	ATP11A	FHL1	CLDN2	CLDN23
VSNL1	CLDN23	GDF15	PKIB	IRX3	GCG
TGM2	SVOP	KIF20A	SPARCL1	THBS2	LYVE1
CHI3L1	FMN2	SOX4	GCNT2	CXCL5	SRPX
KIAA1549	ABCC8	TNS4	METTL7A	DPEP1	AHCYL2
AZGP1	SLC30A10	GINS1	PGM5	S100P	SLC17A4
HKDC1	C16orf89	AJUBA	LINC0114	LY6G6D	EDN3
DGAT2	UGT1A8	PSAT1	SRPX	CCL20	TMEM37
RNF43	ADGRL3	AURKA	C2orf88	MSLN	AKR1B10
DUSP14	SLC9A2	RAD54B	ASPA	CEL	CHGA
RAB36	RAB27A	CHI3L1	FGL2	LCN2	GPM6B
GNB1L	CHGB	CTHRC1	GNG2	REG1B	CA2
GPSM2	PTGDR	CHI3L1	FAM107A	REG1A	ITM2C
RNASEH2	CDKL2	LOC72968	RMDN2		C7
AZGP1	ENDOD1	AURKA	STMN2		SDCBP2
CPNE7	LRRC19	PRR7	TCF21		ADTRP
ARNTL2	NR3C2	MCM10	STMN2		ADH1C
THBS2	CAPN13	TPX2	SCG2		
STC2	C2orf88	IQGAP3	CFHR2		SDCBP2
WISP1	PLCL2	TRIM29	MEF2C		CIDEC
PRR7	SEMA6A	SMKR1	NLGN1		ITM2C
KLF7	CHGB	NKD2	UGT1A3		VIP
CBX8	CMAHP	SKA3	PIR-FIGF		CD36
CHCHD6	CDC42SE2	BLACAT1	SPINK2		C2orf40
NEBL	SLC22A23	SCD	NOVA1		ARHGAP44
PHF19	GHR	GPT2	KRT24		VSIG2
RECQL4	DUSP26	BIRC5	C16orf89		MALL
APLN	CLMN	ASCL2	PLPP3		FCGBP
TMEM132	GNG7	UBE2S	LRMP		SLC26A3
ATP11A	DMXL1	S100A2	LRMP		FABP2
AUNIP	PDE4D	COL11A1	SSBP2		ARL14
TUBB3	SLC4A4	DSCC1	GPM6B		VIPR1
ANXA9	TTR	OTUB2	UGT1A3		DPT
MMP11	HAPLN1	ADAM12	ETFDH		PAPSS2
FBXO41	MMP28	TCN1	GPM6B		BCAS1
TMEM206	PDCD4	SRPX2	TPH1		SDCBP2
MMP3	CAPN13	MCM10	PLPP1		HMGCS2
MIR4435-	VPS4B	SNTB1	KCNAB1		CEACAM7
CDCA5	RASSF6	CDK1	HHIP		MFAP5
CBFB	PLCL2	CST1	P2RY14		PDZD3

C2	UGDH	ALDH4A1	GCG	TMEM171
AURKA	PHLPP2	RNFT2	PTN	NEU4
FJX1	SST	PTP4A3	MFAP4	CTSG
PALD1	BCAR3	ULBP2	NBEAL1	LRRN2
RFC3	FAM151A	SLC6A6	ADH1B	NXPE4
GNPDA1	CNNM4	MSX2	GLIPR2	UGT2B7
AHCY	SCN7A	GTF3A	FHL1	CES2
COL12A1	CCL23	CDC6	SYNE1	PCK1
CDC25B	CTNND2	ADAMTS1	BEST4	GBA3
COL8A1	SEMA6D	CST2	ABCA9	SLC22A18AS
ANLN	CAMK2D	ATP11A	PLN	NXPE4
TIMP1	CMAHP	WISP1	LPAR1	TRPM6
MYBL2	NEUROD1	AZGP1	ADTRP	FHL1
PTP4A3	CPB1	CELSR3	FAM107A	TPSG1
CDK5	FEV	EFNA3	ZEB2	UGT2B7
HKDC1	TMEM59	KRT80	HLF	REP15
FAP	FAM107A	BGN	MIR22///MIR22HG	NXPE4
KIF2C	LIFR	SLC7A11	MRC1	MT1H
SRPX2	TMEM9B	PITX1	COL14A1	MT1E
CARD14	ANKRD20/	CLDN2	CNNM2	MYO1A
OXTR	RMDN2	LRRC8E	PEG3	GSN
KIF3C	CLDN8	ATAD2	LY75-CD302///CD30	TUBAL3
CENPN	RBM24	DGAT2	ADAMTSL3	LAMA1
SAA1	SULT1A2	DIAPH3	TNXB///TNXA	CA12
MRGBP	CA7	MMP1	CCL15-CCL14///CCL	HEPACAM2
UBE2S	FAM107A	KLK10	PRKAR2B	LRRC19
PPM1H	TDP2	LRP8	PLAC9	SGK1
MEX3A	MMP28	PITX1	ITM2A	C15orf48
WDR66	SLC17A8	ONECUT2	EPB41L3	CLDN8
CDK4	ANKRD20/	WNT5A	VIP	CEACAM1
XPOT	CA12	G6PD	PDE4D	MT1G
WNT2	SCP2	BGN	DCLK1	CLCA1
PDRG1	GHR	DPEP1	TPH1	HEPACAM2
IER5L	B3GNT7	SP5	PRELP	CMBL
FAM89A	CHP1	COL1A2	RTN1	UGT2B17
CD3EAP	KRT20	ADAMTS2	FAM13C	GGT6
SULT2B1	CBLN2	SQLE	ITPR1	BCHE
GDF11	PLCE1	GAD1	CFHR1	PLAC8
JADE3	ATP5S	MSX1	SYNPO2	SLC44A4
TNS4	TTLL6	TDGF1	CD160	NXPE2
TMPRSS3	SFRP1	ALDH4A1	NAAA	IGFBP6
PLK1	C4orf19	NEK5	EPB41L3	DNASE1L3
CTHRC1	WSCD1	ASPHD1	LILRB5	TCEA3
SKA3	DCLK1	MMP3	PRELP	SLC44A4
PMEPA1	MOB3B	PMEPA1	CD36	MYH11
BCL2L12	ATP1A2	COL11A1	GPM6A	CA12
ZAK	DPF3	GALNT6	MBNL1-AS1	MEP1A
LARGE2	CCDC68	NEBL	ADAMDEC1	CAPN13
MFAP2	TRPM6	UCA1	NDUFC1	HSPB6
TOMM34	C2orf88	GRINA	PLCE1	GPA33
SLC19A1	MFSD4A	ASCL2	PKIB	C15orf48
RNF24	KCNMA1	GPR143	THRB	C10orf99
TKT	KLB	SH3TC2	ARHGAP15	SCGB2A1
UBE2S	SLC51B	DUXAP10	C2orf88	GCNT3
AXIN2	ZNF397	PLEKHS1	ARL14	ITLN1
NFE2L3	UGDH	PTP4A3	IGSF10	AKR1C2
TEX10	CASD1	CGREF1	NR3C1	MYOM1
TRAIP	LILRB5	SLC35D3	MYH11	PTGS1

SNTB1	HPGD	CDC25A	PDE3A	SI
MZT1	HRK	NKD1	LINC01082	UGT2B15
TDGF1	GUCA2B	MIR4435-	ITM2A	TSPAN1
PRELID3A	ADH1B	PAH	CDH6	ZBTB16
DDIAS	KIAA1468	CLEC5A	HAPLN1	CALB2
KLK10	SNX24	COL1A1	MFAP5	TMPRSS2
GPSM2	FNIP2	HKDC1	MYH11	FCGBP
COL12A1	HOXD1	LY6G6F	DHRS11	PLA2G10
TDGF1	RIOK3	TOP2A	MYH11	PGM5
DGAT2	KLK15	LRP8	KLRF1	PGM5
PPM1H	MFSD4A	SF3A2	CP	MS4A8
NEK2	MAOA	CXCL1	GATM	NPTX1
FOXM1	MOGAT2	ADAM12	CNTN4	KCNMA1
DIAPH3	HSD17B11	SLC6A20	PCOLCE2	LMO3
GDPD5	MBOAT1	TRIM29	C2orf40	CKB
ATP6V1F	CDKN2B	HOXB8	PDE3A	CEACAM1
GPR143	CAMK2D	COL11A1	PLCG2	MT1F
TRIP13	GNPTAB	SCD	FOXP2	NR1H4
PHLDA1	ADAMTSL	PMEPA1	LOC102724156	AOC1
ARID3A	DMD	SFTA2	SCN9A	CRYAB
ESPL1	TP53INP2	PLEKHS1	TMEM110-MUSTN1/	CAPN9
IMPDH1	FAM214A	SOX9	KIT	PGM5
IPO5	FRMD3	PABPC1L	GBA3	A1CF
BTBD16	HSD11B2	NEBL	GPM6A	MUC4
HMGB3	MYO1D	AZGP1P1/	MYH11	JCHAIN
EEPD1	SLC26A2	TACSTD2	KLF4	NNAT
MACC1	TRPM6	KRT6B	FGF7	ANPEP
TG	MATN2	VSNL1	ADH1C	SLC51A
NOX4	KIRREL3	COL10A1	C22orf42	MYH11
SOX4	ATP2B1	ONECUT2	AKR1B10	CEACAM1
CD3EAP	LRRC75A	CXCL5	EPB41L3	MGP
PLEKHS1	LNPK	SYT7	DPYD	TCEAL2
MMP10	MATN2	COL10A1	PER3	CASQ2
FANCB	VPS4B	SLC6A6	SLC4A4	LGALS4
C11orf84	PPM1L	KIF26B	ANKRD44	ST6GALNAC1
KIAA1549	KCTD9	IL24	PTGDR	MUC2
TDGF1P3	CHRNA3	BGN	NAAA	SYNM
UTP4	MARCKS	MMP11	FAXDC2	PIGR
LY6E	HSBP1L1	MMP10	NPY6R	LRRC26
SNAI1	SFRP1	KHK	SLC4A4	RBPMS2
ESM1	CASP7	CXCL3	PDZRN4	CNN1
C19orf48	GLTP	SCD	FOXP2	FAM3D
GAL	DHRS11	MACC1	PLN	SFRP2
CENPA	CDH22	CXCL8	CCBE1	ACTG2
ARID3A	ADHFE1	FADS2	AOC3	FOSB
LAPTM4B	KAT2B	SERPINB5	SYNPO2	HBB
UBE2S	SCGB2A1	SLC6A20	SP4	DES
HEATR1	DENND2A	IL11	CHRM2	KCNMB1
KIF26B	ANKRD20/	NMU	CLDN11	MYL9
RRP1	MRPL35	SCD	TNXB	HAND1
CPZ	EPB41L4A	SLC22A3	EPHA7	
COL8A1	AHNAK	COL1A1	NR3C1	
TMEM97	PCBP1-AS	DSG3	CHP2	
AXIN2	ITM2A	CEL	XKR4	
CMSS1	GNAQ	SULT1C2	PLCE1	
TMEM97	SSFA2	HS6ST2	CCL8	
ASIC1	CLCA1	FAP	GATM	
ASCL2	FAM134B	SGO2	KCNMA1	

CXCL2	RAB3C	DSC3	MPEG1
SLC5A6	RIMKLA	KRTAP21-	THRB
SAA2	ST6GALNA	HS6ST2	CCL19
GRAMD1A	ARRDC4	ENC1	NR3C1
CCDC85B	SCARA5	PRRC2C	PDE9A
LRRC20	SLC25A24	IBSP	AKAP12
XKRX	SSTR2	ACSL6	MT1M
WISP1	ANKRD12	TNFRSF12	KRT222
SNHG15	AHNAK	SERPINB7	PRKCB
PTTG1	ADH1A	MSLN	ENTPD5
ZNRF3	SLC9A9	SIX4	CLU
DIEXF	ACO2	SLCO1B3	LINC01279
PKMYT1	PDE6A	IL13RA2	MGP
TRMT112	LGALSL	FABP6	MS4A1
PLK1	NDRG2	ITGBL1	SLC4A4
FGF18	EFNA5	SULT1C2	PLN
GPR19	LGALS2	CXCL5	CR2
NTMT1	ABCG2	MACC1	FOXP2
MFSD12	SYTL4	MIR675	AKAP12
NEBL	STMN2	IL17A	P2RY1
POLR2D	PCK1	THBS2	ABCA9
CDC25C	C4orf19	ZIC2	S100B
TBC1D16	LIPH	WISP3	GLIPR2
RUNX1	P2RY1	CXCL5	TCEAL7
AHCY	MUT	PPBP	MPEG1
ASGR1	KLB	DUOXA2	CRYAB
PTTG2	PTPN21	HSPD1	PLCL2
MSX1	ANKRD20	DUSP27	SYNPO2
CENPH	HAGLR	CXCL8	NBEA
ACBD6	RHBDL2		PCDH9
CENPI	CDC42SE2		UCHL1
TOMM34	VAPA		PIK3CG
CENPO	GFRA3		GUCA2A
MIR503HG	KIAA1468		CNTN3
COL11A1	SRI		PDK4
NT5DC2	MON2		SYNPO2
PPAT	NEDD4L		GFRA1
DIO2	HERPUD1		ZBTB16
TMEM97	SLC35A1		NEGR1
ENOX2	SEMA3E		TMEM72
BYSL	KIAA1211		STAP1
GRHL3	GDPD2		SEMA6D
SMOX	P2RY1		NEXN
GZMB	LGI1		RERG
S100A11	ATP5A1		ACACB
PTTG3P	SEMA6A		OLFML1
OLFML2B	CHGA		FABP4
IL23A	HPGD		TMEM220
STRIP2	LIFR		BEX4
CDR2L	FAM46C		CHL1
NKD1	TMCC3		CMAHP
SLC22A3	SI		FAXDC2
KIF4A	SH3RF1		NAPSB
CDCA3	KIF16B		CD209
CCNB1	AP5M1		CCDC80
SHMT2	CTNND1		PTCHD1
PVT1	TMCC3		PTCH2
TRMT112	ADAM28		CFL2

FXYD5	C7	NR5A2
PUS7	INSL5	NAALAD2
EVA1A	BEST2	CD36
SPNS3	ITM2C	LMOD1
SHCBP1	ARHGAP44	TP73-AS1
ZNF587	TOX	SMIM5
TBRG4	ATP2A3	HPGD
GALNT6	COL2A1	SRI
SMYD3	GBA3	LIFR
ACD	BCL10	ADGRL3
TOP1MT	PRKAA2	PLCL2
BUB1	TNFRSF11A	IL6ST
LRRC6	ANKRD20A11P	MIR4680
ALDH4A1	TFCP2L1	RTN1
ARID3A	CCR2	EPHX2
SIGMAR1	KCNMA1	NR5A2
DDX10	PDZD3	EPHA7
KIF18A	ABCA5	HPGD
UTP4	CES3	HPGD
SORD	RMDN2	GHR
SKP2	SPON1	DMD
SPTBN2	TNFRSF11A	CCL21
GTF3A	HOXD13	KLHL6
FAM188B	KIAA1211	RNF150
GGCT	SIPA1L2	RERG
METTL26	EDN3	CLEC10A
CXCL1	GOLM1	MT1E
CHTF18	CPNE8	C14orf132
NXT1	STMN2	MMP28
FAM83D	HCFC2	CCDC80
LRP8	MAL	MS4A4A
ANKRD13I	SMPD3	FOXP2
MAD2L2	SDCBP2	FGF9
WDR74	HEPACAM2	KLHDC1
MTHFD1L	GALNT12	LRRK2
LAGE3	SPAG5-AS1	MIR4680
C16orf59	ACADM	LEPROT
SLC39A10	ABCA6	SMPDL3A
RRP9	KCTD1	TCEAL2
SLC7A6	NAAA	NEUROD1
SH3TC2	SMIM6	SYNPO2
SAPCD2	STXBP5	PBLD
DTYMK	C1orf186	CLECL1
FAM122B	NDRG2	CWH43
CCNE1	ECHDC2	ID4
TMEM9	KCNMA1	TARP
NHP2	KLF4	ADAMTS1
PSRC1	SCNN1B	BMX
CHTF18	SHROOM3	HAPLN1
FANCI	NXPE1	VSIG4
BOP1	CLIC5	EFEMP1
ABI2	FMO5	ADRB2
NMB	FZD5	OSBPL1A
THBS2	TSPAN3	SYNM
GPR4	SIDT1	SLC51B
POC1A	ST6GAL2	CPM
PYCR1	KITLG	AKAP12
NUF2	TMPRSS2	KLRC2

GADD45G	LGR4	MFAP5
CLPB	MBD1	ANO5
DSN1	NAPEPLD	IL6R
OSBPL3	RETNLB	ZEB2
UBE2I	MBNL3	TLR7
MCM10	CA1	SH3BGR
FAM92A1	SLC35D1	CCL23
RUVBL1	LDHD	FAM129A
CKS2	NXPE4	MBNL1-AS1
SSC4D	C15orf48	PPP1R12B
SH3PXD2E	LRMP	LOC100379224
WDR5	SCGB2A1	TMEM255A
CDT1	SLC35A1	AKAP12
CHI3L1	KLF3	SORBS2
OTUB2	NMNAT1	TLR3
CENPN	AKAP9	RNF125
CDK5RAP1	PTP4A1	SCN4B
LMTK3	TONSL	AKAP7
SLC5A6	PLD1	AGTR1
NANOS3	TLR7	DENND2A
B3GALT6	IL16	TXLNB
ARNTL2	B3GNT6	GNG7
PCID2	EXPH5	FGL2
STX1A	RBM47	MYLK
TICRR	NXPE2	COL14A1
RNF114	KRAS	TARP///TRGV9///TRGC2
H19	DRAIC	NKX2-3
GLA	F2RL1	MAOA
COL4A1	DAO	SLC30A10
MACC1	ATP8B1	CLU
RIPK2	IMPA1	CNN1
EXOSC5	CPNE8	MAOA
JAG2	ARL14	DTNA
TRPM2	PPP1R12B	SLC25A34
TYRO3	LOC100130741	KLRC4-KLRK1///KLRK1
MEST	AQP8	EFHC2
SOX9	DMRTC1	SLC26A2
EPSTI1	CP	PDE2A
PLEKHS1	BTBD7	SEMA6A
WDR4	FBLIM1	UG0898H09
FAM92A1	FAM134B	CNTN1
DTNB	EPB41L4B	MS4A7
CHAC1	PLCD1	MS4A1
PROCR	MBNL1	MAOA
TROAP	PRKACB	ID4
SPARC	ATP2A3	RFX6
PDCD2L	AKAP7	COLEC12
SLC4A11	FRMD3	SORBS1
KLK10	LOC102724760///IGHV1OR15-1	
DARS	FAM189A1	CMAHP
CDC45	PDE9A	FGF2
AGO2	B4GALT4	SCN3A
BFSP1	FGFR2	MT1E
CYP2B6	ISL1	ADAMTS1
ZNF695	KCTD12	HMGCS2
KIFC3	SLC16A9	CCL23
ASPHD1	KLF9	ENTPD5
ACSL6	SGSM1	CLDN23

PRC1	CHST5	DOCK10
MELK	KIAA1324	TMLHE-AS1
RNFT2	ALPI	CECR6
CFI	MTM1	GPAT3
WDR74	CDS1	GPR183
FAM72D	PCK1	C8orf88
CKS1B	NR3C1	TLR3
PPP2R3B	HCFC2	ZFPM2
GPT2	NEURL1	SLC17A4
NAA10	SLC35A3	NAP1L3
SMKR1	PLCD3	IGF1
CKS1B	FMN2	LMO3
ADRM1	CLIC5	UGT1A3
ECM1	FAM189A2	PBLD
CDCA4	IGHA2	FLVCR2
C9orf116	ANO7	HAND2-AS1
GTF2IRD1	BCL10	LRCH2
SFXN3	MT1M	BEST2
RNFT2	AKR1B10	IKZF1
DIAPH3	BMP2	CWH43
SLC6A20	ADTRP	PER1
NOTCH3	ARHGAP24	MT1F
P4HA3	FGD4	C8orf88
KIFC1	NEU4	EBF1
NOX4	ADGRA3	SCG3
CCNF	GUCA2A	SLC26A2
ZNF503	PPARGC1A	ADAM28
PSMC3IP	GREM2	MS4A2
DKC1	PEX26	TMEM47
HMGA1	PPP2CB	DSEL
NKRF	LAMA1	UGT1A1
ERCC6L	FAM126B	FAM49A
FXYD5	TMEM61	PDE1A
ALDH4A1	CDC42SE2	CNTN1
MLXIPL	C2orf72	CCDC69
SUV39H1	PPARGC1A	HMGCS2
FKBP10	RAVER2	ABCC13
CALU	BEND5	SOSTDC1
NELFCD	SPINK2	NCAM1
COL10A1	SSTR1	TCL1A
CHN1	BCAS1	PDE1A
BICD1	HPGDS	EML1
S100A11	AHCYL2	PKHD1L1
DHX37	EPHA10	PAQR5
EIF3B	CBX7	TRHDE
CXCL3	TMEM9B	CLIP4
CKAP2	TMEM61	CD48
ADRM1	PLS1	FAM150B
S100A3	DOCK10	TSC22D3
THY1	GNA11	MMP28
GRINA	RIMS4	IGLC1
CXCL3	TMEM171	DDR2
NAA11	CYCS	KLF4
FIGNL1	LOC105379426	EDN3
CDCA3	FRZB	MYOCD
SPHK1	FRAS1	FAM49A
CXCL2	GREM2	PCDH19
HIP1	BTNL3	RGS2

EXOSC4	GRAMD4	LONRF2
SULF1	SLAMF7	EPHA7
RAD54L	CES2	CITED2
FCGR3A	PRKACB	EIF1
OLFML2B	NPY	RSPO3
CA9	FBLIM1	ABCC13
NAT10	PDCD4	MSRB3
TTYH3	RASD1	CADM2
ADCY3	KIAA0513	UNC5D
GDPD5	SLC9A1	MS4A4A
TICRR	RTN4	DES
LRP8	TNFRSF17	IPW
UBE2T	FAM30A	NLGN1
PLOD3	CACNB2	TRPM6
LIMK1	GPA33	FXYD6
COPRS	SULT1A1	FILIP1
RELL2	HOXD11	NR1H4
EIF4EBP1	CYP2J2	MT1HL1
EBPL	FGFR2	DUSP1
PUS7	ADCY9	TARP
MMS22L	MALL	FCRLA
MACC1	HSD17B11	UGT1A3
TNNI3	SCIN	DPF3
GRPEL2	EPHX2	FGFR2
COL7A1	SLC26A3	MOGAT2
P3H1	CDC42	ACKR1
REG1A	SPPL2A	VPREB3
GRINA	FRZB	DES
AMOTL2	EPB41L3	UGT1A3
CENPN	CTH	KRT27
SIM2	FAM214A	FRMD3
BHLHE40	TRANK1	DDR2
HMGB3P1	CCNYL1	TRPM6
NFE2	NEDD4L	DCN
CARD14	NR3C1	BNC2
GATA2-AS	CCL28	FOXD4
LRRC8E	GPA33	ADGRL3
GJC3	UGT1A6	MB
BLACAT1	RELL1	ASB2
CCDC86	PAG1	MAP4
PRPS1	PARM1	ARHGAP44
TP53RK	RSPO1	MMP28
TTK	RAB3B	CACNB2
PKMYT1	AKR1B10	PTGS1
IL11	METTL7A	LYPD8
FMO3	ERICH5	ZNF677
PLS3	ECI2	UGT2B28
NONO	KIF13B	PTGS1
MCEMP1	TMEM30B	PRKCB
GALK1	CES2	UGT1A1
FCN3	ZNF575	CACNA2D1
TRAP1	PAPSS2	NPY1R
ADAM12	FCRL2	GPX3
COL8A1	B3GALT4	SCIN
MCM7	ASB2	COL4A5
DLGAP5	C1orf115	SLIT2
SIX1	CCR2	DCN
TCF3	SERTAD4	CYAT1///IGLV1-44///IGLC1

CSGALNACT1		TEX11
F2RL2	NMRAL1P1	MYLK
HJURP	RHBDL2	PDE4D
PROX1	CD1D	HSD17B2
EEF1AKMT	BHLHE41	CCDC69
POLD1	KLK1	NR5A2
MMP12	PTPRH	CYSLTR1
ARHGEF19	HHIP	NFASC
CENPF	DYRK2	CA2
FCHO1	MS4A12	CACNB2
STX1A	MCTP2	KLRB1
EGFL6	CHP2	AK9
FLVCR1	ZBTB16	CAPN13
RNF183	PANK3	RBMS3
CPT1C	ELL3	MT1X
ITGA11	FOXP1	EPB41L4A
FARP1	EPHA4	NEXN
FOXC1	ZFYVE28	BEX1
PRPS1L1	ZNF613	ACTG2
C2orf27A	NECTIN3	C1QTNF3
CEP55	MEF2C	SGCE
WNT5A	SPDEF	DUSP1
HGH1	SLC35D1	SLC17A4
S100A8	FSIP1	WNT2B
MSI2	SETBP1	TRAT1
TCFL5	LYPD8	FGF7P3
PSMB9	MALL	VGLL3
PROX1	KCNK1	CLDN23
ACSL4	BTNL3	EIF4E3
POLR1D	SRGAP1	PDK4
CEP250	NANS	PTGIS
NCAPD3	NEU4	RNF180
COL5A2	GOLM1	CES3
DACH1	IGF1	TUBAL3
DRAM1	SLC25A20	PDE5A
FIBIN	NDNF	P2RY10
ADGRF5	NBEA	BCAS1
CEBPE	ST3GAL4	FCRL1
MLXIPL	MFSD6L	BANK1
TRIM29	CLDN7	CD27
NCAPH	TLCD2	SCUBE2
RPP40	F13A1	TAS2R39
AQP9	PTPRF	MAB21L2
CCDC150	BCHE	SEMA6A
LYRM4	ANXA13	CES3
PRAP1	SLC45A3	ZG16
RHPN1	RHBDL2	TCF21
RELL2	TMEM59	EPHA3
XPOT	FRYL	GPX3
DUSP10	CFD	DNASE1L3
ZP3	GSG1	PDK4
CKAP5	PTGER4	TLR10
CSF3	PTAR1	SULT1B1
NELFCD	SLC17A4	CCBE1
NPEPL1	FCRL2	ISX
WNT3	FOXD2	GCSAM
LRRC32	TP53I3	CHRNA3
PFDN4	SETBP1	CADM2

SPON2	RDH5	MIER3
LYRM4	ZNF350	CFL2
KHK	RAB37	SLIT2
C11orf95	VSIG2	PCK1
ELOVL5	ANKRD20A2	INSM1
SGO1	NTRK2	MIER3
MCRIP1	TCEA3	SLC24A1
TDO2	FUCA1	PCDH10
PMEPA1	CKB	GULP1
POU5F1P3	SLC18A1	SCIN
ZDHHC9	KIF13B	MS4A7
SH2D2A	PLA2G10	MICU3
ADA	PAG1	CLCA2
ECE2	GON7	PPP1R16B
TBC1D16	CNTN1	RBPMS2
ASF1B	CRACR2A	CYBRD1
LAPTM4B	RAP1GAP	SLC26A2
TREM1	FABP2	SPG20
DPY19L1	FAM63A	LRRC19
C2orf70	GNPTAB	NR3C1
SLAMF9	SEPP1	BTNL3
PABPC1L	SGK2	ROR1
COL5A2	CARMIL1	SMIM14
ARHGEF39	CYP3A7	JAM3
C4BPA	IMPA1	LMO3
C2orf70	MB	PID1
MYEOV	LOC643201	MS4A1
POC1A	RILP	ENPP2
CTPS1	PAQR5	RUNDC3B
MTFR2	SEC11C	TAS2R41
CHEK1	KRCC1	ZEB1
DGKZ	MT1E	GPHA2
ECT2	PRKAR2B	TMEM37
SRGAP2	FAM3C	MT1G
CLEC4E	ALDH6A1	CAP2
CPXM1	RNASE4	LINC01467
SPC25	GCNT3	FOXD3
C1QTNF5	MCTP2	FCRLA
CFI	RIOK3	WWTR1
EPSTI1	RHOU	KCTD12
MMP12	RIMKLA	TNFRSF17
PARPBP	EPHA4	PPP1R16B
NPRL3	PBLD	HHLA2
NME1	ANK3	MYOCD
SMKR1	VIT	UGT2B15
SH2D5	SLITRK6	PTPN22
C20orf27	PRPH	CXCL13
RCN1	BLK	THBS1
RIPPLY3	PLAC8	ADAM28
RPUSD1	MYOF	KIAA1211
KAT2A	SPTLC3	TMEM171
ASAP1	AGR3	CCL13
DUSP4	PTPRN2	PDLIM3
RNF183	NTN1	ATRNL1
NEK2	EXPH5	SEPP1
CSF2	SAMD9	BANK1
APOLD1	SULT1B1	ZC3H12C
AURKB	KLRC1	IGLV1-44

STRAP	PINK1	CCDC80
GLS2	CAPN9	ITIH5
RILPL1	SGSM3	KLF9
POU5F1	ZFYVE28	MIER3
PSAT1	SOAT1	SNRK-AS1
DIO2	PROM2	GFM1
NLE1	C11orf86	CSRNP3
NPM3	MT1L	FBLN1
LRRC8E	COL4A6	NAT2
UBD	RCAN1	NXPE1
HGF	VIPR1	LINC00462
CXCL5	CCL23	CYAT1
IL24	C2orf40	CD163L1
ALDH4A1	CCDC85A	FRMD3
SPC24	IL6R	BMS1P20
CDC20	FAM3D	IGLJ3///CKAP2
CLDN14	CLEC10A	SSSCA1-AS1
MET	IPO5P1	CLCA2
C1orf112	TRAF3IP3	UGT2B15
KLHL35	EPB41L4B	MSRB3
CDT1	PNOC	NAPSB
DEF8	JAM2	PPP1R14A
CMTM7	3-Mar	TMEM47
ECT2	CDHR5	IGLJ3
CCNB2	PBLD	ACSM3
BLM	CMBL	SEPP1
SCD	CLCA4	SI
ORC6	SLCO2A1	TTLL6
MCM2	SCG5	IGLC1
CFB	ACAT1	CCL5
STRA6	FCRLA	COL28A1
SLC7A5	KLRC4	CEBPD
KRT17	UGT2B10	B3GALT5
RACGAP1	MUC2	SLIT2
GOLT1A	BMP6	CLCA1
WDR4	MYO1A	IGKC
OTX1	NOSTRIN	DNAJB4
CTSK	HOXB13	CA1
MSX1	SLC22A23	APOD
MNX1-AS	C1orf132	C8orf4
C9orf16	CAMK1D	MSH6
NNMT	CHST6	UGT2B15
TMEM158	WFDC2	CPM
SLC2A8	GLIPR2	SLC16A7
NSUN5	CD36	CAPN13
FPR1	ERN2	BACH2
IL24	WFDC2	TOX
CAD	TPM1	SLC26A3
MACC1	RBFOX1	GPR34
S100A9	C9orf24	OSBPL1A
PLXNA1	PPIC	ST8SIA1
RRM2	GDPD3	HEPACAM2
DHDH	ZC3H12C	CCL2
CKAP2L	TSPAN13	FAS
GINS1	FAM46A	MS4A8
POLR1D	TRIM36	MRGPRF
SOCS1	SEMA4G	ALDH1A1
PLAGL2	PARM1	EMP1

CELSR3	DENND2A	LOC100507073
LIPE	MUC12	FBLN1
KCNJ15	ZSCAN18	PADI2
BORA	TMEM246	PDE7B
ZNF469	CHODL	CD8A
CCDC3	PLAC8	HHLA1
SERPINE2	IGSF9	BTNL8
DEF8	TNXB	IGLJ3
RTEL1	MYH11	KIAA1654
PARVB	PLEKHA7	ACVR1C
PACSIN3	CA4	CA4
CLEC11A	SLC44A4	LAMA1
CENPJ	CRYM	TNS1
KIF18B	OIP5-AS1	SDCBP2
COL9A3	C14orf159	NAALADL1
NOTCH4	CPNE5	CD177
TPD52L1	CALM1	PMP22
MTHFD1L	GPRIN2	IGLJ3
SOD2	SMPDL3B	MFSD4A
EIF6	CYP4V2	TACR2
TMCC1	KIAA1522	BMP2
PLSCR3	MCOLN2	EMP1
FADS1	COX6B2	ATP1A2
DDIT4	KRAS	P2RY10
FADS1	UBE3A	RAB39B
CEBPB	WNK4	FCGBP
SSSCA1	FGF9	MYH11
TMEM160	PARM1	LINC01222
SLCO1B3	NOV	DNAJB5
TMEM74B	KBTBD12	NEUROD1
FTSJ1	LOC100507053	IGLV1-44
CITED4	PIGZ	GPAT2
PML	SSPN	BMS1P20
KLK8	FHL1	ADARB1
FAM19A5	MYH3	WDR78
CXCR2	LTK	TTC28
GINS2	FZD5	SPON1
S100A9	LINC00261	DSEL
GJA4	FOXD2	CA4
SORD	JCHAIN	DHRS9
RAI14	PIGR	FOXP2
SLC11A1	ANPEP	CDKN2B-AS1
DNMT3B	CACNA2D2	MOGAT2
AXIN2	TMED6	SCNN1B
PGC	EPN3	ZBTB7C
EGFL7	ZNF254	RBFOX3
SLC7A1	HIST1H1C	IGK///IGKC
FAM3B	MUC4	PCAT7
ALDH1B1	LINC00597	GZMK
TWIST1	HOXB13	RGS1
PHGDH	ALDH1A1	ANPEP
ERO1A	C1orf115	TNS1
PODXL	BMX	CA12
CDCA8	CD177	LOC100509457///HLA-DQA2///HLA-DQA1
HOMER1	CYP4F12	SLC9A2
KRT6B	FOLR2	IGLL5
BGN	ITLN1	FRMD3
ZDHHC9	SATB2	MFSD4A

FOXRED2	AGR2	PRKACB
PRKDC	RNASE1	STON1
POP1	AOC1	ADRA2A
TFDP1	MUC13	DCN
YDJC	TMEM246	SPON1
TRPM2	MYOM1	SORBS2
SPERT	ARSJ	IGLV1-44
THEM6	FCGBP	HSPB6
SGIP1	ACVRL1	EFNA5
PHLDA1	TMC8	RBMS3
NOLC1	HK2	GEM
TOP2A	NLRC3	PTPN22
PFKFB3	GNG2	PAX5
ADGRL4	KAZALD1	CR1
MCM4	SIAE	PTCHD1
POU5F1P4	CD177	RGS1
MAT1A	FOXA1	VSIG2
IFFO2	GALNT8	IGLV1-44
STC2	RARRES1	PWAR6
TRIP6	CEACAM1	SLC26A3
PLEKHN1	MT1G	LINC00317
HYAL3	CAMK2N1	IGK///IGKC
PAQR4	ITPKA	POPDC2
MX2	CTSE	PTX3
SORD	ZNF493	CHD1L
ASB9	CD19	CPA3
PAM16	B4GALNT3	
PORCN	DES	TPSB2///TPSAB1
SLC43A1	CXCR5	TPSAB1
TNFRSF10	ACSM3	CLIC5
MZT1	CD79A	IGHV4-31///IGHM///IGHG3///IGHG2///IGHG1///IGHD///IGHA2//
IL17D	SLIT2	IGLJ3///IGLV1-44///CKAP2///IGLV@///IGLC1
PLPP4	FGL2	PCSK5
TNFRSF10	ZG16	ITIH4
SCML1	MYH11	B3GALT5
NSMF	MROH7	CYP2C18
HTRA3	BEX4	CD37
OSM	DNAJC12	CA12
CCNO	IGKC	TPSB2
CENPW	TRPM4	DHRS9
RPS2	MT1X	UGT2B17
FCGR3B	IL37	CD69
KIAA0226L	MAOB	CNGA3
GAD1	CCL19	TRDV3
CDCA7	CD27	CCL5
TACSTD2	TTC39A	
ANKRD2	NAT2	TPSB2///TPSAB1
MCM10	KLRC2	HTR4
VWCE	RASA4	TPSAB1
GLYATL1	FAM101A	TPSAB1
DUSP2	ACVR1C	HSPB7
MIR17HG	SYTL5	SNORD114-3
LEF1	FAM129C	FERMT2
ASPM	HOXD9	IGLJ3///CKAP2///IGLV@///IGLC1
GNG4	PDZRN3	COL21A1
TUBB4A	HES2	SFRP2
FITM2	MS4A1	CLCA4
CDKN3	FGFBP1	GPR18

APOBEC1	MT1B	PLPP3
THOC3	PPP1R9A	TMEM37
VEGFA	FDCSP	MGC24103
ARNTL2	CILP	TPSAB1
EVPL	KRBOX1	GPR162
ONECUT2	ATMIN	FMO5
GTSE1	HOXA13	IGK///IGKC
FARP1	HMGCS2	HOPX
B9D1	HSPA2	IGKC
FTSJ1	GSN	MS4A12
WNT5A	CDHR1	TRBC1
UTP14A	SYT13	IGK///IGKC
LYPD1	SMCHD1	TNS1
PPM1N	IGHV3-48///IGHV3-6	C17orf64
BMP8A	MEP1A	CDHR5
CREB5	UGT2B7	IGK///IGKC
EDNRA	SHD	HSD3B2
COL6A3	IGLV6-57	LOC101929272
GABRE	WISP2	IGKC
CKS1B	CXCL13	SPON1
BUB1B	TCL1A	DNALI1
AFAP1L1	RGMA	ZAN
ZFAS1	UGT2B17	C10orf99
MME	IGLL5	SPON1
SRXN1	EMP1	FOXN3
RCN1	MST1	LOC100131662
VEGFA	HES5	FMN2
FNDC1	MST1	SLAMF7
OAS3	CLGN	IGLJ3
ECSCR	UGT2B17	PLAC8
MND1	MUC4	IGKC
FAM210B	BMS1P20	ST6GALNAC1
CXCL5	MT2A	B3GALT2
CCND1	IGHG1	YME1L1
PHLDA1	GSTA2	IL1R2
LTBP2	MT2A	PAPSS2
ALG3	PRAC1	RIOK3
EPDR1	IGHV3-69-1///IGHV3	CNKSR2
PTK7	MT3	TINAG
BATF3	GBP3	PALMD
PFKFB3	CELF3	IGKC
HTRA3	DEFB1	IGKC
HOXB8	GSTA5	MEP1B
DKK2	DQX1	FOS
PTGS2	IFITM5	CD52
GRB10	PCSK1N	CCL28
TSPAN5	DACT2	ABCC13
HCAR3	PHOX2A	VIM
SH3TC2	PRAC1	FXYD3
OAS3	SMARCC2	CYP2C9
ANGPTL2	NPTN	LOC102723479
INHBB	DPT	XDH
SP5	FCRLB	SLC23A1
CDC25A	ZNF467	POU2AF1
NAMPT	IGLL5///CYAT1///IGL	MGC40069///TRAC///TRAJ17///TRAV20///TRDV2///YME1L1
VMA21	IGFALS	IGLJ3
FSTL3	TSPAN10	IGKV1OR2-108
POU5F1	CACNA1E	CAPN9

PLIN2	NKD2	CHST5
SNHG17	LEFTY1	SLC8A1
KRT14	C17orf96	MIR100HG
C7orf50	PSORS1C2	NPTX1
KLK6	IER5	SLAMF7
CRNDE	MEX3D	CPA6
EIF5A2	PRR7	RHOJ
ALG3	SNORA78	TRAC
ASPM	CEBPA	MLIP
LPL	POU3F3	TRBC1
TRIM29	TFF1	ITGA9
KIF21B	LOC101928068	NR5A2
ROBO4	MUC17	NEFL
CASC15	ZSCAN10	CHST5
MSX2P1	REG4	BRINP3
KRTAP4-1		ENTPD5
KIF15		ZDHHC18
DNM1		SLITRK6
KRT6A		IGK///IGKC
TREML2		PLA2G5
ERFE		IGHV3-23///IGHV4-31///IGHM///IGHG1///IGHA1
CXCL6		CLEC2B
TCN1		MBNL1
KIAA1462		PTPRC
MCM8		ENPP3
ZNRF3		LTF
SFRP4		MYL9
PITX1		VSIG2
ERP27		B3GNT7
CNIH3		TAGLN
VWA1		IGLC1
POU5F1		RIOK3
DUSP5P1		CEACAM7
FEN1		CYP2C9
MGC32805		C10orf99
HAS3		ENPP3
SCD		CEACAM7
CXorf36		A1CF
SNRPB		GZMA
MX2		IGK///IGKC
REG3A		MEIS1
DUSP5P1		DDR2
CEACAM6		BPNT1
SLC11A1		SLC16A9
PRSS23		ITLN1
KYNU		FST
ISG15		RNF186
GAS2		ADRB1
ZNF593		MFSD4A
CYR61		CCR7
SLC1A7		EPHA4
NINJ1		PENK
REG1B		PIP5K1B
IL32		SMIM14
NES		YME1L1
CD93		MEP1A
SERPINH1		FOSB
LGALS7		IGHV4-31///IGHM///IGHG3///IGHG1///IGHD///IGHA2///IGHA1

CDCA7	MESP1
ATP6V1C2	SMYD1
PLA2G16	ZSCAN4
NOLC1	PRKAA2
CYR61	SLC13A2
POLQ	KRT20
UCA1	CA12
SHC3	IGHV3-23///IGHV4-31///IGHM///IGHG3///IGHG1///IGHD///IGH
NOP56	IGHV4-31///IGHA1
ETV7	
MROH6	SLITRK6
LEMD1	UBXN10-AS1
SLC11A1	FABP1
KIF23	IGHV4-31///IGHM///IGHG4///IGHG3///IGHG1///IGHD///IGHA2//
MC1R	IL1R2
HTRA1	
PYCR1	LOC102725526///IGHV4-31///IGHM///IGHG3///IGHG2///IGHG1/
ISLR	IGK
MME	IGLL1
PCDH17	HLA-DRA
CXCR1	INF2
GDF15	MZB1
STMN3	TSPAN1
TSNARE1	PIGR
RRM2	IGHV3-23///IGHV4-31///IGHM///IGHG1///IGHA1
ADAMTS4	IGLC1
ANGPTL2	CASP5
DKK3	CDHR5
MYCN	IGHM///IGHG1///IGHA1
CRABP2	SCGB2A1
MAD2L1	
NTNG2	HLA-DPA1
SLC27A5	IGHV4-31///IGHM///IGHG3///IGHG1///IGHD///IGHA1
TIMM8A	IGKC
CXCL8	ALPI
CXCL10	ADIPOQ
MYOM3	IGHV4-31///IGHM///IGHG3///IGHG1///IGHA1
MFSD13A	IGHV4-31///IGHM///IGHG4///IGHG3///IGHG1///IGHD///IGHA2//
LAIR2	IGHM///IGHG1///IGHA1
HDGF	EPHA4
CKMT2	
INHBB	GPA33
DNAH2	NTRK2
WFS1	KCNJ13
PXMP4	SVIL
BMP7	
FPR2	TTN
TGM2	EYA2
CXCR2P1	OTC
DYSF	CASQ2
S100A12	F2RL1
EVA1B	CYP3A4
TNC	LOC100293211
FRMD5	PTPRC
DNAJC15	IGHM
NAMPT	
DHCR7	CALD1
NCAPG	

BACE2	CLDN8
FGFRL1	IGHM///IGHG1
CXCL11	TJP2
MLLT11	IGLL3P
IL13RA2	AGR3
CARHSP1	IGHM
PLCB1	HLA-DRB4
VEGFA	RETNLB
TIMM8A	IGHG1///IGHD///IGHA2///IGH
E2F7	MUC4
MIF-AS1	SOX2
SLC13A3	
IFI6	
LILRA3	IGHM///IGHG1
ONECUT2	IGHM///IGHG1
TGFB2	
PIR	
TCF15	ITSN1
GUCY1A3	C1S
PIPOX	MUC4
PCDHB11	HAND2-AS1
LBH	LOC101060835///HLA-DQB1
CYR61	CYP3A4
MCAM	SERPINA3
CDC6	SRGN
KLHL29	TAC1
ADRA2C	THBS4
FSTL1	IGHA2///IGHA1///IGH
CBS	SLC28A2
SNX5	CALD1
NCS1	TXNIP
FBXL2	PARM1
ITGBL1	CDHR5
TNFRSF12A	CEACAM7
ZAK	MUC4
SELE	
ARG2	TNC
COL4A2	GAS1
SP5	FGF21
ITGA5	LOC102723407///IGHV4-31///IGHM///IGHG4///IGHG1///IGHA2/
COMP	PDZK1
FOXC1	NTS
APCDD1	
NCOA7	
AQP5	
KCNJ8	
FAM20C	
MEDAG	
LMCD1	
NR1D1	
ODAM	
DKK3	
LGALS1	
SCAND1	
AGMAT	
PSPH	
ACSL1	
CAB39L	

CMTM2
RPS2P45
PDGFRB
BACE2
GNG4
EPB41L2
NAMPT
MAP7D2
FBXO2
CGREF1
NOTUM
VCAN
SOX11
PROK2
G0S2
GUCY1B3
TIMM17B
CDH13
SFTA2
DUSP2
CALCA
RASIP1
ENC1
SOCS3
LUM
IL33
CST1
ANGPTL4
POLQ
CSF3R
GLT1D1
IER3
TNFAIP6
MNDA
RGCC
LRRC36
GYG2
LIN7A
TNIP3
PRSS23
SNTB1
CDH11
IFIT3
IRAK1
SSUH2
PRSS23
UNC5CL
LILRB3
TNNT1
SLC39A4
CCL3
CLDN2
CD248
LZTS1
KIAA1257
MSX2
VASN
STMN3

POU5F1
DAPL1
EREG
LRRC15
COL5A1
MICB
PTPN13
FCN1
GMPR
PRSS33
BCAT1
ABCC2
FCN1
S100A4
BATF
DHRS2
SERPINB2
B4GALNT4
DEFB4A
IGF2
FCGR2A
CXCL11
EREG
LOXL1
CDKN2A
PLCB1
MUCL1
CCL4
GINS4
CCL3L3
PRKCDBP
COL5A1
THBD
MCAM
PHLDA1
ALDOB
SCRN1
TNNC2
LRRC34
COL3A1
PXDN
DUSP27
FILIP1L
CDCA2
GALNT18
BMP7
ZP3
ALDOB
NETO2
SLC30A2
NCOA7
KLK7
DSG3
SLC2A3
KRT6C
DHRS2
SPACA3
SOD2

BCL2A1
STMN3
HCAR1
FUOM
FOLH1
DEFA3
CADPS
GRP
FREM1
CCL20
SPRR2C
NCF2
IDO1
RGS5
HSD11B1
PGGHG
C4BPB
IGFBP5
ORM2
IGFBP5
RNF182
CYP4X1
COL3A1
SERPINB4
FGGY
C2CD4B
SERPINB3
FBN1
CXCL9
HSPC102
CACNA1C
LCN2
AREG
BMP7
ORM1
IFI44L
COL18A1
REEP6
FADS2
SERPINB5
SFTA2
CD55
SLC2A14
GRM8
IL4I1
MXRA8
CYP4X1
ACTC1
ACE2
BST2
C10orf82
NPC1L1
COMP
CTSV
IL1RN
TMPRSS5
MARCO
C6orf15

GBP5
SEC14L4
IGF2BP3
PCSK9
C11orf96
MX1
PLA2G3
PPBP
KRTAP5-8
WBSCR27
DUOXA2
COL15A1
GFPT2
PNPLA3
FGGY
CCL3L3
WIF1
SFRP2
CHRDL2
REG3G
GAS1
HS6ST2
FZD10
SBSPON
WNT11
COL3A1
PRAME
NPTX2
CCL11
NMU
LRP4
FOLR1
SNHG17
SFRP2
CHRDL2
DMBT1
PSPH
SMPX
PI3
DEFA6
DEFA6
IGF2
DEFA5
HBA2
MAGEA2B
OLFM4

datasets.

//IGHA1///IGH

A1///IGH

///IGHA1

///IGHA2///IGHA1///IGH

///IGHA1///IGH

'//IGHA1
