# Supplementary material for: The Underlying Roles of Exosome-Associated PIGR in Fatty Acid Metabolism and Immune Signaling in Colorectal Cancer
Source: J Oncol. 2022 Sep 15;2022:4675683. doi: 10.1155/2022/4675683 (PMC9499750; doi:10.1155/2022/4675683)
Supplement: Supplementary Materials — Supplementary Table 1. The upregulated genes and downregulated genes in the three GEO datasets. Supplementary Table 2. The coexpressed genes possess a positive and negative relationships with PIGR. Supplementary Table 3. The top 20 genes positively correlated with PIGR in colorectal cancer. Supplementary Table 4. The top 20 genes negatively correlated with PIGR in colorectal cancer. [file 4675683.f1.zip › 4675683.f1/Supplementary Table 2.pdf]

Supplementary Table 2. The co-expressed genes possessing positive and negative relationsh

Query	Statistic	P-value	FDR (BH)
GDPD5	-0.43427	7.28E-19	2.26E-16
PCMTD2	-0.43223	1.1E-18	3E-16
PAN3	-0.42507	4.63E-18	1.02E-15
ASXL1	-0.42465	5.03E-18	1.1E-15
CHD6	-0.42267	7.43E-18	1.57E-15
C11orf95	-0.42104	1.02E-17	2.08E-15
KBTBD6	-0.4185	1.68E-17	3.2E-15
PLCG1	-0.41631	2.55E-17	4.64E-15
ETNK2	-0.41414	3.88E-17	6.86E-15
SLC2A12	-0.41274	5.05E-17	8.71E-15
BCAM	-0.41005	8.41E-17	1.39E-14
PCIF1	-0.4084	1.15E-16	1.83E-14
ZNF251	-0.40491	2.19E-16	3.37E-14
WBP4	-0.40412	2.54E-16	3.84E-14
GNAS	-0.40361	2.79E-16	4.16E-14
SORBS1	-0.40052	4.91E-16	6.96E-14
PHLDB3	-0.40001	5.39E-16	7.53E-14
ZMYM5	-0.39883	6.67E-16	9.12E-14
HSDL1	-0.39844	7.17E-16	9.74E-14
PRPF6	-0.39811	7.61E-16	1.02E-13
RBM39	-0.39334	1.79E-15	2.16E-13
LTBP3	-0.39318	1.84E-15	2.21E-13
ADNP	-0.39215	2.21E-15	2.62E-13
TPD52L2	-0.39143	2.51E-15	2.91E-13
RBMS1	-0.39114	2.64E-15	3.01E-13
N4BP2L2	-0.38545	7.15E-15	7.57E-13
COL9A3	-0.38542	7.18E-15	7.57E-13
LY6G6E	-0.38239	1.21E-14	1.26E-12
ZNF517	-0.38183	1.33E-14	1.36E-12
THSD1P1	-0.37929	2.05E-14	2.02E-12
ZDHHC17	-0.37835	2.41E-14	2.33E-12
ELMO2	-0.37821	2.46E-14	2.37E-12
ZNF34	-0.37785	2.62E-14	2.49E-12
FGF18	-0.37777	2.66E-14	2.51E-12
STX16	-0.37668	3.19E-14	2.97E-12
LASS5	-0.37659	3.24E-14	3E-12
KLHDC5	-0.37651	3.28E-14	3.03E-12
MBNL2	-0.37388	5.1E-14	4.59E-12
GRIN2C	-0.37321	5.7E-14	5.09E-12
USPL1	-0.37287	6.03E-14	5.33E-12
FAM48A	-0.37266	6.24E-14	5.45E-12
E2F6	-0.37264	6.26E-14	5.45E-12
STK4	-0.37215	6.79E-14	5.88E-12
C6orf48	-0.37101	8.2E-14	6.86E-12
TMEM198	-0.37072	8.6E-14	7.17E-12
ZNF696	-0.36998	9.72E-14	8.06E-12
PTK7	-0.36948	1.05E-13	8.64E-12
WWC2	-0.36877	1.18E-13	9.66E-12
C20orf43	-0.36847	1.24E-13	1.01E-11
RCBTB1	-0.36843	1.25E-13	1.01E-11
NAA16	-0.36817	1.31E-13	1.05E-11
NPTXR	-0.36724	1.52E-13	1.21E-11
SPEG	-0.36654	1.7E-13	1.33E-11
ZNF623	-0.36615	1.81E-13	1.41E-11
GLS	-0.36605	1.84E-13	1.42E-11
PLAG1	-0.36596	1.87E-13	1.43E-11

DPY19L4	-0.36591	1.89E-13	1.44E-11
F10	-0.36587	1.9E-13	1.44E-11
PMEPA1	-0.36576	1.93E-13	1.46E-11
SLC39A10	-0.36555	2E-13	1.5E-11
ASAP1	-0.36504	2.17E-13	1.62E-11
C20orf177	-0.36489	2.22E-13	1.64E-11
PUM2	-0.3646	2.33E-13	1.72E-11
ZNF425	-0.36395	2.59E-13	1.89E-11
AMOTL2	-0.36385	2.63E-13	1.92E-11
CDON	-0.36382	2.64E-13	1.92E-11
SBK1	-0.36364	2.72E-13	1.97E-11
TSPYL2	-0.36274	3.14E-13	2.25E-11
SP3	-0.36129	3.96E-13	2.77E-11
ZCCHC14	-0.35982	5E-13	3.43E-11
CES4	-0.35916	5.56E-13	3.77E-11
ABI2	-0.35894	5.75E-13	3.89E-11
ZNF7	-0.35893	5.76E-13	3.89E-11
ZNF250	-0.35843	6.23E-13	4.19E-11
NFAT5	-0.35826	6.4E-13	4.29E-11
ACVR2B	-0.35809	6.57E-13	4.39E-11
HDAC5	-0.35788	6.8E-13	4.51E-11
CLASP1	-0.35784	6.84E-13	4.52E-11
NCOA6	-0.3576	7.11E-13	4.67E-11
PAQR7	-0.35744	7.29E-13	4.77E-11
USP11	-0.35741	7.32E-13	4.77E-11
CSNK2A2	-0.35727	7.48E-13	4.86E-11
SOX4	-0.35714	7.63E-13	4.94E-11
C20orf203	-0.35634	8.65E-13	5.52E-11
ZMYM2	-0.35624	8.8E-13	5.59E-11
TRPC4AP	-0.35588	9.31E-13	5.88E-11
RPRD1B	-0.35545	9.95E-13	6.24E-11
C20orf11	-0.35522	1.03E-12	6.44E-11
RNF219	-0.35392	1.26E-12	7.72E-11
DHX35	-0.35368	1.31E-12	7.99E-11
L1CAM	-0.3521	1.67E-12	1.01E-10
HOOK3	-0.351	1.98E-12	1.18E-10
DCUN1D2	-0.35011	2.27E-12	1.34E-10
SERINC3	-0.34962	2.45E-12	1.44E-10
GLB1L	-0.34933	2.56E-12	1.49E-10
FLJ22536	-0.34876	2.79E-12	1.6E-10
ATP11A	-0.34769	3.29E-12	1.86E-10
ZNF333	-0.34751	3.38E-12	1.9E-10
ZNF322A	-0.34704	3.62E-12	2.02E-10
CDKN1C	-0.34693	3.68E-12	2.05E-10
ZSCAN21	-0.34659	3.88E-12	2.15E-10
HABP4	-0.34643	3.98E-12	2.2E-10
MPPED2	-0.34633	4.04E-12	2.22E-10
NCK2	-0.34588	4.32E-12	2.37E-10
MYL6B	-0.34502	4.92E-12	2.67E-10
C20orf117	-0.34484	5.06E-12	2.74E-10
TGIF2	-0.34478	5.1E-12	2.75E-10
NCOA5	-0.34341	6.27E-12	3.36E-10
LOC10028	-0.34311	6.55E-12	3.5E-10
LOC38869	-0.34298	6.68E-12	3.56E-10
RNF146	-0.34287	6.79E-12	3.61E-10
PHF20	-0.34276	6.91E-12	3.66E-10
TCFL5	-0.34274	6.92E-12	3.66E-10
C11orf84	-0.34189	7.85E-12	4.12E-10

RAB22A	-0.34178	7.99E-12	4.18E-10
YAP1	-0.3415	8.33E-12	4.35E-10
CTNNBL1	-0.34104	8.92E-12	4.64E-10
C13orf15	-0.34077	9.27E-12	4.79E-10
ZNF707	-0.34055	9.59E-12	4.93E-10
PDS5B	-0.34022	1.01E-11	5.15E-10
LY6G6F	-0.3399	1.06E-11	5.35E-10
LDLRAD3	-0.3388	1.24E-11	6.22E-10
LSM14A	-0.33878	1.25E-11	6.22E-10
DGCR5	-0.33859	1.28E-11	6.36E-10
ZFHX3	-0.33844	1.31E-11	6.47E-10
NXPH3	-0.33819	1.36E-11	6.68E-10
RAB11FIP2	-0.33779	1.44E-11	7.04E-10
FAM70B	-0.337	1.62E-11	7.89E-10
NKD2	-0.33688	1.64E-11	8.01E-10
ZNF12	-0.33663	1.71E-11	8.29E-10
LCA5	-0.33661	1.71E-11	8.29E-10
RALGAPB	-0.33651	1.74E-11	8.4E-10
C20orf4	-0.33629	1.79E-11	8.58E-10
ARFGAP1	-0.33619	1.82E-11	8.68E-10
DLGAP4	-0.33609	1.84E-11	8.79E-10
C21orf34	-0.33582	1.92E-11	9.06E-10
TERF2IP	-0.33526	2.08E-11	9.69E-10
CENPBD1	-0.33502	2.16E-11	9.97E-10
ZGPAT	-0.33464	2.28E-11	1.04E-09
ZC3H13	-0.33374	2.6E-11	1.18E-09
PTPN12	-0.3336	2.65E-11	1.2E-09
CES1	-0.33357	2.66E-11	1.2E-09
CCND1	-0.33346	2.7E-11	1.22E-09
LOC28576	-0.33344	2.71E-11	1.22E-09
AKNAD1	-0.33334	2.75E-11	1.24E-09
NES	-0.33297	2.9E-11	1.29E-09
ZNF335	-0.33266	3.03E-11	1.35E-09
SALL4	-0.33263	3.04E-11	1.35E-09
C16orf52	-0.33247	3.11E-11	1.38E-09
CAMSAP1	-0.33225	3.22E-11	1.41E-09
RAE1	-0.33216	3.26E-11	1.43E-09
EPC2	-0.33212	3.28E-11	1.43E-09
RB1CC1	-0.33201	3.33E-11	1.45E-09
PHACTR3	-0.3319	3.38E-11	1.46E-09
HIF3A	-0.33172	3.47E-11	1.5E-09
KCTD7	-0.3317	3.48E-11	1.5E-09
AGTR1	-0.33104	3.83E-11	1.64E-09
F7	-0.33097	3.87E-11	1.65E-09
BBS10	-0.33094	3.88E-11	1.65E-09
CEBPB	-0.3307	4.01E-11	1.7E-09
CCT6P1	-0.33053	4.12E-11	1.74E-09
STAU2	-0.33042	4.18E-11	1.77E-09
TMTC4	-0.3302	4.31E-11	1.82E-09
ITCH	-0.32956	4.72E-11	1.97E-09
DNTTIP1	-0.32947	4.78E-11	1.98E-09
POFUT1	-0.32913	5.03E-11	2.07E-09
JMY	-0.32908	5.06E-11	2.08E-09
KIF5A	-0.32903	5.1E-11	2.09E-09
PMS2CL	-0.32901	5.11E-11	2.09E-09
SOCS5	-0.32901	5.11E-11	2.09E-09
NALCN	-0.32893	5.17E-11	2.1E-09
NRK	-0.32862	5.4E-11	2.16E-09

LOC10012	-0.3286	5.41E-11	2.16E-09
ANKRD46	-0.32851	5.48E-11	2.19E-09
SDC4	-0.32759	6.25E-11	2.48E-09
KBTBD2	-0.32747	6.35E-11	2.52E-09
LOC72885	-0.32715	6.64E-11	2.62E-09
DNASE1	-0.32696	6.82E-11	2.68E-09
MAP6	-0.32694	6.84E-11	2.69E-09
RMND5A	-0.32627	7.53E-11	2.94E-09
NODAL	-0.32624	7.56E-11	2.94E-09
C7orf52	-0.32593	7.89E-11	3.06E-09
TECTA	-0.3258	8.03E-11	3.11E-09
SNX21	-0.32524	8.7E-11	3.36E-09
WNT11	-0.32523	8.7E-11	3.36E-09
SUGT1L1	-0.32519	8.75E-11	3.37E-09
FOXO3B	-0.32487	9.15E-11	3.51E-09
SCN5A	-0.32469	9.38E-11	3.59E-09
TSNARE1	-0.32456	9.57E-11	3.65E-09
AKAP8L	-0.32409	1.02E-10	3.89E-09
TMEM55A	-0.3238	1.06E-10	4.04E-09
SETDB2	-0.32367	1.08E-10	4.1E-09
RBM26	-0.32356	1.1E-10	4.15E-09
BCL2L1	-0.32342	1.12E-10	4.22E-09
DRD2	-0.32339	1.13E-10	4.23E-09
LOC65227	-0.32336	1.13E-10	4.24E-09
DIDO1	-0.32317	1.16E-10	4.34E-09
ARHGAP2:	-0.32315	1.16E-10	4.35E-09
ATP6V1C1	-0.32313	1.17E-10	4.35E-09
NOTUM	-0.32308	1.18E-10	4.37E-09
TCEA2	-0.3229	1.2E-10	4.46E-09
STAU1	-0.32269	1.24E-10	4.57E-09
KIAA1467	-0.32252	1.27E-10	4.66E-09
STRA6	-0.32251	1.27E-10	4.66E-09
ZAK	-0.32227	1.31E-10	4.81E-09
RNF6	-0.32199	1.37E-10	4.99E-09
ELF1	-0.32182	1.4E-10	5.09E-09
OSGIN2	-0.3218	1.4E-10	5.09E-09
ZFP41	-0.32152	1.46E-10	5.27E-09
ADAMTSL:	-0.32139	1.48E-10	5.35E-09
CBX4	-0.32103	1.56E-10	5.6E-09
LARP6	-0.32091	1.59E-10	5.68E-09
C6orf15	-0.3208	1.61E-10	5.74E-09
IFT52	-0.32033	1.72E-10	6.04E-09
AKAP6	-0.3203	1.73E-10	6.06E-09
NKD1	-0.31978	1.85E-10	6.44E-09
ERCC3	-0.31967	1.88E-10	6.52E-09
ZNF572	-0.31954	1.92E-10	6.63E-09
FOXO3	-0.31925	1.99E-10	6.87E-09
CARKD	-0.31915	2.02E-10	6.95E-09
KIAA1429	-0.31867	2.16E-10	7.39E-09
HDGFRP3	-0.31855	2.19E-10	7.5E-09
GEFT	-0.31843	2.23E-10	7.61E-09
TMEM43	-0.3184	2.24E-10	7.63E-09
SNTB1	-0.31832	2.26E-10	7.69E-09
HSN2	-0.31827	2.28E-10	7.72E-09
MYH7B	-0.31813	2.32E-10	7.84E-09
ERGIC3	-0.31757	2.51E-10	8.39E-09
PTK2	-0.31745	2.55E-10	8.48E-09
GPR155	-0.31735	2.59E-10	8.56E-09

ZNF187	-0.31714	2.66E-10	8.78E-09
CAB39L	-0.31713	2.66E-10	8.78E-09
EPB41L5	-0.31698	2.72E-10	8.95E-09
TTPAL	-0.31694	2.73E-10	8.98E-09
ZNF853	-0.31678	2.79E-10	9.13E-09
PCDHB6	-0.31666	2.84E-10	9.27E-09
IGF1R	-0.31649	2.9E-10	9.45E-09
CEP68	-0.31632	2.97E-10	9.66E-09
CBFA2T2	-0.31627	2.99E-10	9.71E-09
N6AMT2	-0.31578	3.2E-10	1.03E-08
ZSWIM1	-0.31563	3.26E-10	1.05E-08
ZSWIM3	-0.31554	3.3E-10	1.06E-08
SS18L1	-0.31546	3.34E-10	1.07E-08
ZNF713	-0.31542	3.36E-10	1.07E-08
C4orf38	-0.31494	3.58E-10	1.14E-08
RNF216	-0.31441	3.85E-10	1.21E-08
MBD5	-0.31437	3.86E-10	1.21E-08
AKAP11	-0.31426	3.93E-10	1.23E-08
DNAH17	-0.31411	4.01E-10	1.25E-08
ZNF212	-0.31392	4.11E-10	1.28E-08
R3HDML	-0.31391	4.11E-10	1.28E-08
KCNMB3	-0.31356	4.31E-10	1.34E-08
SFRS13B	-0.31349	4.35E-10	1.35E-08
WTIP	-0.3131	4.59E-10	1.42E-08
COMP	-0.31307	4.6E-10	1.42E-08
PTCH1	-0.31306	4.61E-10	1.42E-08
CLK1	-0.31296	4.67E-10	1.43E-08
CLDN11	-0.31269	4.84E-10	1.48E-08
NCOA3	-0.31254	4.94E-10	1.51E-08
C13orf23	-0.31249	4.97E-10	1.51E-08
GAS2	-0.31242	5.02E-10	1.52E-08
ZMYND8	-0.31217	5.19E-10	1.57E-08
TSHZ1	-0.31189	5.39E-10	1.63E-08
MAPRE1	-0.31139	5.76E-10	1.73E-08
UBR5	-0.31018	6.77E-10	2.01E-08
LPCAT2	-0.30996	6.97E-10	2.07E-08
CPNE1	-0.3098	7.12E-10	2.1E-08
ZNF302	-0.30964	7.26E-10	2.14E-08
ZNF498	-0.30963	7.28E-10	2.14E-08
KLHL24	-0.30924	7.66E-10	2.25E-08
ARHGEF7	-0.30883	8.08E-10	2.36E-08
CEP250	-0.30878	8.14E-10	2.37E-08
C20orf111	-0.30876	8.16E-10	2.38E-08
DIS3	-0.30855	8.39E-10	2.44E-08
CHST10	-0.30853	8.41E-10	2.44E-08
DLEU7	-0.3084	8.56E-10	2.47E-08
IRF2BP2	-0.30828	8.7E-10	2.5E-08
FGF3	-0.30803	8.98E-10	2.57E-08
DMRTC1B	-0.30774	9.33E-10	2.66E-08
CG030	-0.30772	9.36E-10	2.67E-08
ZNF490	-0.30753	9.6E-10	2.73E-08
RBM12B	-0.30694	1.04E-09	2.91E-08
SNHG11	-0.30683	1.05E-09	2.94E-08
AIP	-0.30675	1.06E-09	2.97E-08
FAM43B	-0.30674	1.06E-09	2.97E-08
BCORL1	-0.30661	1.08E-09	3.01E-08
ZNF84	-0.3064	1.11E-09	3.08E-08
FGF19	-0.30639	1.11E-09	3.08E-08

TRPC1	-0.30635	1.12E-09	3.1E-08
THBS4	-0.30622	1.14E-09	3.14E-08
MC1R	-0.30607	1.16E-09	3.2E-08
ANKRD27	-0.30597	1.18E-09	3.23E-08
TEAD3	-0.30564	1.23E-09	3.35E-08
RP9P	-0.3056	1.23E-09	3.37E-08
LOC10013	-0.30551	1.25E-09	3.4E-08
HOXA4	-0.3054	1.27E-09	3.45E-08
XPO4	-0.30536	1.27E-09	3.46E-08
SEMA4C	-0.30534	1.28E-09	3.46E-08
ABL2	-0.30517	1.31E-09	3.52E-08
ZNF585A	-0.30515	1.31E-09	3.53E-08
BSN	-0.30501	1.33E-09	3.58E-08
MECP2	-0.30493	1.35E-09	3.6E-08
TMEM91	-0.30493	1.35E-09	3.6E-08
LOC64676	-0.30491	1.35E-09	3.61E-08
DSG1	-0.30484	1.36E-09	3.63E-08
EFHC1	-0.30464	1.4E-09	3.7E-08
ATP9A	-0.30445	1.43E-09	3.79E-08
GDF5	-0.30424	1.47E-09	3.88E-08
GRINA	-0.30414	1.49E-09	3.92E-08
TSSK6	-0.30398	1.52E-09	4E-08
GNG4	-0.30397	1.53E-09	4E-08
C20orf112	-0.30373	1.57E-09	4.12E-08
TMEM65	-0.30339	1.64E-09	4.29E-08
SLC45A1	-0.30336	1.65E-09	4.3E-08
ZNF16	-0.30333	1.66E-09	4.31E-08
CPLX2	-0.30332	1.66E-09	4.31E-08
SLC38A11	-0.30325	1.67E-09	4.34E-08
PHYHIP	-0.30323	1.68E-09	4.34E-08
FARP1	-0.30282	1.77E-09	4.55E-08
CLDN9	-0.30274	1.79E-09	4.58E-08
ZNF235	-0.30272	1.79E-09	4.59E-08
RBAK	-0.30271	1.79E-09	4.59E-08
WDR35	-0.30244	1.86E-09	4.74E-08
CMYA5	-0.3023	1.89E-09	4.82E-08
DCHS2	-0.30221	1.91E-09	4.87E-08
ZNF3	-0.30217	1.92E-09	4.88E-08
YTHDF1	-0.30183	2.01E-09	5.08E-08
UPF3A	-0.30177	2.02E-09	5.11E-08
KIAA0892	-0.30172	2.04E-09	5.14E-08
PPP1R3D	-0.30154	2.08E-09	5.22E-08
SERINC1	-0.30154	2.09E-09	5.22E-08
PCDHB15	-0.30136	2.13E-09	5.32E-08
ANKK1	-0.30116	2.19E-09	5.43E-08
DDX27	-0.30098	2.24E-09	5.54E-08
FOXQ1	-0.30083	2.28E-09	5.63E-08
DYNLRB2	-0.30074	2.31E-09	5.68E-08
COX19	-0.30054	2.37E-09	5.82E-08
H19	-0.30053	2.37E-09	5.82E-08
TH1L	-0.30049	2.39E-09	5.85E-08
PCDHGC3	-0.30018	2.48E-09	6.06E-08
ADAMTS6	-0.30007	2.52E-09	6.15E-08
KIAA0406	-0.29993	2.56E-09	6.22E-08
FAM176A	-0.29982	2.6E-09	6.3E-08
ZSWIM4	-0.29976	2.62E-09	6.34E-08
KIAA1751	-0.29936	2.76E-09	6.65E-08
KIDINS220	-0.29924	2.8E-09	6.74E-08

NUFIP1	-0.2991	2.85E-09	6.85E-08
ZNF362	-0.29909	2.85E-09	6.85E-08
C6orf1	-0.29899	2.89E-09	6.93E-08
WHAMML	-0.29898	2.89E-09	6.93E-08
NCRNA00	-0.29882	2.95E-09	7.06E-08
UTP23	-0.29876	2.97E-09	7.1E-08
MLLT11	-0.29876	2.97E-09	7.1E-08
KCNH2	-0.29873	2.99E-09	7.11E-08
LNX2	-0.29872	2.99E-09	7.11E-08
POPDC2	-0.29862	3.03E-09	7.19E-08
KIAA0355	-0.29843	3.1E-09	7.35E-08
TRIM9	-0.29815	3.21E-09	7.59E-08
TBX19	-0.29792	3.31E-09	7.78E-08
ZNF292	-0.29785	3.34E-09	7.84E-08
TCF7L1	-0.29779	3.36E-09	7.89E-08
WFDC13	-0.29778	3.37E-09	7.89E-08
LRRC4C	-0.29773	3.39E-09	7.92E-08
CACNA2D	-0.29769	3.4E-09	7.94E-08
HOXA1	-0.29736	3.55E-09	8.26E-08
SMARCD1	-0.2973	3.57E-09	8.31E-08
RUSC2	-0.29727	3.59E-09	8.32E-08
GPR133	-0.29717	3.64E-09	8.4E-08
ZFHX4	-0.29674	3.84E-09	8.83E-08
ZCCHC24	-0.29654	3.93E-09	9.02E-08
PALM	-0.29654	3.94E-09	9.02E-08
MYL9	-0.2964	4.01E-09	9.17E-08
STARD3	-0.29633	4.04E-09	9.24E-08
ZNF775	-0.29628	4.07E-09	9.29E-08
AMIGO2	-0.29624	4.09E-09	9.3E-08
SCRG1	-0.29624	4.09E-09	9.3E-08
COPS8	-0.29618	4.12E-09	9.35E-08
RBP1	-0.29606	4.18E-09	9.47E-08
TM9SF4	-0.29604	4.19E-09	9.47E-08
MBTD1	-0.29595	4.24E-09	9.57E-08
LIMS3-LO	-0.29584	4.3E-09	9.7E-08
SEPT7P2	-0.29567	4.39E-09	9.86E-08
PCP2	-0.29563	4.41E-09	9.89E-08
CCDC92	-0.29562	4.42E-09	9.89E-08
HSPH1	-0.2956	4.43E-09	9.91E-08
MATN3	-0.29539	4.54E-09	1.01E-07
MEX3A	-0.29504	4.75E-09	1.05E-07
ZNF507	-0.295	4.77E-09	1.06E-07
DOCK6	-0.29496	4.8E-09	1.06E-07
RPL21	-0.29487	4.85E-09	1.07E-07
DLX3	-0.29484	4.87E-09	1.07E-07
KRIT1	-0.29466	4.98E-09	1.09E-07
LOC28507	-0.29447	5.1E-09	1.12E-07
SLC35C2	-0.29435	5.18E-09	1.13E-07
ATG9B	-0.29432	5.2E-09	1.14E-07
AMMECR1	-0.2943	5.21E-09	1.14E-07
DVL3	-0.29428	5.23E-09	1.14E-07
TIGD7	-0.29427	5.23E-09	1.14E-07
KLHL31	-0.29413	5.32E-09	1.16E-07
N4BP2L1	-0.29401	5.4E-09	1.17E-07
INHBB	-0.29398	5.42E-09	1.18E-07
NRF1	-0.29353	5.73E-09	1.24E-07
NBLA0030	-0.29346	5.78E-09	1.25E-07
HSPBAP1	-0.29344	5.8E-09	1.25E-07

LSM14B	-0.29329	5.91E-09	1.27E-07
DGKI	-0.29326	5.93E-09	1.28E-07
SLC25A36	-0.29303	6.1E-09	1.31E-07
WWC3	-0.29279	6.29E-09	1.35E-07
ARMC2	-0.29266	6.39E-09	1.37E-07
CDK19	-0.29249	6.53E-09	1.4E-07
DYNLRB1	-0.29204	6.9E-09	1.47E-07
RICTOR	-0.29196	6.97E-09	1.48E-07
MORC2	-0.29188	7.03E-09	1.49E-07
ZNF563	-0.29184	7.07E-09	1.49E-07
OBSL1	-0.29152	7.36E-09	1.54E-07
HOMER3	-0.29143	7.44E-09	1.56E-07
GTF3A	-0.29126	7.59E-09	1.59E-07
PHF21A	-0.29119	7.66E-09	1.6E-07
PABPC1L	-0.29116	7.69E-09	1.6E-07
RARA	-0.29112	7.73E-09	1.61E-07
UCKL1	-0.29099	7.85E-09	1.63E-07
C2orf60	-0.29098	7.86E-09	1.63E-07
DNMT3A	-0.29096	7.88E-09	1.63E-07
VPS37D	-0.29085	7.98E-09	1.65E-07
FXYD6	-0.29076	8.08E-09	1.67E-07
GRIK5	-0.29065	8.19E-09	1.69E-07
LDB1	-0.29057	8.27E-09	1.7E-07
MOSPD1	-0.29045	8.39E-09	1.72E-07
KATNAL1	-0.29041	8.43E-09	1.73E-07
TUBGCP3	-0.29038	8.46E-09	1.74E-07
SUPT3H	-0.2903	8.55E-09	1.75E-07
C6orf124	-0.29027	8.58E-09	1.76E-07
SHISA4	-0.29023	8.62E-09	1.76E-07
HSPB7	-0.2902	8.65E-09	1.77E-07
IL17D	-0.29002	8.84E-09	1.81E-07
APOC2	-0.28979	9.1E-09	1.86E-07
TBCEL	-0.28968	9.22E-09	1.88E-07
ZNF2	-0.2894	9.54E-09	1.94E-07
ELF2	-0.28927	9.69E-09	1.96E-07
SIN3B	-0.2892	9.77E-09	1.98E-07
PCDHB7	-0.28898	1E-08	2.03E-07
ST3GAL3	-0.28896	1.01E-08	2.03E-07
NEU1	-0.28881	1.03E-08	2.06E-07
YPEL1	-0.28863	1.05E-08	2.1E-07
UNKL	-0.28863	1.05E-08	2.1E-07
CAMKV	-0.28842	1.08E-08	2.15E-07
TTC17	-0.28838	1.08E-08	2.16E-07
LOC16847	-0.28815	1.11E-08	2.22E-07
FNDC3A	-0.28809	1.12E-08	2.23E-07
BMPR2	-0.28797	1.14E-08	2.26E-07
HAND2	-0.28783	1.16E-08	2.3E-07
KLF17	-0.28757	1.19E-08	2.36E-07
CHMP4B	-0.28749	1.2E-08	2.38E-07
FBXL3	-0.28748	1.21E-08	2.38E-07
CRY2	-0.28706	1.27E-08	2.5E-07
LOC39974	-0.28699	1.28E-08	2.51E-07
MARK4	-0.28686	1.3E-08	2.55E-07
NPTX1	-0.2866	1.34E-08	2.62E-07
MED12	-0.2866	1.34E-08	2.62E-07
CDK13	-0.2865	1.36E-08	2.65E-07
44811	-0.28638	1.38E-08	2.69E-07
NCRNA00	-0.2862	1.41E-08	2.74E-07

NCKAP5L	-0.28619	1.41E-08	2.74E-07
PCDHGA3	-0.28615	1.42E-08	2.75E-07
FLJ16779	-0.28612	1.42E-08	2.76E-07
TP53RK	-0.28608	1.43E-08	2.77E-07
LRCH1	-0.28608	1.43E-08	2.77E-07
SFRS6	-0.28595	1.45E-08	2.81E-07
COL22A1	-0.28591	1.46E-08	2.82E-07
PHF10	-0.28587	1.47E-08	2.83E-07
FAM53A	-0.2858	1.48E-08	2.85E-07
MAGED2	-0.28558	1.52E-08	2.91E-07
THSD7B	-0.28554	1.52E-08	2.92E-07
44809	-0.2854	1.55E-08	2.96E-07
DPH3B	-0.28532	1.57E-08	2.99E-07
TTYH1	-0.28506	1.62E-08	3.07E-07
KIF3C	-0.28492	1.64E-08	3.12E-07
ACTR5	-0.28486	1.65E-08	3.14E-07
GLIS2	-0.28486	1.66E-08	3.14E-07
KIF1A	-0.28476	1.67E-08	3.17E-07
ZNF23	-0.28458	1.71E-08	3.23E-07
CDC16	-0.28443	1.74E-08	3.28E-07
ZKSCAN4	-0.28438	1.75E-08	3.29E-07
RHOQ	-0.2843	1.77E-08	3.32E-07
OXR1	-0.28425	1.78E-08	3.33E-07
C22orf45	-0.28413	1.81E-08	3.38E-07
BRSK1	-0.28408	1.82E-08	3.39E-07
CEL	-0.28372	1.9E-08	3.53E-07
ZNF441	-0.28364	1.91E-08	3.56E-07
GPR179	-0.28362	1.92E-08	3.56E-07
EDNRA	-0.28361	1.92E-08	3.56E-07
HSPB6	-0.28347	1.96E-08	3.61E-07
PIBF1	-0.28326	2.01E-08	3.69E-07
CCDC136	-0.28319	2.02E-08	3.7E-07
SYS1	-0.28306	2.05E-08	3.76E-07
CRLF1	-0.283	2.07E-08	3.77E-07
CLSTN2	-0.28294	2.08E-08	3.79E-07
TNS1	-0.28251	2.19E-08	3.98E-07
ZNF567	-0.28238	2.23E-08	4.04E-07
LOC10012	-0.28225	2.26E-08	4.09E-07
USP35	-0.28197	2.34E-08	4.23E-07
ITGBL1	-0.28194	2.35E-08	4.24E-07
GMEB2	-0.28192	2.35E-08	4.24E-07
BCHE	-0.28191	2.35E-08	4.24E-07
GDI1	-0.28187	2.36E-08	4.26E-07
LONRF2	-0.28171	2.41E-08	4.33E-07
PERP	-0.28157	2.45E-08	4.4E-07
STK3	-0.28157	2.45E-08	4.4E-07
SESN1	-0.28127	2.54E-08	4.54E-07
PRELP	-0.28126	2.54E-08	4.54E-07
WIF1	-0.28124	2.55E-08	4.55E-07
TEAD1	-0.2812	2.56E-08	4.56E-07
INTS6	-0.28117	2.57E-08	4.57E-07
FGF14	-0.28092	2.65E-08	4.71E-07
KCNH8	-0.2809	2.66E-08	4.72E-07
ZNF532	-0.2808	2.68E-08	4.77E-07
ISM1	-0.2806	2.75E-08	4.87E-07
CLSTN3	-0.2806	2.75E-08	4.87E-07
C6orf174	-0.27982	3.01E-08	5.3E-07
GGT7	-0.2797	3.06E-08	5.38E-07

SLIT1	-0.27965	3.08E-08	5.4E-07
RUNDC3A	-0.27953	3.12E-08	5.47E-07
TCHH	-0.27942	3.16E-08	5.53E-07
CCDC50	-0.27941	3.16E-08	5.54E-07
C15orf52	-0.27918	3.25E-08	5.68E-07
PLAGL2	-0.27911	3.28E-08	5.73E-07
MAP1B	-0.27899	3.32E-08	5.8E-07
SUPT7L	-0.27854	3.5E-08	6.07E-07
VPS13B	-0.2785	3.52E-08	6.09E-07
C10orf140	-0.27824	3.63E-08	6.26E-07
ZFR	-0.27823	3.63E-08	6.26E-07
PDZD7	-0.27812	3.68E-08	6.34E-07
OMD	-0.27806	3.71E-08	6.37E-07
PCDHGA6	-0.27798	3.74E-08	6.42E-07
SLC6A3	-0.27771	3.86E-08	6.62E-07
PTPRN	-0.27762	3.9E-08	6.68E-07
HAO2	-0.2775	3.96E-08	6.75E-07
CASQ2	-0.27749	3.96E-08	6.75E-07
DPP6	-0.2774	4.01E-08	6.82E-07
SLC3A2	-0.27738	4.01E-08	6.83E-07
PROX1	-0.27736	4.02E-08	6.84E-07
C2orf54	-0.27706	4.16E-08	7.06E-07
PRSS53	-0.27702	4.19E-08	7.09E-07
GTF3C3	-0.27696	4.21E-08	7.12E-07
CUL4A	-0.27688	4.25E-08	7.18E-07
C16orf68	-0.27677	4.31E-08	7.27E-07
DOCK9	-0.27669	4.35E-08	7.33E-07
C6orf186	-0.27663	4.38E-08	7.37E-07
APLF	-0.27659	4.4E-08	7.39E-07
PKD2	-0.27659	4.4E-08	7.39E-07
LOC10013	-0.27657	4.41E-08	7.4E-07
COBLL1	-0.27651	4.44E-08	7.44E-07
ZNF26	-0.27651	4.44E-08	7.44E-07
SLC2A4RG	-0.27632	4.54E-08	7.6E-07
LAMA5	-0.27626	4.57E-08	7.63E-07
SEMA6C	-0.276	4.71E-08	7.85E-07
KCNIP3	-0.27599	4.72E-08	7.86E-07
STX1A	-0.27598	4.72E-08	7.86E-07
VOPP1	-0.2757	4.88E-08	8.1E-07
NKX3-2	-0.27564	4.91E-08	8.15E-07
PIKFYVE	-0.27559	4.94E-08	8.19E-07
RTKN	-0.27535	5.08E-08	8.41E-07
PFDN4	-0.27533	5.09E-08	8.42E-07
ANXA6	-0.27528	5.12E-08	8.46E-07
ZNF786	-0.27516	5.19E-08	8.57E-07
FLNA	-0.27512	5.22E-08	8.6E-07
NPR3	-0.27505	5.26E-08	8.66E-07
DSTYK	-0.27495	5.32E-08	8.75E-07
CORO2B	-0.27481	5.41E-08	8.87E-07
S1PR3	-0.27478	5.43E-08	8.9E-07
ULK1	-0.27477	5.43E-08	8.9E-07
ZKSCAN5	-0.27475	5.45E-08	8.91E-07
PTGIS	-0.27472	5.46E-08	8.93E-07
SYCP2	-0.2746	5.54E-08	9.04E-07
RBM20	-0.27459	5.55E-08	9.04E-07
GPR113	-0.27442	5.66E-08	9.21E-07
SHROOM4	-0.27441	5.67E-08	9.22E-07
C2orf27A	-0.27436	5.69E-08	9.25E-07

FKBP9	-0.27431	5.73E-08	9.3E-07
DACT3	-0.27425	5.77E-08	9.35E-07
LZTS2	-0.27397	5.96E-08	9.65E-07
PIP4K2B	-0.27374	6.12E-08	9.87E-07
MPP2	-0.27361	6.21E-08	1E-06
MYOZ3	-0.2736	6.22E-08	1E-06
GTF2F2	-0.27357	6.24E-08	1E-06
ZBTB2	-0.27348	6.31E-08	1.01E-06
DNMT3B	-0.2734	6.36E-08	1.02E-06
ZNF473	-0.27337	6.38E-08	1.02E-06
SRRM3	-0.27335	6.4E-08	1.02E-06
SYNM	-0.27326	6.47E-08	1.03E-06
KIAA1704	-0.27302	6.65E-08	1.06E-06
PENK	-0.27299	6.67E-08	1.06E-06
KIAA1310	-0.27286	6.77E-08	1.07E-06
COL27A1	-0.27258	6.99E-08	1.11E-06
TSPAN2	-0.2725	7.05E-08	1.12E-06
AKAP12	-0.27241	7.12E-08	1.13E-06
WWOX	-0.27238	7.15E-08	1.13E-06
ZNF192	-0.27233	7.19E-08	1.13E-06
COMMD7	-0.27228	7.23E-08	1.14E-06
DNM1	-0.27216	7.33E-08	1.15E-06
PSD	-0.27214	7.35E-08	1.16E-06
EFHD1	-0.27207	7.41E-08	1.16E-06
CACNA1D	-0.272	7.46E-08	1.17E-06
ZFP64	-0.272	7.47E-08	1.17E-06
TES	-0.27188	7.57E-08	1.19E-06
SLC5A6	-0.27181	7.64E-08	1.19E-06
KANK2	-0.2718	7.64E-08	1.19E-06
TIA1	-0.27178	7.66E-08	1.19E-06
PPP1R3B	-0.27165	7.77E-08	1.21E-06
NOS1	-0.2716	7.82E-08	1.21E-06
LOC38879	-0.2716	7.82E-08	1.21E-06
ZNF217	-0.27157	7.85E-08	1.22E-06
SCHIP1	-0.27152	7.89E-08	1.22E-06
KCNB1	-0.27146	7.94E-08	1.23E-06
ZSCAN2	-0.27145	7.95E-08	1.23E-06
TNFRSF19	-0.27143	7.97E-08	1.23E-06
C20orf199	-0.27133	8.06E-08	1.24E-06
EID2B	-0.27124	8.15E-08	1.25E-06
PRR3	-0.27123	8.15E-08	1.25E-06
ANKH	-0.2712	8.18E-08	1.26E-06
MPHOSPH	-0.27118	8.2E-08	1.26E-06
VPS54	-0.27116	8.22E-08	1.26E-06
ALS2CR8	-0.27114	8.23E-08	1.26E-06
ZFP1	-0.27079	8.57E-08	1.31E-06
MED30	-0.27073	8.63E-08	1.32E-06
PCMTD1	-0.27058	8.78E-08	1.34E-06
LRRC29	-0.27053	8.83E-08	1.34E-06
PHF20L1	-0.27043	8.92E-08	1.36E-06
LOC72874	-0.27042	8.94E-08	1.36E-06
C6orf195	-0.27038	8.98E-08	1.36E-06
FOXS1	-0.27037	8.99E-08	1.36E-06
PLOD3	-0.27031	9.06E-08	1.37E-06
HAND1	-0.27018	9.18E-08	1.39E-06
ZBTB49	-0.27012	9.25E-08	1.4E-06
TTLL9	-0.2699	9.49E-08	1.43E-06
PBX2	-0.26987	9.52E-08	1.43E-06

C2orf86	-0.26979	9.6E-08	1.44E-06
CCKBR	-0.2697	9.7E-08	1.45E-06
FAM91A1	-0.26966	9.74E-08	1.46E-06
JMJD1C	-0.26962	9.79E-08	1.46E-06
GPR56	-0.2696	9.81E-08	1.47E-06
ENOX1	-0.26956	9.85E-08	1.47E-06
THSD1	-0.2695	9.92E-08	1.48E-06
PHIP	-0.26935	1.01E-07	1.5E-06
DCAF12L2	-0.26933	1.01E-07	1.5E-06
EIF3H	-0.26911	1.04E-07	1.54E-06
SALL2	-0.26909	1.04E-07	1.54E-06
FKBP7	-0.2689	1.06E-07	1.57E-06
ESYT2	-0.2688	1.07E-07	1.59E-06
TMEM185I	-0.26867	1.09E-07	1.61E-06
BCAS3	-0.26863	1.09E-07	1.61E-06
CABLES2	-0.2686	1.1E-07	1.62E-06
FBXO2	-0.26858	1.1E-07	1.62E-06
MLLT10	-0.26854	1.11E-07	1.63E-06
C5orf23	-0.26829	1.14E-07	1.67E-06
MOCS3	-0.26803	1.17E-07	1.71E-06
UBE2V1	-0.26789	1.19E-07	1.74E-06
NDRG4	-0.26775	1.21E-07	1.76E-06
C10orf107	-0.26766	1.22E-07	1.78E-06
LOC64371	-0.26762	1.23E-07	1.78E-06
VAV2	-0.26759	1.23E-07	1.79E-06
SLC4A3	-0.26758	1.23E-07	1.79E-06
ABCC2	-0.26755	1.24E-07	1.79E-06
TNS3	-0.2675	1.24E-07	1.8E-06
DYNC1I1	-0.26738	1.26E-07	1.82E-06
C4orf12	-0.26737	1.26E-07	1.82E-06
LDB3	-0.26733	1.27E-07	1.83E-06
REEP2	-0.26732	1.27E-07	1.83E-06
C20orf20	-0.26731	1.27E-07	1.83E-06
PYGM	-0.2672	1.29E-07	1.85E-06
ZNF709	-0.26715	1.29E-07	1.86E-06
MAP1A	-0.2671	1.3E-07	1.87E-06
MN1	-0.26694	1.32E-07	1.9E-06
LMO3	-0.26688	1.33E-07	1.91E-06
CILP2	-0.26684	1.34E-07	1.92E-06
PIGU	-0.2668	1.34E-07	1.93E-06
SLC6A4	-0.26678	1.35E-07	1.93E-06
SLC9A8	-0.26671	1.36E-07	1.94E-06
BBS9	-0.2667	1.36E-07	1.94E-06
SLC6A2	-0.26654	1.38E-07	1.98E-06
ZNF512B	-0.26648	1.39E-07	1.99E-06
GRB7	-0.2664	1.41E-07	2E-06
SNRNP20(	-0.26631	1.42E-07	2.02E-06
C7orf53	-0.26623	1.43E-07	2.04E-06
HKR1	-0.26608	1.46E-07	2.07E-06
C5orf41	-0.26605	1.46E-07	2.07E-06
FAM168B	-0.26604	1.46E-07	2.08E-06
CPSF1	-0.26578	1.51E-07	2.13E-06
PLA2G5	-0.26562	1.53E-07	2.17E-06
TPM2	-0.2656	1.54E-07	2.17E-06
WFS1	-0.26549	1.55E-07	2.19E-06
POLR1D	-0.26544	1.56E-07	2.2E-06
ZNF365	-0.26542	1.57E-07	2.21E-06
MAPK8IP1	-0.26528	1.59E-07	2.24E-06

HSF1	-0.26527	1.59E-07	2.24E-06
OXTR	-0.26527	1.59E-07	2.24E-06
CYP27A1	-0.26524	1.6E-07	2.24E-06
KDM3A	-0.26521	1.6E-07	2.25E-06
MGP	-0.2652	1.61E-07	2.25E-06
ZNF319	-0.2651	1.62E-07	2.27E-06
CHRM2	-0.26509	1.63E-07	2.27E-06
ZFP14	-0.26501	1.64E-07	2.29E-06
LOC44242	-0.26495	1.65E-07	2.3E-06
TMEM136	-0.26482	1.68E-07	2.33E-06
BNIP2	-0.26478	1.68E-07	2.34E-06
PABPC3	-0.26473	1.69E-07	2.35E-06
PDRG1	-0.26468	1.7E-07	2.36E-06
LOC37519	-0.26454	1.73E-07	2.4E-06
CRYAB	-0.26447	1.74E-07	2.41E-06
ATP5E	-0.26438	1.76E-07	2.43E-06
FLJ37307	-0.26434	1.77E-07	2.44E-06
CDKAL1	-0.26434	1.77E-07	2.44E-06
KL	-0.2643	1.77E-07	2.44E-06
FHL3	-0.26427	1.78E-07	2.45E-06
C20orf135	-0.26421	1.79E-07	2.46E-06
CSRNP2	-0.26417	1.8E-07	2.47E-06
ZNF697	-0.2641	1.81E-07	2.49E-06
KDELC1	-0.26406	1.82E-07	2.5E-06
RPL30	-0.26397	1.84E-07	2.52E-06
RGS9BP	-0.2639	1.85E-07	2.53E-06
SLC12A4	-0.26379	1.88E-07	2.56E-06
C7orf13	-0.26366	1.91E-07	2.59E-06
FSTL3	-0.2636	1.92E-07	2.61E-06
WFDC10B	-0.26356	1.92E-07	2.62E-06
VEGFB	-0.26349	1.94E-07	2.64E-06
LAMP1	-0.2634	1.96E-07	2.66E-06
NGF	-0.26335	1.97E-07	2.67E-06
CYP26A1	-0.26319	2.01E-07	2.72E-06
RGL2	-0.26293	2.06E-07	2.79E-06
TMEM121	-0.26287	2.08E-07	2.81E-06
PRPF40B	-0.26285	2.08E-07	2.81E-06
WHAMML	-0.26274	2.11E-07	2.84E-06
AP3B2	-0.26274	2.11E-07	2.84E-06
FAM182B	-0.26261	2.14E-07	2.88E-06
ISM2	-0.26257	2.15E-07	2.89E-06
MAP2	-0.26257	2.15E-07	2.89E-06
TOMM34	-0.26255	2.15E-07	2.89E-06
CBX8	-0.26232	2.21E-07	2.95E-06
TMEM47	-0.2623	2.21E-07	2.96E-06
PDZD4	-0.26218	2.24E-07	2.99E-06
ING1	-0.26214	2.25E-07	3E-06
NFE2	-0.26201	2.28E-07	3.04E-06
LOC20003	-0.26189	2.31E-07	3.08E-06
C1orf113	-0.26186	2.32E-07	3.08E-06
ZBTB20	-0.26186	2.32E-07	3.08E-06
C7orf64	-0.26183	2.33E-07	3.09E-06
SPATA13	-0.26182	2.33E-07	3.09E-06
SGTB	-0.26155	2.4E-07	3.18E-06
PRKAB2	-0.26145	2.43E-07	3.21E-06
SLC22A3	-0.26138	2.45E-07	3.23E-06
LOC34911	-0.26137	2.45E-07	3.24E-06
HERPUD2	-0.26134	2.46E-07	3.24E-06

UBL3	-0.26132	2.46E-07	3.25E-06
CCNT2	-0.26131	2.47E-07	3.25E-06
CDIPT	-0.261	2.55E-07	3.35E-06
NACAD	-0.26094	2.57E-07	3.37E-06
DLX4	-0.26094	2.57E-07	3.37E-06
RAB11FIP3	-0.26091	2.58E-07	3.37E-06
CIR1	-0.26087	2.59E-07	3.38E-06
MED13	-0.2608	2.61E-07	3.41E-06
PACSIN3	-0.26075	2.62E-07	3.42E-06
IGSF9B	-0.26073	2.63E-07	3.43E-06
CROCCL2	-0.26069	2.64E-07	3.44E-06
PRDM11	-0.26068	2.64E-07	3.44E-06
MTR	-0.26067	2.64E-07	3.44E-06
FAM127C	-0.26065	2.65E-07	3.45E-06
HAP1	-0.26052	2.69E-07	3.49E-06
FTO	-0.26051	2.69E-07	3.49E-06
MYLIP	-0.26048	2.7E-07	3.5E-06
RNF38	-0.26043	2.71E-07	3.51E-06
TMEM169	-0.26042	2.72E-07	3.52E-06
C1orf70	-0.26034	2.74E-07	3.55E-06
ZNF193	-0.26029	2.75E-07	3.56E-06
CADM4	-0.26025	2.77E-07	3.58E-06
SOX11	-0.26024	2.77E-07	3.58E-06
POLR2J4	-0.26018	2.79E-07	3.6E-06
PCDHGA5	-0.26013	2.8E-07	3.61E-06
LOC64699	-0.26013	2.8E-07	3.61E-06
TBX15	-0.26002	2.84E-07	3.65E-06
ITGA7	-0.25999	2.85E-07	3.66E-06
HM13	-0.25994	2.86E-07	3.68E-06
DMTF1	-0.2599	2.88E-07	3.69E-06
ZRANB1	-0.25988	2.88E-07	3.69E-06
CYP4F3	-0.25977	2.92E-07	3.73E-06
TMEM150A	-0.25973	2.93E-07	3.74E-06
NEURL2	-0.25971	2.93E-07	3.75E-06
OGFR	-0.25962	2.96E-07	3.78E-06
TAF4	-0.25947	3.01E-07	3.84E-06
MACC1	-0.25944	3.02E-07	3.85E-06
ZNF92	-0.25937	3.05E-07	3.88E-06
FZD10	-0.25932	3.06E-07	3.89E-06
COL11A2	-0.25931	3.06E-07	3.89E-06
KRT23	-0.25926	3.08E-07	3.91E-06
CACNG1	-0.25915	3.12E-07	3.96E-06
HMCN1	-0.259	3.17E-07	4.01E-06
TPP2	-0.25897	3.18E-07	4.02E-06
SLC2A14	-0.25891	3.2E-07	4.05E-06
NEDD4	-0.2589	3.2E-07	4.05E-06
WDFY2	-0.25872	3.27E-07	4.12E-06
JPH2	-0.25865	3.29E-07	4.15E-06
GTF2IP1	-0.25857	3.32E-07	4.18E-06
MYLK4	-0.25847	3.36E-07	4.22E-06
ZNF434	-0.25806	3.51E-07	4.38E-06
PLN	-0.25782	3.6E-07	4.49E-06
EDEM2	-0.25782	3.6E-07	4.49E-06
ZNF117	-0.25778	3.62E-07	4.51E-06
SLC6A6	-0.25776	3.62E-07	4.51E-06
EXTL1	-0.25766	3.66E-07	4.55E-06
HAS2AS	-0.25764	3.67E-07	4.56E-06
ZNF227	-0.25758	3.7E-07	4.58E-06

CNTNAP1	-0.2574	3.77E-07	4.67E-06
ZC3H12B	-0.25733	3.8E-07	4.69E-06
C8orf33	-0.25731	3.8E-07	4.7E-06
LOC14870	-0.25728	3.82E-07	4.71E-06
PCDHB2	-0.25727	3.82E-07	4.71E-06
INSIG2	-0.25717	3.86E-07	4.76E-06
PCDHB12	-0.25711	3.89E-07	4.78E-06
ZNF606	-0.25706	3.91E-07	4.8E-06
AMDHD1	-0.25697	3.95E-07	4.84E-06
EIF3E	-0.25691	3.97E-07	4.87E-06
CDO1	-0.25688	3.98E-07	4.88E-06
LOH3CR2/	-0.2568	4.02E-07	4.92E-06
MTHFSD	-0.25676	4.04E-07	4.94E-06
NCRNA00(	-0.25641	4.19E-07	5.11E-06
NR6A1	-0.25637	4.21E-07	5.12E-06
SLC30A3	-0.25637	4.21E-07	5.12E-06
RYK	-0.25636	4.21E-07	5.12E-06
BIVM	-0.25617	4.3E-07	5.2E-06
CDH2	-0.25617	4.3E-07	5.2E-06
ADAMTSL:	-0.25615	4.31E-07	5.21E-06
ZNF608	-0.25611	4.33E-07	5.23E-06
HCG18	-0.256	4.38E-07	5.29E-06
SPINK6	-0.25595	4.4E-07	5.31E-06
LOC10012	-0.25592	4.42E-07	5.33E-06
KCNAB1	-0.25585	4.45E-07	5.36E-06
C6orf225	-0.25584	4.45E-07	5.37E-06
CATSPER1	-0.25578	4.48E-07	5.4E-06
ANO6	-0.2557	4.52E-07	5.43E-06
TOP1P1	-0.2556	4.57E-07	5.49E-06
BREA2	-0.25559	4.57E-07	5.49E-06
LRP11	-0.25542	4.66E-07	5.58E-06
CAPN10	-0.25541	4.66E-07	5.58E-06
DTX3	-0.25528	4.73E-07	5.65E-06
PCDHGA4	-0.25522	4.76E-07	5.68E-06
C20orf165	-0.25521	4.76E-07	5.69E-06
UGGT2	-0.25519	4.77E-07	5.69E-06
ADAM17	-0.25515	4.8E-07	5.71E-06
SNAI1	-0.255	4.87E-07	5.79E-06
ZNF843	-0.25499	4.87E-07	5.79E-06
LOC14578	-0.25491	4.92E-07	5.84E-06
GPR161	-0.2549	4.92E-07	5.84E-06
STON1	-0.25475	5E-07	5.92E-06
ABHD1	-0.2547	5.03E-07	5.94E-06
LOC39995	-0.25462	5.07E-07	5.99E-06
MYH3	-0.25459	5.09E-07	6E-06
ZNF182	-0.25457	5.1E-07	6.01E-06
NAV3	-0.25452	5.13E-07	6.04E-06
PCDH10	-0.2545	5.14E-07	6.05E-06
RSF1	-0.25449	5.14E-07	6.05E-06
LASS4	-0.25447	5.15E-07	6.06E-06
GJC1	-0.25446	5.16E-07	6.06E-06
WIPI2	-0.25445	5.16E-07	6.06E-06
UCHL1	-0.25434	5.22E-07	6.13E-06
FOXP4	-0.25427	5.26E-07	6.16E-06
PDLIM4	-0.25425	5.28E-07	6.17E-06
GPRASP2	-0.25419	5.31E-07	6.21E-06
KIF26B	-0.25404	5.39E-07	6.29E-06
EPDR1	-0.25375	5.56E-07	6.47E-06

NUPL1	-0.25357	5.67E-07	6.58E-06
GNAZ	-0.25354	5.69E-07	6.6E-06
NPHP3	-0.25351	5.7E-07	6.62E-06
SERPINA1(	-0.25345	5.74E-07	6.66E-06
PCP4	-0.25333	5.82E-07	6.73E-06
LOC10012	-0.25323	5.88E-07	6.79E-06
ASB5	-0.25312	5.94E-07	6.85E-06
DMWD	-0.25309	5.96E-07	6.88E-06
PCDHGB2	-0.25307	5.98E-07	6.89E-06
AGAP3	-0.25303	6E-07	6.91E-06
CENPJ	-0.25291	6.08E-07	6.98E-06
SFRP4	-0.2529	6.08E-07	6.98E-06
ARHGEF19	-0.25277	6.17E-07	7.08E-06
ZNF737	-0.25275	6.18E-07	7.08E-06
EPHA3	-0.25274	6.19E-07	7.09E-06
TCEAL4	-0.25269	6.22E-07	7.12E-06
ST6GAL2	-0.25259	6.29E-07	7.18E-06
AOX1	-0.25255	6.31E-07	7.2E-06
HEY2	-0.25253	6.33E-07	7.21E-06
CYTH3	-0.25241	6.41E-07	7.29E-06
GPR153	-0.25239	6.42E-07	7.29E-06
CYP2W1	-0.25237	6.44E-07	7.31E-06
PLA2G4C	-0.25225	6.52E-07	7.39E-06
AQP1	-0.25219	6.56E-07	7.43E-06
CRTAC1	-0.25218	6.56E-07	7.43E-06
CHD7	-0.25212	6.61E-07	7.47E-06
PLXNB3	-0.25207	6.64E-07	7.51E-06
DPM1	-0.25198	6.71E-07	7.57E-06
DYNC2H1	-0.25197	6.71E-07	7.57E-06
ZNF680	-0.25165	6.94E-07	7.78E-06
PCGF2	-0.25163	6.96E-07	7.8E-06
CAMK2B	-0.25147	7.07E-07	7.92E-06
TCEAL2	-0.2514	7.13E-07	7.97E-06
HIP1	-0.25138	7.14E-07	7.97E-06
LOC14582	-0.25138	7.14E-07	7.97E-06
RGS19	-0.2512	7.28E-07	8.11E-06
RCOR3	-0.25107	7.38E-07	8.22E-06
FCGRT	-0.251	7.43E-07	8.28E-06
TXLNB	-0.25098	7.45E-07	8.29E-06
ZBTB10	-0.25097	7.46E-07	8.29E-06
MSRB3	-0.25096	7.47E-07	8.29E-06
ABAT	-0.25091	7.5E-07	8.33E-06
IFRD1	-0.25086	7.54E-07	8.36E-06
ST3GAL2	-0.25081	7.58E-07	8.41E-06
NXF1	-0.25075	7.63E-07	8.46E-06
PCYT1B	-0.2507	7.67E-07	8.49E-06
ECEL1	-0.25069	7.67E-07	8.5E-06
CELP	-0.25063	7.72E-07	8.55E-06
LRP6	-0.25059	7.75E-07	8.57E-06
GPM6A	-0.25037	7.94E-07	8.77E-06
TMEM130	-0.25036	7.94E-07	8.77E-06
ARID3A	-0.25026	8.03E-07	8.85E-06
FXR1	-0.25022	8.06E-07	8.88E-06
NUMBL	-0.25022	8.06E-07	8.88E-06
EIF2S2	-0.25012	8.15E-07	8.96E-06
RANBP10	-0.2501	8.16E-07	8.97E-06
KCNE4	-0.25008	8.18E-07	8.97E-06
RABGAP1	-0.24991	8.33E-07	9.11E-06

TMEM59L	-0.24988	8.35E-07	9.14E-06
GAP43	-0.24978	8.44E-07	9.22E-06
ICA1L	-0.24972	8.5E-07	9.26E-06
ZC3H6	-0.2497	8.51E-07	9.27E-06
RASL11B	-0.24942	8.76E-07	9.52E-06
ABCA11P	-0.24938	8.79E-07	9.54E-06
ZDHHC22	-0.24929	8.88E-07	9.64E-06
ZNF154	-0.24928	8.89E-07	9.64E-06
GOLGA7B	-0.24926	8.91E-07	9.64E-06
SP7	-0.24925	8.92E-07	9.65E-06
ARFGEF2	-0.2492	8.96E-07	9.69E-06
EHBP1	-0.24919	8.98E-07	9.7E-06
GIGYF1	-0.24917	8.99E-07	9.71E-06
KLHL38	-0.24916	9E-07	9.71E-06
PAPOLG	-0.24912	9.04E-07	9.75E-06
SAP18	-0.24906	9.1E-07	9.8E-06
TRIM67	-0.24906	9.1E-07	9.8E-06
MFAP2	-0.24898	9.17E-07	9.87E-06
CLCN7	-0.24893	9.22E-07	9.92E-06
SUSD5	-0.24878	9.36E-07	1.01E-05
ZNF548	-0.24872	9.42E-07	1.01E-05
RAPGEF2	-0.24869	9.46E-07	1.02E-05
DPF2	-0.24858	9.56E-07	1.02E-05
CDH8	-0.24856	9.58E-07	1.03E-05
ZKSCAN1	-0.2485	9.64E-07	1.03E-05
TNRC18	-0.24843	9.71E-07	1.04E-05
LOC28317	-0.24838	9.76E-07	1.04E-05
LOC10027	-0.24834	9.8E-07	1.05E-05
FBXO17	-0.24833	9.82E-07	1.05E-05
HSPB8	-0.24829	9.86E-07	1.05E-05
SLC22A17	-0.24821	9.93E-07	1.06E-05
SP4	-0.24808	1.01E-06	1.07E-05
LIMS2	-0.24803	1.01E-06	1.08E-05
TAGLN	-0.24797	1.02E-06	1.08E-05
ZNF25	-0.24785	1.03E-06	1.09E-05
TLK1	-0.24775	1.04E-06	1.1E-05
LOC15869	-0.24773	1.04E-06	1.11E-05
CRHR1	-0.24771	1.05E-06	1.11E-05
E2F3	-0.24763	1.05E-06	1.11E-05
MYT1	-0.24758	1.06E-06	1.12E-05
TET1	-0.24755	1.06E-06	1.12E-05
LOC10019	-0.24751	1.07E-06	1.13E-05
SCT	-0.2475	1.07E-06	1.13E-05
PATZ1	-0.24729	1.09E-06	1.15E-05
ZNF260	-0.24725	1.1E-06	1.15E-05
SPAST	-0.24723	1.1E-06	1.16E-05
GTF2IRD1	-0.24719	1.1E-06	1.16E-05
C1QTNF4	-0.24714	1.11E-06	1.16E-05
PCDHB3	-0.24699	1.13E-06	1.18E-05
NUDT10	-0.24694	1.13E-06	1.19E-05
ZFAND1	-0.24687	1.14E-06	1.19E-05
VENTX	-0.24682	1.15E-06	1.2E-05
DMGDH	-0.24675	1.15E-06	1.21E-05
HSFX2	-0.24669	1.16E-06	1.21E-05
PCSK1N	-0.24666	1.17E-06	1.22E-05
JAM3	-0.24661	1.17E-06	1.22E-05
ZNF720	-0.24654	1.18E-06	1.23E-05
HOXD10	-0.24653	1.18E-06	1.23E-05

SIAH1	-0.24652	1.18E-06	1.23E-05
SUV420H1	-0.24626	1.21E-06	1.26E-05
CCDC82	-0.24624	1.22E-06	1.26E-05
FLJ13197	-0.24617	1.23E-06	1.27E-05
TIMP3	-0.24615	1.23E-06	1.27E-05
ZNF136	-0.24603	1.24E-06	1.29E-05
RDX	-0.24601	1.25E-06	1.29E-05
SR140	-0.24588	1.26E-06	1.31E-05
SNX16	-0.24581	1.27E-06	1.32E-05
TSPYL3	-0.2458	1.27E-06	1.32E-05
BRSK2	-0.24578	1.28E-06	1.32E-05
MEIS3	-0.24578	1.28E-06	1.32E-05
UPK1A	-0.24574	1.28E-06	1.32E-05
CUX1	-0.2457	1.29E-06	1.33E-05
GADD45B	-0.24569	1.29E-06	1.33E-05
TNNC2	-0.24566	1.29E-06	1.33E-05
MAP3K7	-0.24564	1.29E-06	1.33E-05
ODZ3	-0.24558	1.3E-06	1.34E-05
MOBKL1A	-0.24557	1.3E-06	1.34E-05
RASSF9	-0.24555	1.31E-06	1.34E-05
VCPIP1	-0.24545	1.32E-06	1.36E-05
TTC7B	-0.24543	1.32E-06	1.36E-05
KCNAB2	-0.24535	1.33E-06	1.37E-05
BAZ2B	-0.24533	1.34E-06	1.37E-05
OSBPL3	-0.24529	1.34E-06	1.37E-05
RG9MTD3	-0.24517	1.36E-06	1.39E-05
SYPL2	-0.24516	1.36E-06	1.39E-05
UFM1	-0.24515	1.36E-06	1.39E-05
C14orf4	-0.24495	1.39E-06	1.41E-05
INO80D	-0.24495	1.39E-06	1.41E-05
MEIS1	-0.24495	1.39E-06	1.41E-05
TBC1D5	-0.24493	1.39E-06	1.41E-05
HSF2	-0.24479	1.41E-06	1.43E-05
ERF	-0.24479	1.41E-06	1.43E-05
ERCC5	-0.24472	1.42E-06	1.44E-05
ZNF451	-0.24472	1.42E-06	1.44E-05
TNRC6A	-0.24464	1.43E-06	1.45E-05
ZNF418	-0.24454	1.45E-06	1.46E-05
LATS2	-0.2445	1.45E-06	1.47E-05
TCAP	-0.2444	1.47E-06	1.48E-05
CCDC115	-0.24439	1.47E-06	1.48E-05
GRPR	-0.24421	1.5E-06	1.51E-05
GATAD1	-0.24414	1.51E-06	1.52E-05
C7orf60	-0.24411	1.51E-06	1.52E-05
C6orf123	-0.24411	1.51E-06	1.52E-05
C9orf47	-0.2441	1.51E-06	1.52E-05
ZNF766	-0.24398	1.53E-06	1.54E-05
CREB1	-0.24379	1.56E-06	1.57E-05
CGNL1	-0.24368	1.58E-06	1.58E-05
ANKAR	-0.24361	1.59E-06	1.59E-05
MYH2	-0.2436	1.59E-06	1.6E-05
RUNX1	-0.24356	1.6E-06	1.6E-05
IDS	-0.24356	1.6E-06	1.6E-05
KCTD19	-0.24353	1.6E-06	1.6E-05
WDR5B	-0.24348	1.61E-06	1.61E-05
PCDHGC5	-0.24348	1.61E-06	1.61E-05
FGF13	-0.24345	1.62E-06	1.61E-05
CBFB	-0.24324	1.65E-06	1.64E-05

PAK7	-0.24322	1.65E-06	1.64E-05
CNN1	-0.24311	1.67E-06	1.66E-05
TATDN1	-0.24307	1.68E-06	1.67E-05
MED4	-0.24305	1.68E-06	1.67E-05
SPIN1	-0.24302	1.69E-06	1.67E-05
YPEL4	-0.24295	1.7E-06	1.68E-05
SLC6A17	-0.24289	1.71E-06	1.69E-05
PTPRZ1	-0.24287	1.71E-06	1.69E-05
IGSF10	-0.24279	1.73E-06	1.71E-05
ZNF566	-0.24273	1.74E-06	1.71E-05
JUB	-0.24272	1.74E-06	1.72E-05
SLC38A3	-0.24263	1.76E-06	1.73E-05
CLN5	-0.24258	1.77E-06	1.74E-05
PURB	-0.24257	1.77E-06	1.74E-05
C20orf195	-0.24256	1.77E-06	1.74E-05
SEMA3A	-0.24252	1.78E-06	1.74E-05
TSPYL4	-0.24252	1.78E-06	1.74E-05
PCDHB4	-0.24249	1.78E-06	1.75E-05
ZNF805	-0.24246	1.79E-06	1.75E-05
SAMD4B	-0.24244	1.79E-06	1.75E-05
GPSM1	-0.24241	1.8E-06	1.76E-05
ZNF174	-0.24231	1.81E-06	1.77E-05
UBN2	-0.2423	1.82E-06	1.78E-05
ZNF571	-0.24223	1.83E-06	1.79E-05
ACTA1	-0.24195	1.88E-06	1.83E-05
THBS2	-0.24193	1.89E-06	1.83E-05
NKAPL	-0.24192	1.89E-06	1.84E-05
GJC3	-0.24191	1.89E-06	1.84E-05
KLF7	-0.24183	1.91E-06	1.85E-05
GPR135	-0.24178	1.91E-06	1.86E-05
RCN3	-0.24178	1.92E-06	1.86E-05
JARID2	-0.24169	1.93E-06	1.87E-05
C8orf44	-0.24169	1.93E-06	1.87E-05
CTSF	-0.24159	1.95E-06	1.89E-05
RALY	-0.24158	1.95E-06	1.89E-05
ARHGAP3!	-0.24149	1.97E-06	1.9E-05
MYOM1	-0.24137	1.99E-06	1.92E-05
CRISPLD1	-0.24134	2E-06	1.93E-05
HCN2	-0.24131	2.01E-06	1.93E-05
GPC1	-0.24129	2.01E-06	1.93E-05
ZKSCAN2	-0.24129	2.01E-06	1.93E-05
AMOTL1	-0.24114	2.04E-06	1.96E-05
ABCC9	-0.24113	2.04E-06	1.96E-05
GPLD1	-0.24104	2.06E-06	1.98E-05
CAPS2	-0.24099	2.07E-06	1.99E-05
P4HA3	-0.24099	2.07E-06	1.99E-05
CUL4B	-0.24095	2.08E-06	1.99E-05
CYS1	-0.24082	2.11E-06	2.02E-05
FUBP3	-0.24076	2.12E-06	2.03E-05
AIRE	-0.24075	2.12E-06	2.03E-05
C3orf65	-0.24074	2.13E-06	2.03E-05
CSTF1	-0.24061	2.15E-06	2.05E-05
SLC7A6	-0.24058	2.16E-06	2.06E-05
PCP4L1	-0.24044	2.19E-06	2.08E-05
PHKG1	-0.24034	2.21E-06	2.11E-05
PCDHGB1	-0.24029	2.22E-06	2.11E-05
TTC25	-0.24026	2.23E-06	2.12E-05
PHF3	-0.24019	2.25E-06	2.13E-05

TTC35	-0.24003	2.28E-06	2.16E-05
MPP1	-0.23999	2.29E-06	2.17E-05
ZNF382	-0.23996	2.3E-06	2.17E-05
JRK	-0.23984	2.33E-06	2.2E-05
LZTS1	-0.23968	2.36E-06	2.23E-05
TOP1	-0.23956	2.39E-06	2.25E-05
PKD1	-0.23952	2.4E-06	2.26E-05
SRCIN1	-0.23946	2.42E-06	2.27E-05
PDLIM7	-0.23943	2.42E-06	2.28E-05
HSF2BP	-0.23938	2.43E-06	2.29E-05
OSBPL2	-0.2392	2.48E-06	2.32E-05
LOC65434	-0.2392	2.48E-06	2.32E-05
RBM38	-0.23914	2.49E-06	2.34E-05
PNMAL2	-0.23911	2.5E-06	2.34E-05
PLEKHA9	-0.23911	2.5E-06	2.34E-05
NOD1	-0.23893	2.55E-06	2.38E-05
COG6	-0.23892	2.55E-06	2.38E-05
ZNF577	-0.23891	2.55E-06	2.39E-05
SMARCA1	-0.23891	2.55E-06	2.39E-05
ASPN	-0.23864	2.62E-06	2.44E-05
TULP4	-0.23859	2.63E-06	2.45E-05
LY6G6D	-0.23853	2.65E-06	2.46E-05
ZNF221	-0.2385	2.66E-06	2.47E-05
C8orf77	-0.23848	2.66E-06	2.47E-05
KLHL23	-0.23846	2.67E-06	2.48E-05
IPO5	-0.23838	2.69E-06	2.49E-05
LANCL1	-0.23835	2.7E-06	2.5E-05
GP5	-0.23831	2.71E-06	2.51E-05
ARHGEF17	-0.23829	2.71E-06	2.51E-05
RBPMS2	-0.23822	2.73E-06	2.53E-05
FBRS	-0.23821	2.73E-06	2.53E-05
C1orf9	-0.2382	2.74E-06	2.53E-05
AZIN1	-0.23815	2.75E-06	2.54E-05
KLHL21	-0.23813	2.76E-06	2.54E-05
SYNGAP1	-0.23807	2.77E-06	2.55E-05
MANBAL	-0.23804	2.78E-06	2.56E-05
ZNF578	-0.23803	2.78E-06	2.56E-05
RIMKLB	-0.23799	2.79E-06	2.57E-05
ZNF708	-0.23793	2.81E-06	2.58E-05
UBE2E2	-0.23763	2.9E-06	2.65E-05
CALD1	-0.23749	2.94E-06	2.68E-05
ZNRF3	-0.23748	2.94E-06	2.68E-05
PHC3	-0.23746	2.94E-06	2.69E-05
GPRASP1	-0.23741	2.96E-06	2.7E-05
STARD13	-0.23735	2.98E-06	2.72E-05
PRKAA2	-0.23726	3E-06	2.74E-05
MMP16	-0.23725	3.01E-06	2.74E-05
ODZ2	-0.23722	3.02E-06	2.75E-05
BOC	-0.2372	3.02E-06	2.75E-05
WNT10A	-0.23719	3.02E-06	2.75E-05
LHFP	-0.23717	3.03E-06	2.76E-05
SERPINI1	-0.23711	3.05E-06	2.77E-05
ZNF610	-0.23704	3.07E-06	2.78E-05
C8orf31	-0.23704	3.07E-06	2.78E-05
NEK3	-0.237	3.08E-06	2.79E-05
MORN5	-0.23698	3.09E-06	2.8E-05
CBX6	-0.23692	3.11E-06	2.81E-05
AS3MT	-0.23691	3.11E-06	2.82E-05

CACNA1H	-0.23689	3.11E-06	2.82E-05
CRYAA	-0.23688	3.12E-06	2.82E-05
RTL1	-0.23681	3.14E-06	2.84E-05
GDAP1	-0.23677	3.15E-06	2.85E-05
ACTA2	-0.23675	3.16E-06	2.85E-05
ZNF547	-0.23674	3.16E-06	2.85E-05
YWHAB	-0.23662	3.2E-06	2.88E-05
RBM33	-0.23658	3.21E-06	2.89E-05
PLEKHA4	-0.23651	3.23E-06	2.91E-05
FGFR1	-0.23645	3.25E-06	2.93E-05
SLC7A10	-0.23641	3.27E-06	2.94E-05
PGF	-0.23641	3.27E-06	2.94E-05
SETD5	-0.23635	3.29E-06	2.95E-05
ZNF474	-0.23629	3.3E-06	2.96E-05
ZNF780A	-0.23629	3.31E-06	2.96E-05
OTUD6B	-0.23621	3.33E-06	2.98E-05
ZNF160	-0.23616	3.35E-06	2.99E-05
SLC4A1AP	-0.23615	3.35E-06	2.99E-05
CERK	-0.23607	3.38E-06	3.02E-05
GRIN3B	-0.23597	3.41E-06	3.04E-05
FOXC2	-0.23596	3.41E-06	3.04E-05
PTCHD2	-0.23589	3.44E-06	3.06E-05
ZNF510	-0.23578	3.48E-06	3.09E-05
MAPK14	-0.2357	3.5E-06	3.11E-05
HHIPL1	-0.23556	3.55E-06	3.15E-05
ADRA2C	-0.23551	3.57E-06	3.16E-05
XPNPEP2	-0.23542	3.6E-06	3.19E-05
GPATCH8	-0.23536	3.62E-06	3.2E-05
RND2	-0.23534	3.63E-06	3.21E-05
SF3B1	-0.23533	3.63E-06	3.21E-05
CEP63	-0.23531	3.64E-06	3.22E-05
ZNF19	-0.23531	3.64E-06	3.22E-05
DDAH2	-0.23529	3.64E-06	3.22E-05
RAD21	-0.23525	3.66E-06	3.23E-05
RARB	-0.23511	3.71E-06	3.27E-05
CPXM2	-0.23511	3.71E-06	3.27E-05
HNRNPH3	-0.2351	3.71E-06	3.27E-05
SYT7	-0.23507	3.72E-06	3.28E-05
STX2	-0.23499	3.76E-06	3.31E-05
EPB41L1	-0.23488	3.8E-06	3.34E-05
SLC26A10	-0.23481	3.82E-06	3.35E-05
SMPX	-0.23481	3.82E-06	3.35E-05
SMAD7	-0.23473	3.85E-06	3.38E-05
JRKL	-0.2347	3.86E-06	3.38E-05
LOC14318	-0.23469	3.87E-06	3.39E-05
PABPC1	-0.23461	3.9E-06	3.41E-05
ACCN4	-0.23452	3.93E-06	3.44E-05
ANKRD10	-0.23446	3.95E-06	3.46E-05
KLHL4	-0.23442	3.97E-06	3.47E-05
ACVR1B	-0.23439	3.98E-06	3.47E-05
GSC	-0.23435	4E-06	3.49E-05
ZNF17	-0.2343	4.01E-06	3.5E-05
PTCH2	-0.2343	4.01E-06	3.5E-05
CUL7	-0.23429	4.02E-06	3.5E-05
BICD2	-0.23427	4.03E-06	3.51E-05
SLC13A3	-0.23421	4.05E-06	3.53E-05
CEP170	-0.23412	4.09E-06	3.55E-05
TGFBI	-0.23411	4.09E-06	3.55E-05

FTL	-0.2341	4.09E-06	3.55E-05
UQCC	-0.23407	4.1E-06	3.56E-05
RBPJ	-0.23406	4.11E-06	3.56E-05
LGI3	-0.23399	4.14E-06	3.59E-05
C14orf93	-0.23396	4.15E-06	3.59E-05
SAMD14	-0.23394	4.16E-06	3.6E-05
MYBPHL	-0.23391	4.17E-06	3.61E-05
DPYSL3	-0.23387	4.19E-06	3.62E-05
TUG1	-0.23386	4.19E-06	3.62E-05
KDM4D	-0.23382	4.21E-06	3.63E-05
MXRA7	-0.23382	4.21E-06	3.63E-05
MTRF1	-0.23382	4.21E-06	3.63E-05
TMEM150(	-0.23378	4.22E-06	3.64E-05
COL25A1	-0.23371	4.25E-06	3.66E-05
UBAC2	-0.23371	4.25E-06	3.66E-05
VPS36	-0.23365	4.28E-06	3.68E-05
RPL7	-0.23365	4.28E-06	3.68E-05
ZNF224	-0.23359	4.3E-06	3.69E-05
DNAJB2	-0.23358	4.31E-06	3.69E-05
SYNPO2	-0.23355	4.32E-06	3.7E-05
AOC3	-0.23348	4.35E-06	3.73E-05
SBDSP1	-0.23347	4.35E-06	3.73E-05
FILIP1	-0.23333	4.41E-06	3.78E-05
PCDHB10	-0.23333	4.41E-06	3.78E-05
ITGA10	-0.23333	4.41E-06	3.78E-05
STAT5B	-0.23329	4.43E-06	3.79E-05
ZNF430	-0.23328	4.43E-06	3.79E-05
TOP1MT	-0.23325	4.45E-06	3.8E-05
RGAG4	-0.23325	4.45E-06	3.8E-05
ARL10	-0.2332	4.47E-06	3.81E-05
SRGAP2	-0.2332	4.47E-06	3.81E-05
TMEM170I	-0.23316	4.49E-06	3.82E-05
CA3	-0.23316	4.49E-06	3.82E-05
TGFB1I1	-0.23306	4.53E-06	3.86E-05
TRIM13	-0.23302	4.54E-06	3.87E-05
GTF2IRD2I	-0.23301	4.55E-06	3.87E-05
MAP4K3	-0.23301	4.55E-06	3.87E-05
PMS2L3	-0.23291	4.6E-06	3.9E-05
ORAI2	-0.23285	4.62E-06	3.92E-05
WIZ	-0.23278	4.65E-06	3.95E-05
LAYN	-0.23269	4.69E-06	3.98E-05
TACR2	-0.23266	4.71E-06	3.98E-05
FCHSD1	-0.23265	4.71E-06	3.99E-05
C8orf46	-0.23264	4.72E-06	3.99E-05
RNF182	-0.23254	4.76E-06	4.02E-05
HOXD9	-0.2325	4.78E-06	4.03E-05
44896	-0.23246	4.8E-06	4.05E-05
C6orf134	-0.23246	4.8E-06	4.05E-05
ZNF419	-0.23229	4.88E-06	4.11E-05
IGLON5	-0.23229	4.88E-06	4.11E-05
PAM	-0.23223	4.91E-06	4.13E-05
ZNF282	-0.2322	4.92E-06	4.14E-05
B4GALNT1	-0.23219	4.93E-06	4.14E-05
CHD2	-0.23216	4.94E-06	4.15E-05
KRT15	-0.23214	4.95E-06	4.16E-05
ZNF273	-0.23211	4.96E-06	4.17E-05
LMOD1	-0.23206	4.99E-06	4.18E-05
ARHGEF1	-0.23204	5E-06	4.19E-05

RPL32P3	-0.23194	5.05E-06	4.22E-05
CSDC2	-0.23188	5.07E-06	4.24E-05
C1orf105	-0.23188	5.08E-06	4.24E-05
NRP2	-0.23178	5.12E-06	4.28E-05
CLIP3	-0.23175	5.14E-06	4.29E-05
ARL4D	-0.23166	5.18E-06	4.33E-05
CAPS	-0.23166	5.19E-06	4.33E-05
PNMAL1	-0.23164	5.2E-06	4.33E-05
PPP1R9B	-0.23162	5.2E-06	4.34E-05
USP40	-0.2316	5.22E-06	4.35E-05
HLF	-0.23155	5.24E-06	4.36E-05
PRDM6	-0.23153	5.25E-06	4.37E-05
ZNF667	-0.23142	5.31E-06	4.41E-05
ENOX2	-0.2314	5.31E-06	4.41E-05
LOC72881	-0.23139	5.32E-06	4.41E-05
LAMB2	-0.23136	5.34E-06	4.43E-05
HBP1	-0.23128	5.38E-06	4.46E-05
NHLRC3	-0.23128	5.38E-06	4.46E-05
IFT88	-0.23125	5.39E-06	4.46E-05
ZNF673	-0.23125	5.4E-06	4.46E-05
CARTPT	-0.23114	5.45E-06	4.5E-05
C2orf81	-0.23112	5.46E-06	4.51E-05
CTSZ	-0.23105	5.5E-06	4.53E-05
GPR156	-0.231	5.52E-06	4.55E-05
TMEM200I	-0.23099	5.53E-06	4.56E-05
NPHP1	-0.23097	5.54E-06	4.56E-05
PEG3	-0.2309	5.58E-06	4.59E-05
VASH2	-0.23089	5.58E-06	4.59E-05
CAPRIN2	-0.23085	5.6E-06	4.6E-05
KIAA1549	-0.23084	5.61E-06	4.61E-05
SETDB1	-0.23075	5.66E-06	4.64E-05
SPOCK1	-0.23075	5.66E-06	4.64E-05
44808	-0.23073	5.67E-06	4.65E-05
DOK5	-0.23072	5.67E-06	4.65E-05
KCNK2	-0.23071	5.68E-06	4.65E-05
ZNF177	-0.23067	5.7E-06	4.67E-05
CHST13	-0.23064	5.72E-06	4.68E-05
GDAP1L1	-0.23058	5.75E-06	4.71E-05
LOC28554	-0.23053	5.78E-06	4.72E-05
WASF3	-0.23053	5.78E-06	4.72E-05
RP9	-0.23053	5.78E-06	4.72E-05
SEMA3E	-0.23051	5.79E-06	4.73E-05
TRIM23	-0.23048	5.81E-06	4.74E-05
PPP1R14A	-0.23046	5.82E-06	4.74E-05
HOXA3	-0.23044	5.83E-06	4.75E-05
ZNF107	-0.23043	5.83E-06	4.75E-05
ZNF646	-0.23038	5.86E-06	4.77E-05
CYP3A7	-0.23038	5.86E-06	4.77E-05
LOC15077	-0.23034	5.89E-06	4.79E-05
POLR2J2	-0.2303	5.91E-06	4.8E-05
NOD2	-0.23029	5.91E-06	4.8E-05
ZSCAN12	-0.23027	5.93E-06	4.81E-05
TRPM3	-0.23022	5.95E-06	4.82E-05
ART4	-0.23013	6.01E-06	4.87E-05
ZNF514	-0.23009	6.03E-06	4.88E-05
NPIP	-0.23002	6.07E-06	4.91E-05
ATF7	-0.22998	6.09E-06	4.93E-05
ZNF142	-0.22994	6.11E-06	4.94E-05

ENPEP	-0.22994	6.12E-06	4.94E-05
C12orf69	-0.22994	6.12E-06	4.94E-05
RAI14	-0.22981	6.19E-06	5E-05
ADCY2	-0.22963	6.3E-06	5.08E-05
CCBE1	-0.22956	6.34E-06	5.11E-05
MKLN1	-0.22953	6.36E-06	5.12E-05
AP1M1	-0.22952	6.36E-06	5.13E-05
ZNF862	-0.22945	6.41E-06	5.16E-05
TAF2	-0.22944	6.42E-06	5.16E-05
CYHR1	-0.22938	6.45E-06	5.19E-05
XRCC5	-0.22933	6.48E-06	5.21E-05
MYLK	-0.22932	6.49E-06	5.21E-05
PTH1R	-0.2292	6.56E-06	5.26E-05
FCRLB	-0.229	6.69E-06	5.36E-05
BAI1	-0.22894	6.73E-06	5.39E-05
WSCD2	-0.22893	6.73E-06	5.39E-05
LRRC37A3	-0.2289	6.75E-06	5.4E-05
TJAP1	-0.22887	6.77E-06	5.42E-05
FNTA	-0.22879	6.82E-06	5.45E-05
PALM3	-0.22879	6.82E-06	5.45E-05
NRSN1	-0.22876	6.84E-06	5.46E-05
ANXA9	-0.22876	6.84E-06	5.46E-05
CREBBP	-0.22874	6.86E-06	5.47E-05
RTP3	-0.22873	6.86E-06	5.47E-05
C9orf109	-0.22872	6.87E-06	5.48E-05
NRG3	-0.22871	6.87E-06	5.48E-05
CHD5	-0.22868	6.89E-06	5.49E-05
SMG7	-0.22866	6.91E-06	5.5E-05
ZNF541	-0.22864	6.92E-06	5.51E-05
ZNF660	-0.22862	6.93E-06	5.51E-05
TRIM39	-0.22857	6.96E-06	5.54E-05
SMTNL2	-0.22853	6.99E-06	5.56E-05
NAT14	-0.22851	7.01E-06	5.57E-05
GTF2IRD2	-0.22851	7.01E-06	5.57E-05
FAM66C	-0.22849	7.02E-06	5.57E-05
AKT3	-0.22848	7.03E-06	5.57E-05
USP51	-0.22847	7.03E-06	5.58E-05
SRRM5	-0.2283	7.14E-06	5.66E-05
COL2A1	-0.22824	7.19E-06	5.69E-05
NOTCH1	-0.22823	7.2E-06	5.69E-05
FJX1	-0.2282	7.21E-06	5.71E-05
SFRS18	-0.22811	7.28E-06	5.75E-05
KIAA2022	-0.22806	7.31E-06	5.76E-05
VPS11	-0.22804	7.32E-06	5.77E-05
PDZRN4	-0.22803	7.33E-06	5.78E-05
ACTC1	-0.22803	7.33E-06	5.78E-05
FNDC1	-0.22801	7.34E-06	5.78E-05
OLFML2B	-0.22799	7.36E-06	5.79E-05
ATXN2L	-0.22796	7.38E-06	5.81E-05
USP27X	-0.22795	7.39E-06	5.81E-05
ZNF431	-0.22783	7.47E-06	5.87E-05
RNF170	-0.22779	7.5E-06	5.89E-05
PEA15	-0.22774	7.54E-06	5.92E-05
LOC64367	-0.22767	7.58E-06	5.95E-05
MME	-0.22766	7.6E-06	5.95E-05
ZC3H3	-0.22759	7.64E-06	5.98E-05
ZNF821	-0.22756	7.67E-06	5.99E-05
NOTCH3	-0.22756	7.67E-06	5.99E-05

ZNF337	-0.22754	7.68E-06	6E-05
BVES	-0.22751	7.7E-06	6.01E-05
KIAA1586	-0.22751	7.7E-06	6.01E-05
ZNF767	-0.22746	7.73E-06	6.03E-05
ZNF782	-0.22742	7.77E-06	6.05E-05
LPAL2	-0.22728	7.87E-06	6.12E-05
PRR4	-0.22726	7.89E-06	6.14E-05
BIRC7	-0.22722	7.91E-06	6.15E-05
LOC25303	-0.22711	7.99E-06	6.21E-05
GABRD	-0.22708	8.02E-06	6.22E-05
ZNF781	-0.22708	8.02E-06	6.23E-05
R3HDM2	-0.22696	8.11E-06	6.29E-05
PTPN14	-0.22678	8.25E-06	6.39E-05
KCNK15	-0.22677	8.26E-06	6.39E-05
RASL10B	-0.22676	8.26E-06	6.39E-05
SMTN	-0.22675	8.28E-06	6.39E-05
KIAA0427	-0.22673	8.29E-06	6.4E-05
MCFD2	-0.22672	8.3E-06	6.41E-05
CDK5RAP1	-0.22669	8.32E-06	6.42E-05
TM6SF1	-0.22668	8.33E-06	6.42E-05
BRD4	-0.22666	8.34E-06	6.43E-05
ZNF568	-0.22654	8.44E-06	6.5E-05
C3orf32	-0.22653	8.45E-06	6.5E-05
GPR1	-0.22651	8.46E-06	6.51E-05
RNF150	-0.22642	8.53E-06	6.56E-05
JPH3	-0.22637	8.57E-06	6.58E-05
YEATS2	-0.22636	8.59E-06	6.59E-05
PGM5P2	-0.22634	8.6E-06	6.59E-05
SOAT2	-0.22633	8.61E-06	6.6E-05
RCBTB2	-0.22632	8.62E-06	6.61E-05
TMEM189	-0.22627	8.66E-06	6.63E-05
44806	-0.2262	8.71E-06	6.67E-05
SHANK3	-0.22616	8.75E-06	6.69E-05
ARFGEF1	-0.22608	8.82E-06	6.73E-05
C9orf25	-0.22594	8.93E-06	6.81E-05
RBM16	-0.2259	8.97E-06	6.83E-05
TMEM99	-0.22584	9.01E-06	6.86E-05
EEF2K	-0.22579	9.05E-06	6.89E-05
UCKL1AS	-0.22572	9.11E-06	6.92E-05
MYL3	-0.22558	9.23E-06	7.01E-05
YPEL3	-0.22556	9.26E-06	7.02E-05
DLL3	-0.22552	9.29E-06	7.04E-05
TMEM185A	-0.22541	9.38E-06	7.1E-05
SMURF2	-0.22536	9.42E-06	7.13E-05
KIAA1462	-0.22533	9.45E-06	7.14E-05
PIPOX	-0.22524	9.53E-06	7.2E-05
ZCCHC11	-0.22522	9.55E-06	7.21E-05
FGF9	-0.22515	9.61E-06	7.26E-05
CCDC45	-0.22515	9.62E-06	7.26E-05
LAMC1	-0.22514	9.63E-06	7.26E-05
REEP1	-0.22511	9.65E-06	7.28E-05
CXXC4	-0.22509	9.67E-06	7.29E-05
AGBL5	-0.22506	9.7E-06	7.31E-05
YTHDC1	-0.22504	9.72E-06	7.32E-05
SETD6	-0.22498	9.77E-06	7.35E-05
DLG2	-0.22492	9.83E-06	7.38E-05
PGR	-0.22492	9.83E-06	7.38E-05
MAP4K4	-0.22488	9.86E-06	7.4E-05

FAM38B	-0.22488	9.86E-06	7.4E-05
MPDZ	-0.22487	9.87E-06	7.41E-05
PARK2	-0.22484	9.9E-06	7.42E-05
C16orf70	-0.22483	9.91E-06	7.42E-05
ZNF503	-0.22481	9.93E-06	7.43E-05
ATM	-0.22476	9.97E-06	7.46E-05
TEX14	-0.22468	1E-05	7.5E-05
FBXO11	-0.22468	1E-05	7.5E-05
ZNF146	-0.22463	1.01E-05	7.54E-05
HEYL	-0.22461	1.01E-05	7.55E-05
PCDHGA2	-0.22458	1.01E-05	7.57E-05
MCF2L	-0.22456	1.02E-05	7.58E-05
ZNF627	-0.22453	1.02E-05	7.6E-05
BCOR	-0.22452	1.02E-05	7.6E-05
ATP5EP2	-0.22446	1.03E-05	7.64E-05
DCHS1	-0.22441	1.03E-05	7.67E-05
CHN2	-0.22437	1.03E-05	7.7E-05
C9orf110	-0.22434	1.04E-05	7.71E-05
PDPK1	-0.22433	1.04E-05	7.72E-05
SHC4	-0.22431	1.04E-05	7.73E-05
SLC7A6OS	-0.2243	1.04E-05	7.73E-05
CNIH3	-0.22425	1.05E-05	7.76E-05
CA11	-0.22424	1.05E-05	7.77E-05
CPT1C	-0.22419	1.05E-05	7.8E-05
KCTD8	-0.22418	1.05E-05	7.8E-05
STK36	-0.2241	1.06E-05	7.86E-05
NAALADL1	-0.2241	1.06E-05	7.86E-05
WNT6	-0.22409	1.06E-05	7.86E-05
LOC28559	-0.22408	1.06E-05	7.86E-05
COL8A1	-0.22403	1.07E-05	7.89E-05
ZEB1	-0.22397	1.07E-05	7.94E-05
COL10A1	-0.22383	1.09E-05	8.03E-05
PPP1R2	-0.22381	1.09E-05	8.04E-05
C9orf153	-0.22376	1.09E-05	8.07E-05
ADAMTS5	-0.22375	1.09E-05	8.08E-05
PGCP	-0.22373	1.1E-05	8.09E-05
GPR17	-0.22372	1.1E-05	8.1E-05
RNF180	-0.22371	1.1E-05	8.1E-05
DFNA5	-0.22364	1.11E-05	8.15E-05
ZFAT	-0.22359	1.11E-05	8.18E-05
PRICKLE2	-0.22356	1.11E-05	8.2E-05
ACR	-0.22352	1.12E-05	8.22E-05
C1orf95	-0.22352	1.12E-05	8.22E-05
SYT3	-0.2235	1.12E-05	8.23E-05
NAB1	-0.22348	1.12E-05	8.24E-05
PCDHGB3	-0.22346	1.12E-05	8.25E-05
CCDC8	-0.2234	1.13E-05	8.29E-05
KBTBD10	-0.22315	1.16E-05	8.48E-05
LOC10027	-0.22299	1.18E-05	8.58E-05
ZNF439	-0.22298	1.18E-05	8.59E-05
BRD7	-0.22295	1.18E-05	8.61E-05
ZDHHC9	-0.22294	1.18E-05	8.61E-05
DENND5A	-0.22291	1.18E-05	8.63E-05
ECM2	-0.2229	1.19E-05	8.63E-05
ZCWPW1	-0.22287	1.19E-05	8.65E-05
ATP1A2	-0.22284	1.19E-05	8.67E-05
MFGE8	-0.22282	1.19E-05	8.68E-05
ANO3	-0.22278	1.2E-05	8.71E-05

GAS1	-0.22277	1.2E-05	8.71E-05
NTSR1	-0.22274	1.2E-05	8.73E-05
KCNMB1	-0.22265	1.21E-05	8.8E-05
HTR2B	-0.22263	1.22E-05	8.81E-05
VAPB	-0.22246	1.23E-05	8.94E-05
TRAPPC9	-0.22244	1.24E-05	8.96E-05
SSC5D	-0.2224	1.24E-05	8.99E-05
FN1	-0.22238	1.24E-05	9E-05
NECAB1	-0.22237	1.24E-05	9E-05
DHRS12	-0.22237	1.25E-05	9E-05
NFYA	-0.22235	1.25E-05	9.01E-05
MTIF3	-0.22224	1.26E-05	9.1E-05
ZFPM2	-0.2222	1.26E-05	9.12E-05
CACNA2D	-0.22216	1.27E-05	9.16E-05
RAB9B	-0.2221	1.28E-05	9.2E-05
C20orf103	-0.22209	1.28E-05	9.2E-05
GRM7	-0.22201	1.29E-05	9.26E-05
FAM171A2	-0.22196	1.29E-05	9.3E-05
C20orf106	-0.22188	1.3E-05	9.36E-05
SPINK13	-0.22186	1.3E-05	9.37E-05
EBF2	-0.22186	1.3E-05	9.37E-05
RGL1	-0.22186	1.3E-05	9.37E-05
MGC23270	-0.22186	1.3E-05	9.37E-05
TUB	-0.22182	1.31E-05	9.39E-05
ADRA1D	-0.22175	1.32E-05	9.44E-05
LOC10027	-0.22175	1.32E-05	9.44E-05
GLTSCR1	-0.22173	1.32E-05	9.46E-05
FRMPD1	-0.22158	1.34E-05	9.58E-05
IQUB	-0.22157	1.34E-05	9.58E-05
PCDH19	-0.22156	1.34E-05	9.59E-05
MYOM3	-0.22152	1.35E-05	9.62E-05
CDKN1B	-0.2215	1.35E-05	9.63E-05
APBB1	-0.22146	1.35E-05	9.66E-05
PGBD1	-0.22144	1.36E-05	9.67E-05
GCLC	-0.22141	1.36E-05	9.69E-05
VGF	-0.22141	1.36E-05	9.69E-05
AHNAK2	-0.22137	1.37E-05	9.72E-05
FAM65A	-0.22136	1.37E-05	9.73E-05
PDGFB	-0.22128	1.38E-05	9.79E-05
ZNF225	-0.22114	1.39E-05	9.91E-05
UBTF	-0.22109	1.4E-05	9.95E-05
MRPS31	-0.22101	1.41E-05	0.0001
GALNS	-0.22098	1.41E-05	0.0001
PSD2	-0.22087	1.43E-05	0.000101
GUSBL2	-0.22085	1.43E-05	0.000101
KIAA0240	-0.2208	1.44E-05	0.000102
SMARCD3	-0.22074	1.45E-05	0.000102
BHLHB9	-0.22072	1.45E-05	0.000102
C20orf107	-0.22072	1.45E-05	0.000102
PALM2-AK	-0.22072	1.45E-05	0.000102
RAMP2	-0.22071	1.45E-05	0.000102
BOD1L	-0.22068	1.45E-05	0.000103
FLT4	-0.22068	1.45E-05	0.000103
LOC10013	-0.2206	1.46E-05	0.000103
APLP1	-0.22055	1.47E-05	0.000104
MORN3	-0.22052	1.47E-05	0.000104
CLDN15	-0.22049	1.48E-05	0.000104
SULF1	-0.22044	1.49E-05	0.000105

PCDHA12	-0.22039	1.49E-05	0.000105
GREB1L	-0.22039	1.49E-05	0.000105
C11orf63	-0.22038	1.49E-05	0.000105
CCDC122	-0.22028	1.51E-05	0.000106
ZNF134	-0.22008	1.54E-05	0.000108
PTBP2	-0.22004	1.54E-05	0.000108
DFNB59	-0.22003	1.54E-05	0.000108
PCDHB5	-0.2199	1.56E-05	0.000109
ZNF611	-0.2199	1.56E-05	0.000109
UBE2W	-0.21986	1.57E-05	0.00011
CAP2	-0.21985	1.57E-05	0.00011
IFT80	-0.21984	1.57E-05	0.00011
NAP1L3	-0.21981	1.57E-05	0.00011
IRS2	-0.21976	1.58E-05	0.00011
HSPA12A	-0.21971	1.59E-05	0.000111
BCL6	-0.21971	1.59E-05	0.000111
TSPAN18	-0.21968	1.59E-05	0.000111
ZNF263	-0.21961	1.6E-05	0.000112
RASA4	-0.21959	1.61E-05	0.000112
ANKRD40	-0.21946	1.63E-05	0.000113
EEF1A2	-0.21936	1.64E-05	0.000114
ACVR1	-0.2193	1.65E-05	0.000115
SPATA2	-0.2193	1.65E-05	0.000115
CELSR1	-0.21929	1.65E-05	0.000115
NFS1	-0.21923	1.66E-05	0.000115
KPNA5	-0.21922	1.66E-05	0.000115
FHOD3	-0.21916	1.67E-05	0.000116
NKAIN1	-0.21914	1.67E-05	0.000116
RUNX2	-0.21913	1.67E-05	0.000116
DMD	-0.2191	1.68E-05	0.000116
POLI	-0.21907	1.68E-05	0.000116
CREBZF	-0.21905	1.69E-05	0.000117
LAMB2L	-0.21903	1.69E-05	0.000117
CXCR7	-0.21899	1.7E-05	0.000117
RPN2	-0.21898	1.7E-05	0.000117
LRP12	-0.21891	1.71E-05	0.000118
PCDHB18	-0.21891	1.71E-05	0.000118
CHRNB4	-0.21874	1.73E-05	0.000119
LCAT	-0.21868	1.75E-05	0.00012
C9orf96	-0.21865	1.75E-05	0.00012
PROKR1	-0.21865	1.75E-05	0.00012
COMMD6	-0.21864	1.75E-05	0.00012
BBS5	-0.21864	1.75E-05	0.00012
CSPG4	-0.21862	1.75E-05	0.00012
CCDC81	-0.21848	1.78E-05	0.000122
CRTC1	-0.21847	1.78E-05	0.000122
LIME1	-0.21845	1.78E-05	0.000122
ARID4B	-0.21836	1.8E-05	0.000123
PHC1	-0.21833	1.8E-05	0.000123
LOC38858	-0.21833	1.8E-05	0.000123
PTP4A3	-0.21831	1.8E-05	0.000123
THRB	-0.2183	1.81E-05	0.000123
HSD17B14	-0.21829	1.81E-05	0.000123
APH1B	-0.21825	1.81E-05	0.000124
PCID2	-0.21817	1.83E-05	0.000124
GADL1	-0.21814	1.83E-05	0.000124
OR2A9P	-0.21814	1.83E-05	0.000125
ISLR2	-0.21807	1.84E-05	0.000125

DTNA	-0.21807	1.84E-05	0.000125
FNBP4	-0.21804	1.85E-05	0.000126
PMS2L2	-0.21795	1.86E-05	0.000126
PHYHIPL	-0.21793	1.87E-05	0.000126
ANKZF1	-0.21793	1.87E-05	0.000126
LOC22093	-0.21792	1.87E-05	0.000126
APCDD1L	-0.2179	1.87E-05	0.000127
TULP1	-0.21789	1.87E-05	0.000127
BGN	-0.21788	1.88E-05	0.000127
MYH11	-0.21782	1.89E-05	0.000127
ZNF746	-0.21768	1.91E-05	0.000129
ATCAY	-0.21767	1.91E-05	0.000129
PTPN1	-0.21764	1.92E-05	0.000129
GTF2IRD2B	-0.21762	1.92E-05	0.000129
TMEM67	-0.2176	1.92E-05	0.00013
C2orf42	-0.21758	1.93E-05	0.00013
CBL	-0.21754	1.93E-05	0.00013
CDHR3	-0.21751	1.94E-05	0.000131
ELFN1	-0.21747	1.95E-05	0.000131
GIGYF2	-0.21747	1.95E-05	0.000131
DISP1	-0.21745	1.95E-05	0.000131
FREM2	-0.21742	1.95E-05	0.000131
GLP1R	-0.21741	1.96E-05	0.000131
MMP17	-0.21733	1.97E-05	0.000132
KCNMB4	-0.21733	1.97E-05	0.000132
SGCD	-0.21729	1.98E-05	0.000133
FGF10	-0.21727	1.98E-05	0.000133
EZH1	-0.21727	1.98E-05	0.000133
MYOC	-0.21721	1.99E-05	0.000133
HDAC4	-0.21719	2E-05	0.000134
C7orf41	-0.21713	2.01E-05	0.000134
CNTN1	-0.21711	2.01E-05	0.000134
SLC39A4	-0.21708	2.02E-05	0.000135
ZNF284	-0.21707	2.02E-05	0.000135
PRL	-0.21706	2.02E-05	0.000135
C14orf101	-0.21706	2.02E-05	0.000135
DTNB	-0.21702	2.03E-05	0.000135
KRT39	-0.21702	2.03E-05	0.000135
CMTM3	-0.21693	2.04E-05	0.000136
SEC63	-0.21686	2.06E-05	0.000137
SNHG6	-0.21685	2.06E-05	0.000137
ASB1	-0.21682	2.06E-05	0.000137
ZNF100	-0.2168	2.07E-05	0.000138
FGF1	-0.21679	2.07E-05	0.000138
PACS1	-0.21678	2.07E-05	0.000138
ZNF74	-0.21677	2.07E-05	0.000138
EXOC4	-0.21673	2.08E-05	0.000138
ACOT8	-0.21664	2.1E-05	0.000139
CDK5R1	-0.21653	2.12E-05	0.00014
DPY19L2P2	-0.21651	2.12E-05	0.00014
DNAJB5	-0.21645	2.13E-05	0.000141
RAG1	-0.21631	2.16E-05	0.000143
BHMT2	-0.21631	2.16E-05	0.000143
C17orf103	-0.21626	2.17E-05	0.000143
LOC10019	-0.21622	2.18E-05	0.000144
FERMT2	-0.21621	2.18E-05	0.000144
FZD6	-0.21612	2.2E-05	0.000145
ZMIZ2	-0.21608	2.21E-05	0.000145

OXT	-0.21606	2.21E-05	0.000145
SLC8A1	-0.216	2.22E-05	0.000146
RRN3P1	-0.21597	2.23E-05	0.000147
NAP1L6	-0.21596	2.23E-05	0.000147
PGAM2	-0.21589	2.24E-05	0.000147
C20orf201	-0.21588	2.25E-05	0.000147
MAMSTR	-0.21585	2.25E-05	0.000148
TAF9B	-0.21581	2.26E-05	0.000148
FAM198A	-0.21581	2.26E-05	0.000148
NDRG3	-0.21569	2.29E-05	0.00015
RHEB	-0.21566	2.29E-05	0.00015
C20orf194	-0.21554	2.31E-05	0.000152
NCRNA00	-0.21552	2.32E-05	0.000152
AXL	-0.2155	2.32E-05	0.000152
COLQ	-0.21538	2.35E-05	0.000153
PLA2G15	-0.21531	2.36E-05	0.000154
ZBTB12	-0.21531	2.36E-05	0.000154
FAM69B	-0.21527	2.37E-05	0.000155
EFCAB2	-0.21523	2.38E-05	0.000155
DCAF13	-0.21519	2.39E-05	0.000156
GCM1	-0.21515	2.4E-05	0.000156
SCARNA7	-0.21514	2.4E-05	0.000156
ATP10A	-0.21509	2.41E-05	0.000157
VAT1	-0.21507	2.41E-05	0.000157
DUSP15	-0.21507	2.42E-05	0.000157
DOPEY1	-0.21499	2.43E-05	0.000158
SLC7A1	-0.21495	2.44E-05	0.000158
LOC44186	-0.21495	2.44E-05	0.000158
NAPB	-0.21494	2.44E-05	0.000158
TET3	-0.21482	2.47E-05	0.00016
DLX2	-0.21481	2.47E-05	0.00016
DNAJC5	-0.21456	2.53E-05	0.000163
FLJ33360	-0.21456	2.53E-05	0.000163
SLC35F1	-0.21451	2.54E-05	0.000164
SHISA9	-0.2145	2.54E-05	0.000164
PAPD5	-0.21448	2.55E-05	0.000164
SMYD5	-0.21447	2.55E-05	0.000165
LEF1	-0.21445	2.55E-05	0.000165
CACNG4	-0.21441	2.56E-05	0.000165
PPP2R2B	-0.2144	2.56E-05	0.000165
FLJ35390	-0.21435	2.57E-05	0.000166
ABCA17P	-0.21424	2.6E-05	0.000167
TIGD1	-0.2142	2.61E-05	0.000168
COG3	-0.21412	2.63E-05	0.000169
FMNL3	-0.2141	2.63E-05	0.000169
LMO7	-0.21409	2.63E-05	0.000169
NIPBL	-0.21404	2.65E-05	0.00017
ANKDD1A	-0.21393	2.67E-05	0.000172
ACCN2	-0.21389	2.68E-05	0.000172
NR2F2	-0.21388	2.68E-05	0.000172
CDADC1	-0.21381	2.7E-05	0.000173
UBTD1	-0.21374	2.72E-05	0.000174
RNASEH2E	-0.21372	2.72E-05	0.000174
MYCBP2	-0.21367	2.74E-05	0.000175
SSB	-0.21366	2.74E-05	0.000175
MXRA8	-0.21359	2.75E-05	0.000176
SYT11	-0.21359	2.75E-05	0.000176
APCDD1	-0.21358	2.76E-05	0.000176

DOK6	-0.21354	2.77E-05	0.000177
JAG2	-0.21348	2.78E-05	0.000178
CCIN	-0.21348	2.78E-05	0.000178
CORO6	-0.21343	2.79E-05	0.000178
SCRN1	-0.21339	2.8E-05	0.000179
SHISA2	-0.21336	2.81E-05	0.000179
PUS10	-0.21334	2.82E-05	0.000179
WDR19	-0.21332	2.82E-05	0.00018
KGFLP2	-0.21326	2.84E-05	0.00018
TGDS	-0.21326	2.84E-05	0.00018
FRS3	-0.21325	2.84E-05	0.00018
EFNA1	-0.21324	2.84E-05	0.000181
BGLAP	-0.21324	2.84E-05	0.000181
CUL3	-0.21322	2.85E-05	0.000181
TUBA1A	-0.21321	2.85E-05	0.000181
NSMAF	-0.21318	2.86E-05	0.000181
PVT1	-0.21317	2.86E-05	0.000181
CTXN2	-0.21315	2.86E-05	0.000181
C20orf152	-0.21312	2.87E-05	0.000182
GNA12	-0.21312	2.87E-05	0.000182
LEMD3	-0.21308	2.88E-05	0.000182
EPHA6	-0.21298	2.91E-05	0.000184
ITGB8	-0.21297	2.91E-05	0.000184
GRIN2A	-0.21294	2.92E-05	0.000185
MAPT	-0.21289	2.93E-05	0.000185
GUSBP1	-0.21282	2.95E-05	0.000186
POLR2J3	-0.21282	2.95E-05	0.000186
DEF8	-0.21282	2.95E-05	0.000186
COMMD5	-0.21273	2.97E-05	0.000187
TUBG2	-0.2127	2.98E-05	0.000188
ESPNP	-0.21266	2.99E-05	0.000188
MTBP	-0.21262	3E-05	0.000189
LOC10013	-0.21253	3.02E-05	0.00019
C7orf51	-0.21252	3.03E-05	0.00019
CEP170L	-0.21251	3.03E-05	0.000191
PRKD1	-0.21248	3.04E-05	0.000191
GATSL1	-0.21247	3.04E-05	0.000191
PDP1	-0.21246	3.04E-05	0.000191
DARS	-0.21244	3.05E-05	0.000191
MMP14	-0.21242	3.05E-05	0.000192
POU3F4	-0.21242	3.05E-05	0.000192
ZHX2	-0.21232	3.08E-05	0.000193
ZDHHC15	-0.21231	3.08E-05	0.000193
GUCY1A3	-0.21231	3.08E-05	0.000193
FRG1B	-0.21228	3.09E-05	0.000194
RASSF8	-0.21219	3.12E-05	0.000195
SFXN3	-0.21206	3.15E-05	0.000197
NUMA1	-0.21196	3.18E-05	0.000199
CDK8	-0.21195	3.18E-05	0.000199
ZNF791	-0.21191	3.19E-05	0.000199
RNF217	-0.21189	3.2E-05	0.000199
PCBP3	-0.21185	3.21E-05	0.0002
AASDHPP	-0.21178	3.23E-05	0.000201
CDH26	-0.21178	3.23E-05	0.000201
MOV10L1	-0.21162	3.28E-05	0.000204
GTPBP5	-0.2116	3.28E-05	0.000204
WDSUB1	-0.21158	3.29E-05	0.000204
RNF212	-0.21157	3.29E-05	0.000204

LOC28373	-0.21156	3.29E-05	0.000204
ZNF599	-0.21156	3.29E-05	0.000204
SHANK1	-0.2115	3.31E-05	0.000205
SNAP25	-0.21149	3.31E-05	0.000206
ITPKC	-0.21139	3.34E-05	0.000207
GOPC	-0.21139	3.35E-05	0.000207
SATB1	-0.21137	3.35E-05	0.000207
C1orf114	-0.21135	3.36E-05	0.000208
IGFBP7	-0.21135	3.36E-05	0.000208
LOC49375	-0.21134	3.36E-05	0.000208
ZFP28	-0.21124	3.39E-05	0.000209
NFYB	-0.21124	3.39E-05	0.000209
ZNF75A	-0.21121	3.4E-05	0.00021
HOXC13	-0.21119	3.4E-05	0.00021
LOC64453	-0.21113	3.42E-05	0.000211
ZNF483	-0.21113	3.42E-05	0.000211
PPP1R3F	-0.21105	3.44E-05	0.000212
LIG4	-0.21098	3.47E-05	0.000213
RNF32	-0.21097	3.47E-05	0.000214
C3orf47	-0.21093	3.48E-05	0.000214
AOAH	-0.21093	3.48E-05	0.000214
HRNR	-0.21092	3.48E-05	0.000214
ZNF594	-0.21091	3.49E-05	0.000214
SYCP2L	-0.21081	3.52E-05	0.000216
SLC25A27	-0.21076	3.53E-05	0.000217
ANKRD19	-0.21075	3.54E-05	0.000217
KIF3B	-0.21074	3.54E-05	0.000217
MAP9	-0.21064	3.57E-05	0.000219
LBH	-0.21063	3.57E-05	0.000219
SLC22A4	-0.21062	3.58E-05	0.000219
PTRF	-0.2106	3.58E-05	0.000219
PBX1	-0.2106	3.58E-05	0.000219
PCDHGC4	-0.21058	3.59E-05	0.000219
MED10	-0.21058	3.59E-05	0.000219
OSTM1	-0.21057	3.59E-05	0.000219
NGFR	-0.21055	3.6E-05	0.00022
MAP3K12	-0.21052	3.61E-05	0.00022
SCN3B	-0.21051	3.61E-05	0.00022
FADS3	-0.21034	3.67E-05	0.000223
KCNQ1OT	-0.21033	3.67E-05	0.000223
MLL5	-0.21032	3.67E-05	0.000223
ANKS1B	-0.21029	3.68E-05	0.000224
DHX36	-0.21028	3.69E-05	0.000224
NBPF9	-0.21024	3.7E-05	0.000225
LRCH2	-0.21015	3.73E-05	0.000226
IGFBP4	-0.21014	3.73E-05	0.000226
C13orf16	-0.21013	3.73E-05	0.000226
PDE7A	-0.21013	3.73E-05	0.000226
NOG	-0.21012	3.74E-05	0.000226
SMARCAL1	-0.21003	3.77E-05	0.000228
DLX1	-0.21001	3.77E-05	0.000228
NEXN	-0.2099	3.81E-05	0.00023
CCDC102A	-0.20977	3.85E-05	0.000232
ZBTB39	-0.20975	3.86E-05	0.000233
HNF4A	-0.20969	3.88E-05	0.000234
DCBLD2	-0.20968	3.88E-05	0.000234
LRRC23	-0.20964	3.89E-05	0.000235
CRHR2	-0.20963	3.9E-05	0.000235

SUN1	-0.20961	3.91E-05	0.000235
ITIH3	-0.20958	3.92E-05	0.000236
NXN	-0.20958	3.92E-05	0.000236
ARMCX1	-0.20957	3.92E-05	0.000236
ZNF253	-0.20955	3.93E-05	0.000236
NTM	-0.20948	3.95E-05	0.000237
LOC72826	-0.20947	3.95E-05	0.000237
CNPY4	-0.20941	3.97E-05	0.000238
SPERT	-0.20941	3.97E-05	0.000238
CDK6	-0.20937	3.99E-05	0.000239
PRG4	-0.20934	4E-05	0.00024
ITGB5	-0.20934	4E-05	0.00024
F13A1	-0.20922	4.04E-05	0.000242
EPHA8	-0.20919	4.05E-05	0.000242
CSGALNA(	-0.20917	4.06E-05	0.000243
CAND2	-0.20909	4.09E-05	0.000244
SGCA	-0.20905	4.1E-05	0.000245
ISLR	-0.20904	4.1E-05	0.000245
IBSP	-0.20901	4.12E-05	0.000246
NEK5	-0.20899	4.12E-05	0.000246
PNMT	-0.20895	4.14E-05	0.000247
SLC2A3	-0.20894	4.14E-05	0.000247
PCDHA2	-0.20893	4.14E-05	0.000247
C21orf29	-0.20889	4.16E-05	0.000248
HILS1	-0.20889	4.16E-05	0.000248
LOC28444	-0.20888	4.16E-05	0.000248
AKIRIN2	-0.20883	4.18E-05	0.000249
SLC45A2	-0.20879	4.2E-05	0.000249
EBAG9	-0.20874	4.21E-05	0.00025
CCDC93	-0.20873	4.22E-05	0.00025
RPS21	-0.20871	4.22E-05	0.000251
ZNF529	-0.20868	4.24E-05	0.000251
HIVEP2	-0.20866	4.24E-05	0.000251
PCDHB17	-0.20866	4.24E-05	0.000251
ZNF358	-0.20856	4.28E-05	0.000254
TBX1	-0.20852	4.29E-05	0.000254
BICD1	-0.20852	4.29E-05	0.000254
ZNF267	-0.20846	4.32E-05	0.000255
PDLIM3	-0.20844	4.32E-05	0.000256
ZNF449	-0.20841	4.33E-05	0.000256
PABPC5	-0.20841	4.33E-05	0.000256
ANGPTL1	-0.20839	4.34E-05	0.000256
ZNF500	-0.20834	4.36E-05	0.000257
MYLK3	-0.20833	4.36E-05	0.000257
NUAK1	-0.20833	4.37E-05	0.000257
GPC2	-0.20825	4.39E-05	0.000259
KIAA1267	-0.20819	4.42E-05	0.00026
SPARCL1	-0.20816	4.43E-05	0.000261
CST4	-0.20815	4.43E-05	0.000261
ARFRP1	-0.20815	4.43E-05	0.000261
TSPYL5	-0.20812	4.45E-05	0.000261
WNT16	-0.2081	4.45E-05	0.000262
CHRNA3	-0.20799	4.5E-05	0.000264
XIRP2	-0.20797	4.5E-05	0.000264
BMF	-0.20795	4.51E-05	0.000265
C12orf76	-0.20787	4.54E-05	0.000266
ATAD2B	-0.20778	4.58E-05	0.000268
TSHZ2	-0.20772	4.6E-05	0.000269

FILIP1L	-0.20769	4.61E-05	0.00027
ADAMTS1	-0.20766	4.62E-05	0.000271
QKI	-0.2076	4.65E-05	0.000272
FLJ39739	-0.20756	4.67E-05	0.000273
ALG10B	-0.2075	4.69E-05	0.000274
FBXO44	-0.20749	4.69E-05	0.000274
SMO	-0.20747	4.7E-05	0.000275
THUMPD2	-0.20743	4.72E-05	0.000275
PPT2	-0.20742	4.72E-05	0.000275
TCP10L2	-0.20737	4.74E-05	0.000277
C3orf17	-0.20736	4.75E-05	0.000277
STAC2	-0.2073	4.77E-05	0.000278
WRNIP1	-0.20724	4.79E-05	0.000279
LEPR	-0.2072	4.81E-05	0.00028
ADAMTS1	-0.20716	4.83E-05	0.000281
DMPK	-0.20712	4.85E-05	0.000282
TAF1D	-0.20706	4.87E-05	0.000283
PAPL	-0.20706	4.87E-05	0.000283
RAB3IL1	-0.20702	4.89E-05	0.000284
GATS	-0.20702	4.89E-05	0.000284
LMAN2L	-0.20699	4.9E-05	0.000284
ZSCAN18	-0.20682	4.97E-05	0.000288
C10orf68	-0.20678	4.99E-05	0.000289
UTP14C	-0.20673	5.01E-05	0.00029
EHMT2	-0.2067	5.02E-05	0.000291
CFL2	-0.20669	5.03E-05	0.000291
POGK	-0.20667	5.03E-05	0.000291
IQSEC1	-0.20657	5.08E-05	0.000294
C9orf5	-0.20656	5.08E-05	0.000294
ADAM22	-0.20655	5.09E-05	0.000294
RANBP9	-0.20654	5.09E-05	0.000294
ZNF471	-0.20654	5.09E-05	0.000294
C11orf74	-0.20654	5.09E-05	0.000294
FITM2	-0.20652	5.1E-05	0.000294
SYS1-DBN	-0.20651	5.1E-05	0.000294
ZNF137	-0.20648	5.12E-05	0.000295
ALDH1A3	-0.20648	5.12E-05	0.000295
GFPT2	-0.20646	5.12E-05	0.000295
NOX4	-0.20644	5.14E-05	0.000296
TMEM178	-0.20642	5.14E-05	0.000296
LOC37449	-0.20638	5.16E-05	0.000297
NCBP2	-0.20637	5.17E-05	0.000297
VGLL3	-0.20631	5.19E-05	0.000299
NRXN2	-0.2063	5.2E-05	0.000299
FRMD6	-0.20628	5.21E-05	0.000299
LOC44035	-0.20628	5.21E-05	0.000299
PDGFC	-0.20624	5.22E-05	0.0003
LRRTM4	-0.20623	5.23E-05	0.0003
PCDHB19F	-0.20623	5.23E-05	0.0003
MAP4K2	-0.20622	5.23E-05	0.0003
GBX2	-0.20611	5.28E-05	0.000303
TTLL1	-0.20605	5.31E-05	0.000304
KCNC1	-0.20593	5.36E-05	0.000307
NF1	-0.2059	5.38E-05	0.000308
SORCS3	-0.20587	5.39E-05	0.000309
FAM84B	-0.20585	5.4E-05	0.000309
COL4A2	-0.20584	5.41E-05	0.000309
SUGT1	-0.20582	5.41E-05	0.00031

MPHOSPH	-0.20575	5.45E-05	0.000311
HNRNPUL	-0.2057	5.47E-05	0.000313
FAM75A2	-0.20569	5.48E-05	0.000313
ANKRD1	-0.20564	5.5E-05	0.000314
LOC34007	-0.20562	5.51E-05	0.000315
FAM195B	-0.20561	5.51E-05	0.000315
RASL12	-0.20554	5.55E-05	0.000316
DBNDD2	-0.20548	5.58E-05	0.000318
FAM172A	-0.20544	5.59E-05	0.000319
ZNF322B	-0.20536	5.63E-05	0.000321
HOXA2	-0.2053	5.66E-05	0.000322
KIAA1009	-0.20529	5.67E-05	0.000322
RBM12	-0.20516	5.73E-05	0.000326
ATP2B2	-0.20514	5.74E-05	0.000326
C7orf10	-0.20514	5.74E-05	0.000326
DUSP22	-0.20509	5.76E-05	0.000327
PBXIP1	-0.20505	5.78E-05	0.000328
SLC16A10	-0.20499	5.81E-05	0.000329
CTSL2	-0.20489	5.86E-05	0.000332
GRAMD1A	-0.20489	5.86E-05	0.000332
NR1D2	-0.20483	5.89E-05	0.000334
ENTHD1	-0.2048	5.91E-05	0.000334
COL16A1	-0.20473	5.94E-05	0.000336
RASA3	-0.2047	5.96E-05	0.000337
MYST3	-0.20453	6.05E-05	0.000341
TF	-0.20439	6.12E-05	0.000345
RGN	-0.20438	6.12E-05	0.000345
CALY	-0.20438	6.12E-05	0.000345
HCG11	-0.20437	6.12E-05	0.000345
PRSS45	-0.20436	6.13E-05	0.000345
PRICKLE1	-0.20431	6.16E-05	0.000346
HOXD11	-0.20429	6.17E-05	0.000347
PROC	-0.20423	6.2E-05	0.000349
SCN7A	-0.20419	6.22E-05	0.00035
ZNF638	-0.20419	6.22E-05	0.00035
SLC22A16	-0.20415	6.24E-05	0.000351
LRTM2	-0.20411	6.26E-05	0.000352
CSGALNA(	-0.20409	6.27E-05	0.000352
KIAA1614	-0.20404	6.3E-05	0.000354
BEAN	-0.20404	6.3E-05	0.000354
POTEG	-0.20401	6.32E-05	0.000354
NNAT	-0.20397	6.33E-05	0.000355
GUCY1B3	-0.20397	6.33E-05	0.000355
RBBP6	-0.20394	6.35E-05	0.000356
NIPSNAP3	-0.20392	6.36E-05	0.000356
TTBK2	-0.20392	6.36E-05	0.000356
ZNF813	-0.20392	6.37E-05	0.000356
NOTCH4	-0.20389	6.38E-05	0.000357
FAP	-0.20388	6.39E-05	0.000357
TUT1	-0.20386	6.4E-05	0.000357
NRTN	-0.20385	6.4E-05	0.000358
ZNF629	-0.20376	6.45E-05	0.00036
C5orf54	-0.20367	6.5E-05	0.000363
ZNF223	-0.20363	6.52E-05	0.000364
OR2A1	-0.20358	6.55E-05	0.000365
RNF114	-0.20357	6.55E-05	0.000365
LOC14457	-0.20357	6.56E-05	0.000365
UNK	-0.20355	6.56E-05	0.000366

CWF19L2	-0.20342	6.64E-05	0.00037
ATXN7L2	-0.20339	6.66E-05	0.00037
MYST1	-0.20332	6.7E-05	0.000372
DDR2	-0.20327	6.72E-05	0.000374
COL8A2	-0.20325	6.73E-05	0.000374
TH	-0.20322	6.75E-05	0.000375
C21orf82	-0.20317	6.78E-05	0.000376
HS6ST1	-0.2031	6.82E-05	0.000379
GLI2	-0.20308	6.83E-05	0.000379
PPP1R3C	-0.20306	6.84E-05	0.000379
MUM1	-0.20305	6.85E-05	0.000379
BTN2A1	-0.20298	6.89E-05	0.000381
ZNF254	-0.20298	6.89E-05	0.000381
FGF5	-0.20298	6.89E-05	0.000381
ALOX15	-0.2029	6.94E-05	0.000384
PDZK1P1	-0.20289	6.94E-05	0.000384
METTL6	-0.20283	6.98E-05	0.000386
MSL2	-0.20278	7.01E-05	0.000387
ARID1B	-0.20275	7.02E-05	0.000388
DVWA	-0.20271	7.05E-05	0.000389
PRSS50	-0.20269	7.06E-05	0.00039
PHACTR1	-0.20269	7.06E-05	0.00039
FLJ37201	-0.20267	7.07E-05	0.00039
KIAA0196	-0.20265	7.08E-05	0.00039
FBXO30	-0.20265	7.09E-05	0.000391
ZBED5	-0.20261	7.11E-05	0.000391
C20orf3	-0.20259	7.12E-05	0.000392
TBKBP1	-0.20257	7.13E-05	0.000393
PLA2R1	-0.20252	7.16E-05	0.000394
TDRKH	-0.2025	7.17E-05	0.000395
H2BFXP	-0.20245	7.2E-05	0.000396
PRKRIP1	-0.20242	7.22E-05	0.000397
ZNF423	-0.20241	7.23E-05	0.000397
KIAA0182	-0.2024	7.24E-05	0.000397
EMID2	-0.20239	7.24E-05	0.000398
ZNF383	-0.20238	7.24E-05	0.000398
MEST	-0.20225	7.32E-05	0.000402
DES	-0.20225	7.33E-05	0.000402
GIPC3	-0.20222	7.34E-05	0.000402
TMEM145	-0.20216	7.38E-05	0.000404
SNORA39	-0.2021	7.42E-05	0.000406
TMEM90A	-0.20206	7.44E-05	0.000407
SERPIND1	-0.20199	7.49E-05	0.00041
CRYGN	-0.20197	7.5E-05	0.00041
CDYL2	-0.20188	7.55E-05	0.000412
GLRB	-0.20178	7.62E-05	0.000416
LAPTM4B	-0.20171	7.67E-05	0.000418
LAMP2	-0.20166	7.7E-05	0.000419
FUS	-0.20166	7.7E-05	0.000419
STMN3	-0.20163	7.72E-05	0.00042
SFT2D3	-0.20163	7.72E-05	0.00042
POGZ	-0.20156	7.76E-05	0.000422
SH2D5	-0.20153	7.78E-05	0.000423
STAG2	-0.20138	7.88E-05	0.000428
KIAA1107	-0.20133	7.91E-05	0.000429
TCF7	-0.20125	7.96E-05	0.000432
IGFBP3	-0.20125	7.96E-05	0.000432
C7orf70	-0.20111	8.06E-05	0.000436

HTR7P1	-0.20105	8.1E-05	0.000439
C2orf40	-0.20103	8.11E-05	0.000439
HS3ST4	-0.20101	8.13E-05	0.000439
MAGEA5	-0.20099	8.14E-05	0.00044
KRT40	-0.20099	8.14E-05	0.00044
CLU	-0.20097	8.15E-05	0.000441
YPEL5	-0.20095	8.16E-05	0.000441
PICALM	-0.20095	8.17E-05	0.000441
NINL	-0.20094	8.17E-05	0.000441
PLEKHF2	-0.20092	8.19E-05	0.000442
RFPL2	-0.20092	8.19E-05	0.000442
ZNF677	-0.20092	8.19E-05	0.000442
SV2A	-0.20092	8.19E-05	0.000442
AMZ2P1	-0.20078	8.28E-05	0.000446
MYH10	-0.20073	8.32E-05	0.000448
VANGL2	-0.20071	8.33E-05	0.000449
PIGT	-0.20069	8.34E-05	0.000449
NAB2	-0.20068	8.35E-05	0.00045
DDX19B	-0.20066	8.37E-05	0.00045
RAB7L1	-0.20064	8.38E-05	0.000451
ZNF81	-0.20062	8.4E-05	0.000452
44627	-0.2006	8.41E-05	0.000452
KPNA3	-0.2006	8.41E-05	0.000452
MPP6	-0.2004	8.55E-05	0.00046
CPEB1	-0.2003	8.62E-05	0.000463
FRMPD4	-0.2003	8.62E-05	0.000463
TUFT1	-0.20026	8.65E-05	0.000464
BCL9	-0.20025	8.66E-05	0.000464
ACTG2	-0.20023	8.67E-05	0.000465
ALKBH8	-0.20022	8.68E-05	0.000465
DKFZp434	-0.20021	8.69E-05	0.000465
USP34	-0.20021	8.69E-05	0.000465
JAKMIP2	-0.2001	8.77E-05	0.000469
OPRL1	-0.20009	8.78E-05	0.00047
BEST1	-0.20003	8.81E-05	0.000471
GOLGA8C	-0.19995	8.88E-05	0.000474
RS1	-0.19988	8.93E-05	0.000477
FAM20C	-0.19984	8.96E-05	0.000478
IGDCC3	-0.19982	8.97E-05	0.000479
SYDE2	-0.19982	8.97E-05	0.000479
WFDC9	-0.19976	9.02E-05	0.000481
MAP7D2	-0.19972	9.04E-05	0.000482
TGFBR3	-0.19971	9.06E-05	0.000483
PRAF2	-0.1997	9.06E-05	0.000483
HTR2A	-0.1997	9.06E-05	0.000483
ORAI3	-0.19967	9.08E-05	0.000483
SFRP2	-0.19965	9.1E-05	0.000484
KLHDC8A	-0.19963	9.12E-05	0.000485
MAPK4	-0.19962	9.13E-05	0.000485
LOC34234	-0.19961	9.13E-05	0.000485
CTGF	-0.19961	9.13E-05	0.000485
TTC28	-0.1996	9.14E-05	0.000486
WFDC10A	-0.19959	9.14E-05	0.000486
IGFBP6	-0.19956	9.17E-05	0.000487
ALDH8A1	-0.19953	9.19E-05	0.000488
ATXN1	-0.19951	9.21E-05	0.000489
SCARF2	-0.19939	9.3E-05	0.000493
MED13L	-0.19938	9.3E-05	0.000493

LOC10010	-0.19935	9.33E-05	0.000495
RFTN2	-0.19934	9.34E-05	0.000495
ETAA1	-0.19932	9.35E-05	0.000495
DYRK1B	-0.19927	9.39E-05	0.000497
ZNF676	-0.19926	9.4E-05	0.000498
PCDHGA1	-0.19919	9.46E-05	0.0005
DBN1	-0.19916	9.48E-05	0.000501
WDR93	-0.19914	9.49E-05	0.000502
SOX15	-0.19908	9.54E-05	0.000504
EFEMP2	-0.19907	9.55E-05	0.000504
ARID4A	-0.19895	9.65E-05	0.000509
VCAN	-0.19894	9.65E-05	0.000509
FAM120C	-0.19892	9.67E-05	0.00051
SLC17A7	-0.19879	9.77E-05	0.000515
PPP2R2C	-0.19876	9.8E-05	0.000516
ACPT	-0.19869	9.85E-05	0.000519
ZNF252	-0.19867	9.87E-05	0.00052
ANKRD6	-0.19866	9.88E-05	0.00052
PAX1	-0.19855	9.97E-05	0.000524
ACTN1	-0.1985	0.0001	0.000526
RBM43	-0.19848	0.0001	0.000526
SLC1A5	-0.19848	0.0001	0.000526
SLC2A1	-0.19842	0.000101	0.000528
DZIP1	-0.19839	0.000101	0.00053
ADAM11	-0.19837	0.000101	0.00053
ZNF10	-0.19833	0.000101	0.000532
AGBL2	-0.19824	0.000102	0.000535
IGF2	-0.19824	0.000102	0.000535
C10orf62	-0.19824	0.000102	0.000535
HYDIN	-0.19823	0.000102	0.000535
SHANK2	-0.19818	0.000103	0.000537
NTNG2	-0.19813	0.000103	0.00054
LOC28520	-0.19806	0.000104	0.000542
RERG	-0.19805	0.000104	0.000542
ADRM1	-0.19804	0.000104	0.000543
DNAJC6	-0.19793	0.000105	0.000548
ZNF350	-0.19783	0.000106	0.000552
CCNL1	-0.1978	0.000106	0.000553
LDLRAD2	-0.1978	0.000106	0.000553
TBC1D16	-0.1978	0.000106	0.000553
DLG4	-0.19776	0.000106	0.000554
DLX5	-0.19771	0.000107	0.000556
SERPINE2	-0.19767	0.000107	0.000558
GPR26	-0.1976	0.000108	0.000561
ZNF345	-0.19758	0.000108	0.000561
RERE	-0.19753	0.000108	0.000563
SUPT5H	-0.19749	0.000109	0.000565
C20orf123	-0.19746	0.000109	0.000566
ZFR2	-0.19744	0.000109	0.000567
ATP1B2	-0.19743	0.000109	0.000567
FIBIN	-0.1974	0.00011	0.000568
PLK1S1	-0.19734	0.00011	0.000571
44807	-0.19731	0.00011	0.000572
MESTIT1	-0.19729	0.000111	0.000573
POM121C	-0.19728	0.000111	0.000573
MEOX2	-0.19727	0.000111	0.000573
DLGAP2	-0.19727	0.000111	0.000573
PTF1A	-0.19724	0.000111	0.000574

FKBP10	-0.19721	0.000111	0.000576
RUNX1T1	-0.19718	0.000112	0.000576
CASC3	-0.19715	0.000112	0.000578
ZNF317	-0.19715	0.000112	0.000578
NFATC3	-0.19713	0.000112	0.000579
DUS4L	-0.19712	0.000112	0.000579
NCRNA00(	-0.19709	0.000112	0.00058
UBA2	-0.19705	0.000113	0.000582
NTN5	-0.19704	0.000113	0.000582
KIAA0430	-0.19704	0.000113	0.000582
CNTNAP5	-0.19701	0.000113	0.000583
EFNA4	-0.19696	0.000114	0.000585
CYP1B1	-0.19693	0.000114	0.000586
DDIT4L	-0.1969	0.000114	0.000588
SLC22A1	-0.19689	0.000114	0.000588
PFN2	-0.19678	0.000115	0.000593
GTDC1	-0.19673	0.000116	0.000594
TNN	-0.19673	0.000116	0.000594
TAS1R3	-0.19672	0.000116	0.000595
TMOD2	-0.19667	0.000116	0.000597
PABPC4L	-0.19665	0.000116	0.000597
CBLL1	-0.19665	0.000117	0.000597
NUDT11	-0.19664	0.000117	0.000598
VAMP4	-0.19663	0.000117	0.000598
OGN	-0.19651	0.000118	0.000604
BRAF	-0.19647	0.000118	0.000605
TDRD3	-0.19642	0.000119	0.000607
PCF11	-0.19642	0.000119	0.000607
OLFML2A	-0.19641	0.000119	0.000608
TNRC6C	-0.19632	0.00012	0.000611
PHF1	-0.19631	0.00012	0.000611
TDRD6	-0.19629	0.00012	0.000612
ZNF829	-0.19627	0.00012	0.000613
SCUBE3	-0.1962	0.000121	0.000616
C16orf5	-0.19618	0.000121	0.000617
GEM	-0.19617	0.000121	0.000618
LOC10013	-0.19613	0.000122	0.000619
RPRD2	-0.19599	0.000123	0.000625
KLHL15	-0.19598	0.000123	0.000626
PLEKHM3	-0.19598	0.000123	0.000626
PARD6B	-0.19591	0.000124	0.000629
ZNF763	-0.19583	0.000125	0.000632
PCDHGB6	-0.19583	0.000125	0.000632
PSPC1	-0.19583	0.000125	0.000632
FBXL7	-0.19579	0.000125	0.000634
CXorf57	-0.19571	0.000126	0.000637
PGAP3	-0.19566	0.000126	0.00064
FBXO32	-0.1956	0.000127	0.000642
FAM19A5	-0.19556	0.000127	0.000644
LYNX1	-0.19553	0.000128	0.000645
PVR	-0.19553	0.000128	0.000645
C1orf187	-0.19551	0.000128	0.000646
APOD	-0.19551	0.000128	0.000646
C1orf152	-0.19548	0.000128	0.000647
PGM5	-0.19547	0.000128	0.000648
ZMYM3	-0.19544	0.000129	0.000649
TMEM179	-0.19542	0.000129	0.00065
C14orf149	-0.19539	0.000129	0.000651

ATRX	-0.19529	0.00013	0.000655
CWC22	-0.19529	0.00013	0.000656
ANKMY1	-0.1952	0.000131	0.00066
RRM2B	-0.19517	0.000131	0.000662
INA	-0.19512	0.000132	0.000663
TTYH2	-0.19509	0.000132	0.000665
CTDSP2	-0.19506	0.000133	0.000666
ENPP1	-0.19504	0.000133	0.000667
POLR2K	-0.19501	0.000133	0.000668
MYEF2	-0.19495	0.000134	0.000671
GOLGA6A	-0.19482	0.000135	0.000678
ZFP30	-0.19481	0.000135	0.000678
ZNF234	-0.19478	0.000136	0.00068
KIAA0564	-0.19478	0.000136	0.00068
KLHDC1	-0.19477	0.000136	0.00068
CHMP4C	-0.19473	0.000136	0.000681
NFATC2	-0.19468	0.000137	0.000684
MRE11A	-0.19463	0.000137	0.000686
CHRDL1	-0.19462	0.000137	0.000686
ARHGEF4	-0.19451	0.000139	0.000692
LOXL4	-0.1945	0.000139	0.000692
SETD1A	-0.19449	0.000139	0.000693
DHX34	-0.19444	0.000139	0.000695
NHSL2	-0.19443	0.00014	0.000696
EFCAB1	-0.19443	0.00014	0.000696
FOXC1	-0.19435	0.00014	0.0007
C21orf7	-0.1943	0.000141	0.000702
BAI3	-0.19429	0.000141	0.000703
C6orf164	-0.19426	0.000141	0.000705
ZNF155	-0.19424	0.000142	0.000706
KIAA1274	-0.19423	0.000142	0.000706
KIF3A	-0.19421	0.000142	0.000707
KCNH6	-0.19419	0.000142	0.000707
KRTAP3-3	-0.19417	0.000142	0.000708
C5orf4	-0.19414	0.000143	0.00071
FAM110B	-0.19414	0.000143	0.00071
TMEM216	-0.1941	0.000143	0.000711
DDX50	-0.19407	0.000144	0.000713
DPYSL4	-0.19406	0.000144	0.000713
PALM2	-0.19406	0.000144	0.000713
C19orf18	-0.19405	0.000144	0.000714
RSPO4	-0.19387	0.000146	0.000723
SV2B	-0.19387	0.000146	0.000723
LPIN3	-0.1938	0.000147	0.000726
CCDC80	-0.19371	0.000148	0.000731
LMX1B	-0.19365	0.000149	0.000734
DGCR9	-0.19363	0.000149	0.000736
LASS1	-0.19361	0.000149	0.000736
ASPSCR1	-0.19359	0.000149	0.000737
VN1R1	-0.19354	0.00015	0.00074
LRP2BP	-0.19354	0.00015	0.00074
MCC	-0.19353	0.00015	0.00074
TSN	-0.19353	0.00015	0.00074
PCDHA3	-0.19352	0.00015	0.00074
CYP19A1	-0.19352	0.00015	0.00074
LOC15116	-0.19351	0.00015	0.000741
VEGFC	-0.19349	0.00015	0.000741
IL17RD	-0.19348	0.000151	0.000742

TMEM25	-0.19343	0.000151	0.000745
HMGB1	-0.19339	0.000152	0.000747
KLHDC8B	-0.19335	0.000152	0.000749
SLC5A7	-0.19333	0.000152	0.000749
ZC3H8	-0.19333	0.000152	0.000749
TLE3	-0.19327	0.000153	0.000753
RPRM	-0.19323	0.000154	0.000755
PLEKHB1	-0.19316	0.000154	0.000759
FAM176B	-0.19314	0.000155	0.00076
GRID2IP	-0.19314	0.000155	0.00076
NME7	-0.19309	0.000155	0.000763
C2orf68	-0.19306	0.000156	0.000764
SMYD1	-0.19306	0.000156	0.000764
ANTXR1	-0.19306	0.000156	0.000764
KLHL30	-0.19298	0.000157	0.000768
MLXIPL	-0.19298	0.000157	0.000768
MUTED	-0.19298	0.000157	0.000768
CHST1	-0.19294	0.000157	0.00077
F8	-0.1929	0.000158	0.000772
C2orf76	-0.19287	0.000158	0.000774
HCG2P7	-0.19286	0.000158	0.000774
GPSM2	-0.19285	0.000158	0.000774
LRRC17	-0.19283	0.000159	0.000775
SYDE1	-0.19281	0.000159	0.000776
ZFP112	-0.19281	0.000159	0.000776
TBC1D4	-0.19279	0.000159	0.000776
GRP	-0.19269	0.00016	0.000782
LRRC49	-0.19267	0.000161	0.000783
ZSCAN20	-0.19266	0.000161	0.000784
VPS39	-0.19266	0.000161	0.000784
CRAMP1L	-0.19252	0.000163	0.000791
TGFB3	-0.19249	0.000163	0.000793
SMARCE1	-0.19245	0.000163	0.000795
ATIC	-0.19236	0.000165	0.000801
INTS4	-0.19234	0.000165	0.000802
PCDHB16	-0.19226	0.000166	0.000806
TTC14	-0.19221	0.000167	0.00081
UBXN7	-0.19215	0.000168	0.000814
CDKL3	-0.19201	0.000169	0.000822
BMI1	-0.192	0.00017	0.000822
VEZF1	-0.19198	0.00017	0.000823
ARAP1	-0.19195	0.00017	0.000825
SIX1	-0.19195	0.00017	0.000825
FBXL2	-0.19193	0.00017	0.000826
MEIS2	-0.19188	0.000171	0.000829
ZNF496	-0.19186	0.000171	0.00083
C8orf39	-0.19186	0.000171	0.00083
SOHLH1	-0.19177	0.000173	0.000835
LRRC37B	-0.19169	0.000174	0.000841
ACTN2	-0.19168	0.000174	0.000841
FRMD4A	-0.19166	0.000174	0.000842
FLJ44054	-0.19165	0.000174	0.000842
SDC2	-0.19162	0.000175	0.000844
FLG	-0.19152	0.000176	0.00085
HIC2	-0.19148	0.000177	0.000853
SFRP5	-0.19146	0.000177	0.000854
HSD17B6	-0.19144	0.000177	0.000855
FMO2	-0.19144	0.000177	0.000855

ZMAT4	-0.19139	0.000178	0.000858
HELZ	-0.19138	0.000178	0.000858
EIF4A2	-0.19131	0.000179	0.000863
LOC10027	-0.19131	0.000179	0.000863
JHDM1D	-0.19126	0.00018	0.000866
ZNF550	-0.1912	0.000181	0.00087
DCAF17	-0.19119	0.000181	0.00087
CAD	-0.19117	0.000181	0.000871
ANK2	-0.19108	0.000182	0.000877
RAPGEF4	-0.19108	0.000182	0.000877
HAS2	-0.19106	0.000183	0.000877
PHF2	-0.19104	0.000183	0.000878
DACT1	-0.19101	0.000183	0.00088
ZNF442	-0.19101	0.000183	0.00088
YTHDF3	-0.19098	0.000184	0.000882
INTS4L1	-0.19093	0.000185	0.000885
DNAH1	-0.19091	0.000185	0.000886
OBP2B	-0.19087	0.000186	0.000888
C14orf166	-0.19084	0.000186	0.00089
ZBTB24	-0.19082	0.000186	0.000891
PPT1	-0.19081	0.000186	0.000891
PRKG1	-0.19073	0.000188	0.000896
SLC35B4	-0.1907	0.000188	0.000899
RGMA	-0.19067	0.000188	0.0009
FLJ10038	-0.19065	0.000189	0.000901
NKAIN4	-0.19063	0.000189	0.000902
PCDH9	-0.19062	0.000189	0.000903
MLL	-0.19058	0.00019	0.000905
PAIP2	-0.19057	0.00019	0.000906
CRABP2	-0.19057	0.00019	0.000906
ZNF727	-0.19056	0.00019	0.000906
PHF8	-0.19055	0.00019	0.000907
PTDSS1	-0.19051	0.000191	0.000909
HOPX	-0.1905	0.000191	0.00091
44814	-0.19048	0.000191	0.000911
NUDCD1	-0.19048	0.000191	0.000911
LOC34459	-0.19045	0.000192	0.000912
RNF175	-0.19043	0.000192	0.000914
GET4	-0.19032	0.000194	0.00092
LRRC14	-0.19032	0.000194	0.00092
PPL	-0.19032	0.000194	0.00092
TSC22D1	-0.1902	0.000196	0.000929
NRXN3	-0.19019	0.000196	0.000929
RNF13	-0.19017	0.000196	0.00093
MEX3B	-0.19016	0.000196	0.000931
IQCA1	-0.19015	0.000196	0.000931
C20orf200	-0.19015	0.000196	0.000931
GPR124	-0.19009	0.000197	0.000935
TMEM35	-0.19008	0.000198	0.000935
USP6NL	-0.19007	0.000198	0.000935
SLC18A3	-0.19006	0.000198	0.000936
SFRS8	-0.19002	0.000198	0.000939
SLC25A32	-0.18995	0.0002	0.000943
ISYNA1	-0.18993	0.0002	0.000945
A4GALT	-0.18987	0.000201	0.000949
OLR1	-0.18982	0.000202	0.000952
ZNF585B	-0.18981	0.000202	0.000953
ZNF528	-0.18976	0.000203	0.000956

C8orf59	-0.18969	0.000204	0.00096
INMT	-0.18958	0.000205	0.000968
MLLT6	-0.18954	0.000206	0.000972
FIZ1	-0.18952	0.000206	0.000973
NTRK3	-0.18945	0.000207	0.000977
LOC21934	-0.18942	0.000208	0.000979
ZNF445	-0.18939	0.000209	0.000981
ORAOV1	-0.18935	0.000209	0.000983
C19orf44	-0.18933	0.00021	0.000985
ING5	-0.18929	0.00021	0.000987
TMC7	-0.18928	0.00021	0.000988
CAMSAP1	-0.18926	0.000211	0.000989
SPINLW1	-0.18915	0.000213	0.000997
C5orf46	-0.18914	0.000213	0.000997
GSK3B	-0.18914	0.000213	0.000997
SOX5	-0.18912	0.000213	0.000998
SENP6	-0.18903	0.000214	0.001005
OBP2A	-0.18896	0.000216	0.00101
ATG4B	-0.18894	0.000216	0.001011
RILPL1	-0.18893	0.000216	0.001012
NRG2	-0.18889	0.000217	0.001015
COL18A1	-0.18887	0.000217	0.001016
CYP2E1	-0.18879	0.000219	0.001022
UBE2R2	-0.18877	0.000219	0.001023
LINGO3	-0.18868	0.00022	0.00103
LOC64698	-0.18868	0.000221	0.00103
KRTAP5-5	-0.18864	0.000221	0.001033
NTF4	-0.18863	0.000221	0.001033
EIF6	-0.18859	0.000222	0.001036
COX7A1	-0.18858	0.000222	0.001037
VPS41	-0.18854	0.000223	0.001039
CLIC4	-0.18854	0.000223	0.001039
KLHL5	-0.18843	0.000225	0.001047
GYLTL1B	-0.18843	0.000225	0.001047
IGF2BP1	-0.18842	0.000225	0.001048
DCLK1	-0.1884	0.000225	0.001049
KCNK17	-0.18834	0.000226	0.001053
TMPRSS9	-0.18832	0.000227	0.001054
ZNF135	-0.18829	0.000227	0.001057
ZFP36L1	-0.18828	0.000228	0.001057
RPS20	-0.18823	0.000228	0.00106
ADD2	-0.18821	0.000229	0.001062
PPP1R15A	-0.18819	0.000229	0.001063
FOXI3	-0.18806	0.000231	0.001072
NHEDC1	-0.18806	0.000231	0.001072
GPR81	-0.18799	0.000233	0.001078
MITF	-0.18793	0.000234	0.001082
CYP26C1	-0.18789	0.000235	0.001085
HSPB2	-0.18786	0.000235	0.001087
SMG1	-0.18785	0.000235	0.001088
PANX2	-0.18784	0.000236	0.001089
DIXDC1	-0.18782	0.000236	0.00109
PACRG	-0.18782	0.000236	0.00109
PDK2	-0.1878	0.000236	0.001091
SH3RF3	-0.18779	0.000236	0.001092
ETV4	-0.18778	0.000237	0.001092
PRRT1	-0.18769	0.000238	0.001099
PNRC1	-0.18769	0.000238	0.001099

ZFP92	-0.18762	0.000239	0.001104
DKFZP586	-0.18756	0.000241	0.00111
ZNF732	-0.18755	0.000241	0.00111
MRVI1	-0.18754	0.000241	0.00111
ZNF229	-0.18752	0.000242	0.001113
RFPL1S	-0.18745	0.000243	0.001118
THSD7A	-0.18744	0.000243	0.001119
C6orf145	-0.18739	0.000244	0.001122
TPTE2P3	-0.18738	0.000244	0.001123
DPY19L3	-0.18736	0.000244	0.001124
ADAMTS1	-0.18735	0.000245	0.001125
MAN2A2	-0.18735	0.000245	0.001125
KCNJ14	-0.18725	0.000247	0.001133
OPN1MW	-0.18717	0.000248	0.001139
PELI2	-0.18716	0.000248	0.00114
MACF1	-0.18713	0.000249	0.001141
ORMDL1	-0.18712	0.000249	0.001142
LOC28636	-0.18707	0.00025	0.001147
NRIP3	-0.187	0.000251	0.001152
SPTBN5	-0.18698	0.000252	0.001154
SLC6A1	-0.18693	0.000253	0.001158
IQCB1	-0.18687	0.000254	0.001163
ILK	-0.18686	0.000254	0.001163
MMP19	-0.18686	0.000254	0.001163
CORIN	-0.1868	0.000255	0.001168
PPP1R2P3	-0.1868	0.000255	0.001168
RRN3	-0.18676	0.000256	0.001171
HDAC11	-0.18671	0.000257	0.001175
ZNF506	-0.18667	0.000258	0.001179
ROBO1	-0.18667	0.000258	0.001179
MRAS	-0.18667	0.000258	0.001179
LMBRD1	-0.18666	0.000258	0.001179
INHA	-0.18665	0.000258	0.00118
EFHA2	-0.18662	0.000259	0.001182
CSE1L	-0.18661	0.000259	0.001183
TMIE	-0.18659	0.00026	0.001183
TAF1C	-0.18654	0.000261	0.001187
TTTY10	-0.18654	0.000261	0.001187
C1orf190	-0.18652	0.000261	0.001189
FAM122C	-0.1865	0.000261	0.00119
AEBP1	-0.1865	0.000261	0.00119
MRGPRE	-0.18642	0.000263	0.001196
TTLL4	-0.18638	0.000264	0.0012
HIST2H2BI	-0.18636	0.000264	0.001201
LOC10013	-0.18634	0.000265	0.001203
PXDNL	-0.18629	0.000266	0.001207
VGLL4	-0.18627	0.000266	0.001208
CLDN16	-0.18625	0.000266	0.001209
SLC41A3	-0.1862	0.000267	0.001214
SPRY1	-0.18618	0.000268	0.001216
ZNF347	-0.1861	0.00027	0.001223
IGFBP5	-0.18605	0.000271	0.001227
BDNFOS	-0.18598	0.000272	0.001233
ETV1	-0.18598	0.000272	0.001233
KIF17	-0.18595	0.000273	0.001236
C2orf44	-0.18591	0.000274	0.001238
MGEA5	-0.18589	0.000274	0.00124
C1QTNF3	-0.18583	0.000275	0.001245

SPACA3	-0.18582	0.000275	0.001246
RRAGB	-0.18572	0.000278	0.001254
LOC64466	-0.18569	0.000278	0.001257
SERPING1	-0.18559	0.00028	0.001266
PCNX	-0.18558	0.000281	0.001267
AMPD2	-0.18558	0.000281	0.001267
FOXK1	-0.18557	0.000281	0.001268
KRTAP3-2	-0.18552	0.000282	0.001271
NPR2	-0.18551	0.000282	0.001272
IL34	-0.18547	0.000283	0.001276
MAPKBP1	-0.18547	0.000283	0.001276
RIMBP2	-0.18547	0.000283	0.001276
C2orf74	-0.1854	0.000284	0.001282
DNAJB6	-0.1854	0.000285	0.001282
SGCE	-0.18538	0.000285	0.001284
LOC10013	-0.18538	0.000285	0.001284
FAM179B	-0.18535	0.000286	0.001286
TCTE1	-0.18534	0.000286	0.001287
RORA	-0.18528	0.000287	0.001292
LRP3	-0.18526	0.000288	0.001293
ABCB4	-0.18521	0.000289	0.001299
IRGC	-0.1852	0.000289	0.001299
SH3BGR	-0.18518	0.000289	0.001301
C7orf26	-0.18514	0.00029	0.001304
TIE1	-0.18514	0.00029	0.001304
LOC64129	-0.18511	0.000291	0.001306
RIMS4	-0.18508	0.000292	0.001309
LRP1	-0.18498	0.000294	0.001319
TBC1D17	-0.18495	0.000295	0.001321
SESTD1	-0.18491	0.000295	0.001324
DENND2C	-0.18491	0.000296	0.001324
PMFBP1	-0.18491	0.000296	0.001324
ATP6V1G2	-0.1849	0.000296	0.001324
KERA	-0.1849	0.000296	0.001324
CTHRC1	-0.18488	0.000296	0.001327
ZNF828	-0.18486	0.000297	0.001328
PSG11	-0.18485	0.000297	0.001328
MED29	-0.18485	0.000297	0.001328
COL20A1	-0.18485	0.000297	0.001328
SLIT2	-0.18483	0.000297	0.001329
FZD7	-0.18481	0.000298	0.001331
PAK3	-0.1848	0.000298	0.001331
SCN2B	-0.18479	0.000298	0.001332
PGBD4	-0.18468	0.000301	0.001342
CCDC154	-0.18468	0.000301	0.001342
ALLC	-0.18467	0.000301	0.001343
MFRP	-0.18464	0.000302	0.001345
ALS2CR12	-0.18464	0.000302	0.001345
EML1	-0.18463	0.000302	0.001346
OTOA	-0.18463	0.000302	0.001346
EXOC7	-0.18459	0.000303	0.00135
EVC	-0.18458	0.000303	0.00135
ZNF493	-0.18458	0.000303	0.00135
MAFG	-0.18457	0.000303	0.001351
LOC92249	-0.18456	0.000304	0.001351
ZNF167	-0.18452	0.000305	0.001355
LRRTM1	-0.18447	0.000306	0.00136
ANGEL1	-0.18429	0.00031	0.001379

TCTE3	-0.18427	0.00031	0.00138
AASS	-0.18421	0.000312	0.001386
FAM171B	-0.18418	0.000312	0.001389
PRKCSH	-0.18417	0.000313	0.00139
POSTN	-0.18413	0.000314	0.001394
C1orf129	-0.18412	0.000314	0.001394
CLVS1	-0.18408	0.000315	0.001398
BPIL2	-0.18405	0.000316	0.0014
ZBTB46	-0.18398	0.000318	0.001407
GUSBL1	-0.18396	0.000318	0.001407
ZNF285	-0.18395	0.000318	0.001408
CHIT1	-0.18394	0.000318	0.001408
FBXO27	-0.18392	0.000319	0.001411
GATSL2	-0.18389	0.00032	0.001413
TPT1	-0.18388	0.00032	0.001413
SEC14L4	-0.18388	0.00032	0.001413
CHD4	-0.18387	0.00032	0.001414
TMEFF1	-0.18381	0.000322	0.00142
ZNF280A	-0.1838	0.000322	0.001421
C12orf68	-0.18378	0.000322	0.001422
ZNF830	-0.18377	0.000323	0.001424
PXMP4	-0.18376	0.000323	0.001424
DYNC1I2	-0.18375	0.000323	0.001425
C7orf40	-0.18374	0.000323	0.001426
RBPMS	-0.18366	0.000325	0.001432
ASB3	-0.18366	0.000325	0.001432
WWTR1	-0.1836	0.000327	0.001438
CLCN6	-0.18358	0.000327	0.001441
ZNF454	-0.18352	0.000329	0.001446
HRC	-0.18344	0.000331	0.001453
ADAMTS3	-0.18337	0.000332	0.00146
ZNF415	-0.18335	0.000333	0.001462
CPN1	-0.18334	0.000333	0.001464
ZNF230	-0.18328	0.000335	0.00147
KIAA0415	-0.18325	0.000336	0.001472
SKI	-0.18323	0.000336	0.001474
C12orf59	-0.18322	0.000336	0.001474
FAM26E	-0.1832	0.000337	0.001477
PLXNA4	-0.18315	0.000338	0.001482
CTCFL	-0.18306	0.00034	0.001491
GREB1	-0.18301	0.000342	0.001497
C3orf34	-0.18297	0.000343	0.001501
PPFIBP1	-0.18292	0.000344	0.001505
LOX	-0.18292	0.000344	0.001505
LMBR1	-0.18289	0.000345	0.001508
SNORD11!	-0.18287	0.000345	0.00151
FOXO1	-0.18286	0.000346	0.001511
USHBP1	-0.18286	0.000346	0.001511
LOXL3	-0.18284	0.000346	0.001513
C12orf53	-0.18284	0.000346	0.001513
SIX4	-0.18283	0.000347	0.001513
UPB1	-0.18282	0.000347	0.001514
C11orf58	-0.1828	0.000347	0.001516
SLC30A2	-0.18275	0.000349	0.001521
SPARC	-0.18274	0.000349	0.001522
SSPO	-0.18271	0.00035	0.001525
CHRNE	-0.18269	0.00035	0.001526
CARD8	-0.18269	0.00035	0.001526

LOC40080	-0.18263	0.000352	0.001533
KIN	-0.18259	0.000353	0.001537
SGIP1	-0.18257	0.000353	0.001538
ZNF778	-0.18253	0.000355	0.001543
FAM184A	-0.18251	0.000355	0.001545
ALX3	-0.18246	0.000356	0.00155
PPAP2B	-0.18244	0.000357	0.001551
CXorf50B	-0.1824	0.000358	0.001556
LOC10013	-0.18239	0.000358	0.001556
INPP4A	-0.18238	0.000359	0.001557
EXOC2	-0.18237	0.000359	0.001557
DNM3	-0.18237	0.000359	0.001558
GDF11	-0.18235	0.000359	0.001559
ZSCAN29	-0.18234	0.00036	0.00156
LOC10012	-0.18233	0.00036	0.00156
IRX1	-0.1823	0.000361	0.001564
LOC40079	-0.18222	0.000363	0.001572
PLCL1	-0.18221	0.000363	0.001573
RHOT1	-0.18219	0.000364	0.001574
ANKRD23	-0.18216	0.000365	0.001577
GATAD2B	-0.18216	0.000365	0.001577
MED12L	-0.18214	0.000365	0.001578
THAP8	-0.18211	0.000366	0.001582
CDH11	-0.18209	0.000367	0.001583
FGF20	-0.18209	0.000367	0.001584
CLEC11A	-0.18205	0.000368	0.001588
FHL1	-0.18204	0.000368	0.001588
TAS2R5	-0.18204	0.000368	0.001588
TPR	-0.18197	0.00037	0.001596
ZNF71	-0.18197	0.00037	0.001596
ATG9A	-0.18196	0.00037	0.001596
TRAPPC2P	-0.18196	0.00037	0.001596
DDHD2	-0.18195	0.00037	0.001597
C7orf61	-0.18191	0.000372	0.001601
MTMR6	-0.18188	0.000373	0.001604
LRP5	-0.1818	0.000375	0.001612
CSPP1	-0.18179	0.000375	0.001613
UTP14A	-0.18175	0.000376	0.001617
SENP7	-0.18169	0.000378	0.001623
PUF60	-0.18167	0.000378	0.001625
CCDC114	-0.18166	0.000379	0.001626
CDH23	-0.18165	0.000379	0.001627
THY1	-0.1816	0.00038	0.001632
ZNF275	-0.18151	0.000383	0.001642
MLC1	-0.18151	0.000383	0.001642
PRR12	-0.18146	0.000384	0.001647
LOC28386	-0.18145	0.000385	0.001648
SPP1	-0.18141	0.000386	0.001653
CRTC3	-0.18138	0.000387	0.001656
NAT8L	-0.18134	0.000388	0.00166
IFFO2	-0.18128	0.00039	0.001667
MLL4	-0.18125	0.000391	0.001669
GON4L	-0.18125	0.000391	0.001669
LRIG1	-0.18122	0.000392	0.001673
SPG20	-0.18118	0.000393	0.001677
USP42	-0.18117	0.000393	0.001678
CC2D2A	-0.18116	0.000393	0.001678
ATP6AP1L	-0.18116	0.000393	0.001679

KIAA1949	-0.18115	0.000394	0.001679
BRD3	-0.18113	0.000394	0.001681
SOX6	-0.18112	0.000394	0.001681
WDR83	-0.18112	0.000394	0.001681
FLJ44635	-0.18111	0.000395	0.001683
AKAP4	-0.18106	0.000396	0.001688
LOC64376	-0.181	0.000398	0.001695
TRO	-0.18099	0.000398	0.001695
RANBP3L	-0.18098	0.000399	0.001697
AQP7P3	-0.18096	0.000399	0.001698
SLC39A13	-0.18096	0.000399	0.001698
TMEM206	-0.18096	0.000399	0.001698
FOXP2	-0.18094	0.0004	0.001701
KCNH3	-0.18093	0.0004	0.001702
PRLHR	-0.18092	0.000401	0.001702
FAM9A	-0.18091	0.000401	0.001703
PRKCDBP	-0.18089	0.000401	0.001704
MAGEE1	-0.18081	0.000404	0.001713
QPRT	-0.1808	0.000404	0.001713
PCDHGB7	-0.18066	0.000408	0.001729
PHF14	-0.18061	0.00041	0.001735
TNNT2	-0.18057	0.000411	0.001739
SMARCC2	-0.18053	0.000412	0.001744
UMODL1	-0.18049	0.000414	0.001749
CCDC126	-0.18047	0.000414	0.001751
MMP13	-0.18041	0.000416	0.001759
WDR59	-0.18035	0.000418	0.001766
NRIP2	-0.18033	0.000419	0.001768
NOVA1	-0.18028	0.00042	0.001774
NFRKB	-0.18025	0.000421	0.001777
LPPR3	-0.18009	0.000426	0.001798
NTNG1	-0.18002	0.000429	0.001807
KLF14	-0.18001	0.000429	0.001807
IWS1	-0.17993	0.000432	0.001817
CDH19	-0.17991	0.000432	0.001819
ZNF565	-0.1799	0.000433	0.00182
FOXD3	-0.17989	0.000433	0.00182
MXRA5	-0.17989	0.000433	0.00182
GPBAR1	-0.17982	0.000435	0.001829
TMEM133	-0.1798	0.000436	0.001831
MEF2C	-0.17979	0.000436	0.001831
MAGEL2	-0.17975	0.000437	0.001836
C19orf26	-0.17974	0.000438	0.001838
CC2D2B	-0.17972	0.000438	0.001839
MAML2	-0.17971	0.000439	0.00184
RASGEF1C	-0.17968	0.00044	0.001844
CASP8AP2	-0.17967	0.00044	0.001844
ZNF83	-0.17963	0.000441	0.001849
MTERFD1	-0.17963	0.000441	0.001849
C11orf61	-0.17963	0.000441	0.001849
RFPL1	-0.17961	0.000442	0.001852
ITGA11	-0.17956	0.000444	0.001858
TWIST1	-0.17951	0.000445	0.001864
CASQ1	-0.1795	0.000446	0.001865
ARMCX2	-0.17948	0.000446	0.001867
CA5B	-0.17944	0.000447	0.001872
SCAP	-0.17938	0.00045	0.001879
IL28RA	-0.17936	0.00045	0.001883

CCDC121	-0.17933	0.000451	0.001886
RHBDF2	-0.17929	0.000453	0.001891
GTF3C5	-0.17927	0.000453	0.001893
NMNAT3	-0.17922	0.000455	0.001899
FLJ45244	-0.17922	0.000455	0.0019
ADRB3	-0.1792	0.000456	0.001902
ARGLU1	-0.17919	0.000456	0.001902
C13orf33	-0.17915	0.000457	0.001908
C3orf18	-0.17915	0.000457	0.001908
YAF2	-0.1791	0.000459	0.001914
GALNT14	-0.17906	0.00046	0.001918
ANKLE1	-0.17903	0.000461	0.001921
L3MBTL	-0.17893	0.000465	0.001936
INHBA	-0.17892	0.000465	0.001937
C13orf35	-0.17883	0.000468	0.001949
COL4A1	-0.17881	0.000469	0.001951
DDX42	-0.1788	0.000469	0.001951
RAB31	-0.17878	0.00047	0.001954
FKBP14	-0.17878	0.00047	0.001954
FAM200A	-0.17877	0.000471	0.001955
VSTM2L	-0.17875	0.000471	0.001957
CDH4	-0.17872	0.000472	0.001961
STL	-0.17869	0.000473	0.001965
ATP2B4	-0.17867	0.000474	0.001967
ACVR2A	-0.17863	0.000475	0.001973
NSMCE2	-0.17863	0.000476	0.001973
RXFP1	-0.17863	0.000476	0.001973
ATXN1L	-0.17857	0.000478	0.00198
SLC38A2	-0.17854	0.000479	0.001983
PLEKHO2	-0.17853	0.000479	0.001984
KLF12	-0.17846	0.000481	0.001992
LOC10024	-0.17837	0.000485	0.002004
YWHAG	-0.17837	0.000485	0.002004
KTELC1	-0.17836	0.000485	0.002006
SGCZ	-0.17831	0.000487	0.002012
ZBTB26	-0.17831	0.000487	0.002012
NMUR1	-0.17827	0.000488	0.002018
SUGT1P1	-0.17824	0.00049	0.002022
OR2A25	-0.17823	0.00049	0.002022
ZNF443	-0.17821	0.000491	0.002025
C17orf80	-0.17815	0.000493	0.002034
ST3GAL6	-0.17814	0.000493	0.002034
MAGED4	-0.17808	0.000495	0.002044
TAF6	-0.17807	0.000496	0.002044
MEAF6	-0.17807	0.000496	0.002044
C5orf49	-0.17805	0.000496	0.002046
SNORD11(	-0.17795	0.0005	0.00206
FIGN	-0.17782	0.000505	0.002079
USP39	-0.1778	0.000506	0.002082
EYS	-0.17778	0.000506	0.002085
PNMA6A	-0.17777	0.000507	0.002086
EVC2	-0.17775	0.000508	0.002088
THRA	-0.17772	0.000509	0.002093
FAM119B	-0.17769	0.00051	0.002097
PHF16	-0.17763	0.000512	0.002106
CLNS1A	-0.17758	0.000514	0.002112
FMR1	-0.17749	0.000518	0.002125
HECA	-0.17739	0.000521	0.00214

KIAA1045	-0.17736	0.000522	0.002144
ARL8A	-0.17736	0.000522	0.002144
MLLT4	-0.17734	0.000523	0.002146
GLI3	-0.17722	0.000528	0.002162
BCL11A	-0.17722	0.000528	0.002163
SOX10	-0.17719	0.000529	0.002166
PLEKHG2	-0.17713	0.000531	0.002174
CHRD	-0.17712	0.000532	0.002175
ZNF148	-0.17709	0.000533	0.002179
ORC3L	-0.17703	0.000535	0.002189
CALCOCO	-0.17699	0.000537	0.002195
NBPF14	-0.17697	0.000538	0.002197
RAB34	-0.17687	0.000542	0.002213
PIK3IP1	-0.1768	0.000545	0.002224
LBX2	-0.1768	0.000545	0.002224
GPATCH2	-0.17677	0.000546	0.002227
CD109	-0.17674	0.000547	0.002231
KIAA1826	-0.17673	0.000547	0.002232
PHLDB2	-0.17673	0.000548	0.002232
IGSF11	-0.17659	0.000553	0.00225
SNORD11(	-0.17658	0.000553	0.002251
NGRN	-0.17658	0.000554	0.002252
MAGEH1	-0.17658	0.000554	0.002252
LOC10013	-0.17653	0.000555	0.002257
SND1	-0.17651	0.000556	0.00226
ZNF750	-0.17651	0.000556	0.00226
ZNF197	-0.17651	0.000556	0.00226
GABRA3	-0.17648	0.000557	0.002263
C22orf23	-0.17644	0.000559	0.00227
THBS3	-0.1764	0.000561	0.002276
PCDHGB5	-0.17638	0.000562	0.002278
ELMOD1	-0.17637	0.000562	0.002278
PCDHA6	-0.17636	0.000562	0.00228
PAN2	-0.17636	0.000563	0.00228
MTTP	-0.17633	0.000564	0.002283
OLFML3	-0.17627	0.000566	0.002293
ARL4C	-0.17626	0.000567	0.002294
EFS	-0.17626	0.000567	0.002294
OTOF	-0.17622	0.000568	0.002299
CFLP1	-0.17613	0.000572	0.002313
TNFSF4	-0.17612	0.000573	0.002315
RPLP0P2	-0.17611	0.000573	0.002315
OSBPL8	-0.17607	0.000575	0.002322
NISCH	-0.17606	0.000575	0.002322
PLEKHH2	-0.17605	0.000575	0.002323
C10orf10	-0.17605	0.000575	0.002323
HECTD2	-0.17594	0.00058	0.00234
PBX3	-0.1759	0.000582	0.002346
BEND6	-0.17585	0.000584	0.002354
KBTBD7	-0.17582	0.000585	0.002358
HOXA5	-0.17579	0.000587	0.002363
EIF2C4	-0.17578	0.000587	0.002363
GAB2	-0.17572	0.000589	0.002372
PEX12	-0.17564	0.000593	0.002385
ZNF665	-0.17559	0.000595	0.002394
LOC65378	-0.17556	0.000596	0.002398
C13orf36	-0.17556	0.000597	0.002398
LINGO2	-0.17554	0.000597	0.0024

FYN	-0.17553	0.000598	0.002401
ARIH1	-0.17548	0.0006	0.00241
C8orf51	-0.17544	0.000602	0.002415
SLIT3	-0.17541	0.000603	0.002419
ENDOU	-0.1754	0.000603	0.00242
TRPS1	-0.17539	0.000604	0.002423
FTCD	-0.17538	0.000604	0.002423
REV3L	-0.17531	0.000608	0.002435
SLITRK4	-0.1753	0.000608	0.002436
DPYSL2	-0.17527	0.000609	0.002439
PLXNA3	-0.17521	0.000612	0.002448
TRIM46	-0.17521	0.000612	0.002448
LHX3	-0.17516	0.000614	0.002456
SYNPO2L	-0.17515	0.000615	0.002456
RAI2	-0.17515	0.000615	0.002457
FGD5	-0.17513	0.000616	0.00246
RFXAP	-0.17512	0.000616	0.00246
EYA1	-0.17512	0.000616	0.00246
EFHA1	-0.17508	0.000618	0.002467
TNPO2	-0.17508	0.000618	0.002467
CRMP1	-0.17498	0.000622	0.002482
NEK9	-0.17496	0.000623	0.002485
PLB1	-0.17496	0.000623	0.002485
C10orf90	-0.17491	0.000625	0.002492
PLTP	-0.17489	0.000626	0.002496
C2orf49	-0.17487	0.000627	0.0025
GALNTL1	-0.17486	0.000628	0.0025
GTF2I	-0.17486	0.000628	0.0025
ZNF701	-0.17479	0.000631	0.002513
WLS	-0.17479	0.000631	0.002513
RBL1	-0.17478	0.000632	0.002514
ANKRD49	-0.17474	0.000633	0.002519
TP53BP2	-0.17474	0.000633	0.002519
MACROD2	-0.17472	0.000634	0.002523
KIFC3	-0.17471	0.000635	0.002523
BTBD11	-0.1747	0.000635	0.002525
BRD2	-0.17464	0.000638	0.002535
TCAM1P	-0.17463	0.000638	0.002535
PLXNB1	-0.1746	0.00064	0.002539
KRT35	-0.17455	0.000642	0.002547
NXPH2	-0.17448	0.000645	0.002558
FUT1	-0.17448	0.000645	0.002558
BMP4	-0.17446	0.000646	0.002562
GTF2E1	-0.17443	0.000648	0.002567
MRGPRD	-0.17438	0.00065	0.002574
SBF2	-0.17437	0.000651	0.002576
CNKSR2	-0.17434	0.000652	0.002581
FAM75A6	-0.17432	0.000653	0.002584
MRFAP1L1	-0.1743	0.000654	0.002588
SOS1	-0.17429	0.000654	0.002588
ORC2L	-0.17429	0.000654	0.002588
RNF44	-0.17428	0.000655	0.00259
HLX	-0.17428	0.000655	0.00259
EPYC	-0.17426	0.000656	0.002592
C6orf155	-0.17424	0.000657	0.002594
NKPD1	-0.17415	0.000661	0.00261
USP50	-0.17411	0.000663	0.002617
CBLN1	-0.17409	0.000664	0.002619

C6orf41	-0.17408	0.000665	0.002621
PPP3R2	-0.17407	0.000665	0.002622
ELOVL4	-0.17405	0.000666	0.002624
GPR144	-0.17405	0.000666	0.002625
UPF1	-0.17402	0.000668	0.00263
HDAC2	-0.17401	0.000668	0.002631
HAPLN4	-0.17399	0.000669	0.002632
FBN1	-0.17399	0.000669	0.002632
ZNF564	-0.17396	0.00067	0.002636
TRAF5	-0.17395	0.000671	0.002637
NPEPL1	-0.17395	0.000671	0.002637
NCKAP1	-0.1739	0.000673	0.002646
HRNBP3	-0.17387	0.000675	0.002652
NECAB3	-0.17385	0.000676	0.002654
OBFC2A	-0.17384	0.000676	0.002656
ATP6V0A4	-0.17383	0.000676	0.002657
SPTBN4	-0.17381	0.000678	0.002662
LOC28305	-0.17377	0.00068	0.002668
CSRP2	-0.17374	0.000681	0.002672
SPSB4	-0.17373	0.000682	0.002674
NBPF15	-0.17367	0.000685	0.002684
NHEDC2	-0.17366	0.000685	0.002685
NLGN3	-0.17365	0.000686	0.002687
HS2ST1	-0.17362	0.000687	0.002691
TLK2	-0.17358	0.000689	0.002698
THOC2	-0.17357	0.00069	0.002701
GLTSCR2	-0.17356	0.00069	0.002702
PLSCR3	-0.17351	0.000693	0.002711
MEGF8	-0.17351	0.000693	0.002711
TRIM4	-0.17346	0.000695	0.002718
NCAM2	-0.17346	0.000695	0.002718
ZNF621	-0.17345	0.000696	0.00272
NEBL	-0.17337	0.0007	0.002733
SCG2	-0.17336	0.0007	0.002733
RARS2	-0.17334	0.000701	0.002737
ZNF675	-0.17333	0.000702	0.002739
ANGEL2	-0.17328	0.000704	0.002749
MT3	-0.17323	0.000707	0.002758
TGFBR2	-0.17322	0.000707	0.002758
MMP11	-0.17322	0.000707	0.002758
SARDH	-0.1732	0.000708	0.002762
KCNT1	-0.17316	0.00071	0.002767
BAMBI	-0.17312	0.000712	0.002775
RANBP6	-0.17312	0.000713	0.002775
BNC2	-0.17311	0.000713	0.002777
FLOT1	-0.17308	0.000715	0.002782
C1orf101	-0.17302	0.000717	0.002792
C20orf108	-0.17301	0.000718	0.002795
LAMA4	-0.17299	0.000719	0.002797
CLK4	-0.17293	0.000722	0.00281
CSMD2	-0.17288	0.000725	0.002818
TXNDC2	-0.17286	0.000726	0.002821
DNAH12	-0.17285	0.000726	0.002823
ASH1L	-0.17285	0.000727	0.002823
COL11A1	-0.17283	0.000728	0.002825
LOC33904	-0.17282	0.000728	0.002826
SOX17	-0.17281	0.000729	0.002829
WNT5B	-0.1728	0.000729	0.00283

LOC64874	-0.17273	0.000733	0.002844
ZNF420	-0.17269	0.000735	0.002851
ZNF248	-0.17265	0.000737	0.002859
RYR2	-0.17255	0.000742	0.002878
PI15	-0.17252	0.000744	0.002884
MGC4210!	-0.17246	0.000747	0.002894
CDR2	-0.17246	0.000747	0.002894
MEPCE	-0.17242	0.000749	0.002901
CST5	-0.17239	0.000751	0.002905
ZNF280C	-0.17237	0.000752	0.002909
CUBN	-0.17236	0.000753	0.002911
ZNF226	-0.17234	0.000753	0.002913
PARP4	-0.17228	0.000757	0.002926
CDH9	-0.17225	0.000759	0.002932
BCO2	-0.17224	0.000759	0.002932
ZNF570	-0.17217	0.000763	0.002947
TMEM86A	-0.17216	0.000763	0.002947
NCRNA00	-0.1721	0.000767	0.002959
NAP1L1	-0.17209	0.000767	0.00296
HSPG2	-0.17206	0.000769	0.002965
PDE1A	-0.172	0.000772	0.002975
NONO	-0.17197	0.000774	0.002982
CLK2	-0.17192	0.000777	0.002991
VAX2	-0.17192	0.000777	0.002991
C9orf102	-0.17189	0.000778	0.002996
RNF216L	-0.17184	0.000781	0.003007
PTPRM	-0.17182	0.000782	0.003009
C3orf59	-0.17182	0.000782	0.003009
IGFL4	-0.17181	0.000783	0.00301
USP47	-0.1718	0.000783	0.003012
CHSY3	-0.17178	0.000785	0.003016
ZNF790	-0.17177	0.000785	0.003018
EPHA5	-0.17172	0.000788	0.003026
CCT6A	-0.17172	0.000788	0.003026
TERF1	-0.1717	0.000789	0.00303
DSCR6	-0.1717	0.000789	0.00303
BEX4	-0.17168	0.00079	0.003032
CNOT4	-0.17168	0.00079	0.003032
SAV1	-0.17165	0.000792	0.003039
MGMT	-0.17164	0.000793	0.00304
VCX3A	-0.1716	0.000795	0.003047
ABHD13	-0.17155	0.000797	0.003057
ARSI	-0.17152	0.000799	0.003062
WDFY3	-0.17151	0.0008	0.003065
C14orf132	-0.17143	0.000804	0.00308
DUXA	-0.17143	0.000805	0.003081
TMED4	-0.1714	0.000806	0.003085
GNAI2	-0.17139	0.000807	0.003087
CUEDC1	-0.17138	0.000808	0.003089
RHBDD2	-0.17138	0.000808	0.003089
SLC24A5	-0.17137	0.000808	0.00309
NRN1L	-0.17136	0.000809	0.003092
ZNF616	-0.17131	0.000811	0.003101
KCNA7	-0.17125	0.000815	0.003114
MYO7A	-0.17124	0.000815	0.003115
ZNF101	-0.17123	0.000816	0.003118
GABRA5	-0.17113	0.000822	0.003137
ANKRD13I	-0.17108	0.000825	0.003146

ZNF44	-0.17105	0.000827	0.003152
ZNF880	-0.17102	0.000828	0.003157
TMEM31	-0.17101	0.000829	0.00316
RALYL	-0.17101	0.000829	0.00316
TROVE2	-0.17097	0.000832	0.003168
IPW	-0.17093	0.000834	0.003175
PSTPIP2	-0.17091	0.000835	0.003179
LUC7L2	-0.17088	0.000837	0.003183
DSC1	-0.17086	0.000838	0.003188
HAMP	-0.17082	0.000841	0.003196
PDGFRB	-0.1708	0.000842	0.0032
NCRNA00(	-0.17079	0.000842	0.003201
MAD1L1	-0.17074	0.000845	0.003211
PPM1F	-0.17071	0.000847	0.003216
TRMT112	-0.17071	0.000847	0.003216
FNDC5	-0.17067	0.000849	0.003223
SPDYA	-0.17064	0.000851	0.003231
KIAA1024	-0.17056	0.000856	0.003247
ANKRD11	-0.17052	0.000858	0.003254
C22orf26	-0.17048	0.000861	0.003263
ALG11	-0.17047	0.000862	0.003265
POM121	-0.17047	0.000862	0.003266
CPNE3	-0.17045	0.000863	0.003268
RTN4RL2	-0.17043	0.000864	0.003272
MCAM	-0.17043	0.000864	0.003273
SIAH3	-0.1704	0.000866	0.003278
ZNF470	-0.17037	0.000868	0.003284
C2orf15	-0.17037	0.000868	0.003284
NUDCD3	-0.17035	0.000869	0.003287
SCARNA6	-0.17024	0.000876	0.003312
MLL3	-0.17024	0.000876	0.003313
ZNF331	-0.17021	0.000878	0.003318
GOLGA8B	-0.17019	0.000879	0.003322
MAF1	-0.17012	0.000883	0.003337
CIAO1	-0.17012	0.000883	0.003337
CYP17A1	-0.17011	0.000884	0.003339
MEF2A	-0.17006	0.000887	0.003349
MUCL1	-0.17003	0.000889	0.003355
AQP7P1	-0.17002	0.00089	0.003356
DCTN1	-0.17002	0.00089	0.003356
TUBB1	-0.17	0.000891	0.003358
EEF1D	-0.17	0.000891	0.003359
RRAGD	-0.16998	0.000892	0.003361
TSHZ3	-0.16996	0.000894	0.003365
ARL11	-0.16994	0.000895	0.003368
ATXN7L1	-0.1699	0.000898	0.003377
HOXC11	-0.16989	0.000898	0.003378
KCNA1	-0.16989	0.000898	0.003379
ACD	-0.16987	0.0009	0.003382
DOCK1	-0.16986	0.0009	0.003382
FXYD2	-0.16985	0.000901	0.003384
GOLGA6B	-0.16981	0.000903	0.00339
ELF5	-0.16981	0.000903	0.00339
ALG5	-0.1698	0.000904	0.003391
LOC10013	-0.16973	0.000908	0.003407
MOSPD3	-0.16972	0.000909	0.003409
GGA2	-0.16972	0.000909	0.00341
SAMD4A	-0.16969	0.000911	0.003415

ZXDC	-0.16966	0.000913	0.003421
SLC25A30	-0.16965	0.000914	0.003425
IL22RA1	-0.16964	0.000914	0.003425
OSBPL10	-0.16964	0.000914	0.003425
LCN12	-0.16959	0.000918	0.003436
ZNF385A	-0.16958	0.000918	0.003436
HDAC7	-0.16954	0.000921	0.003447
ABCA1	-0.16952	0.000922	0.003449
KIAA0831	-0.1695	0.000923	0.003455
GBGT1	-0.16948	0.000925	0.003458
NPAS2	-0.16942	0.000929	0.003472
ZNF211	-0.16937	0.000932	0.003481
POLM	-0.1693	0.000936	0.003496
KRTAP10-	-0.1693	0.000937	0.003496
KIAA1147	-0.16927	0.000938	0.003501
RNF139	-0.16924	0.00094	0.003507
AFAP1L1	-0.16922	0.000942	0.003513
FAM76B	-0.16914	0.000947	0.003531
GALNT13	-0.16907	0.000952	0.003547
LUZP6	-0.16906	0.000952	0.003549
PROS1	-0.16905	0.000953	0.00355
MYOZ1	-0.16902	0.000955	0.003557
C6orf170	-0.16901	0.000956	0.003558
LPAR4	-0.16896	0.000959	0.003569
TEKT4	-0.16895	0.00096	0.003571
PJA1	-0.16888	0.000965	0.003587
MED25	-0.16884	0.000968	0.003597
TAF1	-0.16877	0.000972	0.003613
TAS2R10	-0.16876	0.000973	0.003613
GXYLT1	-0.16876	0.000973	0.003614
NEB	-0.16874	0.000974	0.003616
ZNF559	-0.16874	0.000974	0.003617
LRRC4	-0.16874	0.000974	0.003617
SF4	-0.16867	0.000979	0.003631
FOXO4	-0.16863	0.000982	0.003642
LRRC70	-0.16861	0.000983	0.003645
BRAP	-0.16853	0.000989	0.003665
WNK1	-0.16846	0.000994	0.00368
PLAC1	-0.16843	0.000996	0.003685
C17orf100	-0.16841	0.000997	0.003689
KRT83	-0.16834	0.001002	0.003706
NPTX2	-0.16832	0.001003	0.00371
PM20D2	-0.16826	0.001008	0.003725
LMCD1	-0.16825	0.001009	0.003727
GDF10	-0.16821	0.001011	0.003735
FLRT2	-0.16821	0.001012	0.003735
GRK7	-0.1682	0.001012	0.003737
DMP1	-0.16807	0.001022	0.00377
VPS24	-0.16804	0.001024	0.003776
NIN	-0.16804	0.001024	0.003776
CBX1	-0.16803	0.001024	0.003778
ERAS	-0.16796	0.00103	0.003795
IKZF4	-0.16792	0.001032	0.003802
TTF1	-0.16786	0.001037	0.003818
CILP	-0.16786	0.001037	0.003818
KCNJ8	-0.16784	0.001038	0.003822
FAM182A	-0.16781	0.00104	0.003828
SLC12A9	-0.16778	0.001043	0.003836

PAAF1	-0.16775	0.001045	0.003842
ESYT3	-0.16773	0.001046	0.003848
FAM155A	-0.16771	0.001048	0.003853
KIAA0174	-0.1677	0.001049	0.003855
LRRC61	-0.16768	0.00105	0.003858
C1orf156	-0.16767	0.001051	0.00386
CYP46A1	-0.16765	0.001052	0.003864
SHARPIN	-0.16765	0.001052	0.003864
HTRA4	-0.16759	0.001057	0.00388
CACHD1	-0.16757	0.001058	0.003883
EFNA3	-0.16756	0.001059	0.003885
MERTK	-0.16755	0.001059	0.003886
FAM129A	-0.16748	0.001065	0.003906
ZCCHC18	-0.16747	0.001065	0.003907
TENC1	-0.16746	0.001066	0.003909
DACH1	-0.16746	0.001066	0.003909
ZC3H18	-0.16741	0.00107	0.003921
TMCC2	-0.16739	0.001071	0.003924
FLNC	-0.16739	0.001071	0.003924
ZNF876P	-0.16737	0.001073	0.003929
MPP4	-0.16737	0.001073	0.003929
PDPR	-0.16735	0.001075	0.003934
ZNF446	-0.16734	0.001075	0.003937
LOC28479	-0.16731	0.001077	0.003942
SUMO4	-0.16727	0.00108	0.003952
LRRC14B	-0.16725	0.001082	0.003956
COL5A2	-0.1672	0.001085	0.003969
CHRNB2	-0.1672	0.001086	0.00397
TIMP2	-0.16715	0.00109	0.003983
ATP13A3	-0.16712	0.001092	0.00399
TM7SF4	-0.16708	0.001095	0.003999
CADM1	-0.16706	0.001097	0.004005
CLDN1	-0.16702	0.001099	0.004013
ARHGEF33	-0.16699	0.001102	0.004021
ARHGEF12	-0.16697	0.001103	0.004026
OSCAR	-0.16695	0.001105	0.00403
ZNF579	-0.16695	0.001105	0.00403
CXorf36	-0.16695	0.001105	0.00403
CLTCL1	-0.16694	0.001106	0.00403
TMEM117	-0.16694	0.001106	0.00403
ITGB1BP2	-0.16688	0.00111	0.004044
C11orf36	-0.16683	0.001114	0.004057
GDF6	-0.16683	0.001114	0.004057
CCDC54	-0.16678	0.001118	0.004071
MSL3L2	-0.16674	0.001121	0.004079
ZNF280D	-0.16673	0.001122	0.004082
THADA	-0.1667	0.001124	0.004088
RECK	-0.16669	0.001125	0.004092
LEPRE1	-0.16667	0.001127	0.004095
PSMA7	-0.16666	0.001127	0.004097
RNF25	-0.16664	0.001129	0.004101
C22orf36	-0.16656	0.001135	0.004123
ABCC1	-0.16652	0.001139	0.004133
COL15A1	-0.16651	0.001139	0.004134
SMEK2	-0.16651	0.00114	0.004134
RBMS3	-0.16647	0.001143	0.004143
GPR143	-0.16646	0.001143	0.004143
TM4SF19	-0.16643	0.001146	0.004151

PLEKHA5	-0.16638	0.00115	0.004165
FAM160B1	-0.16634	0.001153	0.004177
TRRAP	-0.1663	0.001156	0.004185
HIGD1B	-0.16629	0.001157	0.004187
C10orf41	-0.16628	0.001158	0.00419
ERC2	-0.16625	0.001161	0.004199
ALDH5A1	-0.16624	0.001161	0.0042
REM1	-0.16621	0.001163	0.004205
IGFL1	-0.16621	0.001163	0.004206
CPSF7	-0.1662	0.001164	0.004208
LRRC69	-0.1662	0.001164	0.004208
ZCWPW2	-0.16615	0.001168	0.004221
TCF15	-0.1661	0.001172	0.004233
RPS15AP1	-0.16602	0.001179	0.004255
NEFH	-0.16601	0.00118	0.004257
LOC10013	-0.16595	0.001185	0.004273
PKLR	-0.16592	0.001187	0.00428
ZNF835	-0.16588	0.00119	0.004288
WDR45	-0.16583	0.001195	0.004301
LOC91149	-0.16583	0.001195	0.004301
GPATCH1	-0.16579	0.001198	0.004309
FBLN5	-0.16576	0.0012	0.004318
CSMD3	-0.16573	0.001203	0.004325
ZNF525	-0.16569	0.001206	0.004337
HS1BP3	-0.16566	0.001209	0.004346
GRIK2	-0.16563	0.001211	0.004351
ZNF649	-0.16563	0.001211	0.004352
ITGB1	-0.16562	0.001212	0.004352
ZNF625	-0.16562	0.001212	0.004352
GULP1	-0.16562	0.001213	0.004354
ZNHIT6	-0.16557	0.001216	0.004364
MGC1614	-0.16555	0.001218	0.004368
WDR88	-0.16555	0.001218	0.00437
TUBB6	-0.16553	0.00122	0.004375
DENND5B	-0.16551	0.001222	0.004379
PLGLB2	-0.16549	0.001223	0.004383
BLCAP	-0.16547	0.001225	0.004388
IFT46	-0.16546	0.001225	0.00439
RGAG1	-0.16544	0.001228	0.004396
HERC5	-0.16536	0.001234	0.004416
APOLD1	-0.16534	0.001236	0.004423
LDOC1	-0.16533	0.001237	0.004423
PPM1E	-0.1653	0.00124	0.004433
BAT4	-0.16528	0.001241	0.004438
ZNF582	-0.16526	0.001243	0.004443
CST1	-0.16523	0.001246	0.004449
XAB2	-0.16517	0.00125	0.004464
NCS1	-0.16511	0.001256	0.004483
CCDC46	-0.16505	0.001261	0.004498
ANKRD13I	-0.16505	0.001261	0.004499
NCRNA00	-0.16495	0.00127	0.004528
ZNF841	-0.16495	0.00127	0.004529
CLIP1	-0.16493	0.001271	0.004533
HCG22	-0.1649	0.001274	0.004542
C1orf126	-0.16486	0.001277	0.004551
PCYOX1	-0.16476	0.001286	0.004582
PRSS55	-0.16476	0.001287	0.004582
C11orf65	-0.16475	0.001287	0.004582

CCDC55	-0.16474	0.001288	0.004584
NOVA2	-0.16473	0.001289	0.004587
HPS4	-0.16473	0.001289	0.004587
WDR17	-0.16473	0.00129	0.004587
DSE	-0.16471	0.001291	0.004593
STRN4	-0.16463	0.001298	0.004615
RGS5	-0.16459	0.001302	0.004622
FBXO21	-0.16455	0.001305	0.00463
MAMDC2	-0.16453	0.001307	0.004636
BCL6B	-0.16446	0.001314	0.004657
DAAM2	-0.16443	0.001316	0.004664
ZFC3H1	-0.16442	0.001317	0.004669
CST2	-0.16435	0.001323	0.004687
SPIRE2	-0.16434	0.001324	0.00469
EBF3	-0.16428	0.001329	0.004707
C5orf13	-0.16425	0.001333	0.004717
DPYSL5	-0.16423	0.001335	0.004724
CCDC89	-0.1642	0.001337	0.004729
PCDHA10	-0.16419	0.001338	0.00473
AIG1	-0.1641	0.001347	0.00476
OGT	-0.16409	0.001348	0.004762
RPIA	-0.16408	0.001348	0.004762
BBX	-0.16405	0.001351	0.004773
HIST4H4	-0.16402	0.001354	0.004782
GLI1	-0.16399	0.001356	0.00479
SLC8A2	-0.16395	0.00136	0.004802
ZBTB34	-0.16392	0.001363	0.004812
TTLL2	-0.16387	0.001368	0.004827
ENAH	-0.16386	0.001368	0.004827
GPR146	-0.16372	0.001382	0.00487
KIAA1210	-0.16371	0.001383	0.004874
AP1S2	-0.16363	0.00139	0.004896
ZNF461	-0.16363	0.001391	0.004896
HFM1	-0.16363	0.001391	0.004896
RPL37A	-0.1636	0.001393	0.004904
GZF1	-0.16356	0.001398	0.004918
GLIPR1L2	-0.16354	0.0014	0.004924
KIAA0776	-0.16353	0.001401	0.004926
LFNG	-0.16352	0.001401	0.004927
CLIP4	-0.16352	0.001401	0.004927
GGT5	-0.1635	0.001403	0.00493
HNRPDL	-0.16348	0.001406	0.004936
FAM122B	-0.16347	0.001406	0.004938
PURG	-0.16346	0.001407	0.004938
ZC3HC1	-0.16338	0.001414	0.004962
ZNF549	-0.16334	0.001418	0.004976
GNAO1	-0.16333	0.001419	0.004978
DDX18	-0.16331	0.001422	0.004986
P2RX7	-0.16327	0.001426	0.004998
EEPD1	-0.16326	0.001426	0.005
ZNF761	-0.16323	0.00143	0.00501
VKORC1L1	-0.16319	0.001433	0.00502
NEK7	-0.16316	0.001436	0.005029
WAC	-0.16315	0.001437	0.005031
PPP1R1A	-0.16309	0.001443	0.005052
ZNF772	-0.16308	0.001444	0.005052
XPO1	-0.16306	0.001446	0.005057
TMEM74	-0.16299	0.001453	0.005078

FYTTD1	-0.16293	0.001459	0.005097
CNTN2	-0.16291	0.001461	0.005102
ERBB3	-0.16287	0.001465	0.005113
FGFRL1	-0.16286	0.001466	0.005113
C20orf151	-0.16286	0.001466	0.005115
SIRPA	-0.16283	0.001469	0.005125
ASIP	-0.16281	0.001471	0.005131
LOC84931	-0.1628	0.001472	0.005133
PHF12	-0.16277	0.001475	0.00514
VIM	-0.16264	0.001489	0.005187
KRT222	-0.16261	0.001491	0.005196
MTDH	-0.1626	0.001492	0.005197
AKAP8	-0.1626	0.001492	0.005197
C17orf57	-0.16258	0.001494	0.005203
KIRREL	-0.16257	0.001496	0.005207
C1QTNF9	-0.16251	0.001502	0.005227
CHPF	-0.16246	0.001506	0.005242
CYP21A2	-0.16242	0.001511	0.005256
LOC90586	-0.16239	0.001513	0.005263
TRAM1	-0.16239	0.001514	0.005263
UBE2QL1	-0.16239	0.001514	0.005263
FADS6	-0.16238	0.001515	0.005265
EGR2	-0.16234	0.001519	0.005279
RPL31P11	-0.16234	0.001519	0.00528
RHOJ	-0.16229	0.001524	0.005294
PPAPDC1A	-0.16226	0.001528	0.005306
USP31	-0.16209	0.001545	0.005363
TCEAL1	-0.16203	0.001551	0.005383
C21orf99	-0.16203	0.001551	0.005383
EFEMP1	-0.16202	0.001552	0.005386
PDE8B	-0.16198	0.001557	0.005398
PLEKHA8	-0.16196	0.001558	0.005403
RYR3	-0.16195	0.00156	0.005406
COL3A1	-0.16194	0.001561	0.005408
HOXA6	-0.16192	0.001563	0.005416
LOH12CR2	-0.16191	0.001564	0.005418
UCMA	-0.16191	0.001564	0.005418
IMPAD1	-0.16183	0.001573	0.005443
TOR1AIP1	-0.16182	0.001574	0.005445
SMOC1	-0.16181	0.001574	0.005447
FTSJD2	-0.16174	0.001582	0.00547
HTRA3	-0.16174	0.001582	0.00547
COL24A1	-0.16171	0.001585	0.005478
SHC1	-0.16171	0.001586	0.00548
SMC3	-0.16166	0.001591	0.005494
HSD11B1L	-0.16165	0.001592	0.005495
MDGA2	-0.16164	0.001593	0.005499
NDNL2	-0.16162	0.001596	0.005505
SLMO2	-0.16157	0.001601	0.005522
ATP6V1H	-0.16155	0.001603	0.005527
ZNF114	-0.16154	0.001603	0.005528
UVRAG	-0.16153	0.001604	0.005531
RFX8	-0.16147	0.001611	0.005552
C14orf37	-0.16145	0.001614	0.005559
UBXN1	-0.16142	0.001617	0.005568
DUSP28	-0.16138	0.001621	0.005582
PUS7	-0.16137	0.001623	0.005587
KREMEN1	-0.16134	0.001626	0.005596

DGAT2	-0.16132	0.001628	0.005601
COPZ2	-0.16131	0.001629	0.005604
ZNF526	-0.16125	0.001636	0.005626
ITGA5	-0.16125	0.001636	0.005626
PTPN5	-0.16121	0.001641	0.00564
STK25	-0.1612	0.001641	0.00564
CALCRL	-0.16118	0.001643	0.005644
TMCC1	-0.16118	0.001643	0.005644
NBR1	-0.16118	0.001644	0.005645
C1QTNF8	-0.16117	0.001645	0.005647
SH3TC1	-0.16107	0.001656	0.00568
LOC72887	-0.16106	0.001656	0.005681
SGCG	-0.16104	0.001659	0.005688
LGI4	-0.16104	0.00166	0.00569
LOC55011	-0.16101	0.001663	0.005699
RGS22	-0.161	0.001664	0.005699
ZNF175	-0.16096	0.001668	0.00571
FMNL2	-0.16093	0.001672	0.005722
TRAM1L1	-0.16093	0.001672	0.005722
HSD17B3	-0.16085	0.001681	0.005752
FLT1	-0.16084	0.001681	0.005752
TMEM132I	-0.16083	0.001683	0.005756
ARMCX3	-0.16082	0.001684	0.005759
SEMA5B	-0.16081	0.001686	0.005761
C14orf39	-0.1608	0.001686	0.005761
RPL8	-0.16077	0.00169	0.005773
C7orf29	-0.16076	0.00169	0.005774
RFX3	-0.16074	0.001693	0.00578
SERPINI2	-0.16072	0.001695	0.005787
NPFFR1	-0.16071	0.001697	0.005791
FAM190A	-0.1607	0.001698	0.005794
ZC3H7A	-0.16069	0.001699	0.005795
TSNAX-DI	-0.16067	0.001701	0.0058
KLHL7	-0.16067	0.001701	0.0058
DNM1P35	-0.16066	0.001702	0.005804
XPO5	-0.16063	0.001705	0.005813
EFHB	-0.16063	0.001706	0.005814
KDM5A	-0.16062	0.001707	0.005815
TPTE2P1	-0.16059	0.00171	0.005824
TNC	-0.16055	0.001715	0.005841
CD81	-0.16052	0.001719	0.005852
KCTD18	-0.16051	0.00172	0.005854
RNF2	-0.16048	0.001723	0.005864
GDF7	-0.16047	0.001724	0.005867
PSG1	-0.16043	0.001729	0.005884
RELL2	-0.16042	0.00173	0.005884
KRT80	-0.1604	0.001733	0.005893
ZNF281	-0.16037	0.001736	0.005903
ZNF208	-0.16033	0.00174	0.005917
ZNF43	-0.16032	0.001742	0.005921
ITGA9	-0.16029	0.001746	0.005932
GRIA2	-0.16028	0.001747	0.005934
C1orf26	-0.16026	0.001749	0.005941
NAIF1	-0.16023	0.001753	0.005952
SCN1B	-0.16011	0.001766	0.005995
IQSEC3	-0.16009	0.001769	0.006005
C6orf64	-0.16007	0.001771	0.00601
PLXDC1	-0.16005	0.001774	0.006019

LOC10027	-0.16004	0.001775	0.006022
GPX4	-0.16003	0.001776	0.006024
PCDH12	-0.15993	0.001788	0.006062
RPH3A	-0.15992	0.00179	0.006065
TMEM151I	-0.15982	0.001801	0.006099
CCDC40	-0.15979	0.001805	0.006109
EMILIN1	-0.15978	0.001806	0.006112
FOXN3	-0.15976	0.001809	0.00612
C17orf69	-0.15974	0.001811	0.006125
SVIL	-0.15971	0.001815	0.006136
PRPF3	-0.15969	0.001817	0.006139
ZNF674	-0.15969	0.001817	0.00614
VMAC	-0.15968	0.001819	0.006144
FXYD1	-0.15964	0.001823	0.006156
SLC7A14	-0.15961	0.001827	0.006167
TUBE1	-0.1596	0.001828	0.006171
ZNF181	-0.15956	0.001833	0.006183
PAGE2B	-0.15954	0.001836	0.006188
THRSP	-0.15954	0.001836	0.006188
GJD4	-0.15948	0.001842	0.006209
SP9	-0.15948	0.001843	0.006209
AKAP9	-0.15947	0.001845	0.006214
C4orf39	-0.15944	0.001848	0.006225
NSMCE1	-0.15937	0.001856	0.006251
RNF113A	-0.15937	0.001857	0.006251
SPSB1	-0.15934	0.00186	0.00626
BZRAP1	-0.15932	0.001863	0.006268
KLHL10	-0.15931	0.001864	0.006269
DYNC1LI2	-0.1593	0.001865	0.006271
PAQR6	-0.15928	0.001868	0.00628
DUSP26	-0.15924	0.001873	0.006292
FAM13C	-0.15922	0.001875	0.006298
ARL4A	-0.15916	0.001883	0.006324
SSBP4	-0.15913	0.001886	0.006332
VEGFA	-0.15913	0.001887	0.006334
VSTM2B	-0.1591	0.00189	0.006344
PLEKHG4B	-0.15905	0.001896	0.006361
JUN	-0.15899	0.001904	0.006385
PUM1	-0.15889	0.001917	0.006419
GNB4	-0.15885	0.001922	0.006435
LOC90110	-0.15884	0.001924	0.006439
ZNF30	-0.1588	0.001929	0.006456
ACAD11	-0.15879	0.001929	0.006456
SPAG9	-0.15875	0.001935	0.006472
TMEM114	-0.15873	0.001938	0.006481
BAT2L1	-0.15872	0.001939	0.006483
GOLGA8D	-0.1587	0.001941	0.006488
SYP	-0.15868	0.001944	0.006494
ADARB1	-0.15867	0.001945	0.006496
GABARAPI	-0.15865	0.001948	0.006503
OIT3	-0.15857	0.001958	0.006536
PKD1L2	-0.15856	0.00196	0.006542
CCDC84	-0.15854	0.001962	0.006545
TGFB1	-0.1585	0.001968	0.006561
STK24	-0.1585	0.001968	0.006562
LOC22171	-0.15847	0.001972	0.006571
PREPL	-0.15845	0.001974	0.006579
ZBTB33	-0.15843	0.001977	0.006584

SPOCK3	-0.15842	0.001978	0.006587
VIPR2	-0.15842	0.001979	0.00659
ZNF785	-0.15841	0.00198	0.006592
MRC2	-0.1584	0.001981	0.006594
TNNC1	-0.1584	0.001981	0.006594
LIPI	-0.15839	0.001982	0.006595
DHDPSL	-0.15838	0.001984	0.0066
ZNF846	-0.15837	0.001985	0.006603
ZNF540	-0.1583	0.001994	0.00663
ANKRD35	-0.1583	0.001994	0.00663
PTGIR	-0.15829	0.001996	0.006635
EPM2A	-0.15822	0.002005	0.006658
LATS1	-0.15822	0.002005	0.006658
L3MBTL3	-0.15819	0.002009	0.006671
QSOX2	-0.15813	0.002017	0.006695
NCOR2	-0.15812	0.002019	0.0067
SYT1	-0.15811	0.00202	0.006701
ZNF773	-0.15808	0.002024	0.006713
USP21	-0.15806	0.002026	0.006719
PAIP1	-0.15803	0.00203	0.006726
PPAPDC3	-0.15794	0.002042	0.006761
PARD6G	-0.15789	0.002049	0.00678
ARSG	-0.15782	0.002059	0.006806
H2BFM	-0.15782	0.002059	0.006806
NUFIP2	-0.15782	0.00206	0.006806
C16orf86	-0.15776	0.002067	0.00683
GJA5	-0.15773	0.002071	0.00684
RBL2	-0.15769	0.002076	0.006857
USP49	-0.15765	0.002082	0.006872
ULBP1	-0.15765	0.002082	0.006872
ZNF404	-0.15763	0.002085	0.00688
KAZ	-0.15763	0.002086	0.006881
FLJ45340	-0.15761	0.002088	0.006886
FBLL1	-0.15761	0.002088	0.006886
CYP11A1	-0.1576	0.00209	0.006891
ST7OT4	-0.15754	0.002097	0.006912
FSTL1	-0.15751	0.002101	0.006925
C15orf59	-0.15751	0.002102	0.006926
ITPR2	-0.15748	0.002105	0.006934
OPRK1	-0.15748	0.002106	0.006935
ZNF844	-0.15747	0.002107	0.006937
WDR27	-0.15745	0.00211	0.006947
EFR3A	-0.15743	0.002113	0.006956
ZNF643	-0.15741	0.002116	0.006964
BACE1	-0.1574	0.002117	0.006967
GPR172A	-0.15739	0.002119	0.00697
LOXHD1	-0.15737	0.002121	0.006978
SLC46A2	-0.15736	0.002123	0.006982
CHST14	-0.15734	0.002125	0.006986
LRRC10B	-0.15733	0.002127	0.006988
PPP1R12A	-0.15732	0.002129	0.006994
NAALAD2	-0.15731	0.002129	0.006995
GNL1	-0.15731	0.00213	0.006995
IMPG1	-0.1573	0.002131	0.006995
CHCHD7	-0.1573	0.002131	0.006995
GALNTL4	-0.15725	0.002137	0.007013
RUNDC2C	-0.15723	0.002141	0.007023
PYGO1	-0.15712	0.002157	0.00707

WIT1	-0.15708	0.002162	0.007083
MSTN	-0.15708	0.002163	0.007083
DHX38	-0.15704	0.002167	0.007095
SFTPD	-0.15704	0.002169	0.007098
TM6SF2	-0.15701	0.002172	0.007109
BICC1	-0.15696	0.002179	0.007127
PIK3CA	-0.15696	0.00218	0.00713
ICK	-0.15695	0.002181	0.007132
SH3TC2	-0.15695	0.002182	0.007132
PNCK	-0.15691	0.002187	0.007148
KIF7	-0.15689	0.002189	0.007153
PION	-0.15689	0.00219	0.007155
USP36	-0.15688	0.002191	0.007158
PCBP4	-0.15681	0.002201	0.007187
AP3M2	-0.15674	0.002212	0.007221
DIP2C	-0.15665	0.002225	0.007263
PLAC9	-0.15663	0.002228	0.007272
ELAVL3	-0.15662	0.002229	0.007274
SCARNA5	-0.15657	0.002236	0.007297
LOC40112	-0.15653	0.002242	0.007313
CCNJ	-0.15653	0.002243	0.007315
GRM2	-0.15652	0.002244	0.007316
LOC28385	-0.15651	0.002246	0.007321
GNRHR	-0.15644	0.002255	0.007349
WASH3P	-0.15643	0.002257	0.007355
SFRS16	-0.15642	0.002258	0.007358
ZNF542	-0.1564	0.002262	0.007369
CBX2	-0.15639	0.002263	0.007369
ABHD12	-0.15637	0.002266	0.007378
C2orf63	-0.15636	0.002268	0.007382
GABARAPI	-0.15635	0.002269	0.007386
DLC1	-0.15634	0.002271	0.007389
AHSA2	-0.15633	0.002273	0.007394
PCDHA11	-0.15628	0.002279	0.007413
FLJ39653	-0.15626	0.002283	0.007425
C11orf88	-0.15625	0.002283	0.007425
MKL2	-0.15622	0.002289	0.007439
CELSR2	-0.15621	0.002291	0.007443
BCL7A	-0.1562	0.002291	0.007443
C4orf49	-0.15619	0.002294	0.007451
CABYR	-0.15615	0.002299	0.007468
ATRNL1	-0.15614	0.0023	0.007469
ZNF33A	-0.15614	0.002301	0.007471
XYLT1	-0.15613	0.002302	0.007474
HTRA1	-0.15613	0.002303	0.007474
IGF2AS	-0.1561	0.002306	0.007483
LOC20278	-0.15607	0.002311	0.007498
BTBD19	-0.15602	0.002318	0.00752
PTGER3	-0.15598	0.002324	0.007537
LHX6	-0.15595	0.002329	0.007551
PABPC4	-0.15589	0.002339	0.00758
ZNF141	-0.15587	0.002342	0.007588
CRB1	-0.15584	0.002346	0.0076
SCD5	-0.15579	0.002354	0.007623
DIRAS1	-0.15578	0.002356	0.007627
C10orf55	-0.15578	0.002356	0.007627
RHBDF1	-0.15576	0.002359	0.007635
ANKLE2	-0.15575	0.002361	0.007639

VSX1	-0.15573	0.002363	0.007643
TMPRSS5	-0.15573	0.002364	0.007644
PRSS42	-0.15571	0.002366	0.00765
TYW1	-0.1557	0.002368	0.007653
PCDHA9	-0.15566	0.002374	0.007673
FAM20B	-0.15563	0.002379	0.007683
AP4M1	-0.15562	0.00238	0.007686
POU3F1	-0.15559	0.002385	0.0077
ZC3H11A	-0.15558	0.002386	0.007701
LOC40138	-0.15556	0.00239	0.007709
KCNJ9	-0.15555	0.002392	0.007714
TUBB3	-0.15552	0.002396	0.007726
POU2F1	-0.15546	0.002406	0.007757
PCDHB11	-0.15542	0.002412	0.007773
LOC72839	-0.15536	0.002421	0.007798
WBSCR17	-0.15533	0.002427	0.007813
LPPR2	-0.15527	0.002435	0.00784
PRPF40A	-0.15527	0.002436	0.00784
FGD1	-0.15526	0.002437	0.007841
ATP8A2	-0.15522	0.002444	0.007862
KRI1	-0.1552	0.002447	0.007867
COLEC11	-0.1552	0.002448	0.00787
LOC25516	-0.15514	0.002457	0.007895
KIAA1958	-0.15514	0.002457	0.007895
ZNF75D	-0.15512	0.00246	0.007901
RALGDS	-0.1551	0.002463	0.007911
CCDC91	-0.15507	0.002469	0.007927
CERCAM	-0.15504	0.002473	0.00794
BMS1P4	-0.15503	0.002475	0.007944
KCTD20	-0.155	0.00248	0.007956
CGB	-0.15495	0.002487	0.007974
TARBP1	-0.15488	0.002498	0.00801
INPP5E	-0.15484	0.002506	0.008027
TTC21B	-0.15479	0.002513	0.008049
EHD2	-0.15479	0.002513	0.00805
NCRNA00	-0.15477	0.002516	0.008057
ZNF521	-0.15476	0.002518	0.008061
SEMA4F	-0.15475	0.002519	0.008065
S1PR5	-0.15474	0.002521	0.008067
KIAA0947	-0.15473	0.002524	0.008076
PTPRS	-0.15472	0.002525	0.008078
COL6A2	-0.15471	0.002527	0.008083
GDNF	-0.1547	0.002528	0.008086
SNCA	-0.15469	0.00253	0.008089
MAPKAP1	-0.15466	0.002534	0.0081
USP7	-0.15457	0.00255	0.008148
ZNF574	-0.15455	0.002552	0.008153
C6orf25	-0.15455	0.002554	0.008156
POLR2C	-0.15454	0.002554	0.008157
BBS1	-0.15454	0.002555	0.008158
DDIT3	-0.15452	0.002558	0.008163
PCDHGA8	-0.15452	0.002558	0.008163
EVPL	-0.15451	0.002559	0.008165
ACSS3	-0.15451	0.00256	0.008167
PPEF1	-0.15451	0.00256	0.008167
ZIC4	-0.15445	0.002571	0.008195
FAM198B	-0.15441	0.002577	0.008213
GOLGA8A	-0.15439	0.002579	0.008218

GLI4	-0.1543	0.002595	0.008264
KISS1	-0.15428	0.002599	0.008275
SERPINH1	-0.15425	0.002603	0.008288
TSPAN10	-0.1542	0.002611	0.008309
ZNF286B	-0.15419	0.002613	0.008314
TNFAIP6	-0.15419	0.002614	0.008315
KRT79	-0.15418	0.002615	0.008315
ACRC	-0.15415	0.002621	0.008328
MOXD1	-0.15403	0.002641	0.008389
BAT2	-0.15403	0.002642	0.008392
TSPAN9	-0.154	0.002646	0.008403
TTC18	-0.154	0.002647	0.008404
LIX1L	-0.15392	0.00266	0.008439
PRRT4	-0.15389	0.002665	0.008452
ABCC4	-0.15382	0.002678	0.008486
TLE2	-0.1538	0.002681	0.008497
FAM186B	-0.15378	0.002685	0.008506
WBP2NL	-0.15373	0.002692	0.008527
MAP4K5	-0.15371	0.002696	0.008537
ITGB3	-0.15369	0.0027	0.008548
ZNF391	-0.15369	0.0027	0.008548
ZNF304	-0.15368	0.002703	0.008552
RAB3GAP2	-0.1536	0.002716	0.008589
GABRR2	-0.15356	0.002723	0.008607
PGS1	-0.15355	0.002725	0.008614
ZCCHC5	-0.15354	0.002726	0.008616
CDK14	-0.15352	0.00273	0.008626
CFDP1	-0.1535	0.002734	0.008638
SUSD3	-0.15347	0.002739	0.00865
C11orf73	-0.15344	0.002744	0.008661
LOC10028	-0.15343	0.002745	0.008665
LOC13446	-0.1534	0.002752	0.008682
GSS	-0.15337	0.002756	0.00869
HEY1	-0.15337	0.002756	0.00869
OSBPL6	-0.15334	0.002761	0.008706
C6orf217	-0.15328	0.002773	0.008737
CASKIN2	-0.15328	0.002773	0.008737
C8orf45	-0.15327	0.002775	0.008741
TRPC4	-0.15321	0.002785	0.008768
CALU	-0.1532	0.002787	0.008772
UXS1	-0.15316	0.002795	0.008792
NCOA2	-0.15313	0.0028	0.008804
LOC10024	-0.15311	0.002803	0.008813
PXDN	-0.15311	0.002803	0.008813
CEACAM1	-0.15308	0.002808	0.008828
SNCB	-0.15305	0.002814	0.008844
LUC7L3	-0.15298	0.002827	0.008882
COL5A1	-0.15295	0.002833	0.0089
SNAI2	-0.1529	0.002842	0.008924
ZBTB40	-0.15285	0.002852	0.008949
SNX13	-0.15283	0.002854	0.008956
CAV2	-0.15279	0.002861	0.008977
SAMD10	-0.15276	0.002866	0.00899
AARS	-0.15276	0.002867	0.008991
ALS2	-0.15272	0.002875	0.009015
SRPK3	-0.15267	0.002884	0.009037
ANKS3	-0.15265	0.002888	0.009047
KRT32	-0.15264	0.00289	0.009054

CNTN6	-0.15259	0.002899	0.009078
NLGN1	-0.1525	0.002917	0.009127
SLC12A6	-0.15245	0.002926	0.00915
FAM133A	-0.15242	0.002931	0.009164
PAR-SN	-0.1524	0.002935	0.009174
NLGN2	-0.15234	0.002947	0.009206
MAGED4B	-0.15231	0.002951	0.009219
MAPK8IP3	-0.1523	0.002953	0.009224
C6orf168	-0.1523	0.002954	0.009225
ADAM23	-0.15226	0.002962	0.009249
LRRC39	-0.15225	0.002964	0.009252
RNF113B	-0.15219	0.002974	0.00928
FOXL1	-0.15219	0.002974	0.00928
C5orf53	-0.15219	0.002974	0.00928
C1orf54	-0.15216	0.00298	0.009295
CTNNB1	-0.15216	0.00298	0.009295
CDK5R2	-0.15211	0.00299	0.009321
SYCE1L	-0.15208	0.002996	0.009339
ZNF98	-0.15204	0.003003	0.009357
ZNF551	-0.15199	0.003013	0.009387
FBXL20	-0.15194	0.003022	0.009413
TCEAL7	-0.15193	0.003026	0.009422
NXPH1	-0.15185	0.003041	0.009468
BBS2	-0.15183	0.003044	0.009475
KNDC1	-0.15183	0.003045	0.009475
C10orf72	-0.15181	0.003048	0.009483
GUSBP3	-0.15181	0.003048	0.009483
ELN	-0.15178	0.003053	0.009493
TBCB	-0.15175	0.003059	0.00951
TMEM204	-0.15175	0.00306	0.009512
LOC72802	-0.15172	0.003065	0.009522
ZNF546	-0.15166	0.003077	0.009557
TMEM63A	-0.15164	0.003082	0.009571
NAA30	-0.1516	0.00309	0.009593
C10orf114	-0.15159	0.003091	0.009595
DLEU2L	-0.15159	0.003091	0.009596
PHKA2	-0.15157	0.003096	0.009607
ODF3L2	-0.15156	0.003098	0.009611
LGI2	-0.15154	0.003102	0.009621
PRPF4B	-0.15152	0.003105	0.00963
SCAI	-0.15147	0.003116	0.009661
WNT9B	-0.15145	0.003118	0.009665
PTPN4	-0.15142	0.003126	0.009686
SLC10A4	-0.15141	0.003127	0.009686
ZNF143	-0.15141	0.003127	0.009686
TM9SF2	-0.1514	0.003129	0.00969
WWP1	-0.1514	0.00313	0.009691
ADCY3	-0.15134	0.003141	0.009722
RAB30	-0.15133	0.003143	0.009726
TFAP4	-0.15132	0.003145	0.00973
CTNNA2	-0.15128	0.003154	0.009754
EIF2B4	-0.15124	0.00316	0.009771
GPR173	-0.15124	0.003161	0.009772
C4orf22	-0.15123	0.003164	0.009779
KLRG2	-0.15117	0.003176	0.009815
POLG2	-0.15112	0.003185	0.009837
TBPL1	-0.15112	0.003185	0.009837
GPR157	-0.1511	0.003189	0.009846

PRSS37	-0.1511	0.003189	0.009846
FUT7	-0.15109	0.003192	0.009852
PDGFA	-0.15107	0.003196	0.009863
FGFR1OP2	-0.15102	0.003205	0.009889
CDK17	-0.15098	0.003214	0.009911
SORL1	-0.15097	0.003216	0.009914
APBA3	-0.15092	0.003226	0.009942
NBEA	-0.15084	0.003242	0.009992
SKIL	-0.15084	0.003243	0.009993
PLD5	-0.15084	0.003244	0.009993
SKAP2	-0.15083	0.003245	0.009996
TFCP2	-0.1508	0.003251	0.010013
CEP290	-0.15078	0.003256	0.010027
ZNF268	-0.15077	0.003257	0.010028
BRWD1	-0.15076	0.003259	0.010032
FST	-0.15064	0.003284	0.010103
ANO4	-0.15061	0.003292	0.010123
SNAP91	-0.1506	0.003293	0.010124
TGFB2	-0.1506	0.003293	0.010124
TAS2R20	-0.15054	0.003306	0.010156
ZBTB44	-0.15044	0.003327	0.010216
SULT4A1	-0.15038	0.003339	0.010251
TREX2	-0.15035	0.003346	0.010272
CREB5	-0.15034	0.003349	0.010279
CCNA1	-0.1503	0.003357	0.010302
PRIC285	-0.1503	0.003357	0.010302
ZBTB16	-0.15029	0.003358	0.010303
SCAMP5	-0.15028	0.00336	0.010305
TFE3	-0.15027	0.003364	0.010312
CSRNP3	-0.15026	0.003365	0.010313
AQPEP	-0.15022	0.003374	0.010337
ZNF77	-0.15021	0.003376	0.010342
PLA1A	-0.15017	0.003384	0.010363
FLYWCH1	-0.15016	0.003386	0.010366
SERHL2	-0.15016	0.003387	0.010367
LOC15857	-0.15014	0.003391	0.010376
FAM124B	-0.15013	0.003392	0.010377
DPT	-0.15012	0.003396	0.010387
C4orf37	-0.15012	0.003396	0.010387
LOC57255	-0.1501	0.0034	0.010397
WASH7P	-0.15003	0.003415	0.010441
BAGE2	-0.15001	0.00342	0.010453
MMP8	-0.14999	0.003423	0.010461
ARRB2	-0.14998	0.003425	0.010461
OAZ2	-0.14998	0.003425	0.010461
C17orf67	-0.14998	0.003427	0.010464
ELOVL2	-0.14995	0.003431	0.010475
EGFL8	-0.1499	0.003443	0.010508
POT1	-0.14988	0.003448	0.010523
NUDT3	-0.14985	0.003455	0.01054
CLEC2L	-0.14984	0.003457	0.010546
MSL1	-0.14982	0.00346	0.010554
FBXL22	-0.1498	0.003465	0.010566
SPATA4	-0.14974	0.003478	0.010603
ATF7IP	-0.14972	0.003482	0.010611
SUV420H2	-0.14968	0.00349	0.010634
GDPD4	-0.14968	0.003491	0.010634
ZNF780B	-0.14968	0.003491	0.010634

LDB2	-0.14967	0.003493	0.010635
TRMT11	-0.14967	0.003493	0.010635
HPCAL4	-0.14966	0.003497	0.010643
TSGA10	-0.14961	0.003506	0.010668
DCX	-0.1496	0.003508	0.010672
TBXA2R	-0.14955	0.00352	0.010706
OR13J1	-0.14955	0.003521	0.010706
ELAVL2	-0.14954	0.003523	0.010709
CXXC5	-0.14954	0.003523	0.010709
PCDHGA1	-0.14953	0.003524	0.010709
FER1L4	-0.14953	0.003526	0.010712
DIRC1	-0.14953	0.003526	0.010712
NCRNA00	-0.14952	0.003527	0.010714
SNAPC5	-0.14951	0.00353	0.01072
HEATR7A	-0.14949	0.003533	0.01073
DDHD1	-0.14948	0.003535	0.010733
CRABP1	-0.14946	0.00354	0.010747
KIAA0907	-0.14945	0.003543	0.010754
LOC10013	-0.14944	0.003545	0.010759
ARHGAP1(	-0.14942	0.003549	0.010768
CNIH2	-0.14941	0.003553	0.010777
LOC28503	-0.1494	0.003553	0.010777
MAN2C1	-0.14939	0.003556	0.010784
KCNIP2	-0.14939	0.003557	0.010785
TDRD9	-0.14938	0.003558	0.010788
GRIP1	-0.14935	0.003566	0.010807
RPL21P44	-0.14932	0.003573	0.010826
TMEM200	-0.1493	0.003577	0.010837
EBF4	-0.14924	0.00359	0.010876
ZNF417	-0.14923	0.003593	0.010883
CCL16	-0.14919	0.003602	0.010909
LRP2	-0.14916	0.003607	0.010923
SLC24A1	-0.1491	0.003621	0.010963
HDAC6	-0.14908	0.003626	0.010976
FAM5B	-0.14907	0.003629	0.010982
COL1A2	-0.14903	0.003637	0.011001
LOC15117	-0.14894	0.003658	0.01106
NAPSA	-0.14889	0.003671	0.011091
SYT6	-0.14884	0.003682	0.011116
CYBRD1	-0.14881	0.003689	0.011134
NEK10	-0.14879	0.003693	0.011145
LRRC3B	-0.14878	0.003696	0.011152
PRR24	-0.14874	0.003706	0.011178
SERPINF1	-0.14871	0.003713	0.011198
ZNF764	-0.1487	0.003713	0.011198
ANGPT1	-0.14869	0.003716	0.011204
TRIO	-0.14863	0.00373	0.011243
GPX7	-0.1486	0.003737	0.01126
BCAT1	-0.14859	0.00374	0.01127
MDM4	-0.14853	0.003754	0.011304
BCAP31	-0.14852	0.003756	0.011307
POU6F1	-0.14849	0.003763	0.011326
CHN1	-0.14846	0.00377	0.011342
ODZ1	-0.14842	0.003781	0.011375
CREG1	-0.14834	0.003799	0.011426
KCND1	-0.14834	0.0038	0.01143
TMEM182	-0.1483	0.003808	0.01145
STC1	-0.14824	0.003824	0.011497

FLJ26850	-0.14822	0.003828	0.011508
RSPH10B2	-0.14818	0.003838	0.011533
ZNF682	-0.14816	0.003842	0.011542
BEND5	-0.14808	0.003861	0.011593
LRRC32	-0.14808	0.003861	0.011593
KRTAP3-1	-0.14808	0.003863	0.011595
DIRAS3	-0.14808	0.003863	0.011595
LOC28490	-0.14807	0.003865	0.011599
ELK3	-0.14797	0.00389	0.011669
CHST3	-0.14793	0.003898	0.011692
PLEKHA2	-0.14792	0.003902	0.011702
ZNF776	-0.14786	0.003916	0.011743
LGALS14	-0.14783	0.003922	0.011757
FAM119A	-0.14783	0.003922	0.011757
NPR1	-0.14782	0.003925	0.011764
SPSB3	-0.1478	0.003931	0.011778
SLC29A1	-0.14778	0.003936	0.011788
SEZ6L	-0.14769	0.003958	0.011852
CPA4	-0.14765	0.003967	0.011876
LGALS8	-0.14765	0.003968	0.011879
FRY	-0.14762	0.003975	0.011895
WDR33	-0.14757	0.003986	0.011925
SPDYE5	-0.14755	0.003992	0.011939
CHRAC1	-0.14753	0.003998	0.011956
MYST2	-0.14746	0.004015	0.012003
NRP1	-0.14746	0.004016	0.012004
HMP19	-0.14744	0.00402	0.012014
TCEB1	-0.14744	0.00402	0.012014
FSCB	-0.14744	0.004021	0.012014
COBL	-0.14743	0.004023	0.012017
C6orf70	-0.14743	0.004024	0.012017
C11orf66	-0.14742	0.004024	0.012017
VKORC1	-0.14742	0.004025	0.012018
SH3PXD2E	-0.14742	0.004025	0.012018
DHFRL1	-0.14737	0.004037	0.012051
PLIN4	-0.14737	0.004037	0.012051
ATP4B	-0.14736	0.004039	0.012053
ARHGAP3(	-0.14734	0.004044	0.012066
ODZ4	-0.14734	0.004044	0.012066
MRGPRF	-0.14728	0.004059	0.012108
ITPKB	-0.14728	0.00406	0.01211
SYT14	-0.14727	0.004062	0.012113
ZNF554	-0.14727	0.004064	0.012115
ATP2B3	-0.14723	0.004072	0.012136
IYD	-0.14716	0.00409	0.012185
LOC64575	-0.14711	0.004104	0.012224
TGM2	-0.14709	0.004109	0.012238
ZNF788	-0.14708	0.004111	0.012241
LOC44120	-0.14708	0.004112	0.012242
PPFIA2	-0.14706	0.004117	0.012255
C2orf50	-0.14705	0.00412	0.012262
MAPK12	-0.14704	0.00412	0.012262
NCRNA00:	-0.14699	0.004134	0.012295
DST	-0.14696	0.004142	0.012315
SNX31	-0.14696	0.004142	0.012315
GRHL2	-0.14692	0.004152	0.012342
TSC1	-0.1469	0.004158	0.012356
SOX8	-0.1468	0.004184	0.012428

FMN2	-0.14679	0.004186	0.012431
PRB3	-0.14679	0.004187	0.012431
SUZ12P	-0.14678	0.004188	0.012433
C14orf128	-0.14678	0.00419	0.012436
DYSF	-0.14676	0.004193	0.012445
C1orf51	-0.14674	0.0042	0.01246
ACSS1	-0.14673	0.004201	0.012462
FAM83C	-0.14672	0.004204	0.012462
WNT7A	-0.14672	0.004204	0.012462
C21orf91	-0.14672	0.004205	0.012462
CBLN4	-0.14666	0.004221	0.012506
LOC10012	-0.14663	0.004228	0.012522
SLC13A4	-0.14663	0.004229	0.012525
C1orf127	-0.14661	0.004234	0.012538
CEP110	-0.14659	0.004238	0.012547
SHROOM2	-0.14657	0.004245	0.012563
PRAMEF14	-0.14655	0.004248	0.012571
UHRF2	-0.14653	0.004255	0.012588
RIC3	-0.14652	0.004258	0.012594
GPM6B	-0.1465	0.004263	0.012608
MSX2P1	-0.14649	0.004264	0.012609
B3GALTL	-0.14648	0.004267	0.012612
TREM2	-0.14648	0.004267	0.012612
TNR	-0.14648	0.004267	0.012612
LIMCH1	-0.14641	0.004287	0.012665
ERO1LB	-0.14638	0.004294	0.012686
RBM9	-0.14634	0.004304	0.012712
DLEU2	-0.14632	0.004309	0.012724
MMRN2	-0.14632	0.00431	0.012724
MLH3	-0.14625	0.004329	0.012774
C8orf37	-0.14615	0.004357	0.012849
CCDC108	-0.14606	0.004381	0.01291
ZNF573	-0.14605	0.004382	0.012913
ZNF845	-0.14604	0.004386	0.012922
TRPV4	-0.14602	0.004391	0.012933
FAM124A	-0.14596	0.004409	0.01298
KLC1	-0.14592	0.004418	0.013001
OPCML	-0.14587	0.004433	0.01304
LRRC15	-0.14586	0.004434	0.013043
ZNF429	-0.14584	0.004439	0.013053
C14orf135	-0.14583	0.004444	0.013065
KANK1	-0.14583	0.004444	0.013065
ZNF202	-0.14581	0.004448	0.013073
GLCCI1	-0.1458	0.004453	0.013084
GAS5	-0.14579	0.004454	0.013086
CST9	-0.14575	0.004464	0.01311
KCND2	-0.14566	0.004491	0.013182
TMEM17	-0.14564	0.004497	0.013198
MMD	-0.14559	0.00451	0.013231
ADAM12	-0.14559	0.00451	0.013231
MFF	-0.14557	0.004517	0.01325
FAM19A2	-0.14556	0.004518	0.013252
MST1	-0.1455	0.004534	0.013291
DKK2	-0.14549	0.004537	0.013297
MLL2	-0.14547	0.004543	0.013311
KRT74	-0.14546	0.004545	0.013313
USP20	-0.14543	0.004556	0.013343
MGAT5B	-0.14541	0.00456	0.013353

EDAR	-0.14541	0.004561	0.013353
LOC10028	-0.1454	0.004562	0.013355
C17orf102	-0.14538	0.004569	0.01337
PLA2G12B	-0.14535	0.004577	0.013392
NCRNA00	-0.14532	0.004586	0.013417
TAF3	-0.14525	0.004605	0.013458
ARAP3	-0.14525	0.004605	0.013458
EEF1G	-0.14522	0.004613	0.013477
APOC1	-0.1452	0.004621	0.0135
EYA4	-0.14516	0.00463	0.013524
KIFAP3	-0.14515	0.004635	0.013537
PDE1B	-0.14508	0.004654	0.013586
BAT3	-0.14506	0.004659	0.013594
GGN	-0.14501	0.004673	0.013634
RRN3P2	-0.145	0.004676	0.013637
TMEM22	-0.14499	0.004681	0.013648
ZNF815	-0.14498	0.004683	0.013654
VWCE	-0.14495	0.004691	0.013672
LOC28583	-0.14494	0.004693	0.013676
IRAK4	-0.14493	0.004696	0.013682
TWIST2	-0.14492	0.0047	0.013691
SEMA3G	-0.14491	0.004704	0.013699
ZNF33B	-0.14489	0.004708	0.013707
IKBKB	-0.14488	0.004712	0.013717
ZNF222	-0.14488	0.004713	0.013717
FBXO41	-0.14486	0.004717	0.013727
WASF1	-0.14485	0.004719	0.013732
MAP7D1	-0.14485	0.00472	0.013732
C8orf48	-0.14485	0.004721	0.013735
UBXN2A	-0.14483	0.004726	0.013745
RAB3GAP1	-0.14482	0.004729	0.013751
RPL23AP6	-0.1448	0.004734	0.013764
CCDC11	-0.14479	0.004738	0.013776
ANKS1A	-0.14471	0.004762	0.013839
SERPINF2	-0.1447	0.004765	0.013844
C12orf54	-0.14468	0.00477	0.013857
FEZ1	-0.14466	0.004777	0.013873
BIRC6	-0.14465	0.004778	0.013874
CEP120	-0.14465	0.004779	0.013877
C7orf27	-0.1446	0.004794	0.013916
POLR3E	-0.14459	0.004796	0.013923
MCTP1	-0.14456	0.004806	0.013947
CES7	-0.14454	0.00481	0.013958
CHRND	-0.14451	0.004821	0.013984
HNF1A	-0.14445	0.004838	0.014031
LTBP2	-0.1444	0.004853	0.014069
TNXB	-0.14439	0.004855	0.014073
ZNF397OS	-0.14438	0.004857	0.014079
UPK2	-0.14434	0.00487	0.014109
SMARCAD	-0.14431	0.004878	0.014124
LRRC58	-0.1443	0.004884	0.014139
RPSAP52	-0.14429	0.004886	0.014142
HECW2	-0.14428	0.004889	0.01415
SAPS3	-0.14427	0.00489	0.014151
XPOT	-0.14427	0.004891	0.014153
FBXO3	-0.14426	0.004894	0.014157
ST8SIA3	-0.1442	0.004911	0.014201
RGS7BP	-0.14418	0.004917	0.014217

IMMP2L	-0.14418	0.004919	0.014221
FOXN2	-0.14409	0.004944	0.014283
SLITRK3	-0.14409	0.004947	0.014289
DEFB104A	-0.14407	0.004951	0.014295
ERBB4	-0.14407	0.004952	0.014295
GAMT	-0.14404	0.004961	0.014313
GPR182	-0.14403	0.004963	0.014318
NR2F1	-0.14401	0.004971	0.014335
PARN	-0.14399	0.004978	0.014349
CNOT3	-0.14395	0.004988	0.014375
LOC34001	-0.14394	0.00499	0.01438
CST6	-0.14393	0.004993	0.014386
PRKG2	-0.14388	0.005009	0.01443
TSNAXIP1	-0.14383	0.005024	0.014465
ERCC1	-0.14375	0.00505	0.014534
KCNK4	-0.1437	0.005065	0.014573
PRTG	-0.14369	0.005069	0.014585
FANCF	-0.14366	0.005076	0.014596
INS-IGF2	-0.14366	0.005076	0.014596
SIAH2	-0.14362	0.005088	0.014629
GPR116	-0.1436	0.005096	0.014648
SLC22A2	-0.14359	0.005099	0.014654
C20orf12	-0.14358	0.005101	0.014657
PABPC1P2	-0.14357	0.005104	0.014663
CLK2P	-0.14355	0.005112	0.014682
SLC2A9	-0.14352	0.005121	0.014704
C3orf35	-0.14348	0.005133	0.014731
TAF1B	-0.14346	0.005141	0.01475
BPTF	-0.14345	0.005144	0.014755
GPX3	-0.14344	0.005145	0.014757
LRRC36	-0.14343	0.00515	0.014769
SCN2A	-0.14342	0.005152	0.014772
USP12	-0.14339	0.005163	0.014801
SART1	-0.14335	0.005175	0.014835
CNRIP1	-0.14331	0.005187	0.014864
RAP2A	-0.1433	0.005191	0.014875
KCNH5	-0.14328	0.005196	0.014887
SFRS13A	-0.14328	0.005198	0.014888
SAMD11	-0.14322	0.005217	0.014942
FGF7	-0.14316	0.005234	0.014984
COPS5	-0.14316	0.005236	0.014989
S100A2	-0.14307	0.005265	0.015062
PCDH7	-0.14306	0.005266	0.015063
CCNB1IP1	-0.14305	0.005269	0.015069
APBB2	-0.143	0.005286	0.015114
FAM113B	-0.14296	0.005298	0.015144
TEKT3	-0.14295	0.005302	0.015155
RCN1	-0.14294	0.005306	0.015164
C1QL1	-0.14291	0.005317	0.015188
CYP7A1	-0.14285	0.005335	0.015239
LOC12195	-0.14281	0.005349	0.015273
ZNF385B	-0.1428	0.00535	0.015273
TOP1P2	-0.1428	0.005351	0.015273
ZNF569	-0.1428	0.005351	0.015273
SMPD4	-0.14279	0.005354	0.01528
CLUAP1	-0.14276	0.005362	0.0153
LOC61920	-0.14272	0.005378	0.015342
C1orf216	-0.14268	0.005391	0.015376

KIAA1984	-0.14263	0.005406	0.015415
SERPINE1	-0.14262	0.00541	0.015425
CYP27C1	-0.1426	0.005417	0.015443
HGF	-0.14259	0.005418	0.015445
SULF2	-0.14258	0.005421	0.015451
CD99L2	-0.14257	0.005426	0.015463
CHSY1	-0.14253	0.005438	0.015494
TTC3	-0.14251	0.005444	0.01551
COLEC12	-0.14249	0.005452	0.015529
ISY1	-0.14248	0.005457	0.015539
TM2D3	-0.14238	0.005489	0.015626
GPAM	-0.14237	0.005492	0.01563
SLC26A11	-0.14236	0.005495	0.015636
POLN	-0.14233	0.005507	0.015664
FSTL4	-0.14231	0.005511	0.015674
FAM131C	-0.1423	0.005515	0.015682
C1orf107	-0.1423	0.005516	0.015682
SLC38A8	-0.1423	0.005517	0.015682
LUM	-0.14224	0.005535	0.015726
PCDHGA9	-0.14224	0.005535	0.015726
CUTC	-0.14222	0.005541	0.01574
EPHB6	-0.14214	0.005568	0.01581
C2orf28	-0.14211	0.00558	0.015839
TMEM105	-0.1421	0.005581	0.015839
DKC1	-0.1421	0.005583	0.015841
CLGN	-0.1421	0.005584	0.015842
DBX2	-0.14208	0.005589	0.015849
GTF2A1L	-0.14206	0.005595	0.01586
DYRK3	-0.14206	0.005596	0.015861
RNF19A	-0.14205	0.005599	0.015864
ANGPTL2	-0.14205	0.0056	0.015864
ZNF334	-0.14203	0.005608	0.015881
C7orf58	-0.14202	0.005611	0.015886
MAB21L2	-0.14199	0.005619	0.015903
AGAP11	-0.14198	0.005625	0.015916
TPPP3	-0.14196	0.005632	0.015932
ZNF354C	-0.14194	0.005637	0.015941
FABP4	-0.14184	0.00567	0.01603
UACA	-0.14184	0.005671	0.016032
MSR1	-0.14178	0.005691	0.016082
SLC25A42	-0.14177	0.005697	0.016096
SALL3	-0.14175	0.005703	0.016109
LGALS1	-0.14172	0.005713	0.016132
EXTL2	-0.14171	0.005716	0.016134
C16orf45	-0.14169	0.005724	0.016153
C13orf31	-0.14167	0.00573	0.016169
C10orf82	-0.14166	0.005735	0.016179
ARHGAP6	-0.14165	0.005736	0.01618
TBC1D25	-0.14163	0.005744	0.016201
C1orf88	-0.14161	0.005752	0.016218
EPM2AIP1	-0.1416	0.005753	0.016218
FCGR1B	-0.14153	0.005778	0.01628
GRINL1A	-0.14153	0.00578	0.016282
ZNF605	-0.14152	0.00578	0.016282
SLC16A11	-0.14152	0.005783	0.016288
POLR1A	-0.1415	0.005789	0.016299
CCDC146	-0.14149	0.005794	0.016309
VSIG10L	-0.14146	0.005804	0.016334

SPON2	-0.14143	0.005815	0.016358
THPO	-0.14142	0.005816	0.016359
NT5M	-0.14142	0.005817	0.01636
PCOLCE2	-0.14137	0.005834	0.016402
TCF4	-0.14132	0.005852	0.016446
CRIP2	-0.1413	0.005859	0.016463
GPC6	-0.14128	0.005866	0.01648
ZSCAN1	-0.14126	0.005873	0.016496
TP53BP1	-0.14125	0.005877	0.016505
ZNF277	-0.14124	0.00588	0.016512
FNIP1	-0.14123	0.005883	0.016517
BMPR1B	-0.14117	0.005906	0.016572
FAM60A	-0.14116	0.005908	0.016575
TRIM24	-0.14116	0.005909	0.016575
STRC	-0.14113	0.005919	0.016599
RND3	-0.14109	0.005935	0.016635
AMFR	-0.14104	0.00595	0.016672
NCKAP5	-0.14103	0.005954	0.016679
TJP1	-0.14103	0.005955	0.016681
MSI1	-0.14099	0.00597	0.016717
CDH13	-0.14097	0.005978	0.016737
ZDHHC4	-0.14095	0.005984	0.016752
ARL13B	-0.14094	0.005986	0.016755
SMOX	-0.14087	0.006012	0.016824
USP44	-0.14086	0.006017	0.016835
GPR19	-0.14082	0.006031	0.01687
RGPD1	-0.14081	0.006033	0.016874
PMP22	-0.14081	0.006034	0.016874
FES	-0.14081	0.006035	0.016874
CRTAP	-0.14077	0.006048	0.016904
CSN2	-0.14077	0.006049	0.016904
FSTL5	-0.14077	0.00605	0.016906
GOLGA6L1	-0.14074	0.006061	0.016932
WIPF3	-0.14068	0.006081	0.016984
RABGEF1	-0.14068	0.006082	0.016984
TBC1D13	-0.14067	0.006084	0.016988
C11orf46	-0.14067	0.006085	0.016988
LRRN4	-0.14062	0.006103	0.017038
PKN1	-0.14061	0.006105	0.017041
RBMS2	-0.14059	0.006116	0.017066
SUPT6H	-0.14049	0.006153	0.017164
MTSS1L	-0.14038	0.006194	0.01727
WDYHV1	-0.14034	0.006207	0.017303
TSC22D2	-0.14034	0.006208	0.017303
MYO16	-0.14034	0.006208	0.017303
NDN	-0.14033	0.006212	0.017311
ILF3	-0.14032	0.006214	0.017313
TUBA8	-0.14025	0.00624	0.017383
DNAJC14	-0.14021	0.006254	0.017416
ZNF792	-0.14017	0.006269	0.017454
TCP10	-0.14017	0.006271	0.017455
MAP1D	-0.14014	0.006281	0.017481
HEATR1	-0.14012	0.00629	0.0175
RPL31	-0.14008	0.006304	0.017536
KRT38	-0.14007	0.006306	0.01754
G6PC	-0.14005	0.006315	0.017561
APOB	-0.14005	0.006316	0.017562
SV2C	-0.14002	0.006325	0.017582

TRIM6	-0.14002	0.006327	0.017584
PTPN11	-0.14002	0.006327	0.017584
HSP90AB1	-0.14001	0.006329	0.017587
COL1A1	-0.14	0.006334	0.017598
SNX32	-0.13997	0.006346	0.01762
KIAA1755	-0.13996	0.00635	0.017628
ZNF444	-0.13996	0.006351	0.01763
C3orf27	-0.13994	0.006356	0.017641
TMEM68	-0.13988	0.00638	0.017702
PCGF3	-0.13985	0.006391	0.017729
C16orf87	-0.13984	0.006393	0.017734
NRXN1	-0.13984	0.006394	0.017735
SFRP1	-0.13981	0.006406	0.017766
DOCK4	-0.13981	0.006408	0.017767
ZNF717	-0.1398	0.006409	0.017767
MMP23B	-0.13979	0.006413	0.017777
IFNA10	-0.13975	0.00643	0.017816
SPDYE1	-0.13975	0.006431	0.017816
HSPA1B	-0.13972	0.006442	0.017841
C8orf56	-0.13971	0.006443	0.017842
MGC1612[illegible]	-0.13971	0.006446	0.017847
TRH	-0.13965	0.006466	0.017899
ZNF354B	-0.13955	0.006508	0.018004
FBRSL1	-0.13952	0.006517	0.018026
GRB10	-0.13952	0.00652	0.018029
CGB7	-0.13951	0.006522	0.018033
HIGD2B	-0.1395	0.006526	0.018041
C17orf51	-0.13941	0.006561	0.01813
ZFAND3	-0.1394	0.006565	0.018137
ARMC9	-0.13938	0.006572	0.018153
KCNH4	-0.13935	0.006585	0.018184
KRT13	-0.13935	0.006585	0.018184
ZFP90	-0.13929	0.006607	0.018234
UBXN2B	-0.13927	0.006616	0.018253
ITGAV	-0.13922	0.006636	0.0183
BBOX1	-0.13921	0.006638	0.018303
CARS2	-0.13916	0.006659	0.018356
LOC93432	-0.13913	0.006673	0.01839
NOL8	-0.13911	0.00668	0.018402
RBM44	-0.13911	0.00668	0.018402
SLC5A12	-0.1391	0.006683	0.018407
ESPNL	-0.1391	0.006684	0.018408
TMSB15B	-0.13909	0.006686	0.01841
COL12A1	-0.13908	0.006693	0.018426
LOC28545	-0.13903	0.006712	0.018475
IGSF21	-0.13901	0.006721	0.018497
STC2	-0.139	0.006722	0.018499
LOC44094	-0.13895	0.006742	0.018548
ATP6AP1	-0.13894	0.006748	0.018563
PRCP	-0.13893	0.006753	0.018571
NYNRIN	-0.13889	0.006766	0.018599
DNHD1	-0.13885	0.006784	0.018644
ZNF138	-0.13884	0.006788	0.018649
FAM180A	-0.13879	0.006807	0.018697
NAALADL[illegible]	-0.13873	0.006832	0.018763
FAM164A	-0.13869	0.006849	0.018805
TTYH3	-0.13868	0.006851	0.018808
ACTR3C	-0.13867	0.006855	0.018815

DSEL	-0.13866	0.006858	0.01882
B4GALT5	-0.13865	0.006863	0.018831
LTBP1	-0.13861	0.006881	0.018872
TCTEX1D1	-0.13856	0.006901	0.018924
CBX3	-0.13855	0.006905	0.018932
KIAA0146	-0.13855	0.006906	0.018932
BPI	-0.1385	0.006927	0.018987
PHF13	-0.13846	0.006941	0.019016
KRT36	-0.13841	0.006963	0.019072
RGR	-0.1384	0.006967	0.019079
SLC16A4	-0.1384	0.006968	0.019081
DNAJC2	-0.13835	0.006987	0.019128
MASP1	-0.13824	0.007034	0.019247
FAM19A3	-0.13823	0.007038	0.019251
ZNF691	-0.1382	0.007049	0.01927
LOC40002	-0.1382	0.00705	0.01927
GHRHR	-0.1382	0.00705	0.01927
C4orf6	-0.1382	0.007051	0.01927
PRSS35	-0.13812	0.007084	0.019347
C20orf118	-0.13809	0.007094	0.019372
SLC14A1	-0.13809	0.007095	0.019373
CCDC130	-0.13809	0.007096	0.019373
BOP1	-0.13807	0.007102	0.019386
C14orf33	-0.13805	0.007112	0.019404
BAHCC1	-0.138	0.007134	0.01946
ZNFX1	-0.13799	0.007137	0.019465
FAM82B	-0.13795	0.007155	0.019511
FZD4	-0.13792	0.007168	0.019543
LECT2	-0.13788	0.007183	0.019583
ERG	-0.13787	0.007186	0.019587
RAB11B	-0.13787	0.007189	0.019594
GRK1	-0.13785	0.007194	0.019605
SDPR	-0.13785	0.007197	0.019607
ZNF586	-0.13785	0.007197	0.019607
GIT1	-0.13785	0.007198	0.019607
DBP	-0.13783	0.007205	0.019622
EPGN	-0.13775	0.00724	0.019708
GREM1	-0.13774	0.007245	0.019718
LOC72832	-0.13769	0.007263	0.019763
PLCXD2	-0.13767	0.007272	0.019785
C17orf104	-0.13762	0.007292	0.019834
VASN	-0.13759	0.007305	0.019864
LOC44166	-0.13756	0.007318	0.019897
GNGT1	-0.13756	0.007321	0.019902
CLEC5A	-0.13755	0.007325	0.019909
ADAMTSL4	-0.13752	0.007337	0.019939
ADAMTS1	-0.13752	0.007339	0.019943
GDF1	-0.13751	0.007343	0.019949
SCGB2A2	-0.1375	0.007346	0.019951
ATP6V1E2	-0.1375	0.007347	0.019951
MYCT1	-0.13748	0.007357	0.019971
GRM5	-0.1374	0.00739	0.020052
TLN1	-0.13737	0.0074	0.020078
SHISA7	-0.13735	0.007412	0.020104
SCN4A	-0.13735	0.007413	0.020104
LOC28376	-0.13728	0.007442	0.020172
FAM134A	-0.13725	0.007454	0.020198
LOC10019	-0.13724	0.007458	0.020204

PSPH	-0.13724	0.007458	0.020204
UBE2C	-0.13717	0.00749	0.020276
USF2	-0.13716	0.007492	0.020278
ARMCX5	-0.13716	0.007496	0.020284
TBC1D9	-0.13706	0.007538	0.020386
PSMD7	-0.13706	0.007538	0.020386
TRHR	-0.137	0.007565	0.020449
CCDC158	-0.13695	0.007588	0.020507
RYR1	-0.13694	0.007592	0.020515
WASH2P	-0.13692	0.007599	0.020525
NACA2	-0.1369	0.007608	0.020543
KEL	-0.13688	0.007617	0.020562
FAM157A	-0.13688	0.00762	0.020568
FAM101B	-0.13682	0.007643	0.020624
C20orf160	-0.13681	0.007652	0.020641
TRIM78P	-0.13678	0.007664	0.020673
SOX7	-0.13677	0.007668	0.020681
C20orf46	-0.13676	0.00767	0.020684
GNAL	-0.13676	0.007674	0.020689
LOC37444	-0.13675	0.007674	0.020689
POMP	-0.13671	0.007694	0.020733
OR5AK2	-0.13669	0.007703	0.020755
UBAP2L	-0.13662	0.007733	0.020834
IMPG2	-0.1366	0.007746	0.020862
PLEKHA3	-0.13656	0.007764	0.020904
LOC64516	-0.13643	0.007822	0.02105
CLEC1B	-0.13642	0.007824	0.021052
KIAA1644	-0.13642	0.007825	0.021053
RAB23	-0.1364	0.007837	0.021077
RENBP	-0.13639	0.007838	0.021077
WDR47	-0.13636	0.007856	0.021119
BTF3L4	-0.13635	0.007859	0.021123
RBM4	-0.13628	0.007888	0.02119
CRYBA1	-0.13625	0.007905	0.021232
GABRE	-0.13624	0.007907	0.021235
RBM6	-0.13621	0.007922	0.021272
HOXA7	-0.13619	0.007932	0.021291
NEFM	-0.13618	0.007935	0.021293
ANKRD53	-0.13616	0.007948	0.021316
LAPTM4A	-0.13615	0.007948	0.021316
PIM1	-0.13615	0.007949	0.021316
SLC5A4	-0.13615	0.00795	0.021317
DCUN1D3	-0.13614	0.007955	0.021328
LOC28437	-0.13609	0.007976	0.021375
ZNF555	-0.13608	0.007982	0.021385
ZIM2	-0.13608	0.007983	0.021385
FAM196A	-0.13607	0.007986	0.02139
NID1	-0.13607	0.007987	0.02139
CPPED1	-0.13607	0.007988	0.02139
CCKAR	-0.13602	0.008011	0.021443
NR2C2	-0.13601	0.008017	0.021456
LRAT	-0.13599	0.008027	0.021478
ASCL2	-0.13598	0.008028	0.02148
DERL1	-0.13595	0.008042	0.021513
DCAF16	-0.13594	0.00805	0.021531
IFFO1	-0.13591	0.008064	0.021565
SCG3	-0.13584	0.008093	0.021638
MURC	-0.13582	0.008104	0.021666

TMEM79	-0.13582	0.008106	0.021667
FLJ45983	-0.13581	0.00811	0.021676
GPR180	-0.13577	0.008129	0.021716
MCF2	-0.13577	0.00813	0.021717
ZNF436	-0.13576	0.008135	0.021727
EBF1	-0.13574	0.008144	0.021747
C21orf49	-0.13567	0.008174	0.021817
C1S	-0.13566	0.00818	0.021825
ATF2	-0.13564	0.008188	0.021837
PCGF1	-0.13562	0.0082	0.021863
ARHGEF15	-0.1355	0.008255	0.022003
LRRC2	-0.13549	0.00826	0.022014
SNORD11(	-0.13548	0.008268	0.022033
MPZL3	-0.13544	0.008288	0.022076
BNC1	-0.13544	0.008288	0.022076
C8orf85	-0.13541	0.008302	0.02211
TMEM154	-0.13538	0.008313	0.022136
C1R	-0.13537	0.008318	0.022147
GSTT2	-0.13533	0.008338	0.022192
AMZ1	-0.13531	0.008349	0.022218
TMOD1	-0.1353	0.008352	0.022222
SPAG17	-0.13527	0.008366	0.022257
POLR3C	-0.13527	0.00837	0.022264
A2M	-0.13525	0.008379	0.022285
TSKS	-0.13523	0.008386	0.022297
INTS8	-0.13521	0.008397	0.02232
GDF9	-0.13504	0.00848	0.022524
GARNL3	-0.13503	0.008485	0.022535
COL6A3	-0.135	0.008499	0.022567
HOTAIR	-0.13499	0.008505	0.022573
CCL14	-0.13495	0.008526	0.022626
PHACTR2	-0.13485	0.008572	0.022732
ST8SIA2	-0.13485	0.008573	0.022732
ARHGAP18	-0.13483	0.008586	0.02276
SCARNA23	-0.13482	0.008588	0.022761
KLHL35	-0.13481	0.008596	0.022774
TCEA1	-0.13479	0.008601	0.022785
SLC38A9	-0.13474	0.008629	0.022853
LAT2	-0.13471	0.008643	0.022884
IKZF5	-0.1347	0.008648	0.022893
WFDC11	-0.13468	0.008659	0.022913
GRLF1	-0.13467	0.008664	0.022923
MPRIP	-0.13459	0.008704	0.023019
CCDC142	-0.13459	0.008705	0.023019
CYTH2	-0.13458	0.008706	0.02302
OSMR	-0.13456	0.008717	0.023045
ZNF671	-0.13452	0.008737	0.023095
ATP6V1C2	-0.13451	0.008742	0.023105
SDCCAG8	-0.13451	0.008745	0.023109
LPHN2	-0.13451	0.008746	0.023109
NBPF10	-0.13449	0.008753	0.023121
ROMO1	-0.13448	0.008758	0.023132
CCDC37	-0.13444	0.008781	0.023185
FER	-0.13443	0.008784	0.02319
LRRC16B	-0.13442	0.008788	0.023197
DMRTC1	-0.13441	0.008794	0.023209
NCRNA00(	-0.13438	0.008808	0.02323
ZNF195	-0.13434	0.008832	0.023278

GFAP	-0.1343	0.00885	0.023312
STMN4	-0.1343	0.008853	0.023314
MYH1	-0.13429	0.008854	0.023314
ZMAT1	-0.13424	0.008883	0.023386
ST6GALNA	-0.13423	0.008885	0.023388
KIF6	-0.13423	0.008888	0.023392
RBM18	-0.1342	0.008901	0.023423
KRT37	-0.13418	0.008912	0.023445
GPNMB	-0.13418	0.008914	0.023448
NNMT	-0.13411	0.008948	0.023526
CLVS2	-0.13411	0.00895	0.023526
FAM150A	-0.13409	0.008956	0.023539
EMX2OS	-0.13408	0.008965	0.023557
PRNP	-0.13406	0.008974	0.023578
SHKBP1	-0.13406	0.008976	0.023579
GCFC1	-0.13405	0.008979	0.023584
NECAB2	-0.13403	0.008987	0.023601
ZBBX	-0.13403	0.008988	0.023601
MYLK2	-0.13402	0.008997	0.023619
KIAA2026	-0.13399	0.009011	0.023651
RFPL3S	-0.13398	0.009013	0.023653
ZNF140	-0.13397	0.009018	0.023662
RLIM	-0.13389	0.009063	0.023769
TAS2R4	-0.13386	0.009077	0.0238
CDH6	-0.13385	0.009082	0.02381
C17orf86	-0.13385	0.009083	0.02381
C10orf47	-0.13383	0.009094	0.023832
GAB1	-0.13378	0.009119	0.023891
SCRIB	-0.13375	0.009133	0.023923
TAB2	-0.13375	0.009136	0.023928
GPR4	-0.13373	0.009148	0.023954
NPAS3	-0.1337	0.00916	0.023977
RASSF4	-0.1337	0.00916	0.023977
DHX40	-0.13364	0.009194	0.024061
DACH2	-0.13359	0.009221	0.024122
DDX19A	-0.13359	0.009222	0.024122
CRTC2	-0.13358	0.009225	0.024128
PPDPF	-0.13357	0.009228	0.024132
GPR162	-0.13356	0.009234	0.024146
SYN3	-0.13355	0.00924	0.024155
TNFSF18	-0.13354	0.009245	0.024165
EMP3	-0.13346	0.00929	0.024274
AMY1A	-0.13341	0.009317	0.024339
FIGF	-0.13339	0.009325	0.024355
CHST11	-0.13333	0.009358	0.02443
LPPR4	-0.13328	0.009383	0.024491
SNN	-0.13324	0.009404	0.024539
CTF1	-0.13323	0.009411	0.024544
ZDHHC8	-0.13323	0.009411	0.024544
INPPL1	-0.13323	0.009412	0.024544
LYRM4	-0.13317	0.009446	0.02462
SYNGR1	-0.13309	0.009486	0.024711
CNTN4	-0.13307	0.009497	0.024736
AGAP6	-0.13304	0.009517	0.024784
THAP5	-0.13298	0.009545	0.024854
CCNL2	-0.13297	0.00955	0.024861
RIF1	-0.13297	0.009551	0.024861
GLRA3	-0.13295	0.009566	0.024898

ACTN4	-0.13292	0.009581	0.024925
ZNF233	-0.13292	0.009581	0.024925
RBM4B	-0.13291	0.009583	0.024927
EIF4EBP2	-0.13291	0.009586	0.024932
XKR5	-0.1329	0.009589	0.024936
TBC1D28	-0.13289	0.009596	0.02495
DGKE	-0.13285	0.009618	0.024998
PHYHD1	-0.13283	0.009629	0.025022
ZNF513	-0.13282	0.009637	0.025037
FAM138B	-0.13281	0.009638	0.025037
ZNF91	-0.13279	0.009653	0.025072
GAST	-0.13278	0.009659	0.025085
ZNF205	-0.13271	0.009696	0.025175
WNK3	-0.1327	0.009702	0.025185
PRDM2	-0.1327	0.009703	0.025185
DGKH	-0.13268	0.00971	0.025201
TMEM194I	-0.13267	0.009716	0.02521
SYT4	-0.13261	0.009752	0.025293
LRRK2	-0.13259	0.009761	0.025313
GSX1	-0.13249	0.009821	0.025457
LOC72872	-0.13247	0.009828	0.025472
MEIG1	-0.13245	0.009839	0.025495
KIAA0467	-0.13243	0.009854	0.025529
NR1D1	-0.13242	0.009857	0.025536
CPLX1	-0.13241	0.009865	0.025552
STON1-G	-0.1324	0.00987	0.025563
THOC5	-0.13238	0.009878	0.025579
BDH2	-0.13237	0.009883	0.025589
KIAA1143	-0.13237	0.009886	0.025593
RBP7	-0.13234	0.0099	0.025626
FAM125B	-0.13231	0.00992	0.025668
HSPA1L	-0.13225	0.009951	0.025744
CRELD1	-0.1322	0.009979	0.025804
HSPC157	-0.13215	0.010009	0.025878
PFN4	-0.13212	0.010028	0.025925
CYP2F1	-0.1321	0.01004	0.025951
ADCYAP1I	-0.13201	0.010088	0.026068
NOS3	-0.13195	0.010124	0.026155
LGR5	-0.13194	0.010132	0.026173
HSPA1A	-0.13192	0.010143	0.026195
ALK	-0.13186	0.010175	0.026266
KPNA1	-0.13185	0.010182	0.026281
C7orf42	-0.13184	0.010189	0.026294
CCDC27	-0.13183	0.010191	0.026294
TESC	-0.13183	0.010191	0.026294
SCAND1	-0.13183	0.010192	0.026294
LBX1	-0.1318	0.010209	0.026331
TAGLN3	-0.1318	0.01021	0.026331
FLJ42709	-0.1318	0.010211	0.026331
RXRB	-0.13178	0.010222	0.026357
PRND	-0.13176	0.010234	0.026384
C18orf1	-0.13175	0.010242	0.026401
FDX1L	-0.13173	0.010251	0.02642
DNAJB9	-0.13173	0.010252	0.02642
MYO5A	-0.13171	0.010262	0.026442
FREM1	-0.13171	0.010265	0.026448
PCDHGB4	-0.13169	0.010273	0.026463
MMP21	-0.13168	0.010283	0.026486

TANC1	-0.13164	0.010305	0.026532
WBP1	-0.13163	0.010307	0.026534
ZNF131	-0.13162	0.010314	0.026549
ABCC5	-0.13162	0.010316	0.026549
ATF6B	-0.13159	0.01033	0.026577
PTPRN2	-0.13155	0.010354	0.02663
ZNF556	-0.13155	0.010355	0.02663
ZNF256	-0.13154	0.010362	0.026645
MRP63	-0.13152	0.010372	0.026667
RIT1	-0.13147	0.010404	0.026739
ZNF37B	-0.13145	0.010413	0.026758
MAN1C1	-0.13139	0.010451	0.026834
STK31	-0.13138	0.010454	0.026834
ELSPBP1	-0.13138	0.010454	0.026834
SNRNP48	-0.13138	0.010458	0.026839
RLTPR	-0.13136	0.010467	0.026858
NUDT21	-0.13133	0.010487	0.0269
ANXA5	-0.1313	0.010504	0.026939
SPIN3	-0.13123	0.010548	0.027042
AKTIP	-0.13122	0.010551	0.027047
ZNF518B	-0.13115	0.010592	0.027142
C3orf71	-0.13113	0.010607	0.027172
TRIM17	-0.13111	0.010618	0.027194
KRT10	-0.13106	0.010648	0.027259
KCNT2	-0.13104	0.010659	0.027281
TMEM41A	-0.13103	0.010664	0.027291
PALMD	-0.13103	0.010667	0.027295
DCP2	-0.13101	0.010678	0.02732
C13orf27	-0.13097	0.010703	0.027377
OR2K2	-0.13089	0.01075	0.027489
DDX6	-0.13087	0.010759	0.027509
DCN	-0.13085	0.010774	0.027542
EFNB3	-0.13085	0.010775	0.027542
CRCP	-0.13085	0.010776	0.027543
HSPB1	-0.13083	0.010787	0.027566
CNP	-0.13083	0.010788	0.027566
NRBP2	-0.1308	0.010806	0.027606
INGX	-0.1308	0.010807	0.027606
MEGF10	-0.13076	0.010829	0.027658
ASGR1	-0.13073	0.010845	0.027693
ACSL4	-0.13073	0.010849	0.027695
C4orf47	-0.13073	0.01085	0.027695
BTBD8	-0.1307	0.010868	0.027732
CUL1	-0.13069	0.01087	0.027732
LOC10013	-0.13066	0.010892	0.027773
LOC15018	-0.13059	0.010931	0.027858
SSH1	-0.13057	0.010944	0.027883
PDX1	-0.13057	0.010945	0.027883
FLJ12825	-0.13055	0.010957	0.027907
C20orf132	-0.13054	0.010965	0.027919
PCDHGA7	-0.13053	0.010969	0.027922
SLC22A11	-0.1305	0.010987	0.027957
ZNF662	-0.13049	0.010994	0.027973
DNAJB4	-0.13048	0.010999	0.02798
SUCLA2	-0.13046	0.011015	0.028018
PKP1	-0.13045	0.011022	0.02803
LOC44245	-0.13045	0.011022	0.02803
ZNF836	-0.13038	0.011062	0.028117

GOLGA9P	-0.13032	0.011097	0.0282
NOX5	-0.1303	0.011115	0.028238
NRSN2	-0.13025	0.011145	0.028302
CLASP2	-0.13025	0.011147	0.028305
psiTPTE22	-0.13021	0.011166	0.028345
C2orf52	-0.13019	0.011179	0.028374
OSGEPL1	-0.13016	0.011202	0.028429
C7orf25	-0.13015	0.011204	0.02843
FAM66E	-0.13011	0.011231	0.028488
ID4	-0.1301	0.011236	0.028493
NUDT9P1	-0.13009	0.011244	0.02851
WFDC8	-0.13	0.011302	0.028639
TBC1D26	-0.12995	0.011336	0.028717
LRP1B	-0.12993	0.011344	0.028731
OR2J3	-0.12993	0.011346	0.028732
WISP2	-0.12989	0.01137	0.028784
PAR5	-0.1298	0.01143	0.028927
DACT2	-0.12977	0.011448	0.028967
FSD1L	-0.12977	0.01145	0.02897
ASB14	-0.12975	0.011461	0.028994
TNFAIP8L3	-0.12971	0.011486	0.029053
AKR1C4	-0.12967	0.011514	0.02912
HPD	-0.12966	0.011518	0.029125
HNMT	-0.12965	0.011527	0.029146
CHERP	-0.12964	0.011532	0.029153
ENY2	-0.12955	0.011591	0.029299
BCL2L11	-0.12955	0.011592	0.029299
LOC34035	-0.12951	0.011615	0.029351
IRX5	-0.1295	0.011625	0.029368
FAM188B	-0.1295	0.011626	0.029368
SLC27A1	-0.12949	0.011628	0.029372
LOC92659	-0.12948	0.011637	0.029386
HCG9	-0.12946	0.01165	0.029414
DPY19L2	-0.12942	0.011675	0.029471
CACNG6	-0.12941	0.011682	0.02948
DUSP19	-0.12939	0.011692	0.029498
STK19	-0.12939	0.011697	0.029499
LRIT1	-0.12938	0.011701	0.029503
ALAD	-0.12936	0.011711	0.029517
BRPF1	-0.12931	0.011748	0.029603
MED28	-0.12927	0.011776	0.029664
SYNE1	-0.12924	0.011791	0.0297
SLC44A5	-0.12922	0.011805	0.029727
FZD1	-0.12922	0.011808	0.029728
BTN2A2	-0.12921	0.011815	0.02974
LRRC43	-0.1292	0.011818	0.02974
ZNF624	-0.12919	0.011826	0.029754
STXBP1	-0.12918	0.01183	0.02976
ROR2	-0.12917	0.011842	0.029785
C2orf56	-0.12916	0.011847	0.029793
MOAP1	-0.12916	0.011848	0.029793
SLC35D3	-0.12911	0.011878	0.029865
FNDC3B	-0.12907	0.011902	0.029918
GLIS1	-0.12906	0.011913	0.029942
LOC38842	-0.12903	0.011932	0.029983
GJA4	-0.12902	0.01194	0.029997
KHDRBS3	-0.12899	0.011958	0.030038
PCNXL2	-0.12899	0.011959	0.030038

TMED10P1	-0.12897	0.011975	0.03007
THAP2	-0.12894	0.011988	0.0301
LOC22144	-0.12893	0.011999	0.030125
GLT8D2	-0.12889	0.012022	0.030166
SYT9	-0.12889	0.012025	0.030166
NUP153	-0.12886	0.012042	0.030202
FAM138F	-0.12886	0.012043	0.030202
LOC40004	-0.12885	0.012052	0.030219
GYG1	-0.12883	0.012063	0.030243
VPS72	-0.12883	0.012067	0.030249
FCGR1A	-0.12882	0.012073	0.030257
KCTD10	-0.12876	0.012109	0.030327
ENGASE	-0.12872	0.012138	0.030388
KCTD21	-0.12871	0.012143	0.030396
DNAI1	-0.12871	0.012149	0.030407
ATP8B2	-0.12867	0.012175	0.030454
BRWD3	-0.12865	0.012189	0.030484
CTCF	-0.12862	0.012205	0.030522
UBE2Q2P1	-0.12861	0.012213	0.030536
C1QTNF7	-0.1286	0.012217	0.030539
SYNC	-0.12858	0.012237	0.030582
FAM71F2	-0.12856	0.012246	0.030599
DCAF5	-0.12853	0.012265	0.03064
NMNAT2	-0.12853	0.012267	0.030641
ADPRH	-0.1285	0.012291	0.030689
ARMCX6	-0.12844	0.012328	0.030771
CRIPT	-0.12841	0.012347	0.030804
LOC10017	-0.12841	0.012348	0.030804
ZNF669	-0.12841	0.012352	0.030812
TGFBR1	-0.12839	0.012365	0.03084
TSPAN6	-0.12838	0.012372	0.030853
SCAPER	-0.12837	0.012379	0.030865
RNF214	-0.12835	0.012391	0.030881
RRS1	-0.12833	0.012408	0.030919
KCNE1	-0.12832	0.012414	0.030922
GPIHBP1	-0.1283	0.012424	0.030943
GRIA3	-0.12823	0.012471	0.031048
ANKHD1	-0.12817	0.012513	0.03115
PABPC1L2	-0.12811	0.012556	0.031249
VMA21	-0.1281	0.012563	0.031259
SRPX2	-0.12805	0.012597	0.031332
SCN9A	-0.12801	0.012623	0.031385
TMEM106I	-0.12798	0.012647	0.03144
C21orf90	-0.12797	0.012657	0.03146
PRPS1L1	-0.12796	0.012659	0.031462
PTPLAD2	-0.12793	0.012682	0.031516
C21orf129	-0.12791	0.0127	0.031556
DZIP1L	-0.1279	0.012706	0.031567
HEATR5B	-0.12788	0.012719	0.031593
SIRT2	-0.12786	0.012733	0.031615
CAMK2N1	-0.12785	0.012742	0.031632
ASTN1	-0.12784	0.012749	0.031646
FAM105B	-0.12781	0.012769	0.031691
CHST8	-0.12781	0.01277	0.031691
NAP1L2	-0.12779	0.012779	0.031708
MASP2	-0.12778	0.012788	0.031727
FLJ43663	-0.12777	0.012795	0.031741
C10orf11	-0.12772	0.012831	0.031822

C9orf4	-0.12772	0.012834	0.031825
PLEKHG4	-0.12771	0.01284	0.031833
LOC10012	-0.12771	0.012841	0.031833
UQCRB	-0.1277	0.012842	0.031833
MNX1	-0.12763	0.012893	0.031949
PCDHA13	-0.12763	0.012894	0.031949
SEPHS1	-0.12762	0.012902	0.031966
SLC25A15	-0.1276	0.012914	0.03199
RECQL5	-0.12756	0.012946	0.032063
PROK1	-0.12753	0.012963	0.032096
LIN7B	-0.12752	0.012972	0.03211
SPEN	-0.12752	0.012975	0.032114
TMEM151/	-0.12747	0.013007	0.032185
CPE	-0.12747	0.013009	0.032187
MAP3K1	-0.12746	0.013015	0.032198
NUDT4	-0.12746	0.013018	0.032201
CRNKL1	-0.12745	0.013025	0.032204
NFATC4	-0.12744	0.013034	0.032219
KIAA1712	-0.12743	0.013036	0.032221
HGS	-0.12741	0.01305	0.032251
ZNF394	-0.12727	0.013155	0.032483
WSB1	-0.12724	0.013174	0.032523
LOC14772	-0.12723	0.013185	0.032542
ITSN2	-0.1272	0.013207	0.032587
GNG3	-0.12717	0.013227	0.032628
KIAA1430	-0.12716	0.013234	0.032641
GRIPAP1	-0.12715	0.013244	0.032662
UBE2Q1	-0.12714	0.013248	0.032669
SLC26A1	-0.12713	0.013255	0.032682
LOC28635	-0.1271	0.013274	0.032724
ZNF384	-0.12707	0.013302	0.032789
TIMP1	-0.12705	0.013312	0.032804
PCSK4	-0.12702	0.013335	0.032853
SLC12A1	-0.12701	0.013342	0.032866
PKDCC	-0.12698	0.013364	0.032913
ODF3L1	-0.12698	0.013366	0.032914
HIPK4	-0.12696	0.013383	0.032945
COL6A1	-0.12695	0.013384	0.032945
ZBTB47	-0.12695	0.013386	0.032946
FGFR4	-0.12689	0.013433	0.033055
SNED1	-0.12687	0.013446	0.033083
SLC7A5	-0.12687	0.013449	0.033085
C1orf203	-0.12686	0.013451	0.033087
ATP6AP2	-0.12685	0.013463	0.033112
C14orf19	-0.12683	0.013477	0.03314
C12orf35	-0.12678	0.013509	0.03321
SCARNA16	-0.12678	0.01351	0.03321
C1orf227	-0.12677	0.013519	0.033229
SLC13A5	-0.12671	0.013563	0.033325
SERPINA5	-0.1267	0.013572	0.033338
FAM192A	-0.12669	0.013578	0.03335
OR52K1	-0.12663	0.013623	0.033448
MTMR12	-0.12661	0.01364	0.03348
RGS4	-0.1266	0.013644	0.033486
RFX7	-0.1266	0.013647	0.03349
C7	-0.12652	0.013704	0.033602
SCARNA9I	-0.1265	0.013721	0.033637
CXCL12	-0.1265	0.013724	0.033641

CYB5B	-0.12646	0.013749	0.033697
ZNF749	-0.12646	0.013754	0.033702
GPR83	-0.1264	0.013796	0.033795
NKAIN2	-0.12639	0.013806	0.033813
C9orf130	-0.12638	0.01381	0.033819
TTC30B	-0.12636	0.01383	0.033862
C3orf64	-0.12635	0.013838	0.033877
KCTD15	-0.12631	0.013863	0.03393
HES7	-0.1263	0.013877	0.033956
NFIX	-0.12627	0.013894	0.033989
PTPRT	-0.12626	0.013905	0.034013
RAPSN	-0.12625	0.013913	0.034028
CWC15	-0.12622	0.013934	0.034075
ASCC1	-0.1262	0.01395	0.03411
FAM22F	-0.12616	0.013977	0.034168
PGBD3	-0.12616	0.013982	0.034169
HIST1H2B/	-0.12616	0.013982	0.034169
CAPN7	-0.12616	0.013982	0.034169
ZNF484	-0.12615	0.013984	0.034169
MTMR2	-0.12613	0.014004	0.034208
UNC13A	-0.12609	0.014035	0.03428
PLCD4	-0.12607	0.014045	0.034301
SETBP1	-0.12607	0.014051	0.03431
FLJ10213	-0.12603	0.014079	0.034375
DNAJC24	-0.126	0.014106	0.034433
C17orf105	-0.126	0.014106	0.034433
BLOC1S3	-0.12599	0.014108	0.034433
GPR176	-0.12598	0.014118	0.034453
ARHGAP4:	-0.12597	0.014126	0.034467
SLC2A4	-0.12596	0.014134	0.034481
CELF4	-0.12591	0.014172	0.034554
C7orf68	-0.12589	0.014187	0.034584
DIO2	-0.12588	0.014196	0.034602
SIRT4	-0.12585	0.014216	0.034645
SYF2	-0.12585	0.014221	0.034653
GRK5	-0.12581	0.014247	0.034712
KRBA1	-0.12579	0.014268	0.034754
KCNJ5	-0.12573	0.014308	0.034835
ARL5A	-0.12573	0.01431	0.034837
CCDC90A	-0.12571	0.014324	0.034865
STAMBP	-0.12571	0.014327	0.034868
BAIAP2	-0.12565	0.014374	0.034966
PRR19	-0.12558	0.014427	0.03509
SKIV2L	-0.12557	0.014439	0.035111
C4orf44	-0.12556	0.014441	0.035113
C9orf50	-0.12549	0.014499	0.035234
FOXJ3	-0.12549	0.014501	0.035235
AXIN2	-0.12545	0.014534	0.035301
NLGN4Y	-0.12544	0.014539	0.035301
C4A	-0.12544	0.014539	0.035301
MAP1LC3/	-0.12544	0.01454	0.035301
HEG1	-0.12542	0.014556	0.035331
LOC67865	-0.12539	0.01458	0.03538
DUSP9	-0.12538	0.014584	0.035386
CUL5	-0.12536	0.014599	0.035418
BAT2L2	-0.12536	0.014603	0.035423
DSN1	-0.12533	0.01463	0.035483
AMACR	-0.12531	0.014641	0.035505

PRAMEF20	-0.1253	0.014647	0.035513
XIAP	-0.12529	0.01466	0.035539
SCNM1	-0.12527	0.014676	0.035565
RPL37	-0.12524	0.014695	0.035605
SUCNR1	-0.12518	0.01475	0.035726
HINT3	-0.12517	0.014751	0.035726
ARID5B	-0.12515	0.01477	0.035764
STMN2	-0.12515	0.014771	0.035764
ZNF607	-0.1251	0.014813	0.035859
GAS7	-0.12504	0.014855	0.035947
FAM168A	-0.12504	0.014861	0.035953
LUC7L	-0.12502	0.014875	0.035982
FNDC4	-0.12497	0.014917	0.036073
C13orf38	-0.12489	0.014978	0.036192
HRH3	-0.12486	0.015006	0.036253
C5orf45	-0.12486	0.015007	0.036253
C19orf40	-0.12483	0.015029	0.036293
HERC2	-0.12479	0.01506	0.036362
SEC61A2	-0.12472	0.015115	0.036491
POMGNT1	-0.12469	0.015144	0.036553
GPR111	-0.12466	0.015168	0.036605
LRRC24	-0.12465	0.015178	0.036626
ITM2B	-0.12464	0.015183	0.036633
NOTCH2	-0.12463	0.015196	0.03665
CGREF1	-0.12459	0.015228	0.036723
TMEM209	-0.12457	0.015243	0.036756
KRTAP5-7	-0.12453	0.015272	0.036806
CCDC147	-0.12453	0.015272	0.036806
PLCB1	-0.12453	0.015275	0.036806
LOC40075	-0.12453	0.015275	0.036806
LOXL2	-0.12451	0.015289	0.036834
RORB	-0.12449	0.015308	0.036877
RDH10	-0.12448	0.015316	0.036888
PEG3AS	-0.12446	0.015334	0.036926
PTX3	-0.12445	0.015343	0.036943
GPR22	-0.12443	0.01536	0.036979
KIAA0495	-0.12442	0.01537	0.036994
ANGPTL7	-0.12441	0.015377	0.037005
AMELX	-0.1244	0.015382	0.037013
GCK	-0.12439	0.015392	0.037034
KLF10	-0.12438	0.015397	0.037037
FBXL6	-0.12435	0.015423	0.037082
UBQLNL	-0.12434	0.015432	0.037098
ARGFXP2	-0.12432	0.015453	0.037144
UBE2D4	-0.12431	0.015459	0.037154
PTHLH	-0.12428	0.015487	0.037213
AGAP4	-0.12427	0.015493	0.037221
TTC32	-0.12424	0.015516	0.037267
PPM1M	-0.12423	0.015527	0.037291
KRTAP1-1	-0.1242	0.015553	0.037348
C13orf39	-0.12416	0.015585	0.037417
LRRC37A	-0.12414	0.015601	0.037451
C8orf38	-0.12408	0.015651	0.037552
OCM	-0.12401	0.015711	0.037672
UBR3	-0.12388	0.015825	0.037942
PYCR2	-0.12384	0.015852	0.038002
BAT1	-0.12382	0.01587	0.038031
APOE	-0.12381	0.015884	0.038059

DDX55	-0.12376	0.01592	0.038131
SPANXN5	-0.12374	0.015943	0.038178
SNTB2	-0.1237	0.015975	0.038245
FRMD7	-0.12363	0.016033	0.038381
GRRP1	-0.12363	0.016035	0.038381
FGF17	-0.1236	0.016063	0.038437
LY6E	-0.12359	0.016067	0.038443
EXOSC5	-0.12359	0.016074	0.038454
CLCF1	-0.12355	0.016102	0.038518
RNF169	-0.12354	0.016111	0.038533
C2orf84	-0.12354	0.016112	0.038533
RPS2P32	-0.1235	0.01615	0.038609
PRDXDD1I	-0.12344	0.0162	0.038716
LOC15153	-0.12344	0.016201	0.038716
SLC7A9	-0.12342	0.016214	0.03874
ADAMTS1	-0.12342	0.016221	0.03875
PILRB	-0.12341	0.016224	0.03875
SLCO4A1	-0.12341	0.016225	0.03875
HECW1	-0.12337	0.016262	0.038829
RC3H2	-0.12335	0.016278	0.038862
CEP164	-0.12332	0.016306	0.03892
CTNNA3	-0.1233	0.016319	0.038943
SIK3	-0.12326	0.016355	0.039023
MICAL1	-0.12324	0.016378	0.039075
FAM49B	-0.12321	0.0164	0.039113
ALX4	-0.12321	0.016404	0.039113
ZNF462	-0.12319	0.016414	0.039133
RUFY2	-0.12319	0.016417	0.039134
CDR1	-0.12319	0.016421	0.03914
GPBP1	-0.12318	0.016428	0.039151
ETV5	-0.12316	0.016442	0.03918
ANGPTL5	-0.12312	0.016483	0.039258
NDFIP2	-0.1231	0.016497	0.039286
LRRC34	-0.1231	0.016499	0.039287
PPP1R12C	-0.12308	0.016512	0.039314
CDK12	-0.12302	0.016568	0.039434
PRR25	-0.12302	0.01657	0.039434
PTPRU	-0.12302	0.016571	0.039434
HEATR4	-0.12298	0.016606	0.039504
PTN	-0.12296	0.016619	0.03953
TSR2	-0.12296	0.016626	0.039541
RNF185	-0.12295	0.01663	0.039548
WDR67	-0.12295	0.016634	0.039552
ZNF20	-0.12294	0.016643	0.039567
SRD5A2	-0.12292	0.016662	0.039608
KCNG1	-0.1229	0.016674	0.039631
ZHX1	-0.12284	0.016725	0.039729
DCLRE1C	-0.12284	0.016729	0.039736
ITPR1	-0.12281	0.016753	0.039783
VPS8	-0.1228	0.016768	0.039809
SLC9A10	-0.12275	0.01681	0.039897
CLEC14A	-0.12274	0.016821	0.03992
MAF	-0.12273	0.016828	0.039931
ANKRD26	-0.12271	0.016845	0.039962
LRRN3	-0.12267	0.016886	0.040046
STK38L	-0.12262	0.016923	0.040128
ACTL6B	-0.1226	0.016942	0.040164
CRKL	-0.12258	0.016961	0.040204

OPHN1	-0.12257	0.016976	0.040227
FAM126A	-0.12256	0.016977	0.040227
RBM5	-0.12256	0.016985	0.040242
MCHR1	-0.12255	0.016989	0.040246
CAMK1G	-0.12251	0.017025	0.040327
ROCK2	-0.12247	0.017062	0.040405
FKBP9L	-0.12247	0.017065	0.040407
PPFIA4	-0.12246	0.017068	0.04041
PLP1	-0.12245	0.017077	0.040426
STON2	-0.12245	0.01708	0.040427
ZACN	-0.12244	0.017094	0.040453
MMP24	-0.12243	0.017095	0.040453
OFD1	-0.12243	0.017102	0.040457
DPP7	-0.12243	0.017102	0.040457
CFHR3	-0.12242	0.017112	0.040465
SAMD12	-0.1224	0.017127	0.040491
TMEM51	-0.12238	0.017142	0.040522
COL6A4P2	-0.12238	0.017145	0.040525
FLJ43860	-0.12237	0.017151	0.040534
RAB6A	-0.12232	0.017198	0.040628
SYNPO	-0.1223	0.017223	0.040683
TMEM132	-0.12229	0.01723	0.040695
NCRNA00	-0.12228	0.017232	0.040696
RSBN1L	-0.12225	0.017265	0.040769
SVEP1	-0.12224	0.017273	0.040783
MUM1L1	-0.12218	0.017328	0.040898
C1QL4	-0.12217	0.017334	0.040907
CSAD	-0.12217	0.017341	0.040919
MDGA1	-0.12215	0.017356	0.040949
LOC28579	-0.12211	0.017392	0.04103
SIPA1	-0.1221	0.017403	0.041049
RHEBL1	-0.12206	0.017436	0.041121
PRDM13	-0.12202	0.017478	0.041202
ARMC5	-0.12199	0.017501	0.041253
GZMB	-0.12195	0.017539	0.041332
POU4F3	-0.12194	0.017548	0.041348
TAL2	-0.12193	0.017563	0.041377
CHRNA2	-0.1219	0.017591	0.041424
KBTBD5	-0.12183	0.017652	0.041548
C15orf62	-0.12182	0.017663	0.041569
MAP1LC3I	-0.1218	0.017685	0.041612
SIM2	-0.12177	0.017715	0.041672
STARD3NI	-0.12175	0.017726	0.041693
HCN3	-0.12169	0.017781	0.041813
SPOP	-0.12169	0.01779	0.041829
ZCCHC4	-0.12165	0.017822	0.041889
IGFBP1	-0.12165	0.017826	0.041889
REST	-0.12163	0.017839	0.04191
FAM75A3	-0.12158	0.017888	0.041992
FBLN2	-0.12158	0.017889	0.041992
LRRC37B2	-0.12158	0.017891	0.041992
CDK10	-0.12148	0.017983	0.042177
SNTA1	-0.12146	0.018006	0.042221
EP400	-0.12138	0.018079	0.042373
CSTL1	-0.12132	0.018141	0.042502
TAF1L	-0.1213	0.018158	0.042533
WDR91	-0.12128	0.018179	0.042576
PLEKHO1	-0.12127	0.018182	0.042578

ZNF699	-0.12127	0.018188	0.042588
LOC10027	-0.12126	0.0182	0.04261
MAFB	-0.12125	0.018209	0.042626
PAX7	-0.12123	0.018225	0.042653
ADCY1	-0.12121	0.018249	0.042705
ERCC6	-0.12119	0.018268	0.042745
NGB	-0.12116	0.018297	0.042807
SNX10	-0.12115	0.018299	0.042808
GPR89A	-0.12115	0.018307	0.042821
LIMS3	-0.12111	0.018339	0.042884
CASC1	-0.1211	0.01835	0.042907
ELTD1	-0.1211	0.018353	0.042909
KRT3	-0.12108	0.018366	0.042934
ZNF765	-0.12106	0.018386	0.042975
ZFP37	-0.12104	0.018412	0.04303
FLJ36031	-0.12104	0.018414	0.04303
SSTR5	-0.12103	0.018421	0.043042
IL11RA	-0.12102	0.018426	0.043047
OR11H6	-0.12102	0.018432	0.043052
H2AFY2	-0.12097	0.018476	0.04315
PMS2	-0.12097	0.018478	0.04315
IGF2BP2	-0.12096	0.018488	0.043168
GOLGA8G	-0.12092	0.018525	0.043243
EGFLAM	-0.1209	0.018547	0.04328
MFAP5	-0.1209	0.018547	0.04328
TUBA4B	-0.12089	0.018556	0.043296
CCL21	-0.12088	0.018562	0.043304
MTSS1	-0.12088	0.01857	0.043318
XRCC1	-0.12087	0.018573	0.043321
ZNF692	-0.12085	0.018591	0.043358
UPF3B	-0.12083	0.018617	0.043413
KCNK9	-0.12076	0.018687	0.043555
MTHFD1L	-0.12073	0.018712	0.043608
PREX2	-0.12072	0.018723	0.04363
CCDC53	-0.12071	0.018735	0.043653
KIAA0556	-0.12067	0.018767	0.043712
C11orf57	-0.12066	0.018784	0.043746
BAZ1B	-0.12065	0.018796	0.043762
SPRED3	-0.12063	0.018812	0.043793
TXNDC3	-0.1206	0.01884	0.043842
TRPC3	-0.1206	0.018841	0.043842
CHST15	-0.12058	0.018863	0.043879
ZFYVE16	-0.12054	0.018902	0.043963
PAH	-0.12052	0.018923	0.044003
FAM46B	-0.12046	0.018986	0.044143
CPNE9	-0.12044	0.018997	0.044165
KRTAP4-1	-0.12044	0.019006	0.044179
C1orf175	-0.12043	0.019013	0.044186
IGFBPL1	-0.12037	0.019071	0.044309
LRRC10	-0.12035	0.019092	0.044341
UBXN4	-0.12031	0.019127	0.044414
MEGF6	-0.1203	0.019145	0.044449
LMF1	-0.12027	0.019168	0.044499
C11orf67	-0.12027	0.019174	0.044506
ZNF501	-0.12021	0.019228	0.044611
CYP7B1	-0.12019	0.019252	0.044658
KIAA1407	-0.12015	0.019295	0.044747
SUMO1P3	-0.12013	0.019309	0.04477

SLC26A7	-0.12013	0.01931	0.04477
SPHK1	-0.12013	0.019316	0.044779
STYXL1	-0.12009	0.01935	0.044841
44626	-0.12006	0.019388	0.044915
FAM200B	-0.12004	0.019408	0.044955
CSDE1	-0.11999	0.019453	0.045056
EHD3	-0.11996	0.019483	0.045113
GPHA2	-0.11994	0.01951	0.045171
FAM178B	-0.11992	0.019525	0.045198
LOC10013	-0.11992	0.019526	0.045198
FAM183A	-0.11989	0.019558	0.045267
GPR52	-0.11988	0.019572	0.045293
EXOSC8	-0.11985	0.019603	0.045354
TAS2R3	-0.11983	0.019619	0.045386
GDPD1	-0.11983	0.019622	0.045388
XIRP1	-0.11982	0.01963	0.0454
TPI1P2	-0.11977	0.019685	0.045513
FLJ25758	-0.11974	0.019716	0.045568
ZNF597	-0.11973	0.019724	0.045581
BTN2A3	-0.11972	0.019733	0.045597
BAALC	-0.11971	0.019743	0.045615
ZNF530	-0.11965	0.019802	0.045745
CRISPLD2	-0.11965	0.01981	0.045758
GHDC	-0.11963	0.019831	0.045798
GABRA6	-0.11962	0.019838	0.045803
PRSS22	-0.1196	0.01986	0.045845
C14orf118	-0.11953	0.019927	0.045978
ADO	-0.11953	0.019928	0.045978
PSG4	-0.11953	0.019931	0.04598
ZDHHC20	-0.11952	0.019941	0.045996
MID2	-0.11949	0.019973	0.046065
ORMDL3	-0.11948	0.019985	0.046081
GHRH	-0.11948	0.019987	0.046081
PRRX1	-0.11946	0.020003	0.04611
SLC11A1	-0.11946	0.020004	0.04611
LOC44004	-0.11945	0.020016	0.046127
CPNE7	-0.11944	0.020019	0.046129
INE1	-0.11944	0.020025	0.046137
UNC45A	-0.11938	0.02009	0.046265
PMS2L5	-0.11934	0.020125	0.04632
LOC28392	-0.11934	0.020125	0.04632
HIC1	-0.11932	0.020146	0.046357
MSLNL	-0.11927	0.0202	0.046464
SNX30	-0.11926	0.020209	0.04648
RPL13P5	-0.11925	0.020222	0.046504
GAA	-0.11924	0.020236	0.046532
CUL9	-0.11922	0.02026	0.046577
ARL16	-0.11921	0.020261	0.046577
SLC35E2	-0.11919	0.020284	0.046619
ANKRD36	-0.11919	0.020286	0.046619
TTLL10	-0.11911	0.020372	0.046812
ATAD2	-0.11911	0.020377	0.046817
PIP5K1A	-0.11905	0.020431	0.046929
STXBP5L	-0.11905	0.020433	0.046929
COPS6	-0.11905	0.020435	0.046929
ZZZ3	-0.11903	0.020456	0.046967
HEPHL1	-0.11895	0.020547	0.047159
TAC1	-0.11894	0.020557	0.047177

USP6	-0.11891	0.020586	0.047237
JAM2	-0.11887	0.020623	0.047317
ST13	-0.11887	0.020627	0.04732
BRI3	-0.11886	0.020644	0.047348
PPHLN1	-0.11885	0.020652	0.047361
ZNF879	-0.11882	0.020679	0.047412
TAF7	-0.11882	0.020684	0.047413
C8orf12	-0.1188	0.020702	0.047448
KLHL12	-0.11878	0.02072	0.04748
MBD6	-0.11877	0.020735	0.047507
NKAP	-0.11876	0.020742	0.047518
LOC15391	-0.11874	0.020771	0.04758
IGF2R	-0.11869	0.020822	0.047686
DHX57	-0.11867	0.02084	0.047722
HAPLN2	-0.11863	0.02089	0.04783
GAS8	-0.1186	0.020918	0.047882
DKK3	-0.1186	0.020921	0.047884
LOC34884	-0.11857	0.020958	0.047957
RFC1	-0.11856	0.020966	0.047964
C8orf79	-0.11849	0.021036	0.048092
TMEM233	-0.1184	0.021133	0.048299
CGGBP1	-0.1184	0.021134	0.048299
SCRT1	-0.11832	0.021225	0.048475
CPN2	-0.11832	0.021225	0.048475
C15orf41	-0.11829	0.021255	0.048537
BAZ2A	-0.11823	0.021321	0.048675
SPRY4	-0.11822	0.021337	0.048707
C13orf34	-0.11818	0.021384	0.048797
S100A3	-0.11817	0.021393	0.048812
RSRC2	-0.11811	0.021461	0.04894
PCDH8	-0.11809	0.021478	0.048968
RPS25	-0.11809	0.021479	0.048968
GOLGA6L1	-0.11806	0.021515	0.049045
RAB2B	-0.11794	0.021644	0.04931
SYCE1	-0.11793	0.021656	0.049328
ING3	-0.11793	0.02166	0.04933
VWA3A	-0.1179	0.021694	0.049396
ZNF329	-0.11783	0.021768	0.049543
GATA5	-0.11777	0.02184	0.049696
RNF20	-0.11775	0.021862	0.049739
PDE2A	-0.11774	0.021875	0.049763
RXRG	-0.11772	0.021892	0.049782
COMMD2	-0.11768	0.021945	0.04988
ZBTB8B	-0.11767	0.021954	0.049891
SUSD2	-0.11765	0.021977	0.04993
CD248	-0.11764	0.021988	0.04994
ZBTB6	-0.11761	0.02202	0.049995
MIR17HG	-0.1176	0.022027	0.050006
EPO	-0.1176	0.022037	0.050023
LIX1	-0.11759	0.022042	0.050028
SF3B2	-0.11758	0.022059	0.050061
DUSP3	-0.11753	0.022112	0.050164
ASNSD1	-0.11753	0.022114	0.050164
ELL	-0.11753	0.022114	0.050164
INTS4L2	-0.11752	0.022121	0.050173
PHOSPHO	-0.1175	0.022143	0.050218
RNF220	-0.11745	0.022198	0.050331
POFUT2	-0.11743	0.022221	0.050354

CADM2	-0.11742	0.022237	0.050385
C2orf48	-0.11741	0.022244	0.050395
LY6G5C	-0.1174	0.022265	0.050436
AFG3L1	-0.11737	0.022292	0.050486
C17orf56	-0.11736	0.022312	0.050525
TRIM49	-0.11735	0.022316	0.050529
TMEM90B	-0.11734	0.022333	0.050561
ATP1A3	-0.11733	0.022344	0.050581
ANAPC1	-0.11732	0.022356	0.050598
GOLGA6L5	-0.11726	0.022422	0.050728
RPL13	-0.11725	0.022433	0.050736
FBXO43	-0.11725	0.022437	0.050739
NXF2	-0.11721	0.02248	0.050825
ZNF45	-0.11717	0.02252	0.050899
MED1	-0.11715	0.022544	0.050945
SNORA71I	-0.11715	0.022551	0.050956
C7orf31	-0.11714	0.022563	0.050973
EIF4G3	-0.11714	0.022564	0.050973
LOC72999	-0.11707	0.022637	0.051133
CEP350	-0.11706	0.022652	0.051155
OC90	-0.11704	0.022675	0.051201
UBE2H	-0.11703	0.022694	0.051238
ANKRD34/	-0.11701	0.022708	0.051264
WFIKKN2	-0.11701	0.022715	0.051274
FABP3	-0.11699	0.022732	0.051302
VCX3B	-0.11698	0.022752	0.051334
OLFML1	-0.11697	0.022764	0.051356
CHD8	-0.11696	0.022774	0.051373
SCARNA17	-0.11691	0.022831	0.051483
SBSN	-0.11686	0.022882	0.051593
LTC4S	-0.11682	0.022933	0.051696
GPR32	-0.11681	0.02295	0.051722
CIDECP	-0.11674	0.023023	0.051864
LSAMP	-0.11674	0.023034	0.051883
C5orf27	-0.11672	0.023057	0.051921
FOXI2	-0.1167	0.023073	0.051947
ABCA9	-0.11667	0.02311	0.052012
RAET1K	-0.11667	0.023113	0.052012
COX4I2	-0.11666	0.023127	0.052038
TMEM88B	-0.11665	0.02313	0.05204
ZIK1	-0.11663	0.023156	0.052093
SLC1A3	-0.11662	0.023172	0.052123
VIPAR	-0.1166	0.023193	0.052163
SPAG8	-0.11658	0.023214	0.052188
STK32B	-0.11658	0.023215	0.052188
CCDC88A	-0.11651	0.023302	0.05236
SRC	-0.1165	0.023313	0.052379
SQLE	-0.11649	0.023326	0.052403
HOXC12	-0.11647	0.023351	0.052448
MMP9	-0.11646	0.023358	0.052457
PAPOLB	-0.11644	0.023382	0.052506
MARCO	-0.11644	0.023386	0.052507
C21orf54	-0.11643	0.023397	0.052526
ERCC2	-0.11643	0.0234	0.052527
ZMYND11	-0.11641	0.023425	0.052567
LASS2	-0.11641	0.023426	0.052567
MEX3C	-0.11638	0.023463	0.052639
SELE	-0.11632	0.023534	0.052787

ATG2B	-0.11631	0.02354	0.052794
SHFM1	-0.11631	0.023547	0.052803
EIF2C2	-0.1163	0.023552	0.052809
CRBN	-0.11629	0.02356	0.052821
THAP1	-0.11629	0.023564	0.052825
ZNF133	-0.11629	0.023571	0.052834
EPC1	-0.11626	0.023598	0.052889
STAG3L1	-0.11625	0.02361	0.052904
TRIM44	-0.11625	0.023612	0.052904
TPH2	-0.11625	0.023613	0.052904
ZNF93	-0.11624	0.023623	0.052915
APOC1P1	-0.1162	0.023669	0.053005
ZC3H4	-0.11619	0.023683	0.05303
FAM123A	-0.11619	0.02369	0.053034
WNT2	-0.11619	0.023692	0.053034
C2orf16	-0.11619	0.023693	0.053034
TBCK	-0.11617	0.023714	0.053076
RBMXL3	-0.11616	0.023718	0.053078
KCNJ10	-0.116	0.023922	0.053499
ERN1	-0.11595	0.02398	0.053623
RBPJL	-0.11592	0.024014	0.053692
SGK3	-0.11588	0.024062	0.053789
RELN	-0.11588	0.02407	0.053801
EREG	-0.11583	0.024124	0.053908
LOC72875	-0.11582	0.024144	0.05394
NBAS	-0.11579	0.024173	0.053999
OR1C1	-0.11577	0.024194	0.05404
GCOM1	-0.11577	0.024196	0.05404
PIP5KL1	-0.11575	0.024228	0.054105
INTS1	-0.11574	0.024234	0.054112
C3orf63	-0.11571	0.024273	0.054167
EDN1	-0.11566	0.024331	0.05429
NHLRC4	-0.11566	0.024333	0.05429
COPS7B	-0.11565	0.024345	0.054312
KCNH7	-0.11561	0.024395	0.05441
CSNK1E	-0.11561	0.024399	0.05441
LRFN5	-0.11556	0.024462	0.054535
RFTN1	-0.11554	0.024483	0.05457
CCDC102E	-0.11554	0.024484	0.05457
DCAF8	-0.11542	0.024641	0.05489
BACH2	-0.11537	0.024693	0.054988
ZNF664	-0.11537	0.024703	0.055005
CHD9	-0.11535	0.024719	0.055034
PCOLCE	-0.11533	0.024743	0.055061
ARVCF	-0.11533	0.024748	0.055067
OTUD7B	-0.11532	0.024758	0.055083
FBXL12	-0.11532	0.024766	0.055095
DIS3L2	-0.11531	0.024777	0.055113
FCGR1C	-0.1153	0.024782	0.055118
SLC25A2	-0.11529	0.024799	0.055149
CLCN5	-0.11526	0.024831	0.055202
LPA	-0.11525	0.024846	0.055229
REPS1	-0.11525	0.024851	0.055233
ALB	-0.11524	0.024858	0.055243
NPRL3	-0.1152	0.024907	0.055345
RFX2	-0.11518	0.024938	0.055396
CDC42EP3	-0.11517	0.024946	0.055407
MYADM	-0.11515	0.024971	0.055457

IAPP	-0.11513	0.024998	0.055511
TAS2R38	-0.11511	0.025031	0.055573
ZNF687	-0.1151	0.025036	0.055577
DPY19L2P	-0.11508	0.025062	0.055628
GRID1	-0.11506	0.025094	0.055693
DSPP	-0.11504	0.025109	0.055721
SETD4	-0.11499	0.025183	0.055859
ANKRD50	-0.11495	0.025225	0.05594
PPP1R1C	-0.11491	0.025284	0.056058
HSF4	-0.1149	0.025291	0.056064
RPL23	-0.1149	0.025292	0.056064
KCNQ3	-0.11488	0.025317	0.056107
TTC31	-0.11486	0.02534	0.056145
SCN1A	-0.11486	0.025344	0.056145
APOBEC2	-0.11486	0.025346	0.056145
CALB2	-0.11484	0.025374	0.056189
CMTM5	-0.11482	0.025398	0.056229
LYVE1	-0.1148	0.025418	0.056267
PRR16	-0.1148	0.025421	0.056267
HS3ST5	-0.1148	0.025423	0.056267
LOC10027	-0.11479	0.025431	0.056278
NPHP4	-0.11476	0.025472	0.056355
CTPS2	-0.11475	0.02549	0.056384
LOC10013	-0.11473	0.025514	0.056426
APLP2	-0.11471	0.025533	0.056457
C16orf72	-0.11471	0.025535	0.056457
ADAM5P	-0.11468	0.025578	0.056524
GABRG2	-0.11468	0.025579	0.056524
ZNF581	-0.11467	0.025594	0.056549
OLFM2	-0.11464	0.025624	0.056609
CD83	-0.11462	0.025653	0.056668
TPX2	-0.11456	0.025729	0.056824
NKTR	-0.11452	0.025778	0.056918
KRT17	-0.11451	0.025796	0.056952
ASAM	-0.11447	0.025851	0.05706
PRKRA	-0.11446	0.025865	0.057086
GPR85	-0.11444	0.025892	0.057138
LIN37	-0.11441	0.025934	0.057218
POTEH	-0.1144	0.02594	0.057224
DRD1	-0.1144	0.025945	0.057229
KIAA1731	-0.11439	0.025949	0.057231
CROCC	-0.11437	0.025975	0.057282
FAM78B	-0.1143	0.026071	0.057489
CXorf42	-0.11427	0.026113	0.057574
LOC10013	-0.11426	0.026122	0.057588
SEL1L2	-0.11421	0.026186	0.057703
HOMER2	-0.11421	0.026192	0.05771
C6orf62	-0.11421	0.026196	0.057713
PRX	-0.11417	0.026238	0.057793
ATP6V1G1	-0.11413	0.026294	0.05789
ZNF793	-0.11409	0.026351	0.058003
TPST1	-0.11408	0.026358	0.058011
GLYR1	-0.11408	0.026366	0.058024
KIAA1033	-0.11404	0.026416	0.058119
ZXDA	-0.11402	0.026441	0.058161
ARID2	-0.11401	0.02646	0.058198
LOC10012	-0.114	0.026467	0.058206
AFMID	-0.114	0.026475	0.058217

DUSP18	-0.11398	0.026497	0.058254
ZNF14	-0.11398	0.0265	0.058254
KANK4	-0.11398	0.026501	0.058254
NCDN	-0.11397	0.026508	0.058261
SLU7	-0.11395	0.026536	0.058312
TMEM192	-0.11391	0.026594	0.058433
PCDHA5	-0.1139	0.026608	0.058457
CNST	-0.11388	0.026624	0.058486
SCXB	-0.11387	0.026646	0.058528
HCG4P6	-0.11386	0.026659	0.058544
HPCAL1	-0.11384	0.02668	0.058584
ZNF426	-0.11382	0.026707	0.058637
SBDS	-0.11379	0.026757	0.05874
CASP4	-0.11375	0.026809	0.058848
RSPH4A	-0.11374	0.026824	0.058874
SLC25A26	-0.11372	0.026839	0.058898
GUCA1A	-0.11372	0.026841	0.058898
GYPC	-0.11372	0.026848	0.058907
C11orf68	-0.1137	0.026867	0.058941
SENP1	-0.11369	0.026879	0.058962
WBSCR27	-0.11368	0.026899	0.058999
VENTXP7	-0.11366	0.026925	0.059049
MPV17	-0.11366	0.026929	0.05905
FAM133B	-0.11364	0.026952	0.059089
PARD3B	-0.11363	0.026964	0.05911
ECSCR	-0.11358	0.027036	0.059249
ZPLD1	-0.11358	0.027037	0.059249
CBX7	-0.11355	0.027072	0.059319
FBXO46	-0.11353	0.027096	0.059352
HUWE1	-0.11352	0.027116	0.059389
RHOBTB1	-0.1135	0.027145	0.059447
ZNF8	-0.11349	0.027161	0.059453
DHX8	-0.11348	0.027165	0.059453
RBM41	-0.11348	0.027168	0.059453
SUMF2	-0.11348	0.027168	0.059453
PSKH1	-0.11348	0.027171	0.059453
ZNF595	-0.11348	0.027171	0.059453
CDH10	-0.11348	0.027172	0.059453
FXYD7	-0.11346	0.027198	0.059503
PIAS3	-0.11345	0.027215	0.059534
HSPA12B	-0.11343	0.027239	0.059581
EML3	-0.11337	0.027319	0.059735
HIST2H2BI	-0.1133	0.027411	0.05991
STX4	-0.11327	0.027453	0.059996
KDM2A	-0.11326	0.027466	0.060017
CKMT2	-0.11326	0.027477	0.060034
BMP7	-0.11322	0.027522	0.06009
LENEP	-0.11322	0.027523	0.06009
TFDP2	-0.11322	0.02753	0.060091
TMCO2	-0.11318	0.027588	0.060211
C11orf87	-0.11316	0.027607	0.06024
C13orf26	-0.11316	0.027616	0.060252
TRAF3IP1	-0.11315	0.027624	0.060264
TLL1	-0.11314	0.027639	0.060289
CYP2D7P1	-0.11311	0.027676	0.060353
ATL1	-0.11311	0.027677	0.060353
OTUD5	-0.1131	0.027687	0.060366
ZNF274	-0.11309	0.027702	0.060387

ADAM19	-0.11309	0.027709	0.060395
TMEM116	-0.11308	0.027716	0.060397
LASS3	-0.11306	0.027754	0.060473
IGBP1	-0.11301	0.027813	0.060594
ANKRD36I	-0.11299	0.027845	0.060659
PDK4	-0.11298	0.027853	0.060662
GPR89C	-0.11295	0.027902	0.060756
PLEC	-0.11293	0.027922	0.060793
TM7SF2	-0.11293	0.027933	0.06081
LOC38963	-0.1129	0.027968	0.060872
CCL2	-0.11288	0.027995	0.060918
PRDM15	-0.11288	0.027995	0.060918
LOC40065	-0.11288	0.027999	0.06092
LOC10026	-0.11284	0.028052	0.061029
PHOX2B	-0.11283	0.028075	0.061066
CCDC85A	-0.11278	0.028144	0.061182
ZSCAN23	-0.11278	0.028144	0.061182
BEX1	-0.11272	0.028226	0.061346
CFH	-0.11271	0.02824	0.061371
HSD11B1	-0.11268	0.028272	0.061428
ADHFE1	-0.11268	0.028273	0.061428
IDI2	-0.11264	0.02834	0.061553
ARL8B	-0.11263	0.028357	0.061584
STEAP4	-0.11262	0.028367	0.061599
MGAT4C	-0.11261	0.028382	0.061617
ZNF516	-0.11261	0.028385	0.061617
WNT3	-0.11259	0.028404	0.061653
COMT	-0.11258	0.028421	0.061675
HSPC072	-0.11258	0.028424	0.061675
TM4SF1	-0.11257	0.028433	0.061681
EPSTI1	-0.11256	0.028445	0.0617
ABCA3	-0.11255	0.028469	0.06174
GCNT7	-0.1125	0.028528	0.061854
RAB39	-0.1125	0.02854	0.061873
H2BFWT	-0.11235	0.028748	0.062282
NPPB	-0.1123	0.028819	0.062423
ASB16	-0.11228	0.028854	0.062493
KRTAP5-1	-0.11223	0.028921	0.062618
RNPS1	-0.11216	0.029022	0.062815
PAR4	-0.11214	0.029057	0.062879
CHRNA1	-0.11207	0.029153	0.063078
AOC2	-0.11206	0.029172	0.063111
KRTAP10-	-0.112	0.029246	0.063265
ACMSD	-0.112	0.029259	0.063284
GABBR2	-0.11199	0.029261	0.063284
ZNF740	-0.11197	0.029295	0.063343
ATXN7L3B	-0.11195	0.029327	0.063398
CHRNB3	-0.11194	0.029341	0.063422
MAP3K3	-0.11193	0.02936	0.063458
KCNF1	-0.11192	0.029376	0.063483
TSPAN31	-0.11185	0.029472	0.063679
C6orf127	-0.11181	0.029523	0.063781
WDTC1	-0.11179	0.029561	0.063837
KIAA1875	-0.11179	0.029566	0.063837
ANP32C	-0.11178	0.029569	0.063837
LOC65365	-0.11178	0.029569	0.063837
SCN8A	-0.11178	0.029571	0.063837
SPHAR	-0.11176	0.029597	0.063878

SPG7	-0.11176	0.029605	0.063888
TGOLN2	-0.11175	0.029611	0.063894
ZNF239	-0.11172	0.029657	0.063986
RAG2	-0.11172	0.02966	0.063986
C2orf39	-0.11172	0.029666	0.063991
DCTN4	-0.11169	0.029701	0.06406
CDYL	-0.11165	0.029761	0.064183
DSCC1	-0.11158	0.029866	0.064369
ABCA6	-0.11156	0.029901	0.064429
SELM	-0.11155	0.029905	0.064432
C1orf189	-0.11153	0.029936	0.064483
FAM113A	-0.11152	0.029963	0.064527
MUC15	-0.11151	0.029968	0.064529
ARL9	-0.11147	0.030032	0.064649
GNB1L	-0.11145	0.030063	0.0647
C12orf42	-0.11144	0.030079	0.064729
CSRP1	-0.11141	0.03012	0.064796
CYP24A1	-0.11136	0.030187	0.064926
CPNE8	-0.11134	0.030226	0.064989
PRPF39	-0.11132	0.030257	0.065048
DNAJC3	-0.1113	0.030287	0.065105
SF1	-0.11122	0.030399	0.065339
PILRA	-0.11121	0.030413	0.06536
SPTA1	-0.11121	0.030416	0.06536
POM121L1	-0.11121	0.030422	0.065366
PRKCQ	-0.1112	0.030427	0.065366
TRIP12	-0.1112	0.030428	0.065366
IGFN1	-0.1112	0.030435	0.065373
MAGI2	-0.11119	0.030451	0.065402
KCNJ15	-0.11117	0.030476	0.065441
HESX1	-0.11117	0.030484	0.065452
C1orf213	-0.11111	0.03057	0.065606
PCDHA7	-0.11108	0.030622	0.065704
ATP6V1B1	-0.11106	0.030644	0.065743
SSTR2	-0.11105	0.030666	0.065784
C9orf98	-0.11103	0.030686	0.065821
LOC44120	-0.11102	0.030711	0.06586
PLEKHG5	-0.111	0.03073	0.065893
PNO1	-0.11098	0.030765	0.065961
MGAT5	-0.11097	0.030775	0.065976
NPAS4	-0.11094	0.030824	0.066067
KIAA0754	-0.11094	0.030833	0.066078
ZCCHC7	-0.11092	0.030851	0.06611
DUSP12	-0.11086	0.030945	0.066305
IL1RAP	-0.11085	0.030966	0.066339
KIAA1377	-0.11085	0.030968	0.066339
MAP3K2	-0.11082	0.031008	0.066417
CDNF	-0.11079	0.031052	0.066506
PCDHA8	-0.11078	0.031074	0.066539
C5AR1	-0.11078	0.031075	0.066539
BPGM	-0.11076	0.031107	0.066586
RFC3	-0.11075	0.031119	0.066604
TSEN2	-0.11072	0.031167	0.066694
IL1RAPL1	-0.11068	0.031217	0.066771
TMEM81	-0.11067	0.031243	0.066819
DCAF4L1	-0.11066	0.031251	0.066826
TRIM33	-0.11066	0.031253	0.066826
C22orf29	-0.11065	0.031276	0.066868

LMX1A	-0.11064	0.031279	0.066868
C1orf56	-0.11064	0.031292	0.066888
RAMP1	-0.11063	0.031308	0.066914
GALNTL2	-0.11062	0.03132	0.066934
CGB8	-0.11061	0.031326	0.06694
ANKRD31	-0.11056	0.031402	0.067081
ADCK5	-0.11054	0.031439	0.067152
CD34	-0.11048	0.03153	0.067339
ZNF432	-0.11046	0.031566	0.067409
CKAP2	-0.11043	0.03161	0.067488
DPEP1	-0.11033	0.031769	0.067776
SCARNA21	-0.11032	0.031774	0.067781
DUSP16	-0.1103	0.031807	0.067844
PIK3R1	-0.1103	0.031813	0.067848
ZNF236	-0.11026	0.031871	0.067958
TP53TG5	-0.11026	0.031875	0.067958
LYL1	-0.11024	0.03191	0.06802
HERC2P4	-0.11024	0.031911	0.06802
DGKG	-0.11022	0.031933	0.068054
GLS2	-0.11021	0.031958	0.068099
ZDHHC19	-0.1102	0.031973	0.068117
GGNBP2	-0.11017	0.032014	0.06819
ANGPT4	-0.11014	0.032056	0.068272
ZPBP	-0.11014	0.032063	0.068279
SRGAP1	-0.11004	0.032227	0.068613
LOC28331	-0.11	0.032279	0.068716
NMBR	-0.10996	0.032344	0.068842
B3GALNT1	-0.10996	0.032345	0.068842
RERGL	-0.10995	0.032359	0.068864
CD3EAP	-0.10995	0.032367	0.068874
GAPVD1	-0.10994	0.032377	0.068888
SLC39A6	-0.10994	0.032384	0.068896
TLR2	-0.10989	0.032455	0.06904
DEPDC7	-0.10986	0.032497	0.069121
EMCN	-0.10985	0.032525	0.069174
KCMF1	-0.10982	0.032563	0.069247
LOC40092	-0.10982	0.032572	0.069256
AVPR2	-0.10982	0.032574	0.069256
OPN5	-0.10977	0.032649	0.069392
LRRC37A2	-0.10972	0.032722	0.069541
DOCK3	-0.10972	0.032732	0.069543
RIMS2	-0.10969	0.032781	0.069636
C9orf131	-0.10967	0.03281	0.069675
MAGI1	-0.10964	0.032854	0.069755
PQBP1	-0.10953	0.033039	0.070116
LOC34919	-0.1095	0.033082	0.0702
MYO15A	-0.10944	0.033176	0.070377
SLC25A12	-0.1094	0.033237	0.070491
C19orf2	-0.10938	0.033268	0.070542
DIRAS2	-0.10938	0.033272	0.070543
SATL1	-0.10936	0.033305	0.070606
PCLO	-0.10934	0.033341	0.070666
NCL	-0.10934	0.033344	0.070666
AHCTF1	-0.10929	0.033413	0.070797
UNC50	-0.10929	0.033419	0.070802
BCL11B	-0.10929	0.033426	0.070808
SRPX	-0.10928	0.033429	0.070808
CALR3	-0.10927	0.03346	0.070852

SERPINA1:	-0.10925	0.033481	0.070879
TNNI2	-0.10925	0.033491	0.070893
LOC10013	-0.10923	0.033515	0.070929
PMAIP1	-0.10923	0.033515	0.070929
LOC28504	-0.10923	0.033519	0.070929
ERC1	-0.10922	0.033537	0.070961
OR2T35	-0.1092	0.03356	0.070994
ZNF615	-0.10918	0.033603	0.071077
LRCH4	-0.10911	0.033712	0.071287
DKFZp686	-0.10911	0.033715	0.071287
KCTD16	-0.10909	0.033755	0.071346
FAM190B	-0.10906	0.033789	0.071409
FAM92A3	-0.10905	0.033808	0.071428
TCF3	-0.10904	0.033831	0.071467
GXYLT2	-0.10902	0.033866	0.071535
SC65	-0.109	0.0339	0.07159
HIST1H4E	-0.10896	0.033962	0.0717
OR2T10	-0.10896	0.033968	0.071704
RUNDC2A	-0.10892	0.034034	0.071836
CD93	-0.1089	0.034066	0.071895
CLEC4M	-0.10889	0.034083	0.071922
VPS52	-0.10888	0.034086	0.071922
BRCA2	-0.10883	0.034171	0.072048
LOC65443	-0.10882	0.034199	0.072092
PLS3	-0.10876	0.034298	0.072293
FOXB1	-0.10874	0.034331	0.072354
WDR69	-0.10872	0.034363	0.072408
PIWIL4	-0.10871	0.03437	0.072413
IRGM	-0.1087	0.034393	0.072439
FAT3	-0.1087	0.034397	0.07244
KPRP	-0.10866	0.034465	0.072561
PFDN2	-0.10862	0.034519	0.072652
SPEM1	-0.10862	0.034526	0.072658
MED23	-0.10861	0.034535	0.072664
KCNMA1	-0.10861	0.034536	0.072664
KRTAP8-1	-0.1086	0.034555	0.072694
DNAJC27	-0.1086	0.034558	0.072694
C2orf3	-0.10858	0.034598	0.07277
MCM9	-0.10856	0.03463	0.072829
HIPK3	-0.10854	0.034654	0.072873
C15orf28	-0.10854	0.034659	0.072877
ZNF467	-0.10854	0.034667	0.072885
OR51E2	-0.1085	0.034723	0.072979
PHF21B	-0.10846	0.034801	0.073119
SOX18	-0.10845	0.034807	0.073125
LOC12183	-0.10844	0.034822	0.073149
SH3BGRL	-0.10838	0.034925	0.073332
LRRC8A	-0.10838	0.03493	0.073332
CCDC23	-0.10838	0.034931	0.073332
MEF2D	-0.10838	0.034933	0.073332
MGC7085	-0.10838	0.034935	0.073332
INPP5B	-0.10835	0.034983	0.073409
NIPAL4	-0.10831	0.035041	0.073516
C3orf66	-0.1083	0.035057	0.073537
MAGEF1	-0.1083	0.035059	0.073537
TRAF1	-0.10829	0.035086	0.073587
GGA3	-0.10821	0.03522	0.073828
MKL1	-0.1082	0.035233	0.073848

GP6	-0.10818	0.035271	0.07392
CHPT1	-0.10817	0.03528	0.073931
PLXND1	-0.10812	0.035372	0.074108
TMEM38B	-0.10811	0.035384	0.074125
ENO3	-0.1081	0.035403	0.07415
FAM20A	-0.1081	0.035411	0.074156
AMY2B	-0.10809	0.035414	0.074156
ZXDB	-0.10807	0.035464	0.074237
ZNF512	-0.10803	0.035518	0.074336
C5orf40	-0.10801	0.035564	0.074423
PRCD	-0.10799	0.0356	0.074486
ZNF157	-0.10799	0.035601	0.074486
CNPY3	-0.10794	0.035686	0.07464
JAZF1	-0.10793	0.035695	0.07465
GKAP1	-0.10793	0.035702	0.074654
SCAND2	-0.10789	0.03576	0.074763
WIPF1	-0.10787	0.035803	0.07483
KCNIP1	-0.10784	0.035851	0.074905
PEX2	-0.10784	0.035851	0.074905
UBN1	-0.10782	0.035878	0.074946
PCDHA1	-0.10781	0.035902	0.074989
GPR119	-0.1078	0.035927	0.075034
ZNF600	-0.10779	0.035934	0.075037
OPTC	-0.10779	0.035937	0.075037
KLHL9	-0.10777	0.035975	0.075098
GGH	-0.10776	0.035984	0.075098
CNBP	-0.10776	0.035984	0.075098
C12orf41	-0.10776	0.035985	0.075098
CDH12	-0.10775	0.036006	0.075127
MARK1	-0.10774	0.036021	0.075149
MEG3	-0.10773	0.036039	0.075179
ZNF583	-0.10768	0.036125	0.075336
CALHM2	-0.10767	0.036145	0.075347
GRTP1	-0.10767	0.036146	0.075347
ALX1	-0.10755	0.036353	0.07573
SLITRK2	-0.1075	0.036439	0.075887
TAS2R42	-0.10748	0.03647	0.075939
METT10D	-0.10748	0.036485	0.075958
DNAJC5G	-0.10745	0.036533	0.076026
PDZK1	-0.10742	0.036588	0.076124
CHRNG	-0.1074	0.036619	0.076182
OR2C1	-0.10739	0.036636	0.076209
LOC72861	-0.10738	0.036651	0.076231
KCNK3	-0.10737	0.036669	0.076261
BRD1	-0.10737	0.036675	0.076266
DUSP14	-0.10731	0.036784	0.076476
TTC39C	-0.10729	0.036806	0.076505
C1orf162	-0.10725	0.036876	0.076636
ABCG4	-0.10724	0.0369	0.076676
FAM105A	-0.10719	0.036982	0.076828
AR	-0.10716	0.037042	0.076939
TMEM128	-0.10715	0.037066	0.076981
LOC64685	-0.10714	0.037079	0.077001
ADAMTS7	-0.10712	0.037115	0.077067
PRKDC	-0.10709	0.037174	0.077182
TMEM155	-0.10707	0.037207	0.077241
HS3ST2	-0.10705	0.037238	0.077291
FERD3L	-0.10702	0.037294	0.077383

TMEM132	-0.10701	0.037301	0.077389
LOC11643	-0.10701	0.037317	0.077413
TACR1	-0.107	0.037329	0.077431
CCAR1	-0.10695	0.037425	0.077614
EDDM3A	-0.10691	0.037496	0.077727
MICAL3	-0.10685	0.037598	0.077914
RNF112	-0.10683	0.037634	0.077982
PLA2G4E	-0.10681	0.03766	0.078028
DNAH8	-0.10679	0.037709	0.078112
SLC16A6	-0.10674	0.037789	0.078271
CCDC52	-0.10673	0.037811	0.078308
PI4KAP1	-0.10667	0.037924	0.078517
RPTOR	-0.10662	0.03801	0.07867
LOC44046	-0.10657	0.038103	0.078841
TUBB2B	-0.10653	0.038182	0.078993
HIST1H2BI	-0.10651	0.038206	0.079036
FBLN1	-0.10649	0.03825	0.0791
FAM117A	-0.10649	0.038256	0.079105
ZNF641	-0.10647	0.038276	0.079139
DLL4	-0.10646	0.03831	0.079201
LOC10012	-0.1064	0.038415	0.079399
RPS28	-0.1064	0.038419	0.079399
C1D	-0.10639	0.038422	0.079399
CCDC39	-0.10639	0.038427	0.079401
KCNC2	-0.10631	0.038584	0.079701
ABT1	-0.10628	0.038633	0.079784
ANKRD20I	-0.10627	0.03866	0.079832
PAK2	-0.10626	0.038673	0.079842
SCARA3	-0.10625	0.038695	0.079879
NCRNA00	-0.10624	0.038709	0.0799
SHBG	-0.10623	0.038721	0.079917
C2orf80	-0.10621	0.038756	0.079981
ZNF469	-0.10621	0.038764	0.07999
C13orf1	-0.10617	0.038827	0.08011
PHF17	-0.10609	0.038986	0.080396
SCGB3A1	-0.10607	0.039015	0.080448
EMX2	-0.10605	0.039057	0.080526
GABARAPI	-0.10599	0.039175	0.080744
MTA1	-0.10596	0.039224	0.08083
SLC20A2	-0.10596	0.039225	0.08083
TREML2P1	-0.10594	0.039259	0.080888
BMP8A	-0.10594	0.039267	0.080892
SCAF1	-0.10592	0.039297	0.080945
ODAM	-0.10588	0.039383	0.081106
PRH1	-0.10582	0.039492	0.081314
METTL5	-0.10579	0.039538	0.081391
FAM161A	-0.10578	0.039563	0.081425
RAB41	-0.10577	0.039584	0.081451
TYRO3	-0.10577	0.039588	0.081451
DAZL	-0.10575	0.039625	0.081511
PCDHB8	-0.10572	0.039673	0.081578
RPL23AP3	-0.10572	0.039676	0.081578
ITIH2	-0.10572	0.039678	0.081578
PPIG	-0.10571	0.039696	0.081606
ZC4H2	-0.10569	0.039727	0.081662
LOC64395	-0.10567	0.039766	0.081725
FAM75A5	-0.10564	0.039835	0.081858
NCRNA00	-0.10559	0.039918	0.081994

LIPN	-0.10557	0.039956	0.082064
PMS2L1	-0.10557	0.039965	0.082068
ARMC4	-0.10556	0.03998	0.082088
KLHL3	-0.10555	0.039988	0.082096
CCDC90B	-0.10555	0.040004	0.082121
RASA2	-0.10554	0.040012	0.082128
LEPREL2	-0.10545	0.040182	0.082451
GNB5	-0.10545	0.040192	0.082454
LOC28402	-0.10539	0.040296	0.082658
RASGRP3	-0.10539	0.040299	0.082658
SIK2	-0.10536	0.040349	0.082752
C9orf173	-0.10529	0.040487	0.083008
TREML3	-0.10529	0.040491	0.083009
GNG11	-0.10527	0.040528	0.08306
EPHB1	-0.10525	0.040573	0.083142
SMOC2	-0.1052	0.040669	0.083322
YWHAZ	-0.10518	0.040708	0.083393
TWISTNB	-0.10517	0.040716	0.083401
ELOVL5	-0.10516	0.04074	0.08344
TRIM52	-0.10512	0.040815	0.083581
C1orf223	-0.10512	0.040817	0.083581
LOC44035	-0.1051	0.040864	0.083669
LRRIQ3	-0.10509	0.040884	0.083693
DISC2	-0.10506	0.040928	0.083774
CER1	-0.10501	0.041027	0.08395
RASA4P	-0.10501	0.041036	0.083951
TSPAN4	-0.10501	0.041037	0.083951
TAOK1	-0.10501	0.04104	0.083951
FBLN7	-0.105	0.041056	0.083975
AKAP2	-0.10499	0.04107	0.083987
TFPI	-0.10497	0.041108	0.084031
ZEB2	-0.10492	0.041206	0.084178
MBTPS1	-0.1049	0.041249	0.084257
PRMT8	-0.10488	0.041284	0.084318
RPL13AP6	-0.10486	0.041317	0.084362
YPEL2	-0.10486	0.041331	0.084381
TERF2	-0.10481	0.041413	0.084523
HDAC10	-0.10481	0.041413	0.084523
ZNF738	-0.1048	0.041441	0.084571
RGPD8	-0.10479	0.041461	0.084604
CXorf48	-0.10471	0.041616	0.084898
SLC5A11	-0.10471	0.041623	0.084898
SLC25A41	-0.10469	0.041656	0.08495
FAM48B2	-0.10462	0.041789	0.085203
SEMA3D	-0.10461	0.041813	0.085233
CCDC106	-0.1046	0.041834	0.085259
EIF2AK1	-0.10452	0.041982	0.085534
UTF1	-0.10449	0.042045	0.085644
LOC10012	-0.10448	0.042063	0.085663
GOLGA8F	-0.10444	0.04214	0.085804
KAT2A	-0.10444	0.042156	0.085826
LPO	-0.10443	0.042162	0.085829
MGC4580(	-0.10443	0.042166	0.085829
MYH6	-0.10442	0.042178	0.085845
NFX1	-0.10441	0.042202	0.085885
ARHGEF1(	-0.10435	0.042331	0.08614
APOM	-0.10431	0.042394	0.08625
XPC	-0.10428	0.042473	0.086366

THAP11	-0.10425	0.042526	0.086456
NINJ1	-0.10423	0.042556	0.086508
ASPRV1	-0.1042	0.042622	0.086634
ZNF180	-0.10419	0.042639	0.086646
LOC64869	-0.10418	0.042661	0.086668
C1orf198	-0.10417	0.042686	0.086701
HOXD13	-0.10416	0.042704	0.086722
HOMER1	-0.10416	0.042705	0.086722
GSG1L	-0.1041	0.042818	0.086907
FBXO9	-0.10407	0.042876	0.087016
MLF1	-0.10407	0.042892	0.08703
STAC	-0.10406	0.042905	0.087049
OVGP1	-0.10405	0.042926	0.087074
CACNB4	-0.10401	0.043014	0.087225
CLCNKB	-0.10397	0.043094	0.087361
SLC19A2	-0.10393	0.043168	0.087489
SYNGR3	-0.10392	0.043191	0.087521
RAPGEF5	-0.1039	0.043235	0.0876
BNIP3	-0.10389	0.043253	0.087629
LGI1	-0.10386	0.043307	0.087729
MSC	-0.10384	0.04334	0.087787
ZNF492	-0.10384	0.043357	0.087812
ACSM5	-0.10382	0.043396	0.087883
ALPL	-0.10381	0.043402	0.087886
C3orf43	-0.10378	0.043462	0.087999
MAB21L1	-0.10378	0.043476	0.088016
MAL2	-0.10377	0.043489	0.088035
CLDN10	-0.10372	0.043596	0.088242
OR2W1	-0.1037	0.043625	0.088284
SNRPN	-0.10368	0.043676	0.088378
MEPE	-0.10367	0.043687	0.088391
BAI2	-0.10366	0.043722	0.088451
GABPB1	-0.10363	0.043776	0.088544
FAM149B1	-0.10363	0.043786	0.088555
CDKN2A	-0.10359	0.043849	0.088646
MAP2K7	-0.10356	0.043922	0.088767
MLLT1	-0.10355	0.043936	0.088785
LOC14780	-0.10351	0.044022	0.088923
ISPD	-0.1035	0.044044	0.088959
RELA	-0.10348	0.04408	0.08902
SHC2	-0.10348	0.04409	0.089024
EN1	-0.10347	0.044113	0.08906
C1orf87	-0.10345	0.044137	0.089092
NAV1	-0.1034	0.044256	0.089305
RNF43	-0.10337	0.044303	0.08938
RSL1D1	-0.10336	0.044321	0.089409
ENTPD1	-0.10336	0.044331	0.089419
PHLDA3	-0.10332	0.044424	0.089588
SRCAP	-0.1033	0.044453	0.08963
LOC65356	-0.10326	0.044537	0.089763
KRT33B	-0.10325	0.044554	0.089788
C1orf52	-0.10321	0.044636	0.089925
SPCS2	-0.10321	0.04465	0.089945
TMEM146	-0.10319	0.04469	0.090015
CDCP2	-0.10318	0.044714	0.090046
IFNA5	-0.10317	0.044734	0.090076
STT3B	-0.10312	0.044832	0.09025
CACNB1	-0.10304	0.044995	0.090538

C1orf103	-0.10303	0.045012	0.090563
LOC40109	-0.10301	0.045062	0.090637
LOC26102	-0.10301	0.045062	0.090637
PLA2G6	-0.10297	0.045149	0.090782
NHEG1	-0.10294	0.045214	0.090905
ALS2CR11	-0.10293	0.04523	0.090928
COL21A1	-0.10292	0.045238	0.090935
RUNX3	-0.10288	0.045334	0.0911
LOC28574	-0.10287	0.045363	0.091127
ULK2	-0.10286	0.045366	0.091127
ABCC10	-0.10286	0.045373	0.091127
GRM3	-0.10285	0.045389	0.091136
WDR43	-0.10283	0.045427	0.091204
PREB	-0.10283	0.045442	0.091217
AMBRA1	-0.10281	0.045481	0.091285
TBRG1	-0.10278	0.045545	0.091386
CAV1	-0.10277	0.045568	0.091414
ZNF416	-0.10276	0.045576	0.091419
ARMC1	-0.1027	0.045709	0.091631
LYZL2	-0.10269	0.045735	0.091664
OCLM	-0.10262	0.045879	0.091914
ACCS	-0.10262	0.045883	0.091914
C3orf15	-0.10261	0.04591	0.091959
CSNK1A1F	-0.10259	0.045946	0.092007
HIST1H2BI	-0.10253	0.046067	0.092199
ERBB2IP	-0.10246	0.046217	0.092471
LAMB3	-0.10244	0.046257	0.092533
RHOB	-0.10241	0.04634	0.09268
C10orf79	-0.10239	0.04638	0.09274
AACSL	-0.10238	0.046389	0.092742
RECQL	-0.10238	0.046389	0.092742
EXOC6B	-0.10234	0.046489	0.092932
FLJ40852	-0.10231	0.046541	0.093016
IGSF1	-0.10231	0.046553	0.093017
SERPINA4	-0.10231	0.046555	0.093017
HBS1L	-0.10231	0.046555	0.093017
CYP20A1	-0.10228	0.046617	0.093121
SLC29A3	-0.10227	0.046631	0.093134
LOC34050	-0.10227	0.046633	0.093134
CABC1	-0.10227	0.046643	0.093135
DNAJC15	-0.10226	0.046659	0.093159
SUMO1	-0.1022	0.046792	0.093395
FGA	-0.10219	0.046809	0.093419
OR2H2	-0.10217	0.046858	0.09349
KRTAP4-8	-0.10213	0.046933	0.093588
RMST	-0.10213	0.046936	0.093588
TTC23L	-0.10209	0.047029	0.093727
SF3B14	-0.10208	0.047041	0.093736
SNTN	-0.10208	0.047043	0.093736
SLCO3A1	-0.10202	0.047174	0.093987
ZNF213	-0.10202	0.047178	0.093987
CPA6	-0.102	0.047214	0.094039
MYO9B	-0.10199	0.047246	0.094093
LOC10013	-0.10197	0.047286	0.094164
SLC7A3	-0.10192	0.047394	0.094369
SH3BP4	-0.10189	0.047455	0.094482
ZNF614	-0.10183	0.047598	0.094737
ZBTB4	-0.10178	0.047692	0.094916

ADIG	-0.10178	0.047705	0.09493
SLC10A3	-0.10178	0.047709	0.09493
KCNE1L	-0.10175	0.047754	0.095006
SAFB	-0.10175	0.047757	0.095006
HHAT	-0.10174	0.047789	0.09506
CLCC1	-0.10164	0.048005	0.095461
LOC10012	-0.10163	0.048037	0.095516
SESN3	-0.10161	0.048073	0.095578
CCDC120	-0.10161	0.048082	0.095586
RAB3C	-0.10156	0.048182	0.095756
SLC15A1	-0.10153	0.048247	0.095875
HSPA6	-0.10153	0.048259	0.095889
REV1	-0.1015	0.048325	0.09601
CYYR1	-0.10148	0.048366	0.096069
SF3A2	-0.10148	0.048369	0.096069
DOM3Z	-0.10142	0.048503	0.096287
SPESP1	-0.10141	0.04852	0.096312
URB1	-0.10136	0.048639	0.096519
MAPK10	-0.10133	0.048706	0.096632
MEGF11	-0.10132	0.048711	0.096632
KRTAP12-	-0.1013	0.048759	0.096709
MOCS1	-0.1013	0.048766	0.096713
GALR2	-0.10129	0.048775	0.096721
PRIMA1	-0.10129	0.048795	0.096751
SLC24A3	-0.10128	0.048805	0.096754
SNX9	-0.10127	0.048826	0.096784
C7orf47	-0.10126	0.048847	0.096804
TYW1B	-0.10126	0.048851	0.096804
MYADML2	-0.10125	0.048871	0.096814
THBS1	-0.10124	0.048906	0.096864
KSR1	-0.10118	0.049042	0.097115
DDX5	-0.10117	0.049052	0.097124
ADRBK2	-0.10116	0.049078	0.097161
TEX261	-0.10115	0.04909	0.09717
ASPDH	-0.10115	0.049095	0.09717
LOC72960	-0.10115	0.04911	0.097191
CALCR	-0.10112	0.049157	0.097274
TXNDC6	-0.10106	0.049294	0.097496
CP110	-0.10106	0.049305	0.097499
RPS19	-0.10104	0.04934	0.097557
PMS1	-0.10103	0.049372	0.097602
FSHR	-0.10101	0.049419	0.097676
C10orf113	-0.10101	0.049426	0.097677
LOC54147	-0.10099	0.049465	0.097728
AVL9	-0.10097	0.049495	0.097777
TMEM176A	-0.10097	0.049515	0.097807
PRAM1	-0.10093	0.049592	0.09794
GPN1	-0.10092	0.049613	0.097972
C3orf21	-0.10088	0.049699	0.098108
TMEM184	-0.10088	0.049702	0.098108
NKX2-5	-0.10082	0.049846	0.098372
ZCCHC3	-0.10082	0.049857	0.098384
TSPAN5	-0.10081	0.049871	0.098403
ZNF323	-0.1008	0.049899	0.098448
HMGB4	-0.10076	0.049977	0.098582
BCKDHA	0.100794	0.049907	0.098453
ERI3	0.100872	0.04973	0.098153
C2orf47	0.100895	0.049676	0.098076

TFF2	0.100905	0.049655	0.098044
DUSP10	0.100954	0.049543	0.097852
TMSB10	0.100997	0.049445	0.097698
KRT24	0.101004	0.04943	0.097677
CALM1	0.101027	0.049378	0.097604
CALB1	0.101039	0.049349	0.097567
SEC22B	0.101062	0.049299	0.097496
C15orf63	0.101109	0.049191	0.097303
NAPSB	0.10111	0.04919	0.097303
SRP72	0.101111	0.049187	0.097303
ABCA7	0.10112	0.049167	0.097285
TMPRSS4	0.101159	0.04908	0.097161
ZNF695	0.1012	0.048987	0.097016
MFSD2B	0.101242	0.048894	0.096849
SAAL1	0.101252	0.04887	0.096814
C6orf103	0.101259	0.048856	0.096804
ZNF185	0.101259	0.048856	0.096804
USP54	0.101281	0.048806	0.096754
CLEC9A	0.101316	0.048727	0.096654
PLEKHA6	0.101338	0.048677	0.096585
CDC27	0.101371	0.048603	0.096458
NHLH2	0.101399	0.048541	0.096343
DCAF6	0.101417	0.048501	0.096287
FAM27C	0.101427	0.04848	0.096261
IGFBP2	0.10144	0.04845	0.09621
PTPRC	0.101469	0.048387	0.096095
CDK16	0.101493	0.048334	0.096018
HYLS1	0.101576	0.048148	0.095698
SGOL2	0.101604	0.048087	0.095587
PTPN2	0.10166	0.047962	0.095386
DNAJC12	0.101675	0.047929	0.095328
PDF	0.101828	0.047593	0.094737
G6PC2	0.101835	0.047577	0.094715
LOC90784	0.102002	0.047214	0.094039
OGG1	0.102098	0.047004	0.093687
HSCB	0.102106	0.046986	0.093661
GLRX2	0.102113	0.046971	0.09364
TMEM218	0.102127	0.04694	0.093588
NFYC	0.102134	0.046927	0.093588
ZNF784	0.102134	0.046926	0.093588
RAB4B	0.102137	0.046919	0.093588
EMX1	0.102145	0.046902	0.093568
ABHD10	0.102177	0.046832	0.093448
LRRC18	0.102179	0.046828	0.093448
AIFM3	0.102217	0.046746	0.093313
GORASP1	0.102227	0.046725	0.093281
CCL13	0.102266	0.04664	0.093135
KIF18B	0.102277	0.046617	0.093121
KIAA1598	0.102318	0.046528	0.093
IQGAP1	0.102392	0.046369	0.092728
NAAA	0.102417	0.046317	0.092642
PRSS3	0.102457	0.04623	0.092487
TMEM59	0.102479	0.046182	0.092411
APEX2	0.102504	0.046131	0.092317
CCNK	0.102574	0.04598	0.092035
C17orf106	0.102583	0.045962	0.092007
GTF2H2	0.102584	0.045959	0.092007
TXLNA	0.102586	0.045955	0.092007

C2orf58	0.102589	0.045948	0.092007
PRR5-ARH	0.102591	0.045944	0.092007
TNFRSF18	0.102623	0.045876	0.091914
C5orf33	0.102649	0.04582	0.091816
GIPC2	0.102686	0.045742	0.091669
C21orf58	0.102693	0.045727	0.091658
SLC39A3	0.102704	0.045704	0.09163
NAA50	0.10271	0.045691	0.091612
VTI1B	0.102718	0.045674	0.091588
TMEM135	0.102745	0.045617	0.091484
IPMK	0.102762	0.045582	0.091422
C3orf67	0.102768	0.045569	0.091414
VPS26A	0.10279	0.045521	0.091346
CAPG	0.102797	0.045507	0.091328
TIMELESS	0.102828	0.045443	0.091217
TXNDC16	0.102856	0.045383	0.091133
CD180	0.10286	0.045375	0.091127
SDHAF1	0.102869	0.045356	0.091127
GCN1L1	0.102873	0.045347	0.091117
AP2S1	0.102888	0.045316	0.091073
MUC20	0.102919	0.04525	0.09095
ACSS2	0.102982	0.045118	0.090729
MSL3	0.102999	0.045083	0.090668
CSNK1A1	0.103018	0.045042	0.090615
POP4	0.103041	0.044994	0.090538
DAO	0.103101	0.044869	0.090304
REG3G	0.103114	0.044842	0.090257
CCNB3	0.103118	0.044834	0.09025
SLC22A15	0.103123	0.044824	0.090249
ANKRD45	0.103178	0.044709	0.090046
NDEL1	0.103228	0.044605	0.089873
BTNL9	0.103248	0.044562	0.089795
ATL3	0.103263	0.044532	0.089761
DEPDC4	0.103265	0.044528	0.089761
CINP	0.103291	0.044474	0.089662
CTLA4	0.103313	0.044428	0.089588
HOXB5	0.103327	0.044399	0.089548
ATP12A	0.103392	0.044266	0.089315
KLF3	0.103419	0.044209	0.089219
KPNB1	0.103422	0.044204	0.089217
LOC15038	0.103459	0.044127	0.08908
SCFD1	0.10348	0.044083	0.08902
ZDHHC6	0.103537	0.043966	0.088819
CRTAM	0.103538	0.043965	0.088819
MAP2K2	0.103544	0.043952	0.088809
GPR37L1	0.103575	0.04389	0.08871
ADH1A	0.103577	0.043885	0.08871
NAT8	0.103601	0.043836	0.088629
RABAC1	0.103616	0.043805	0.088575
TSSC1	0.103619	0.043799	0.088571
DTYMK	0.103653	0.043729	0.088457
C11orf82	0.103714	0.043605	0.088251
UBE2S	0.103928	0.043171	0.087489
NEIL3	0.103929	0.043168	0.087489
MRPL52	0.10397	0.043085	0.08735
RAF1	0.104003	0.043019	0.087225
SUMO3	0.104005	0.043015	0.087225
SLC25A6	0.104022	0.042982	0.087176

TRAIP	0.104057	0.042911	0.087052
LYRM7	0.104068	0.042889	0.08703
KLRF1	0.104116	0.042792	0.086863
NKX6-3	0.104135	0.042754	0.086795
CCDC58	0.10414	0.042744	0.086784
ITGA8	0.104146	0.042731	0.086766
SAC3D1	0.104176	0.042672	0.086682
ARHGEF37	0.104189	0.042646	0.086646
ELAC1	0.10419	0.042643	0.086646
MARS2	0.104198	0.042627	0.086635
MEFV	0.104259	0.042506	0.086425
UGT2B11	0.104288	0.042448	0.086323
MX1	0.104299	0.042426	0.086288
GSDMA	0.104307	0.04241	0.086265
RASD2	0.104312	0.042399	0.086252
IDE	0.104329	0.042367	0.086204
CRYZL1	0.104443	0.04214	0.085804
EPHA1	0.104489	0.042049	0.085644
KRT6A	0.104504	0.042019	0.0856
KRT6B	0.104562	0.041905	0.085386
AGPS	0.104583	0.041864	0.085312
DENND1B	0.104605	0.04182	0.08524
AQP10	0.10461	0.04181	0.085233
BLVRA	0.104674	0.041685	0.084998
AP1S1	0.1047	0.041633	0.084911
C16orf59	0.104707	0.04162	0.084898
ZNF536	0.104718	0.041599	0.084876
LOC10012	0.104815	0.041409	0.084523
C11orf59	0.104875	0.041292	0.084318
COBRA1	0.104877	0.041289	0.084318
GNGT2	0.104934	0.041177	0.084128
RAP2B	0.104937	0.041172	0.084126
ESR2	0.104944	0.041157	0.084104
FAM75C1	0.104947	0.041153	0.084104
CCDC88C	0.104964	0.041119	0.084044
DDX54	0.10498	0.041088	0.083998
FAIM3	0.104982	0.041083	0.083997
LOC15222	0.104987	0.041074	0.083987
PRKAG1	0.104988	0.041073	0.083987
FUBP1	0.105014	0.041022	0.083949
BCAR1	0.105037	0.040977	0.083866
METAP2	0.105094	0.040869	0.08367
AGXT2L2	0.10524	0.040586	0.08316
TRANK1	0.10527	0.040529	0.08306
ETNK1	0.105271	0.040528	0.08306
TRAPPC1	0.105326	0.040422	0.082884
ARPC5	0.105332	0.040411	0.082871
GLE1	0.10545	0.040186	0.082451
TRIM55	0.105471	0.040145	0.082384
LZIC	0.105519	0.040054	0.082206
ISG15	0.105566	0.039966	0.082068
TMEM219	0.105605	0.039891	0.081947
IFT27	0.105618	0.039868	0.081909
SH3BP2	0.105625	0.039854	0.08189
DPH2	0.105675	0.039759	0.081719
C17orf93	0.105736	0.039646	0.081536
PARK7	0.105741	0.039634	0.081522
TTF2	0.105752	0.039614	0.081497

PYCARD	0.105773	0.039576	0.081444
TNIK	0.105782	0.039558	0.081423
PPARGC1A	0.105794	0.039536	0.081391
RTKN2	0.105828	0.039473	0.081282
CPS1	0.105882	0.039371	0.08109
FAM111A	0.105941	0.039261	0.080888
DHRS7B	0.105999	0.039152	0.080706
LYPLA2	0.106002	0.039148	0.080705
ELP4	0.10612	0.038928	0.080285
CASP8	0.10615	0.038873	0.080181
PPP1R11	0.106165	0.038845	0.080131
RGS14	0.106171	0.038835	0.080118
EVI5	0.106261	0.038667	0.079838
NPY5R	0.106304	0.038589	0.079702
THRAP3	0.106365	0.038478	0.079489
SPRR2D	0.10637	0.038468	0.079477
ATP13A5	0.106431	0.038356	0.079286
ERLIN2	0.106489	0.03825	0.0791
EIF4EBP1	0.106501	0.038228	0.079072
HBB	0.106569	0.038104	0.078841
PPP2R5E	0.1066	0.038047	0.07874
ARFGAP3	0.10663	0.037994	0.078645
ALCAM	0.106638	0.037979	0.078622
QPCTL	0.106699	0.037868	0.07841
IFT122	0.106707	0.037854	0.078389
S100A11	0.106805	0.037677	0.078054
TAC3	0.106893	0.037519	0.07776
DYNLL1	0.106904	0.037499	0.077727
MRPL55	0.10693	0.037453	0.077647
WDFY4	0.106934	0.037445	0.077639
SPRED1	0.106935	0.037443	0.077639
TSR1	0.10697	0.037381	0.077531
LOC72767	0.107026	0.037281	0.077364
C1orf135	0.10703	0.037273	0.077355
SLC3A1	0.107059	0.037222	0.077266
DTX1	0.107193	0.036984	0.076828
FZD3	0.107201	0.03697	0.076814
CKLF	0.107253	0.036877	0.076636
PSMD13	0.1073	0.036795	0.076491
ROBLD3	0.107315	0.036768	0.076451
RBM3	0.107422	0.03658	0.076116
WDR45L	0.107452	0.036526	0.07602
VRK3	0.107456	0.036519	0.076013
MCM2	0.107463	0.036507	0.075995
PIK3AP1	0.107483	0.036472	0.075939
NEUROG3	0.107503	0.036438	0.075887
C9orf86	0.10754	0.036372	0.075762
GEN1	0.107591	0.036284	0.075596
TCL6	0.10761	0.036251	0.075534
FAM102B	0.107631	0.036213	0.075464
CCR4	0.107634	0.036208	0.075461
SUMF1	0.107652	0.036176	0.075403
GRM4	0.107674	0.036139	0.075347
FAM149A	0.107675	0.036137	0.075347
MDH2	0.107711	0.036074	0.075236
RCAN1	0.107712	0.036073	0.075236
CEBPD	0.107759	0.035992	0.075105
CHRNA7	0.107775	0.035963	0.075084

DCAF8L1	0.107831	0.035867	0.07493
FARSB	0.107863	0.035812	0.074839
SNRNP35	0.107868	0.035803	0.07483
RPE65	0.107874	0.035792	0.074822
GLMN	0.107925	0.035704	0.074654
CBY1	0.107942	0.035676	0.074626
PTGES3	0.107956	0.035651	0.074583
ONECUT3	0.10805	0.035491	0.074286
PDE4B	0.108067	0.035461	0.074237
FAIM	0.108092	0.035418	0.074157
DUSP2	0.108109	0.035389	0.074128
TMEM126/	0.108148	0.035324	0.074014
PIGV	0.108241	0.035164	0.073718
TRIM41	0.108264	0.035126	0.073647
STUB1	0.108265	0.035123	0.073647
VPREB1	0.108273	0.035111	0.07363
BCKDHB	0.108333	0.035008	0.073453
FADD	0.10836	0.034963	0.073375
OCIAD2	0.108372	0.034943	0.07334
AMIGO3	0.108388	0.034915	0.073329
MYBPC3	0.108407	0.034883	0.073269
CA5BP	0.108488	0.034748	0.073016
MIA3	0.108498	0.03473	0.072987
LOC10012	0.108518	0.034697	0.072933
DALRD3	0.108522	0.03469	0.072926
GFM1	0.108633	0.034504	0.072627
TSTA3	0.108649	0.034478	0.07258
RRP9	0.108668	0.034445	0.072525
RUVBL2	0.108689	0.03441	0.07246
TIMD4	0.108701	0.03439	0.072439
EOMES	0.108702	0.034389	0.072439
UTP15	0.108719	0.03436	0.072408
CCR3	0.10883	0.034175	0.072049
HSBP1L1	0.108846	0.034149	0.07201
MOCS2	0.108847	0.034148	0.07201
SELO	0.108858	0.034129	0.071982
C16orf91	0.108868	0.034114	0.071958
TMEM97	0.108877	0.034099	0.071934
FKBP4	0.108882	0.03409	0.071924
RFC2	0.108983	0.033923	0.071624
RNASEK	0.108993	0.033906	0.071596
MT1L	0.109015	0.033871	0.071537
ASAH1	0.109059	0.033798	0.071414
WDR61	0.109062	0.033794	0.071412
TNIP2	0.109094	0.03374	0.071321
NIPA1	0.109106	0.03372	0.071287
RFPL4A	0.109107	0.03372	0.071287
AAA1	0.10913	0.033682	0.071237
ADRB2	0.109206	0.033557	0.070994
ABCA13	0.109263	0.033464	0.070852
PKN3	0.109273	0.033448	0.070833
ANXA1	0.109274	0.033447	0.070833
TMEM45B	0.109308	0.033391	0.070758
CCDC112	0.109338	0.033343	0.070666
R3HCC1	0.1094	0.033241	0.070492
GCLM	0.109412	0.033223	0.07047
NSFL1C	0.109442	0.033174	0.070377
CD28	0.109445	0.033169	0.070376

EAF2	0.109528	0.033035	0.070116
CCDC18	0.109558	0.032988	0.070022
OR1D4	0.109573	0.032962	0.069976
GMEB1	0.109664	0.032817	0.069683
GSDMD	0.109672	0.032805	0.069671
DTD1	0.109684	0.032785	0.069637
TMPO	0.109716	0.032734	0.069543
TCF25	0.10972	0.032726	0.069542
MCM4	0.109784	0.032624	0.069348
SLC45A3	0.1098	0.0326	0.069303
CSF3	0.11008	0.032157	0.068471
DOC2B	0.110194	0.031977	0.068118
HMGCLL1	0.110197	0.031972	0.068117
FANCM	0.110229	0.031922	0.068038
MRPL23	0.110276	0.031848	0.067916
ALKBH5	0.110328	0.031768	0.067776
CLN6	0.110342	0.031746	0.067742
LIN7C	0.110368	0.031706	0.067664
TOMM5	0.110392	0.031668	0.067591
TIAF1	0.110393	0.031666	0.067591
LOC40237	0.110399	0.031657	0.067582
ZNF622	0.110446	0.031585	0.067442
GM2A	0.110575	0.031385	0.067052
PIP	0.110578	0.03138	0.067048
AP3S2	0.110688	0.03121	0.066764
MBLAC1	0.110703	0.031188	0.066724
PSMG3	0.110705	0.031184	0.066723
EXOC3L	0.110744	0.031125	0.06661
44628	0.110759	0.031103	0.066584
ZCCHC2	0.110762	0.031098	0.066582
SLC36A4	0.110948	0.030814	0.066052
GOLGA2B	0.111016	0.030711	0.06586
UCHL5	0.111081	0.030613	0.065692
TMED5	0.111147	0.030514	0.065493
BCKDK	0.111159	0.030495	0.06546
PDE9A	0.111163	0.030489	0.065454
IER5	0.111173	0.030475	0.065441
EGR3	0.111346	0.030215	0.064972
IRF2	0.111357	0.030199	0.064943
MAPK1IP1	0.111372	0.030177	0.064911
SLFN5	0.111418	0.030108	0.064776
CA14	0.111433	0.030085	0.064734
PMF1	0.111453	0.030056	0.064692
EFTUD1	0.11148	0.030016	0.064622
GPRC5C	0.111511	0.02997	0.064529
LRFN4	0.111525	0.029949	0.064506
SLC46A3	0.111538	0.029929	0.064476
HLA-DPA1	0.111564	0.029891	0.064415
HIST1H3C	0.111582	0.029865	0.064369
FRAT2	0.111598	0.029841	0.064329
ACOX3	0.111608	0.029826	0.064302
ZSWIM7	0.111618	0.029812	0.06428
RPS10P7	0.111624	0.029803	0.064267
DNAJC16	0.111775	0.02958	0.06385
PGM3	0.111784	0.029567	0.063837
ITGB3BP	0.111792	0.029556	0.063837
ME1	0.111855	0.029463	0.063665
NCOR1	0.11196	0.02931	0.063369

DECR1	0.111977	0.029286	0.063332
CCT2	0.112134	0.029058	0.062879
ETV6	0.112163	0.029016	0.06281
FANCE	0.112182	0.02899	0.062758
HOXB7	0.112253	0.028887	0.062549
PTPRVP	0.112255	0.028884	0.062549
RPUSD2	0.112322	0.028788	0.062363
NPTN	0.112361	0.028732	0.062255
SFRS2	0.112368	0.028722	0.06224
MT1IP	0.11241	0.028663	0.062119
CHCHD3	0.112411	0.028661	0.062119
SNX5	0.112492	0.028545	0.061878
IPO11	0.11252	0.028505	0.061811
C9orf142	0.112555	0.028457	0.061719
ZNF593	0.112574	0.028429	0.061679
INTS5	0.112583	0.028417	0.061673
CSTF2	0.112611	0.028376	0.061613
SARS2	0.112647	0.028325	0.061529
NOL4	0.112668	0.028297	0.061473
SLFN12L	0.112748	0.028184	0.061262
PRKACG	0.112804	0.028105	0.061111
COPS7A	0.112815	0.028089	0.061083
C10orf118	0.112822	0.02808	0.06107
CSNK1G3	0.112836	0.028061	0.061041
SNHG3-R(	0.112917	0.027947	0.060835
LIPT2	0.112972	0.02787	0.060694
CHST6	0.112986	0.02785	0.060662
MMP3	0.113086	0.027712	0.060396
C9orf140	0.113095	0.027699	0.060386
MRPS26	0.113116	0.02767	0.060349
RABIF	0.113171	0.027595	0.060218
AIPL1	0.11322	0.027526	0.06009
RELT	0.113232	0.02751	0.060073
LRRC28	0.113238	0.027502	0.060062
MCPH1	0.113243	0.027495	0.060054
WDR8	0.113249	0.027486	0.060041
CCND3	0.11325	0.027485	0.060041
C17orf53	0.113315	0.027395	0.059883
ARHGAP2:	0.113333	0.027371	0.059836
PXN	0.113351	0.027347	0.05979
OSBPL5	0.11341	0.027266	0.059626
SOD3	0.113415	0.027258	0.059616
LARP1B	0.113492	0.027154	0.059453
RAP1GAP2	0.113538	0.027092	0.05935
TMEM56	0.11355	0.027076	0.059321
KRT1	0.113614	0.026988	0.059155
SERPINA9	0.113656	0.026931	0.05905
C2orf69	0.11386	0.026656	0.058544
LCE1C	0.11397	0.026509	0.058261
TALDO1	0.114031	0.026428	0.058139
BHLHE41	0.114049	0.026405	0.058102
C21orf70	0.114128	0.026299	0.057895
USP1	0.114146	0.026276	0.057857
FAHD2A	0.114169	0.026246	0.057796
CDC42BPE	0.114169	0.026245	0.057796
SPEF2	0.114184	0.026226	0.057771
ANKRD9	0.114221	0.026177	0.05769
BOK	0.114222	0.026175	0.05769

EIF4A3	0.114257	0.026129	0.057597
LOC84740	0.114432	0.0259	0.057149
SLC25A19	0.114502	0.025808	0.056972
PHLPP2	0.114531	0.02577	0.056907
ARHGAP1:	0.114568	0.025722	0.056814
PIGA	0.11468	0.025577	0.056524
C1orf180	0.114701	0.025549	0.056477
PSMG1	0.11471	0.025538	0.056457
SEC22A	0.114728	0.025515	0.056426
FAM21B	0.114749	0.025487	0.056384
POLD2	0.114787	0.025439	0.056288
GPBP1L1	0.114832	0.02538	0.056196
JUND	0.114846	0.025363	0.056171
C17orf28	0.114854	0.025352	0.056153
RFFL	0.114863	0.025341	0.056145
POLE3	0.114886	0.025311	0.0561
IL10RB	0.114933	0.025252	0.055993
C14orf145	0.11498	0.025191	0.055871
RTN4R	0.115	0.025166	0.055828
NCKIPSD	0.115033	0.025124	0.05574
TMCC3	0.115039	0.025116	0.055729
MAFF	0.115125	0.025007	0.055525
NT5C2	0.11518	0.024937	0.055396
SMAD5OS	0.115197	0.024916	0.05536
DDO	0.115265	0.02483	0.055202
PDCD4	0.115279	0.024812	0.055173
SIPA1L1	0.115335	0.024741	0.055061
HIST1H3G	0.11534	0.024735	0.055057
PRKACB	0.115349	0.024724	0.055039
AADAT	0.115379	0.024686	0.05498
XAF1	0.11538	0.024685	0.05498
SLC16A5	0.115415	0.024641	0.05489
CEACAM2	0.115459	0.024586	0.05478
PPARA	0.115471	0.024571	0.054754
SNAI3	0.115477	0.024563	0.054742
LOC84989	0.115568	0.024451	0.054516
TDRD1	0.115608	0.024401	0.05441
NDUFB4	0.115621	0.024385	0.054393
SDSL	0.115714	0.024269	0.054165
NCAPH	0.115717	0.024266	0.054165
SLC38A5	0.115734	0.024245	0.054123
EIF2B3	0.115736	0.024242	0.054123
PSMB3	0.11582	0.024139	0.053936
MBNL3	0.115846	0.024107	0.053876
NR4A2	0.115889	0.024054	0.053776
CAMK1	0.116055	0.023851	0.053347
CBLN2	0.116075	0.023826	0.053296
TTC24	0.116085	0.023815	0.053277
DPH1	0.116111	0.023782	0.053211
WDR13	0.116131	0.023758	0.053163
GLIS3	0.11623	0.023639	0.052943
KIFC1	0.116244	0.023622	0.052915
CYP4Z1	0.116356	0.023486	0.052685
S100A7	0.116401	0.023432	0.052576
SAP30	0.116417	0.023413	0.052551
NPM1	0.116484	0.023333	0.052413
GIF	0.116521	0.023288	0.052336
ANO10	0.116564	0.023238	0.052228

TBX21	0.116566	0.023235	0.052228
ARSE	0.116583	0.023214	0.052188
JAK1	0.116586	0.023212	0.052188
KIF20B	0.116683	0.023096	0.051987
MNS1	0.116685	0.023094	0.051987
SPINT2	0.116709	0.023066	0.051936
FOXD4L6	0.116723	0.023049	0.05191
FAM110C	0.116754	0.023013	0.051846
RIPK1	0.116759	0.023007	0.051838
GJB2	0.116805	0.022953	0.051722
TBXAS1	0.116817	0.022939	0.051702
SELS	0.116844	0.022907	0.051642
SEMA6A	0.116938	0.022796	0.05141
OGDH	0.116946	0.022786	0.051394
LRRC6	0.116976	0.022752	0.051334
FRYL	0.116997	0.022727	0.051296
FLVCR2	0.117062	0.022651	0.051155
GOLGA1	0.117184	0.02251	0.05088
BSG	0.117198	0.022493	0.050848
FETUB	0.117213	0.022476	0.050822
SBNO2	0.117255	0.022428	0.05073
TMEM52	0.117256	0.022426	0.05073
GBP7	0.117265	0.022416	0.050721
ASCC3	0.117287	0.02239	0.050668
SLC25A44	0.117316	0.022357	0.050598
C15orf38	0.117379	0.022284	0.050475
SNX11	0.117437	0.022218	0.050353
CAPN1	0.117443	0.022211	0.050343
CENPA	0.117448	0.022206	0.050337
C22orf30	0.117452	0.022201	0.050332
KRT9	0.117454	0.022198	0.050331
ADRA2A	0.117537	0.022104	0.050157
SRP54	0.117619	0.022011	0.04998
FASN	0.117635	0.021992	0.049944
ERCC6L	0.11764	0.021987	0.04994
RMND5B	0.117647	0.021978	0.04993
PML	0.117654	0.02197	0.049922
SCCPDH	0.117675	0.021947	0.04988
ULBP3	0.117685	0.021935	0.049867
PLD1	0.117718	0.021898	0.049787
BST2	0.117722	0.021893	0.049782
GPS2	0.117724	0.021892	0.049782
SH3GLB2	0.117728	0.021886	0.049782
IL17RC	0.117803	0.021802	0.049615
FLRT3	0.11785	0.021749	0.049505
EBI3	0.117881	0.021714	0.049431
DFFB	0.117895	0.021699	0.049402
EXOSC7	0.117914	0.021678	0.049365
PDCD1	0.117942	0.021646	0.04931
EFCAB4A	0.117956	0.021631	0.049287
C22orf25	0.11797	0.021615	0.049256
PPM1G	0.117975	0.021609	0.04925
CD209	0.118016	0.021563	0.049149
FAM25A	0.118111	0.021458	0.048938
BAT5	0.118111	0.021458	0.048938
CLTA	0.11812	0.021447	0.048925
ARAP2	0.118132	0.021434	0.048901
MYO18A	0.118207	0.021351	0.048727

DBI	0.118216	0.021341	0.04871
LRTOMT	0.118275	0.021276	0.04858
TADA2B	0.118321	0.021225	0.048475
OXSR1	0.118325	0.021221	0.048475
TRAP1	0.118347	0.021196	0.04843
KIAA0141	0.118351	0.021192	0.048426
PARP14	0.118478	0.021053	0.048126
SPATA5L1	0.118499	0.02103	0.048084
LOC38933	0.118505	0.021024	0.048075
MGAT1	0.11852	0.021007	0.048043
SHMT2	0.118543	0.020982	0.047991
EPRS	0.118547	0.020977	0.047985
DLEC1	0.118559	0.020965	0.047964
KRT75	0.118596	0.020924	0.047885
ZNF648	0.118625	0.020893	0.047832
NME1	0.118699	0.020812	0.047668
ZBTB7A	0.118787	0.020718	0.047479
FAM35B	0.118818	0.020684	0.047413
RDM1	0.118824	0.020678	0.047412
PPP2R1B	0.118856	0.020643	0.047348
CHEK1	0.11897	0.020521	0.047104
ITM2A	0.119024	0.020463	0.046978
MS4A2	0.119034	0.020452	0.046963
DKFZp761	0.119084	0.0204	0.046865
TET2	0.119208	0.020268	0.046589
SNX7	0.119301	0.02017	0.046401
SLC30A4	0.119309	0.020162	0.046387
SCNN1G	0.11933	0.020139	0.046345
SUOX	0.11935	0.020118	0.046313
VDAC1	0.119359	0.020109	0.046297
LCN15	0.119362	0.020106	0.046297
PIGK	0.119392	0.020074	0.046234
C16orf61	0.119405	0.020061	0.046209
NEK2	0.119415	0.02005	0.04619
KIF2C	0.119449	0.020014	0.046127
SFMBT1	0.119485	0.019977	0.046069
RBBP7	0.119548	0.019912	0.045951
UEVLD	0.119571	0.019887	0.045899
VAT1L	0.119596	0.019861	0.045845
C1orf150	0.119618	0.019838	0.045803
OSGEP	0.119625	0.019832	0.045798
MLXIP	0.119742	0.01971	0.04556
C4orf7	0.119761	0.01969	0.045519
DEPDC5	0.119799	0.019652	0.04544
RANBP1	0.119813	0.019637	0.045411
WDR62	0.119846	0.019602	0.045354
MTL5	0.119978	0.019468	0.045085
PLCD1	0.120063	0.019381	0.044904
PID1	0.120068	0.019376	0.044898
RP2	0.120111	0.019333	0.044807
C17orf101	0.120114	0.019329	0.044804
ZNF575	0.120155	0.019288	0.044734
PITRM1	0.120205	0.019237	0.044627
CADPS2	0.120217	0.019225	0.044611
UBA5	0.120235	0.019207	0.044573
CCDC110	0.120255	0.019187	0.044532
SLC46A1	0.120348	0.019093	0.044341
TRAPPC5	0.120351	0.01909	0.044341

FAM164C	0.120358	0.019082	0.044331
CENPN	0.12039	0.019051	0.044268
IL1RL2	0.120429	0.019012	0.044186
MAPK9	0.120531	0.01891	0.043977
MIF	0.120595	0.018846	0.043843
SIGLEC12	0.120597	0.018844	0.043843
SLC25A45	0.120626	0.018816	0.043793
KLHL25	0.120626	0.018815	0.043793
GPC3	0.120649	0.018793	0.043762
C16orf42	0.120682	0.01876	0.0437
SLC14A2	0.120687	0.018755	0.043693
CEACAM1	0.120768	0.018675	0.043532
ANXA4	0.120796	0.018648	0.043474
PIK3C2B	0.120816	0.018628	0.043433
MT1X	0.120917	0.018529	0.043249
DCTN3	0.120955	0.018492	0.043171
C11orf16	0.121021	0.018427	0.043047
GBAP1	0.121121	0.01833	0.042871
TTC9	0.121234	0.018222	0.042651
PET112L	0.1213	0.018158	0.042533
ANKFY1	0.121327	0.018132	0.042487
ANKRD5	0.121328	0.018131	0.042487
CD9	0.121382	0.018079	0.042373
LTK	0.121432	0.018032	0.042271
PCTP	0.121435	0.018029	0.04227
IKZF3	0.121466	0.017998	0.042208
APH1A	0.121517	0.01795	0.042106
SDC1	0.121525	0.017943	0.042093
CHCHD5	0.121562	0.017908	0.042015
PGRMC2	0.121567	0.017902	0.042008
DPAGT1	0.121574	0.017896	0.041998
SLC25A23	0.121587	0.017883	0.041988
CENPO	0.121588	0.017883	0.041988
TCTA	0.121591	0.01788	0.041988
DEFA5	0.121602	0.017869	0.041969
MPP7	0.121605	0.017867	0.041969
SEC23IP	0.121635	0.017838	0.04191
CCDC103	0.121648	0.017826	0.041889
OBFC1	0.121651	0.017823	0.041889
TRPT1	0.121661	0.017813	0.041879
KIR3DL2	0.121726	0.017752	0.04175
TMX1	0.121777	0.017704	0.04165
ARHGAP3:	0.121807	0.017676	0.041596
WDR65	0.121839	0.017646	0.041538
GPD1	0.121855	0.017631	0.041509
MYH14	0.121879	0.017609	0.041461
DEFB1	0.121903	0.017586	0.041419
AKT1S1	0.121905	0.017584	0.041418
POLD1	0.121911	0.017578	0.041409
PIK3CG	0.121959	0.017534	0.041325
TBC1D22A	0.122021	0.017476	0.041202
PSRC1	0.122032	0.017466	0.041185
KIF26A	0.122063	0.017437	0.041121
INTS12	0.122194	0.017316	0.040873
CDRT15P	0.122197	0.017313	0.040871
KIF23	0.12236	0.017164	0.040553
KPNA6	0.122365	0.017159	0.040547
NCOA7	0.122404	0.017124	0.040487

CHCHD8	0.122418	0.01711	0.040465
SLFN13	0.122424	0.017105	0.040457
CTNNBIP1	0.122426	0.017103	0.040457
CDHR2	0.122476	0.017058	0.040399
TYMP	0.122576	0.016967	0.040213
MOV10	0.122607	0.016938	0.040159
PTGFRN	0.122675	0.016877	0.040029
HMSD	0.12268	0.016873	0.040024
MRPL2	0.122713	0.016843	0.039962
C10orf78	0.122766	0.016795	0.039868
FAM35A	0.122807	0.016759	0.03979
MINPP1	0.122833	0.016736	0.039745
SPATA5	0.122858	0.016713	0.039706
PITPNC1	0.122859	0.016712	0.039706
MTCH2	0.122878	0.016695	0.039672
SGOL1	0.122882	0.016691	0.039668
GNAI1	0.122999	0.016587	0.039464
SEC23B	0.12301	0.016577	0.039445
BTN3A1	0.123032	0.016558	0.039419
MFSD5	0.123119	0.016481	0.039258
RAB5C	0.123145	0.016458	0.039209
PSMD2	0.123161	0.016444	0.039181
KRT19	0.123206	0.016404	0.039113
APRT	0.123224	0.016389	0.03909
CCR7	0.123229	0.016384	0.039084
C17orf55	0.123315	0.016309	0.038922
CMPK2	0.123326	0.016299	0.03891
CPT1B	0.123378	0.016253	0.038814
INSIG1	0.123431	0.016207	0.038727
FKBP3	0.12345	0.016191	0.038702
VIPR1	0.123505	0.016143	0.038597
S100A13	0.123512	0.016137	0.038588
C21orf130	0.123606	0.016056	0.038426
SLC25A38	0.123714	0.015963	0.038221
NOC4L	0.123738	0.015943	0.038178
CYTH1	0.123771	0.015914	0.038122
DUT	0.123798	0.015891	0.038072
IL1RL1	0.123824	0.015869	0.038031
LRRFIP2	0.12383	0.015863	0.038024
KLRC4	0.124014	0.015707	0.037668
ANKRD37	0.124014	0.015707	0.037668
MTHFD2	0.124015	0.015706	0.037668
UPK3A	0.124036	0.015688	0.037637
IL9R	0.124099	0.015635	0.037519
C1orf57	0.124109	0.015627	0.037503
NUSAP1	0.124134	0.015606	0.037457
STXBP5	0.124182	0.015565	0.037373
ASCC2	0.124262	0.015498	0.037231
BOLA2	0.124275	0.015487	0.037213
TMEM62	0.124375	0.015404	0.037039
INO80C	0.124377	0.015403	0.037039
ADAM9	0.124378	0.015401	0.037039
SRI	0.124385	0.015396	0.037037
SPATA24	0.124421	0.015366	0.036989
C9orf93	0.12448	0.015317	0.036888
CEACAM1	0.124538	0.015269	0.036806
CAPN14	0.124556	0.015254	0.036778
MYL6	0.124631	0.015192	0.036646

KDELR2	0.124632	0.015191	0.036646
HSPBP1	0.124689	0.015145	0.036553
CLDN18	0.124851	0.015012	0.036256
THEM4	0.124854	0.01501	0.036255
CCDC42B	0.124895	0.014976	0.036191
POC1B	0.124906	0.014968	0.036175
C12orf52	0.124912	0.014963	0.036167
PEX11B	0.124921	0.014956	0.036155
FMO5	0.124932	0.014946	0.036136
GSTA1	0.124946	0.014935	0.036114
SUB1	0.124984	0.014904	0.036047
PSMA3	0.125041	0.014858	0.035949
TIPIN	0.12506	0.014843	0.035922
TRUB1	0.125079	0.014828	0.035889
MAP2K4	0.125141	0.014778	0.035778
RAB32	0.12523	0.014706	0.035626
C2orf89	0.125243	0.014696	0.035605
MFSD9	0.125272	0.014673	0.035563
PTPMT1	0.125277	0.014669	0.035557
CD97	0.125311	0.014642	0.035505
IQGAP3	0.125395	0.014575	0.035373
RUFY1	0.125433	0.014545	0.035309
C9orf117	0.125443	0.014537	0.035301
NR5A2	0.125484	0.014505	0.035237
PSAT1	0.125485	0.014504	0.035237
ATP2A2	0.125495	0.014496	0.035233
KLK10	0.125539	0.014462	0.035154
MYO3A	0.12554	0.01446	0.035154
RNF138P1	0.12558	0.014429	0.035092
NOXO1	0.125683	0.014348	0.034907
FKBPL	0.125686	0.014346	0.034906
TXNL4A	0.125698	0.014337	0.034888
FAM128B	0.125747	0.014299	0.034817
MESP1	0.125756	0.014292	0.034804
RNASE7	0.125784	0.014269	0.034754
MTX1	0.125796	0.01426	0.03474
BCL2	0.125895	0.014184	0.034579
PLA2G4F	0.125925	0.01416	0.034531
RAP1GAP	0.125933	0.014154	0.034521
PSME3	0.12594	0.014149	0.034512
DAB2IP	0.125958	0.014135	0.034481
MESDC1	0.126141	0.013994	0.034189
C15orf57	0.126163	0.013977	0.034168
EXOG	0.12628	0.013888	0.03398
ACE2	0.126303	0.01387	0.033945
UTS2R	0.126345	0.013839	0.033877
TMEM134	0.1264	0.013797	0.033795
IFI6	0.126433	0.013772	0.033742
CCNF	0.126458	0.013753	0.033702
PDIA4	0.126523	0.013705	0.033602
OR2A2	0.126553	0.013682	0.033554
TDRD7	0.126561	0.013676	0.033544
RGNEF	0.126571	0.013668	0.033529
HDAC3	0.126573	0.013667	0.033529
DCAF11	0.126575	0.013666	0.033529
C9orf7	0.126614	0.013636	0.033475
DRGX	0.126675	0.013591	0.033372
SLC35F2	0.126683	0.013585	0.033361

HNRNPM	0.126702	0.013571	0.033338
FAM27A	0.126728	0.013551	0.0333
SIGIRR	0.126741	0.013542	0.03328
RAB14	0.126827	0.013478	0.03314
LIMK2	0.126897	0.013426	0.033042
C5orf22	0.126973	0.01337	0.032921
RAN	0.127004	0.013347	0.032876
DAPK1	0.12704	0.013321	0.032824
PPP4C	0.127054	0.01331	0.032804
C12orf32	0.127169	0.013227	0.032628
ATXN10	0.127213	0.013195	0.032561
TRIM5	0.127238	0.013177	0.032524
DDA1	0.127247	0.01317	0.032516
PP14571	0.127288	0.01314	0.03245
LASP1	0.127319	0.013118	0.0324
APOA1BP	0.127364	0.013086	0.032323
GAS2L2	0.127369	0.013082	0.032319
POLR2E	0.127391	0.013066	0.032283
GUK1	0.127404	0.013057	0.032265
VPS25	0.127447	0.013026	0.032204
NUP54	0.127448	0.013025	0.032204
CXCL6	0.127452	0.013022	0.032204
DERL2	0.127474	0.013006	0.032185
NCEH1	0.127532	0.012964	0.032096
TMEM159	0.127538	0.012961	0.032095
HIST1H3H	0.127576	0.012933	0.032035
SLC10A7	0.127636	0.012891	0.031949
MYO3B	0.127729	0.012824	0.031809
RIBC2	0.127865	0.012729	0.031607
PCYOX1L	0.127875	0.012722	0.031594
MESP2	0.127877	0.01272	0.031593
CREB3	0.128017	0.012622	0.031385
HSD17B11	0.128021	0.012619	0.031382
NDOR1	0.128085	0.012575	0.031279
C10orf76	0.128093	0.012569	0.03127
PDCD10	0.128108	0.012558	0.031251
HORMAD1	0.128135	0.01254	0.031213
COPB2	0.128235	0.012471	0.031048
RNF135	0.128267	0.012448	0.031
NOL12	0.128321	0.012411	0.030919
C17orf42	0.128322	0.012411	0.030919
EPHB2	0.128352	0.01239	0.030881
TEKT5	0.128361	0.012384	0.030871
GLYCTK	0.128366	0.01238	0.030865
CRK	0.128414	0.012347	0.030804
PPM1B	0.128417	0.012345	0.030804
NAA35	0.128478	0.012303	0.030712
PWP2	0.128479	0.012303	0.030712
GBP1	0.128506	0.012284	0.030677
MRPS22	0.128517	0.012277	0.030661
MAP3K6	0.128549	0.012255	0.030618
FRMD4B	0.128582	0.012232	0.030574
TIMM44	0.128607	0.012216	0.030539
RHBDL3	0.128667	0.012175	0.030454
ZCCHC10	0.12867	0.012173	0.030454
SARS	0.128694	0.012157	0.030419
EPHA4	0.128703	0.012151	0.030408
MAN2A1	0.128736	0.012128	0.030368

KCTD14	0.128753	0.012117	0.030343
GGTLC2	0.12877	0.012106	0.030322
RASGEF1A	0.128789	0.012093	0.030293
TRAF3IP3	0.128802	0.012084	0.030276
SRP9	0.128814	0.012076	0.030259
AEN	0.128819	0.012072	0.030257
ACAA2	0.128857	0.012047	0.03021
SYAP1	0.128892	0.012024	0.030166
C1orf122	0.128912	0.01201	0.030141
C8orf74	0.12892	0.012004	0.03013
MICALL2	0.128923	0.012003	0.03013
LCE1B	0.128986	0.011961	0.030038
PPP1R16B	0.129041	0.011924	0.029966
PTCD2	0.129087	0.011893	0.0299
WDR1	0.129192	0.011824	0.029753
ARRB1	0.129204	0.011816	0.02974
NTAN1	0.129218	0.011807	0.029727
UBE2G1	0.129238	0.011794	0.029702
HMGA1	0.129286	0.011762	0.029635
YARS2	0.129311	0.011746	0.029601
DCPS	0.129365	0.01171	0.029517
TMED10	0.129377	0.011703	0.029503
ENTPD5	0.129382	0.011699	0.029502
LOC10012	0.129387	0.011696	0.029499
MT1F	0.129391	0.011693	0.029498
TTC1	0.129395	0.011691	0.029498
IFNG	0.129412	0.01168	0.029479
ARHGDIA	0.129448	0.011656	0.029428
SULT1A1	0.12948	0.011635	0.029386
NLRC3	0.129511	0.011616	0.029351
C1orf49	0.129818	0.011417	0.028898
GVIN1	0.129883	0.011376	0.028797
PRKAR2B	0.129924	0.01135	0.028738
CYBASC3	0.129944	0.011337	0.028717
RNF152	0.129984	0.011312	0.02866
NT5C	0.130007	0.011298	0.028631
ACTB	0.130067	0.011259	0.028538
DMBX1	0.130074	0.011255	0.028529
C12orf43	0.130083	0.011249	0.028519
ARTN	0.130109	0.011233	0.028488
LPAR3	0.13011	0.011232	0.028488
PRSS21	0.130142	0.011212	0.028446
DDC	0.130239	0.011151	0.028311
C1orf174	0.130279	0.011126	0.028259
LRRC45	0.130294	0.011117	0.028238
TMEM143	0.130314	0.011104	0.028213
C14orf179	0.130379	0.011064	0.028117
MAST3	0.130404	0.011048	0.028085
GFM2	0.130409	0.011045	0.028081
KIAA0319	0.130438	0.011027	0.028039
ZNF787	0.130518	0.010978	0.027939
TOMM40	0.130521	0.010976	0.027937
POU2F3	0.130534	0.010968	0.027922
SIVA1	0.130548	0.010959	0.027909
SLAIN2	0.130559	0.010953	0.027899
NPY1R	0.130577	0.010941	0.027881
GPRIN3	0.130611	0.01092	0.027835
CXCR2	0.130643	0.010901	0.027789

PKDREJ	0.130654	0.010894	0.027775
FASLG	0.13067	0.010884	0.027758
C1orf89	0.130671	0.010884	0.027758
ATF3	0.130684	0.010875	0.027742
VPS18	0.130693	0.01087	0.027732
C5orf56	0.130722	0.010853	0.027698
TMED2	0.130729	0.010848	0.027695
TRAPPC4	0.130749	0.010836	0.027673
BSCL2	0.130909	0.010738	0.027463
FAM110A	0.130972	0.010701	0.027374
PPY	0.131047	0.010655	0.027274
SLC5A8	0.131074	0.010639	0.027241
APTX	0.131088	0.010631	0.027223
LYRM5	0.131123	0.010609	0.027175
SRPRB	0.131131	0.010605	0.02717
RPS6KA4	0.131158	0.010588	0.027135
RPUSD3	0.131162	0.010586	0.027134
C1orf31	0.13124	0.010539	0.027024
TKTL2	0.131265	0.010525	0.02699
ARF1	0.131331	0.010485	0.026899
TWF1	0.131338	0.010481	0.026892
ALDH1A2	0.131388	0.010452	0.026834
RAB18	0.131392	0.01045	0.026834
DPP4	0.131397	0.010446	0.02683
NF2	0.131419	0.010433	0.0268
GIYD2	0.131424	0.010431	0.026797
ABCD4	0.131431	0.010426	0.026789
ECT2	0.131499	0.010387	0.026698
MTMR10	0.131503	0.010384	0.026694
TRIM68	0.131586	0.010335	0.026586
MGC1291(	0.131598	0.010328	0.026575
ATP5J2	0.131618	0.010317	0.026549
PDXK	0.131647	0.0103	0.026522
IL16	0.131665	0.010289	0.026499
PTER	0.131866	0.010172	0.026262
PPP1CA	0.131877	0.010166	0.026251
CSRNP1	0.131922	0.010141	0.026191
IFITM1	0.131986	0.010103	0.026105
NCK1	0.132021	0.010083	0.026059
EML2	0.132205	0.009979	0.025804
LIF	0.132209	0.009976	0.025804
SLC25A34	0.132231	0.009964	0.025775
ZMYND10	0.13233	0.009908	0.025639
CYC1	0.132336	0.009904	0.025634
TMEM139	0.132471	0.009829	0.025472
REPS2	0.132519	0.009802	0.025411
ADCK2	0.132525	0.009798	0.025406
C22orf41	0.132641	0.009733	0.025248
KIAA1244	0.132661	0.009722	0.025223
MICB	0.132672	0.009716	0.02521
CYB5D1	0.132725	0.009687	0.025154
IKZF2	0.13282	0.009635	0.025034
FASTK	0.132875	0.009605	0.024966
WHSC1	0.132878	0.009603	0.024965
MCM10	0.132929	0.009575	0.024915
HIRA	0.132936	0.009571	0.024908
TSHR	0.133083	0.009491	0.024723
AIMP2	0.133114	0.009475	0.024686

GK5	0.133118	0.009472	0.024683
SMC5	0.133128	0.009466	0.024671
CYB5A	0.133169	0.009444	0.02462
FAM114A1	0.133195	0.009431	0.024588
NELL1	0.133229	0.009412	0.024544
FAM92B	0.133234	0.009409	0.024544
CLOCK	0.133255	0.009398	0.024525
SLC16A14	0.133303	0.009372	0.024465
COL29A1	0.133377	0.009332	0.024367
DEPDC1B	0.133385	0.009328	0.024359
CCDC21	0.133391	0.009325	0.024355
CHRFAM7	0.133408	0.009316	0.024339
SMPD3	0.133469	0.009283	0.024261
C5orf43	0.133557	0.009237	0.024149
FCHO2	0.133592	0.009218	0.02412
C10orf95	0.133685	0.009169	0.023998
HMGN1	0.133714	0.009154	0.023968
USP14	0.133763	0.009128	0.023912
C15orf61	0.133795	0.009112	0.023875
RAB1A	0.133842	0.009087	0.023817
POLR3K	0.133865	0.009075	0.023799
STEAP2	0.133923	0.009045	0.023725
SCARA5	0.133963	0.009024	0.023674
UMPS	0.133983	0.009014	0.023653
PDHB	0.134031	0.008989	0.023601
GINS2	0.134092	0.008957	0.023539
FHOD1	0.134109	0.008948	0.023526
PSMB6	0.134145	0.00893	0.023483
C4orf29	0.13417	0.008917	0.023452
CAPN12	0.134189	0.008907	0.023436
SERBP1	0.134273	0.008864	0.023338
RPL26L1	0.134299	0.008851	0.023312
ADIPOR2	0.134299	0.008851	0.023312
PPP2R5B	0.134309	0.008846	0.023308
BRCA1	0.134322	0.008839	0.023294
UPP1	0.134343	0.008829	0.023273
KIF2A	0.134349	0.008825	0.023267
SHISA5	0.134375	0.008813	0.023236
WDHD1	0.134379	0.00881	0.023233
USF1	0.134385	0.008807	0.02323
COL4A5	0.134387	0.008806	0.02323
GLRA4	0.13439	0.008805	0.02323
CLLU1	0.1344	0.008799	0.02322
STK17B	0.134436	0.008782	0.023185
EXOSC9	0.134469	0.008764	0.023146
SELT	0.134497	0.00875	0.023118
MRPL21	0.134589	0.008704	0.023019
FARSA	0.134641	0.008678	0.022958
KIF22	0.134681	0.008658	0.022913
CD52	0.134687	0.008655	0.022909
CCR9	0.134716	0.00864	0.02288
BIN2	0.134741	0.008628	0.022853
TMEM132I	0.134818	0.00859	0.022761
CHUK	0.13482	0.008589	0.022761
FAM82A2	0.134835	0.008581	0.022751
LOC10012	0.134862	0.008568	0.022724
CDC7	0.134927	0.008536	0.022641
LTA	0.134937	0.008531	0.022632

CCDC47	0.13494	0.00853	0.022631
SETD8	0.13499	0.008505	0.022573
C10orf54	0.134998	0.008501	0.022567
AMH	0.135	0.0085	0.022567
NCAPG	0.135093	0.008454	0.022458
BBS7	0.135139	0.008432	0.022402
INTS10	0.135141	0.008431	0.022402
COMMD3	0.13518	0.008412	0.022355
XPO7	0.135206	0.008399	0.022323
C16orf7	0.135228	0.008388	0.022301
CIAPIN1	0.135245	0.00838	0.022285
NT5DC1	0.135337	0.008335	0.022187
USP15	0.135341	0.008333	0.022185
LYPD6	0.135444	0.008284	0.02207
ASTL	0.135513	0.008251	0.021994
PIN1	0.135629	0.008195	0.021853
AGPAT4	0.135649	0.008186	0.021833
PAK6	0.135651	0.008185	0.021833
NAMPT	0.135655	0.008183	0.021831
GPR137	0.135671	0.008175	0.021817
KIAA0284	0.135672	0.008175	0.021817
ISCA2	0.135689	0.008166	0.021802
C9orf156	0.135734	0.008145	0.021748
NDUFS5	0.135801	0.008114	0.021678
A2LD1	0.135805	0.008112	0.021676
HAR1A	0.135862	0.008085	0.02162
PHF5A	0.136036	0.008003	0.021424
RASAL3	0.136051	0.007996	0.021408
ZBTB32	0.136086	0.00798	0.021382
MTMR14	0.136094	0.007976	0.021375
PPP2R4	0.136121	0.007964	0.021347
BRIP1	0.136165	0.007943	0.021309
NHP2	0.136174	0.007939	0.021301
RCHY1	0.136188	0.007933	0.021291
PRKX	0.136205	0.007925	0.021274
GATAD2A	0.13621	0.007923	0.021272
TMEM180	0.13633	0.007867	0.021137
IFI44	0.136339	0.007863	0.021128
SPZ1	0.136342	0.007862	0.021128
TTC7A	0.136354	0.007856	0.021119
CKAP2L	0.136392	0.007839	0.021077
KIF18A	0.136394	0.007838	0.021077
METTL4	0.136463	0.007806	0.021011
TPSB2	0.136528	0.007777	0.020933
ITGA6	0.136542	0.00777	0.020919
BUB1B	0.136576	0.007755	0.020883
LOC28600	0.136608	0.007741	0.020851
KLF1	0.136736	0.007683	0.020706
SIP1	0.136746	0.007678	0.020696
NUMB	0.136822	0.007644	0.020624
LONP1	0.136836	0.007638	0.020613
APOL3	0.136889	0.007614	0.020558
NDUFB7	0.136905	0.007607	0.020543
SALL1	0.136926	0.007598	0.020524
FAM151B	0.136935	0.007594	0.020516
APOBEC3I	0.136962	0.007582	0.020492
PITPNA	0.137046	0.007544	0.020397
ARL6IP5	0.13705	0.007542	0.020394

DDRGK1	0.137121	0.007512	0.020319
LOC10013	0.13715	0.007499	0.020287
PARL	0.137154	0.007497	0.020284
COPZ1	0.137172	0.007489	0.020275
ACADVL	0.137182	0.007485	0.020265
HMBS	0.137211	0.007472	0.020234
CNTD2	0.137227	0.007465	0.020217
FAM135B	0.137229	0.007464	0.020217
DSG2	0.137277	0.007443	0.020172
LSM2	0.137295	0.007435	0.020156
DOPEY2	0.137311	0.007428	0.02014
PA2G4	0.137343	0.007414	0.020104
ITGAL	0.137361	0.007406	0.020092
ENPP6	0.137416	0.007382	0.020035
GP2	0.137422	0.00738	0.020031
FAM173A	0.137484	0.007353	0.019964
TMEM111	0.137498	0.007347	0.019951
NIPSNAP3	0.137506	0.007343	0.019949
KLRK1	0.137614	0.007297	0.019844
DAD1	0.137656	0.007279	0.0198
TMEM161I	0.137731	0.007247	0.019721
SRXN1	0.137798	0.007218	0.019651
RFX6	0.137818	0.00721	0.019631
PDIA3P	0.137828	0.007205	0.019622
HAR1B	0.138044	0.007114	0.019408
HOXB6	0.138058	0.007108	0.019398
PPP2R5A	0.138061	0.007107	0.019396
NOC2L	0.138165	0.007064	0.019294
TAF9	0.138172	0.007061	0.019289
LOC10013	0.138178	0.007058	0.019285
DUSP6	0.138189	0.007054	0.019275
ATP2B1	0.13822	0.007041	0.019253
LYSMD4	0.138222	0.00704	0.019253
TAF5	0.138231	0.007036	0.019249
MED19	0.138235	0.007035	0.019247
VASP	0.138239	0.007033	0.019247
PDIA5	0.138324	0.006997	0.019153
DCTN6	0.138378	0.006975	0.019098
DHX58	0.138409	0.006962	0.019072
SEMA3B	0.138473	0.006936	0.019007
COMMD9	0.138484	0.006932	0.018997
GATA6	0.138551	0.006904	0.018932
NARS	0.138648	0.006865	0.018832
BAIAP2L2	0.138655	0.006862	0.018829
GCDH	0.138698	0.006845	0.018794
FUT10	0.138798	0.006804	0.018691
PLEKHH1	0.13884	0.006787	0.018649
MYO7B	0.138884	0.006769	0.018606
CNIH	0.138893	0.006766	0.018599
SLC26A3	0.138901	0.006763	0.018595
TRHDE	0.138925	0.006753	0.018571
MAD2L1BI	0.138953	0.006742	0.018548
TAOK3	0.139107	0.00668	0.018402
DGKQ	0.139139	0.006667	0.018377
C15orf55	0.139205	0.006641	0.01831
INTS9	0.139231	0.006631	0.01829
LSM4	0.139249	0.006624	0.018272
WBSCR26	0.139273	0.006615	0.018251

BTBD3	0.139289	0.006608	0.018236
DERA	0.139341	0.006588	0.018185
MMP1	0.139343	0.006587	0.018185
ANKRD52	0.139368	0.006577	0.018166
C3orf57	0.139438	0.00655	0.018101
RASD1	0.139449	0.006546	0.018092
CCDC6	0.139508	0.006523	0.018033
SLC35B3	0.139526	0.006516	0.018023
TPSAB1	0.139567	0.0065	0.017985
CCND2	0.139582	0.006494	0.017971
C19orf21	0.139648	0.006469	0.017903
MRPS30	0.139701	0.006448	0.017852
GRIN2D	0.13973	0.006437	0.017831
FAM160A2	0.139772	0.006421	0.017793
HSDL2	0.139785	0.006416	0.017783
VNN2	0.139906	0.00637	0.017676
GRHPR	0.139971	0.006345	0.01762
TIMM22	0.139973	0.006344	0.01762
APOBEC4	0.139997	0.006335	0.017599
CEACAM2	0.140034	0.006321	0.017574
ARMC7	0.140125	0.006287	0.017493
KIAA1967	0.140137	0.006282	0.017483
CEACAM5	0.140195	0.006261	0.017433
PTTG3P	0.14024	0.006244	0.01739
PPP1R13B	0.140254	0.006239	0.017381
COX4I1	0.140405	0.006183	0.017242
CD58	0.140465	0.00616	0.017182
FAAH	0.140495	0.006149	0.017156
CHP2	0.140604	0.006109	0.017049
PPARG	0.140742	0.006059	0.016927
KAZALD1	0.140789	0.006042	0.016889
PRPSAP1	0.140798	0.006039	0.016883
MRPL51	0.140914	0.005997	0.016782
DNAH2	0.140994	0.005968	0.016713
ZNF831	0.141037	0.005952	0.016677
TMEM167A	0.14106	0.005944	0.016659
PPIH	0.141086	0.005935	0.016635
SCRN2	0.141126	0.005921	0.0166
LOC10012	0.141149	0.005912	0.016581
FOXP1	0.141152	0.005912	0.016581
PTPN22	0.141196	0.005896	0.016547
TMED7	0.141206	0.005892	0.016539
CPOX	0.141231	0.005883	0.016517
ADAT3	0.141328	0.005849	0.016439
SIRT7	0.141334	0.005847	0.016435
LOC64493	0.141393	0.005826	0.016381
IMP3	0.1414	0.005824	0.016377
HNRNPC	0.141451	0.005806	0.016336
ZNF367	0.141456	0.005804	0.016334
NPRL2	0.141491	0.005792	0.016306
PLCD3	0.141512	0.005785	0.01629
C4orf27	0.14156	0.005768	0.016254
C17orf78	0.14159	0.005757	0.016227
ISOC1	0.141596	0.005756	0.016224
COX11	0.141624	0.005746	0.016203
ILVBL	0.141712	0.005715	0.016134
IER3IP1	0.141715	0.005714	0.016133
PIGN	0.141742	0.005705	0.016111

METRNL	0.141749	0.005702	0.016109
C15orf23	0.141802	0.005684	0.016065
ACOXL	0.141828	0.005675	0.016042
STK39	0.14185	0.005668	0.016027
MOBKL1B	0.141944	0.005636	0.01594
C1orf161	0.14197	0.005627	0.01592
ALKBH7	0.141998	0.005617	0.0159
NCAPH2	0.142011	0.005613	0.01589
SLC13A2	0.142036	0.005604	0.015873
LOC72999	0.142037	0.005604	0.015873
JAGN1	0.142055	0.005598	0.015863
KCNE3	0.142073	0.005592	0.015853
ACTL8	0.142079	0.00559	0.015849
CYP1A1	0.142082	0.005589	0.015849
PDHA1	0.142094	0.005585	0.015842
GSTO2	0.142138	0.00557	0.015811
SLC13A1	0.142139	0.005569	0.015811
HSPA14	0.142174	0.005558	0.015783
ATP5L	0.142222	0.005542	0.01574
ALG14	0.142257	0.00553	0.015716
MID1	0.142287	0.00552	0.01569
NTF3	0.142334	0.005504	0.015658
LRFN3	0.142347	0.0055	0.015648
FBXO22	0.142378	0.00549	0.015626
KIF20A	0.142468	0.00546	0.015545
BOLA1	0.142496	0.00545	0.015525
AURKB	0.142689	0.005387	0.015367
TCL1A	0.142824	0.005343	0.015259
ZADH2	0.142913	0.005314	0.015183
OR7E91P	0.142925	0.00531	0.015174
STK17A	0.143017	0.00528	0.0151
CD274	0.143084	0.005259	0.015047
HPR	0.143106	0.005252	0.015029
GGT1	0.143152	0.005237	0.014989
EIF2AK3	0.14317	0.005232	0.01498
NEO1	0.143175	0.00523	0.014977
WSB2	0.143332	0.00518	0.014847
CFC1B	0.143472	0.005136	0.014738
SLC27A5	0.143483	0.005133	0.014731
CDKN2C	0.143497	0.005128	0.014722
PPP4R1	0.143545	0.005113	0.014683
HDC	0.143571	0.005105	0.014664
ARL6IP1	0.14359	0.005099	0.014654
SEC61B	0.143666	0.005076	0.014596
BTLA	0.143676	0.005073	0.014591
NDUFS8	0.143731	0.005055	0.014549
OVOL2	0.143807	0.005032	0.014486
TOB2	0.143847	0.00502	0.014454
CTAGE1	0.143859	0.005016	0.014446
LOC44095	0.14387	0.005013	0.014438
LMNA	0.14398	0.004979	0.014352
UGT2A3	0.143997	0.004974	0.014341
SRL	0.144001	0.004973	0.01434
C12orf5	0.14403	0.004964	0.014319
CD22	0.144051	0.004958	0.014306
CD48	0.144055	0.004956	0.014305
DTL	0.144062	0.004954	0.0143
TAP2	0.144074	0.004951	0.014295

PGM2	0.144134	0.004933	0.014251
COPG	0.14414	0.004931	0.014247
NHLRC2	0.144147	0.004929	0.014243
C12orf49	0.144167	0.004922	0.014227
GCNT4	0.14423	0.004903	0.014181
FANCC	0.144248	0.004898	0.014167
ERP44	0.144319	0.004877	0.014123
HNF1B	0.144327	0.004874	0.014118
C12orf4	0.144335	0.004872	0.014112
GPRIN2	0.144342	0.00487	0.014109
DMRTA2	0.144366	0.004863	0.014092
COQ7	0.144418	0.004847	0.014055
IDO2	0.144469	0.004832	0.014016
C10orf125	0.144516	0.004818	0.013979
FAM166B	0.144694	0.004766	0.013846
MPPE1	0.144709	0.004761	0.013839
KBTBD12	0.144773	0.004743	0.013786
CD38	0.144898	0.004706	0.013705
MFN2	0.144949	0.004692	0.013672
YWHAE	0.144967	0.004686	0.013661
MGLL	0.144989	0.00468	0.013648
C5orf25	0.145012	0.004674	0.013634
C4orf36	0.145072	0.004656	0.013589
FCER2	0.145084	0.004653	0.013583
YEATS4	0.145097	0.004649	0.013574
ITGB4	0.145098	0.004649	0.013574
MND1	0.145251	0.004605	0.013458
THAP4	0.14526	0.004602	0.013456
PDCD6IP	0.145282	0.004596	0.01344
JDP2	0.145288	0.004594	0.013436
TRIM38	0.145309	0.004589	0.013421
KLK7	0.145392	0.004565	0.013361
RAP1A	0.145401	0.004563	0.013355
DDX39	0.145469	0.004543	0.013311
HSD17B4	0.145501	0.004535	0.013291
IKZF1	0.145548	0.004521	0.013256
HELLS	0.145552	0.00452	0.013254
ALDH9A1	0.145558	0.004519	0.013252
TTC13	0.145716	0.004475	0.013136
PRMT5	0.145748	0.004466	0.013113
HLA-DMB	0.14576	0.004462	0.013106
LHPP	0.145764	0.004461	0.013105
CRAT	0.145812	0.004448	0.013073
ZNF774	0.145848	0.004438	0.013053
TSPO2	0.145904	0.004423	0.013013
IL1F7	0.14594	0.004413	0.012988
PMVK	0.145948	0.004411	0.012984
EHD1	0.145982	0.004401	0.01296
RNF167	0.146018	0.004392	0.012933
GMFB	0.146024	0.00439	0.012932
MORN1	0.146085	0.004373	0.012891
YIPF1	0.146093	0.004371	0.012887
TUBA4A	0.146108	0.004367	0.012877
TBC1D12	0.146116	0.004365	0.012873
SPRR2A	0.1462	0.004343	0.012809
USP5	0.146223	0.004336	0.012792
ATG10	0.146249	0.004329	0.012774
SLAMF1	0.146255	0.004328	0.012773

CISD2	0.146263	0.004325	0.012768
MEP1B	0.146325	0.004309	0.012724
SEC24D	0.146456	0.004274	0.012629
CCBL2	0.146518	0.004258	0.012594
BMP5	0.146547	0.00425	0.012575
PSMB2	0.14659	0.004239	0.012547
SLC16A13	0.146679	0.004215	0.01249
CPA3	0.146716	0.004205	0.012462
STBD1	0.14672	0.004204	0.012462
CTNNAL1	0.146722	0.004204	0.012462
TUBB8	0.146723	0.004204	0.012462
NUP37	0.146755	0.004195	0.012449
CALM2	0.146791	0.004186	0.012431
COG7	0.146815	0.00418	0.012417
NCR2	0.14688	0.004163	0.012369
EFNB1	0.146948	0.004145	0.012322
MIF4GD	0.14699	0.004134	0.012296
CITED1	0.146995	0.004133	0.012295
CENPW	0.14701	0.004129	0.012285
CATSPERG	0.147169	0.004088	0.012181
CRIP1	0.147171	0.004088	0.012181
MON1A	0.147262	0.004065	0.012116
PPP6C	0.147271	0.004062	0.012113
NLRP7	0.147571	0.003987	0.011925
CCL22	0.147594	0.003981	0.011912
MRPS15	0.14762	0.003975	0.011895
DDAH1	0.147654	0.003966	0.011876
ITGA3	0.147785	0.003934	0.011785
SLC9A7	0.147802	0.00393	0.011775
WDR18	0.147872	0.003913	0.011733
ATG7	0.147989	0.003884	0.011654
C1orf141	0.14809	0.003859	0.011591
C9orf23	0.148128	0.00385	0.011565
CHRNA5	0.148179	0.003838	0.011533
TSPAN14	0.148201	0.003833	0.011519
HLA-DRB1	0.14847	0.003768	0.01134
H2AFX	0.148486	0.003765	0.01133
MYOF	0.148537	0.003753	0.011301
PTP4A1	0.148544	0.003751	0.011298
ANKRD54	0.148555	0.003748	0.011292
MRPL38	0.148612	0.003735	0.011256
IL2RB	0.148637	0.003729	0.011242
SYTL5	0.148791	0.003693	0.011145
ASTN2	0.148848	0.00368	0.011111
HOMEZ	0.148851	0.003679	0.01111
IQCG	0.148856	0.003678	0.011109
RDH11	0.148867	0.003675	0.011102
GPR82	0.148873	0.003674	0.0111
TSTD1	0.148893	0.003669	0.011089
KIAA1524	0.14891	0.003665	0.011079
GTF3C6	0.148936	0.003659	0.011062
NMU	0.14896	0.003654	0.011049
CD164L2	0.149022	0.00364	0.011008
NUP85	0.149033	0.003637	0.011001
SLC18A1	0.149056	0.003632	0.010989
CHAC1	0.149141	0.003612	0.010937
OAS3	0.149378	0.003559	0.010788
COG2	0.149434	0.003546	0.010761

ITPKA	0.14956	0.003518	0.0107
ARPC3	0.149609	0.003507	0.01067
NIPSNAP1	0.149631	0.003502	0.010658
TARP	0.149662	0.003495	0.010641
SASH3	0.149706	0.003486	0.010621
PRDX2	0.149724	0.003482	0.010611
COPE	0.149798	0.003465	0.010566
MAT2B	0.149865	0.003451	0.010529
POLH	0.149961	0.00343	0.010472
ERAP1	0.149983	0.003425	0.010461
CFP	0.149987	0.003424	0.010461
ZFYVE27	0.150063	0.003408	0.010419
LEO1	0.150159	0.003387	0.010366
MAL	0.150162	0.003386	0.010366
COX6B1	0.150174	0.003384	0.010363
HBXIP	0.150214	0.003375	0.01034
FAM158A	0.150253	0.003367	0.010317
ZFYVE19	0.150272	0.003363	0.01031
NDUFS1	0.15028	0.003361	0.010306
CAPZA1	0.150287	0.003359	0.010305
GIMAP2	0.1505	0.003314	0.010179
NSMCE4A	0.150549	0.003304	0.01015
IL15	0.150567	0.0033	0.01014
FBXL15	0.150569	0.0033	0.01014
RNF34	0.150581	0.003297	0.010134
RASAL1	0.150619	0.003289	0.010116
FAM108B1	0.150642	0.003284	0.010103
PSMD1	0.150673	0.003278	0.010086
C1orf226	0.15068	0.003276	0.010083
SAMD1	0.150762	0.003259	0.010032
EVI5L	0.150946	0.003221	0.00993
DOLK	0.151005	0.003209	0.009897
FAM98C	0.151014	0.003207	0.009894
POMT1	0.151078	0.003194	0.009857
C10orf46	0.151101	0.003189	0.009846
WARS2	0.151159	0.003178	0.009817
GPR114	0.151192	0.003171	0.0098
ARX	0.151246	0.00316	0.009771
PHF23	0.151311	0.003147	0.009735
AQP5	0.151333	0.003143	0.009726
ZMPSTE24	0.151363	0.003137	0.00971
HLA-DRB5	0.151404	0.003128	0.009689
LTB	0.151435	0.003122	0.009676
MCCC1	0.151459	0.003117	0.009664
PPIP5K1	0.151503	0.003109	0.00964
NASP	0.151565	0.003096	0.009607
GALNT1	0.151622	0.003085	0.00958
DOT1L	0.151733	0.003063	0.009518
ELF3	0.151737	0.003062	0.009517
ZFYVE28	0.151784	0.003053	0.009493
SREBF2	0.151793	0.003051	0.00949
MRPL11	0.151802	0.00305	0.009487
AGTRAP	0.151855	0.003039	0.009463
LINS1	0.151985	0.003014	0.009389
CLNK	0.152043	0.003003	0.009357
ENTPD7	0.152142	0.002984	0.009304
SPRR3	0.152175	0.002977	0.009288
G3BP1	0.152245	0.002964	0.009252

ZBTB8A	0.152342	0.002946	0.009205
KIF13A	0.152382	0.002938	0.009183
PARP12	0.152425	0.00293	0.009162
FOXN1	0.152463	0.002923	0.009142
SLC44A2	0.152482	0.002919	0.009133
PRSS8	0.152493	0.002917	0.009127
LGTN	0.152498	0.002916	0.009127
CTDP1	0.152562	0.002904	0.009091
ZNF169	0.152563	0.002904	0.009091
MAPK6	0.152614	0.002895	0.009065
SAT1	0.152675	0.002883	0.009035
NDUFS6	0.15271	0.002877	0.009016
SLC18A2	0.152713	0.002876	0.009016
GANC	0.152782	0.002863	0.008982
STAP2	0.152854	0.00285	0.008946
PTGR1	0.15289	0.002843	0.008926
SLC9A3R1	0.152918	0.002838	0.008913
GPRC5D	0.15292	0.002838	0.008913
C20orf29	0.152985	0.002826	0.00888
RANGAP1	0.153154	0.002795	0.008792
CDC42EP4	0.153156	0.002795	0.008792
TXN	0.153192	0.002788	0.008775
CLECL1	0.1532	0.002787	0.008772
GPR3	0.153221	0.002783	0.008764
SGCB	0.153241	0.00278	0.008754
BANK1	0.153317	0.002766	0.008716
MAPKAPK	0.153334	0.002763	0.008708
C17orf61	0.153339	0.002762	0.008707
MAP2K1	0.153379	0.002755	0.00869
FAM171A1	0.153411	0.002749	0.008675
TNFRSF1A	0.153418	0.002748	0.008672
COX5B	0.153443	0.002744	0.008661
MED31	0.153453	0.002742	0.008658
C1orf115	0.15348	0.002737	0.008645
CDC25B	0.153572	0.002721	0.008602
LPAR5	0.153583	0.002719	0.008598
D4S234E	0.153618	0.002713	0.008581
MFN1	0.153618	0.002713	0.008581
HSH2D	0.153678	0.002702	0.008552
EARS2	0.153711	0.002696	0.008537
ATP6V0E1	0.153769	0.002686	0.008509
CCNE1	0.153825	0.002676	0.008484
KIAA1671	0.153832	0.002675	0.008482
TCN2	0.153863	0.00267	0.008466
PEX11G	0.153911	0.002661	0.008442
LSM6	0.153928	0.002659	0.008435
CYP3A5	0.153954	0.002654	0.008423
LOC15019	0.153975	0.00265	0.008413
KCNK7	0.153994	0.002647	0.008404
ARHGAP9	0.154012	0.002644	0.008398
VDR	0.154113	0.002627	0.008347
CHMP7	0.15416	0.002619	0.008324
TMEM106	0.154178	0.002616	0.008315
MKI67	0.154179	0.002616	0.008315
C16orf54	0.154187	0.002614	0.008315
SPON1	0.154205	0.002611	0.008309
TRPV6	0.154243	0.002605	0.008291
OTUB2	0.154306	0.002594	0.008262

MCM6	0.154382	0.002581	0.008223
CNFN	0.154395	0.002579	0.008218
C17orf73	0.154452	0.002569	0.008192
MLYCD	0.154486	0.002564	0.008175
NDUFA8	0.154496	0.002562	0.008171
PLEKHJ1	0.154539	0.002555	0.008157
AUH	0.154659	0.002535	0.0081
MRPL24	0.154663	0.002534	0.0081
CTSC	0.154677	0.002532	0.008095
TXNL1	0.154746	0.002521	0.008067
EIF3I	0.154851	0.002503	0.008022
SMC2	0.154853	0.002503	0.008022
SYTL4	0.154858	0.002502	0.008021
RASSF6	0.154921	0.002492	0.007991
HARS2	0.154982	0.002482	0.007961
MTG1	0.154995	0.00248	0.007956
TPH1	0.155003	0.002479	0.007954
TRIM11	0.15501	0.002478	0.007952
THOC4	0.155097	0.002464	0.007912
NMI	0.155161	0.002453	0.007885
MPV17L2	0.155163	0.002453	0.007885
RFX4	0.155205	0.002446	0.007867
CENPE	0.155233	0.002442	0.007856
ELMO3	0.155266	0.002436	0.007841
GTPBP8	0.155356	0.002422	0.0078
SCYL2	0.155403	0.002415	0.007779
C15orf29	0.155415	0.002413	0.007774
PPYR1	0.15542	0.002412	0.007773
MSGN1	0.155433	0.00241	0.007769
MYH13	0.155531	0.002395	0.007723
FAM73B	0.155565	0.002389	0.007709
NEK4	0.155585	0.002386	0.007701
CEP76	0.155611	0.002382	0.007691
ARFIP1	0.155633	0.002379	0.007683
RARRES1	0.155653	0.002375	0.007675
ANKRD22	0.155654	0.002375	0.007675
NKG7	0.155705	0.002367	0.007653
ESRP2	0.155742	0.002362	0.00764
BEND4	0.155792	0.002354	0.007622
PPAP2C	0.155839	0.002347	0.0076
EPB41L4A	0.155847	0.002345	0.007599
ANXA7	0.155917	0.002335	0.007568
ATP6V0B	0.155983	0.002325	0.007537
TOX2	0.156082	0.002309	0.007493
FAM98A	0.156217	0.002289	0.007439
LYSMD3	0.15622	0.002289	0.007439
LEPROTL1	0.156284	0.002279	0.007413
SLC29A2	0.156391	0.002263	0.007369
COX16	0.156488	0.002249	0.007329
HLA-H	0.156525	0.002243	0.007315
DAZAP1	0.156785	0.002205	0.0072
POLE2	0.156908	0.002187	0.007148
SEC16A	0.156967	0.002178	0.007127
ZBTB7B	0.15698	0.002177	0.007122
KLB	0.157056	0.002166	0.007091
PPP2CB	0.157076	0.002163	0.007083
KIF13B	0.15708	0.002162	0.007083
GMPR	0.157099	0.00216	0.007077

THOC3	0.157135	0.002155	0.007062
KIAA0748	0.157151	0.002152	0.007056
MDH1	0.157164	0.00215	0.007051
SMAP2	0.157165	0.00215	0.007051
SOCS6	0.157175	0.002149	0.007048
C19orf51	0.157258	0.002137	0.007013
XKR9	0.157284	0.002133	0.007002
FAM64A	0.157303	0.002131	0.006995
AQP3	0.157333	0.002126	0.006988
SYTL2	0.157341	0.002125	0.006986
ZDHHC14	0.157355	0.002123	0.006982
CXCL13	0.1575	0.002103	0.006928
RNASE1	0.157511	0.002102	0.006925
ARPC5L	0.15757	0.002093	0.006902
CHMP6	0.157625	0.002086	0.006881
MFSD11	0.15767	0.002079	0.006866
NUDT5	0.157806	0.002061	0.006809
CXCR2P1	0.157831	0.002057	0.006802
LOC10027	0.157831	0.002057	0.006802
STRADB	0.157833	0.002057	0.006802
MRPL46	0.157866	0.002053	0.00679
RACGAP1	0.157894	0.002049	0.006779
HMG20B	0.157917	0.002046	0.00677
NNT	0.157919	0.002045	0.00677
RNASET2	0.157969	0.002039	0.00675
WFDC2	0.158018	0.002032	0.006729
KIAA1211	0.158023	0.002031	0.006728
C11orf9	0.158024	0.002031	0.006728
SH2D1A	0.158034	0.00203	0.006726
FARS2	0.15804	0.002029	0.006725
SEH1L	0.158051	0.002027	0.006721
TIGIT	0.158068	0.002025	0.006716
PRPF18	0.158078	0.002024	0.006713
CSK	0.15811	0.00202	0.006701
CHTF8	0.15816	0.002013	0.006683
FAM111B	0.158225	0.002004	0.006658
LOC10014	0.158249	0.002001	0.006649
TRIM22	0.15827	0.001998	0.006641
C9orf70	0.158301	0.001994	0.00663
FCRL3	0.158327	0.001991	0.006621
C22orf32	0.15847	0.001972	0.006571
IL19	0.158521	0.001965	0.006553
RGL4	0.158525	0.001964	0.006552
GLB1L2	0.158546	0.001962	0.006545
EXO1	0.158605	0.001954	0.006522
RASEF	0.158636	0.00195	0.00651
HSPA8	0.158686	0.001943	0.006493
BIRC5	0.15869	0.001943	0.006492
GINS4	0.158701	0.001941	0.006488
GPT2	0.158773	0.001932	0.006463
CTNNA1	0.158777	0.001932	0.006462
ADAMTS1	0.158794	0.001929	0.006456
AP1S3	0.158896	0.001916	0.006419
KIF24	0.158916	0.001914	0.006411
LIN9	0.158925	0.001913	0.006408
NAT8B	0.158965	0.001908	0.006392
ENTPD4	0.158967	0.001907	0.006392
MUC6	0.158968	0.001907	0.006392

ACBD3	0.158986	0.001905	0.006387
TRIM3	0.159018	0.001901	0.006375
KCNK10	0.159087	0.001892	0.006348
C14orf50	0.159097	0.001891	0.006345
OXA1L	0.159138	0.001886	0.006332
CYCSP52	0.159177	0.001881	0.006317
ZSWIM6	0.159254	0.001871	0.006288
UGT1A6	0.159276	0.001868	0.00628
LAG3	0.159277	0.001868	0.00628
STIM2	0.159317	0.001863	0.006268
HYAL3	0.159323	0.001862	0.006267
PLBD1	0.159353	0.001859	0.006257
MLST8	0.159497	0.001841	0.006204
KLHDC7A	0.15954	0.001836	0.006188
GPR174	0.159559	0.001833	0.006183
TPRN	0.159565	0.001832	0.006182
PKM2	0.159574	0.001831	0.00618
ABP1	0.159582	0.00183	0.006177
MED8	0.159662	0.001821	0.006149
TIMM10	0.159662	0.00182	0.006149
FAM189A2	0.159704	0.001815	0.006136
UBR7	0.159715	0.001814	0.006134
RIOK3	0.159717	0.001814	0.006134
ETV7	0.159743	0.001811	0.006125
HIST1H2A	0.159812	0.001802	0.006101
BDKRB2	0.15982	0.001801	0.006099
CLSPN	0.159836	0.001799	0.006095
DUSP23	0.159847	0.001798	0.006091
ECSIT	0.159892	0.001793	0.006074
HPCA	0.159932	0.001788	0.006061
PAX5	0.159967	0.001784	0.006048
ALG12	0.160035	0.001775	0.006022
SHD	0.160142	0.001763	0.005985
NDUFB10	0.160247	0.00175	0.005945
MVP	0.160323	0.001741	0.005919
FANCD2	0.160486	0.001722	0.005863
GAR1	0.160623	0.001707	0.005815
CRIP3	0.160675	0.001701	0.0058
RAB5B	0.160741	0.001693	0.00578
C2CD2L	0.160754	0.001691	0.005777
SUV39H2	0.160788	0.001688	0.005766
AMMECR1	0.160814	0.001685	0.005759
SCG5	0.160818	0.001684	0.005759
MRPL47	0.160887	0.001676	0.005737
TUSC2	0.16098	0.001666	0.005705
KRTCAP3	0.160996	0.001664	0.005699
TMED3	0.160997	0.001664	0.005699
MMP12	0.160998	0.001664	0.005699
IARS2	0.161007	0.001663	0.005699
PROM1	0.161094	0.001653	0.005672
NFKB1	0.161127	0.001649	0.00566
ALDH3A1	0.161156	0.001646	0.00565
DNAJC9	0.161167	0.001645	0.005647
C11orf83	0.161192	0.001642	0.005642
VNN3	0.161195	0.001642	0.005642
RAD51AP1	0.161198	0.001641	0.005641
SFT2D1	0.16131	0.001629	0.005604
SERTAD1	0.161442	0.001615	0.005561

TRIOBP	0.161465	0.001612	0.005554
HLA-DRB6	0.161507	0.001607	0.00554
IDO1	0.161614	0.001596	0.005505
PLK4	0.161634	0.001594	0.005499
NQO1	0.16165	0.001592	0.005495
PLAC4	0.16167	0.00159	0.005491
CD63	0.16167	0.00159	0.005491
GSTM2P1	0.161705	0.001586	0.00548
BCCIP	0.161739	0.001582	0.00547
ANXA10	0.161742	0.001582	0.00547
MRPL42	0.161785	0.001577	0.005457
C19orf23	0.161845	0.001571	0.005438
C6orf211	0.161851	0.00157	0.005437
RPS6KB2	0.161891	0.001566	0.005423
RHOG	0.161956	0.001559	0.005405
IMMT	0.162012	0.001553	0.005387
HIF1A	0.162014	0.001553	0.005387
ATG4D	0.162152	0.001539	0.005342
CD69	0.162249	0.001528	0.005307
RALBP1	0.16233	0.00152	0.005281
C16orf62	0.162445	0.001508	0.005247
COQ6	0.162532	0.001499	0.005219
DGCR11	0.162789	0.001473	0.005135
PYHIN1	0.162865	0.001465	0.005113
TMEM107	0.162877	0.001464	0.005112
LOC12784	0.162884	0.001464	0.00511
ANXA2	0.162913	0.001461	0.005102
ARL1	0.162914	0.001461	0.005102
CYB5D2	0.162977	0.001454	0.005082
CD1D	0.162996	0.001452	0.005077
FCRL1	0.16304	0.001448	0.005063
PPP2R2A	0.163058	0.001446	0.005057
CAND1	0.163061	0.001446	0.005057
SSTR3	0.163064	0.001446	0.005057
C22orf13	0.163086	0.001444	0.005052
AKT1	0.163174	0.001435	0.005026
SPC24	0.163174	0.001435	0.005026
CAMTA2	0.163226	0.00143	0.00501
SH2D3A	0.163444	0.001409	0.004943
MKKS	0.163474	0.001406	0.004936
STOML2	0.163488	0.001404	0.004933
MYB	0.163499	0.001403	0.00493
IL27RA	0.163502	0.001403	0.00493
GINS3	0.163507	0.001402	0.00493
AGPAT2	0.163518	0.001401	0.004927
BCR	0.163636	0.00139	0.004896
DDX60L	0.16365	0.001389	0.004892
RNF148	0.163708	0.001383	0.004874
C1orf186	0.163741	0.00138	0.004865
MRPS34	0.163753	0.001379	0.004862
SEC11A	0.163763	0.001378	0.004859
NFIB	0.163812	0.001373	0.004844
C19orf70	0.163867	0.001368	0.004827
TRIAP1	0.163874	0.001368	0.004827
FEZF1	0.16415	0.001342	0.004743
SPRR1A	0.164162	0.001341	0.00474
TMCO4	0.164208	0.001337	0.004728
GLTPD1	0.164217	0.001336	0.004726

C3orf37	0.164229	0.001335	0.004723
APITD1	0.164375	0.001321	0.00468
C18orf32	0.16438	0.001321	0.00468
VIT	0.164499	0.00131	0.004645
EXD3	0.164528	0.001307	0.004636
TMEM144	0.164549	0.001305	0.004631
SLC39A7	0.16456	0.001304	0.004629
PDCD6	0.164565	0.001304	0.004629
TRIM25	0.164571	0.001303	0.004628
PYGB	0.164597	0.001301	0.004621
PPP2R2D	0.164599	0.001301	0.004621
MRPL14	0.1646	0.001301	0.004621
NDUFB3	0.16462	0.001299	0.004616
RAD54L	0.164645	0.001297	0.00461
GLRX3	0.164658	0.001296	0.004607
PDIA3	0.16467	0.001295	0.004603
RAB35	0.164752	0.001287	0.004582
CDK1	0.164858	0.001278	0.004552
VPS4B	0.164868	0.001277	0.004551
ATP6V1D	0.164919	0.001272	0.004536
VPREB3	0.16506	0.00126	0.004497
TIFAB	0.165134	0.001254	0.004476
MUC12	0.165181	0.00125	0.004463
MYH15	0.165239	0.001245	0.004447
WFDC12	0.16524	0.001245	0.004447
YWHAH	0.165266	0.001242	0.004441
ACAA1	0.165266	0.001242	0.004441
AMICA1	0.165358	0.001234	0.004417
ANXA2P2	0.165379	0.001233	0.004412
CMTM6	0.165407	0.00123	0.004405
DMC1	0.165457	0.001226	0.004391
ACAD9	0.165519	0.001221	0.004376
C21orf45	0.165567	0.001217	0.004364
GTSE1	0.16559	0.001215	0.004359
FABP7	0.165591	0.001215	0.004359
C17orf59	0.1656	0.001214	0.004358
LAD1	0.165648	0.00121	0.004348
KRT7	0.165742	0.001202	0.004323
RIN1	0.165801	0.001197	0.004308
SC4MOL	0.165848	0.001193	0.004296
TAS2R60	0.165848	0.001193	0.004296
SNAP29	0.165877	0.001191	0.004289
TMEM71	0.165894	0.001189	0.004285
ARF3	0.165902	0.001189	0.004283
VDAC3	0.165939	0.001186	0.004274
CFB	0.165958	0.001184	0.00427
SNX24	0.165964	0.001184	0.004269
C4orf14	0.166046	0.001177	0.004247
ZZEF1	0.166067	0.001175	0.004242
AKNA	0.166088	0.001173	0.004236
PEF1	0.166115	0.001171	0.00423
RASGEF1B	0.166164	0.001167	0.004217
B9D1	0.16622	0.001163	0.004204
XCR1	0.166236	0.001161	0.0042
C6orf153	0.166323	0.001154	0.00418
C1orf159	0.166326	0.001154	0.004179
LINGO4	0.166443	0.001145	0.004149
PAG1	0.16647	0.001143	0.004143

RAB1B	0.166488	0.001141	0.004139
SIDT1	0.166536	0.001137	0.004128
TDP2	0.166537	0.001137	0.004128
HPGD	0.166575	0.001134	0.004119
CTBP2	0.166634	0.00113	0.004103
ICT1	0.16665	0.001128	0.0041
CLEC17A	0.166674	0.001126	0.004095
C6orf182	0.166723	0.001123	0.004083
UNG	0.166748	0.001121	0.004078
C14orf119	0.166835	0.001114	0.004057
NDC80	0.166849	0.001113	0.004054
C9orf123	0.166887	0.00111	0.004044
CDRT1	0.16694	0.001106	0.00403
GCGR	0.166999	0.001101	0.004019
ZNF165	0.167053	0.001097	0.004006
CYB561	0.167071	0.001096	0.004002
PIGZ	0.167166	0.001088	0.003978
FAM109A	0.167329	0.001076	0.003938
SERPINB4	0.167394	0.001071	0.003924
NDUFA7	0.167484	0.001064	0.003904
PBLD	0.167603	0.001055	0.003876
TOE1	0.167672	0.00105	0.00386
CR2	0.167772	0.001043	0.003837
TLR10	0.167813	0.00104	0.003828
CPB1	0.167838	0.001038	0.003822
RAB9A	0.167912	0.001033	0.003805
MELK	0.167993	0.001027	0.003785
LAMA1	0.167995	0.001027	0.003785
C17orf91	0.16801	0.001026	0.003782
LRP10	0.16811	0.001019	0.003759
PEX26	0.168207	0.001012	0.003735
NRG1	0.168232	0.00101	0.003731
FOXM1	0.168258	0.001008	0.003725
ICOS	0.168388	0.000999	0.003694
PCYT1A	0.168425	0.000996	0.003686
LYSMD2	0.16844	0.000995	0.003683
GPD2	0.168443	0.000995	0.003683
ESRRA	0.168471	0.000993	0.003677
THOP1	0.168479	0.000992	0.003676
SDHC	0.168495	0.000991	0.003673
VTI1A	0.168525	0.000989	0.003665
LMNB1	0.168642	0.000981	0.003638
IFI16	0.168678	0.000979	0.00363
C12orf62	0.16868	0.000978	0.00363
FANCG	0.168731	0.000975	0.003618
LMF2	0.168802	0.00097	0.003605
DSCR3	0.168817	0.000969	0.003601
B4GALT7	0.168823	0.000969	0.003601
VAPA	0.168945	0.00096	0.003572
DAG1	0.168951	0.00096	0.003571
SAMD9	0.169	0.000957	0.00356
PCK2	0.169022	0.000955	0.003557
MBD2	0.169059	0.000953	0.003549
IRF8	0.169214	0.000942	0.003513
PDZD8	0.16925	0.00094	0.003506
CKB	0.169286	0.000937	0.003498
BUB3	0.169299	0.000937	0.003496
MT1A	0.169311	0.000936	0.003495

SLC25A17	0.169324	0.000935	0.003492
HLA-DRA	0.169383	0.000931	0.003479
C14orf166	0.169431	0.000928	0.003468
CCL5	0.169468	0.000925	0.00346
CLCN3	0.169491	0.000924	0.003456
LOC38895	0.169526	0.000922	0.003449
GRHL1	0.169589	0.000917	0.003436
GNG13	0.169629	0.000915	0.003427
PAQR5	0.169721	0.000909	0.003409
RAB8A	0.169815	0.000903	0.00339
AIFM1	0.169842	0.000901	0.003385
SLC25A28	0.169846	0.000901	0.003384
C8orf4	0.16986	0.0009	0.003382
LLGL2	0.169874	0.000899	0.003381
EVI2B	0.169919	0.000896	0.003373
PANK4	0.169932	0.000895	0.00337
MSRA	0.169967	0.000893	0.003364
DULLARD	0.169969	0.000893	0.003364
JAG1	0.16998	0.000892	0.003362
NUCB2	0.170015	0.00089	0.003356
SERPINB8	0.170028	0.000889	0.003355
RUNDC1	0.170046	0.000888	0.003352
IFI35	0.17005	0.000888	0.003351
PFKFB4	0.170143	0.000882	0.003333
PHGR1	0.170209	0.000878	0.003318
KLK11	0.170387	0.000867	0.003281
CCDC129	0.170416	0.000865	0.003275
C9orf129	0.170579	0.000855	0.003243
FBXW10	0.170599	0.000854	0.003239
PPP1R1B	0.170654	0.000851	0.003228
SKA2	0.170676	0.000849	0.003223
USP2	0.170759	0.000844	0.003207
VAMP8	0.170836	0.00084	0.003192
SULT1A3	0.170846	0.000839	0.00319
RBX1	0.170941	0.000833	0.003172
MED16	0.170949	0.000833	0.003171
ERO1L	0.17097	0.000832	0.003167
RIPK3	0.171057	0.000826	0.003151
HCP5	0.171057	0.000826	0.003151
TRUB2	0.171088	0.000825	0.003145
EPHX2	0.171091	0.000824	0.003145
C5orf20	0.171095	0.000824	0.003145
LY6D	0.171154	0.000821	0.003133
E2F8	0.171186	0.000819	0.003126
SLA2	0.171274	0.000814	0.003109
TRMU	0.171326	0.000811	0.003099
CCDC160	0.171421	0.000805	0.003082
IL18RAP	0.171494	0.000801	0.003068
CALR	0.17155	0.000798	0.003057
UROS	0.171609	0.000794	0.003046
CECR5	0.171686	0.00079	0.003032
KLHL18	0.171735	0.000787	0.003024
ZMYND15	0.171784	0.000784	0.003015
RNF138	0.171876	0.000779	0.002999
IDH1	0.172004	0.000772	0.002975
STXBP2	0.172012	0.000772	0.002974
OCEL1	0.172014	0.000772	0.002974
CFTR	0.17206	0.000769	0.002965

TFAP2A	0.172083	0.000768	0.002961
PVRL4	0.172135	0.000765	0.002952
LGMN	0.172141	0.000765	0.002951
CD79B	0.172238	0.000759	0.002932
MRPL17	0.172324	0.000755	0.002916
SLC25A16	0.172327	0.000754	0.002916
DPP3	0.172368	0.000752	0.002909
ARSH	0.172395	0.000751	0.002905
ZWILCH	0.172448	0.000748	0.002896
TSPAN17	0.172468	0.000747	0.002893
C5orf52	0.172481	0.000746	0.002891
CCDC25	0.172496	0.000745	0.002888
IQCD	0.172648	0.000737	0.002859
SPRR1B	0.172829	0.000728	0.002825
C12orf26	0.172874	0.000725	0.002819
NCOA4	0.172886	0.000725	0.002818
KLK12	0.173161	0.00071	0.002767
RNF126	0.173177	0.00071	0.002765
RPP25	0.173195	0.000709	0.002762
LOC28562	0.173259	0.000705	0.002752
ACTR1A	0.173382	0.000699	0.002731
B4GALNT3	0.173413	0.000697	0.002725
STX19	0.173414	0.000697	0.002725
GPR18	0.173475	0.000694	0.002716
FAM25B	0.173484	0.000694	0.002714
CD8A	0.173495	0.000693	0.002713
SERAC1	0.173638	0.000686	0.002689
ALG1	0.17369	0.000684	0.00268
HAVCR1	0.173719	0.000682	0.002675
SUPV3L1	0.173753	0.000681	0.00267
TIMM8B	0.173769	0.00068	0.002668
CPM	0.173927	0.000672	0.002641
MRPS12	0.173979	0.000669	0.002634
SLPI	0.173991	0.000669	0.002632
TIPARP	0.174004	0.000668	0.002631
MRPS25	0.174009	0.000668	0.002631
PITPNM3	0.174102	0.000663	0.002617
SLC25A46	0.174106	0.000663	0.002617
SERPINB6	0.174131	0.000662	0.002613
FASTKD5	0.174191	0.000659	0.002603
OSTF1	0.174212	0.000658	0.002599
LOC81691	0.174253	0.000656	0.002593
GNLY	0.174264	0.000656	0.002592
CXCL16	0.174313	0.000653	0.002585
RNF4	0.174401	0.000649	0.002571
CYP51A1	0.17444	0.000647	0.002565
UGT1A9	0.174536	0.000643	0.002549
DSC2	0.174559	0.000642	0.002546
MTA2	0.174568	0.000641	0.002545
ACTG1	0.174601	0.00064	0.002539
WARS	0.174615	0.000639	0.002538
NRGN	0.174632	0.000638	0.002535
MUC13	0.174645	0.000638	0.002534
MRPL43	0.174647	0.000638	0.002534
PRC1	0.174708	0.000635	0.002524
PSMG2	0.174914	0.000625	0.002492
MRPL34	0.174914	0.000625	0.002492
EFHC2	0.17504	0.00062	0.002473

SPAG5	0.175045	0.000619	0.002472
MTF1	0.175163	0.000614	0.002455
CD5	0.175211	0.000612	0.002448
TMOD3	0.17524	0.000611	0.002444
FAM66D	0.175246	0.00061	0.002443
ANKRD13(	0.17527	0.000609	0.002439
ANKRD32	0.175294	0.000608	0.002436
SSU72	0.175315	0.000607	0.002434
ANKHD1-I	0.175324	0.000607	0.002433
CENPI	0.175382	0.000604	0.002423
C12orf57	0.175443	0.000602	0.002415
GGA1	0.175454	0.000601	0.002413
WDR25	0.175477	0.0006	0.00241
AGPAT5	0.175543	0.000597	0.0024
COQ5	0.17561	0.000594	0.00239
CLDN14	0.175663	0.000592	0.002382
FGFR1OP	0.175703	0.00059	0.002375
POF1B	0.175731	0.000589	0.002372
PICK1	0.175782	0.000587	0.002363
SLC6A19	0.175846	0.000584	0.002354
MCL1	0.175859	0.000584	0.002353
SLC25A20	0.175986	0.000578	0.002332
JKAMP	0.176	0.000578	0.00233
STARD10	0.176028	0.000576	0.002326
DUSP27	0.176043	0.000576	0.002324
HLA-DOB	0.176108	0.000573	0.002315
SNRPD1	0.176123	0.000572	0.002314
VSIG8	0.176191	0.000569	0.002304
G3BP2	0.176244	0.000567	0.002296
KCNA3	0.176283	0.000566	0.002291
LUZP1	0.176329	0.000564	0.002284
SMAD1	0.176387	0.000561	0.002277
IQCK	0.176414	0.00056	0.002273
POLDIP2	0.176475	0.000558	0.002264
OXCT1	0.176502	0.000557	0.00226
CBLC	0.176507	0.000556	0.00226
ENHO	0.176533	0.000555	0.002257
SLC25A39	0.176573	0.000554	0.002252
SLC30A9	0.176669	0.00055	0.002238
C1orf172	0.176669	0.00055	0.002238
SP140L	0.176682	0.000549	0.002237
CFL1	0.176688	0.000549	0.002236
ATG4C	0.176707	0.000548	0.002234
NUP62CL	0.176716	0.000548	0.002233
TOR3A	0.176722	0.000548	0.002232
FAM40B	0.176734	0.000547	0.002231
CCDC72	0.176741	0.000547	0.002231
PYCR1	0.176771	0.000546	0.002227
SENP8	0.176939	0.000539	0.002202
FSIP1	0.176999	0.000537	0.002193
C3orf75	0.177056	0.000534	0.002185
TXNDC11	0.177165	0.00053	0.002169
GRIN3A	0.177188	0.000529	0.002166
ODF3B	0.177208	0.000528	0.002164
PCYT2	0.17722	0.000528	0.002163
FBXO4	0.177236	0.000527	0.002161
SLC25A4	0.177292	0.000525	0.002152
UBXN8	0.177298	0.000525	0.002152

C20orf7	0.177318	0.000524	0.002149
FAM13A	0.177356	0.000523	0.002144
HIP1R	0.177521	0.000516	0.00212
POLR3B	0.177542	0.000515	0.002117
CCNG1	0.177548	0.000515	0.002117
IL22	0.177558	0.000515	0.002116
MYL12A	0.177604	0.000513	0.002109
NAPG	0.177763	0.000507	0.002087
C17orf99	0.177978	0.000499	0.002056
STRA13	0.178051	0.000496	0.002046
KALRN	0.178078	0.000495	0.002044
ALG2	0.178346	0.000486	0.002007
ACVR1C	0.178412	0.000483	0.001999
HTR3C	0.178427	0.000483	0.001997
ELAC2	0.178486	0.000481	0.001989
SLC2A13	0.178519	0.000479	0.001985
ACAP1	0.178546	0.000478	0.001982
FECH	0.178554	0.000478	0.001981
RASSF1	0.178558	0.000478	0.001981
HSPA9	0.178571	0.000478	0.00198
ROPN1	0.178609	0.000476	0.001975
WDR44	0.178786	0.00047	0.001954
KCNQ4	0.178803	0.000469	0.001951
HEXB	0.178819	0.000469	0.00195
TIGD2	0.179007	0.000462	0.001925
UHRF1	0.179066	0.00046	0.001918
SLC25A1	0.179071	0.00046	0.001917
IPCEF1	0.179132	0.000458	0.001909
TCF19	0.179164	0.000457	0.001906
C11orf51	0.179281	0.000453	0.001892
SLC22A18	0.179384	0.000449	0.001879
RABGGTA	0.17943	0.000448	0.001873
SFXN4	0.179492	0.000446	0.001866
EIF4A1	0.179592	0.000443	0.001853
FPGS	0.179631	0.000441	0.001849
FOXI1	0.179677	0.00044	0.001844
TOMM70A	0.179779	0.000436	0.001833
ATP13A2	0.179791	0.000436	0.001831
CD163L1	0.179818	0.000435	0.001829
HMGN2	0.179824	0.000435	0.001829
GBA	0.179902	0.000432	0.00182
DDB2	0.179935	0.000431	0.001816
EIF2S1	0.179957	0.000431	0.001814
ENSA	0.179972	0.00043	0.001812
FABP5	0.180039	0.000428	0.001804
CAPNS1	0.180078	0.000427	0.001799
NOP16	0.180149	0.000424	0.00179
WWP2	0.180251	0.000421	0.001777
TRIM16L	0.180373	0.000417	0.001763
ARHGAP1	0.18043	0.000416	0.001756
RFC5	0.180509	0.000413	0.001747
KIAA1468	0.180582	0.000411	0.001738
ST14	0.180591	0.000411	0.001737
ABHD11	0.180695	0.000407	0.001725
MAGI3	0.180696	0.000407	0.001725
RG9MTD1	0.180708	0.000407	0.001724
ABLIM1	0.180734	0.000406	0.001721
HMGCS2	0.18078	0.000405	0.001715

BAG3	0.180786	0.000405	0.001715
CD44	0.180818	0.000404	0.001712
WWC1	0.180836	0.000403	0.00171
TFRC	0.180855	0.000402	0.001708
NRG4	0.180858	0.000402	0.001708
SCUBE1	0.180885	0.000402	0.001705
PMPCA	0.180908	0.000401	0.001703
EDEM3	0.180974	0.000399	0.001697
GNAI3	0.181045	0.000397	0.00169
PLXNB2	0.181083	0.000396	0.001685
PGLYRP4	0.18112	0.000394	0.001681
TAF13	0.181192	0.000392	0.001676
CASC5	0.181207	0.000392	0.001674
KIF1C	0.181254	0.000391	0.001669
C6orf154	0.181258	0.00039	0.001669
MRPS35	0.181345	0.000388	0.00166
LRPAP1	0.181349	0.000388	0.001659
UBE2D2	0.181467	0.000384	0.001646
PC	0.181471	0.000384	0.001646
DTX2	0.181511	0.000383	0.001642
LSM3	0.181575	0.000381	0.001635
LXN	0.181631	0.00038	0.001629
SERINC4	0.181643	0.000379	0.001627
ARHGAP2(	0.18171	0.000377	0.001621
KRTAP13-	0.181714	0.000377	0.001621
PCDH1	0.181748	0.000376	0.001617
TAPBP	0.181754	0.000376	0.001617
KLHDC9	0.181758	0.000376	0.001617
HTR4	0.181827	0.000374	0.00161
PACSIN2	0.181879	0.000373	0.001604
CARD17	0.181886	0.000372	0.001603
UBE2N	0.18189	0.000372	0.001603
NDUFB8	0.181967	0.00037	0.001596
FAM96A	0.182046	0.000368	0.001588
SOCS2	0.182122	0.000366	0.00158
C14orf72	0.182134	0.000365	0.001579
PLSCR1	0.182199	0.000364	0.001573
IL18R1	0.182257	0.000362	0.001567
MRPL1	0.182265	0.000362	0.001567
FASTKD1	0.182267	0.000362	0.001567
ITK	0.182331	0.00036	0.00156
C18orf22	0.182332	0.00036	0.00156
FBXL14	0.182335	0.00036	0.00156
PGAM5	0.182395	0.000358	0.001556
FRK	0.18244	0.000357	0.001551
CCNB2	0.182445	0.000357	0.001551
LST-3TM1	0.182462	0.000356	0.00155
CDC25C	0.1825	0.000355	0.001546
NLRX1	0.182629	0.000352	0.001533
EPR1	0.182631	0.000352	0.001533
TMEM183,	0.182687	0.00035	0.001527
CCDC88B	0.182713	0.00035	0.001525
C11orf48	0.182728	0.000349	0.001524
ACBD5	0.182933	0.000344	0.001505
MTHFD1	0.18295	0.000343	0.001503
TMTC2	0.183064	0.00034	0.001491
PHB	0.183254	0.000336	0.001472
TMED9	0.183263	0.000335	0.001471

SERINC2	0.183266	0.000335	0.001471
SULT1C3	0.183471	0.00033	0.00145
CNOT10	0.183479	0.00033	0.00145
LAP3	0.183498	0.000329	0.001448
GUCA2A	0.183538	0.000328	0.001444
STAT3	0.183557	0.000328	0.001442
ZFYVE21	0.183633	0.000326	0.001435
GPR171	0.183668	0.000325	0.001432
GCET2	0.183682	0.000325	0.001431
ATAD3B	0.183699	0.000324	0.001429
SERF2	0.183722	0.000324	0.001427
TOX	0.183755	0.000323	0.001424
P2RX4	0.183807	0.000322	0.00142
SIL1	0.183902	0.000319	0.001412
TRIM31	0.183935	0.000319	0.001409
PARP9	0.183964	0.000318	0.001407
MREG	0.183969	0.000318	0.001407
RTN4IP1	0.183985	0.000317	0.001406
RAB43	0.184004	0.000317	0.001404
HIBCH	0.184038	0.000316	0.001401
GNPTAB	0.18404	0.000316	0.001401
GK3P	0.184067	0.000315	0.001399
CAPZB	0.184082	0.000315	0.001398
AP3B1	0.184101	0.000314	0.001396
NGLY1	0.18418	0.000313	0.001389
ABHD14A	0.184428	0.000307	0.001364
HK1	0.184447	0.000306	0.001362
POLD4	0.184698	0.0003	0.001341
GAN	0.18471	0.0003	0.00134
XRCC3	0.184807	0.000298	0.001331
RASSF7	0.184822	0.000298	0.00133
PNPLA3	0.184843	0.000297	0.001328
PRR15L	0.184918	0.000295	0.001324
MUL1	0.184942	0.000295	0.001322
C11orf93	0.185055	0.000292	0.001311
C20orf54	0.185135	0.00029	0.001304
PINX1	0.185168	0.00029	0.001302
APOL2	0.185285	0.000287	0.001292
SNRNP40	0.185542	0.000281	0.00127
RHOH	0.185617	0.00028	0.001264
CDC23	0.18568	0.000278	0.001258
C1orf201	0.185806	0.000276	0.001247
UBE2T	0.185835	0.000275	0.001245
RCOR1	0.185874	0.000274	0.001241
MXD3	0.185916	0.000273	0.001238
YIF1A	0.185917	0.000273	0.001238
MYO1C	0.185999	0.000272	0.001232
GSTT1	0.186179	0.000268	0.001216
KCNG3	0.186285	0.000266	0.001207
ITGAE	0.186308	0.000265	0.001205
RNF141	0.186336	0.000265	0.001203
ITLN2	0.186404	0.000263	0.001198
BAG1	0.186428	0.000263	0.001196
THG1L	0.186518	0.000261	0.001189
MKNK2	0.186564	0.00026	0.001185
ATP2C2	0.186595	0.000259	0.001183
ABO	0.186603	0.000259	0.001183
EBNA1BP2	0.18663	0.000259	0.001181

BTD	0.18676	0.000256	0.001171
RICH2	0.186823	0.000255	0.001166
CFD	0.187038	0.000251	0.001149
PRSS12	0.187143	0.000249	0.001141
RMI1	0.187153	0.000248	0.00114
LMLN	0.187204	0.000247	0.001136
HYAL4	0.187251	0.000247	0.001133
HLA-B	0.187256	0.000246	0.001132
KRAS	0.187283	0.000246	0.00113
VSTM2A	0.187352	0.000245	0.001125
HADHA	0.187403	0.000244	0.001122
LARS2	0.187719	0.000238	0.001097
DONSON	0.187765	0.000237	0.001093
CXCR5	0.187813	0.000236	0.00109
PKMYT1	0.187896	0.000234	0.001085
KYNU	0.18794	0.000234	0.001082
CCNYL1	0.187955	0.000233	0.001081
WRAP53	0.188062	0.000231	0.001072
C1orf21	0.188068	0.000231	0.001072
ADCY6	0.188075	0.000231	0.001072
NUP50	0.188136	0.00023	0.001067
TKT	0.188147	0.00023	0.001067
FCHO1	0.188183	0.000229	0.001064
HLA-A	0.188246	0.000228	0.00106
MYH7	0.188253	0.000228	0.001059
PGK1	0.188375	0.000226	0.00105
ECE2	0.188376	0.000226	0.00105
SLC4A5	0.188411	0.000225	0.001048
ADPRHL2	0.188421	0.000225	0.001048
EFCAB4B	0.188504	0.000224	0.001042
AKR7L	0.188527	0.000223	0.00104
CLDN23	0.188581	0.000222	0.001037
HSPA4L	0.188604	0.000222	0.001035
PIGO	0.188797	0.000218	0.001021
RDH5	0.188806	0.000218	0.001021
ARV1	0.188883	0.000217	0.001015
MAD2L1	0.188975	0.000215	0.001009
HARS	0.189151	0.000212	0.000997
LMAN2	0.189217	0.000211	0.000992
TRNAU1AI	0.189233	0.000211	0.000991
VEPH1	0.189272	0.00021	0.000988
PLIN3	0.189376	0.000209	0.000981
ADI1	0.189387	0.000209	0.000981
PLEKHB2	0.189403	0.000208	0.00098
L3MBTL2	0.18943	0.000208	0.000978
PGLYRP3	0.18943	0.000208	0.000978
BAX	0.189472	0.000207	0.000976
C14orf109	0.189489	0.000207	0.000975
ME2	0.189503	0.000207	0.000974
ASAP3	0.189677	0.000204	0.000961
IMPDH2	0.189757	0.000203	0.000956
PLK1	0.189758	0.000203	0.000956
TRIM58	0.189818	0.000202	0.000952
CHAF1B	0.18994	0.0002	0.000944
MVD	0.190013	0.000199	0.000939
DDTL	0.190022	0.000198	0.000939
OAS2	0.190091	0.000197	0.000935
PPIF	0.190132	0.000197	0.000932

MUT	0.190185	0.000196	0.000929
GCAT	0.19023	0.000195	0.000927
SLC9A4	0.190268	0.000195	0.000924
HIST1H2A	0.190323	0.000194	0.00092
KBTBD11	0.190389	0.000193	0.000916
COPS4	0.190519	0.000191	0.000908
SH3KBP1	0.190579	0.00019	0.000905
C20orf56	0.190602	0.000189	0.000904
FGD4	0.190613	0.000189	0.000903
PDSS2	0.190695	0.000188	0.000899
MMACHC	0.190757	0.000187	0.000895
FRMPD2	0.190812	0.000186	0.000891
CAPN2	0.190882	0.000185	0.000887
CCDC149	0.190895	0.000185	0.000887
DNAH5	0.190918	0.000185	0.000885
TCIRG1	0.190953	0.000184	0.000883
PLEKHM1	0.19102	0.000183	0.00088
TAX1BP3	0.19106	0.000183	0.000877
PLA2G4D	0.191063	0.000183	0.000877
LAIR2	0.191083	0.000182	0.000877
RNF125	0.191109	0.000182	0.000875
TTC22	0.191143	0.000182	0.000873
LEPREL1	0.191245	0.00018	0.000867
PRDX3	0.191463	0.000177	0.000854
CYB5R4	0.191585	0.000175	0.000846
TNFRSF10	0.191587	0.000175	0.000846
CDKN3	0.191716	0.000173	0.000839
AMD1	0.191777	0.000173	0.000835
ADH5	0.191896	0.000171	0.000828
GPRC5A	0.191968	0.00017	0.000824
BIN3	0.192004	0.000169	0.000822
SERPINB3	0.192083	0.000168	0.000818
ATAD3A	0.192263	0.000166	0.000806
RIMKLA	0.192307	0.000165	0.000804
ATG3	0.192572	0.000162	0.000788
NAGLU	0.192574	0.000162	0.000788
EI24	0.192624	0.000161	0.000785
TNS4	0.192673	0.000161	0.000783
C12orf23	0.192787	0.000159	0.000776
AGAP2	0.192793	0.000159	0.000776
CDC25A	0.192831	0.000159	0.000775
IL33	0.192833	0.000159	0.000775
C10orf99	0.192833	0.000159	0.000775
CHMP2B	0.192838	0.000159	0.000775
CYBA	0.19285	0.000158	0.000774
IL23R	0.192881	0.000158	0.000773
EXPH5	0.192918	0.000158	0.000771
MOSC2	0.19302	0.000156	0.000766
CD96	0.193206	0.000154	0.000756
CCNI2	0.193349	0.000152	0.000749
USP38	0.193364	0.000152	0.000748
LOC39132	0.193373	0.000152	0.000748
ROPN1B	0.193379	0.000152	0.000747
UBE2L3	0.193605	0.000149	0.000736
ADAMDEC	0.193613	0.000149	0.000736
SRBD1	0.193657	0.000148	0.000734
NBPF7	0.193686	0.000148	0.000733
FAM83B	0.193709	0.000148	0.000731

PDE6A	0.193794	0.000147	0.000727
GPHN	0.193832	0.000146	0.000725
SP140	0.193882	0.000146	0.000723
PF4	0.193888	0.000146	0.000722
KIT	0.193927	0.000145	0.00072
SFRS9	0.193947	0.000145	0.000719
PIGF	0.194007	0.000144	0.000716
UBE2D3	0.19413	0.000143	0.00071
LIMD1	0.194177	0.000142	0.000708
CD24	0.194201	0.000142	0.000707
GSTCD	0.194246	0.000142	0.000705
LOC10013	0.194625	0.000137	0.000686
UGT1A1	0.194662	0.000137	0.000684
MLF1IP	0.194669	0.000137	0.000684
TM9SF3	0.194732	0.000136	0.000681
MYO19	0.194748	0.000136	0.00068
GRIN1	0.194756	0.000136	0.00068
C17orf50	0.194764	0.000136	0.00068
SLC6A20	0.194766	0.000136	0.00068
THEMIS	0.194848	0.000135	0.000676
PREP	0.194906	0.000134	0.000673
TNFRSF14	0.195013	0.000133	0.000668
MMP7	0.195047	0.000133	0.000667
C9orf116	0.195079	0.000132	0.000665
PIAS2	0.195118	0.000132	0.000664
COL17A1	0.195124	0.000132	0.000663
LDLR	0.195142	0.000132	0.000663
44805	0.195259	0.00013	0.000657
GK	0.195356	0.000129	0.000652
FCRLA	0.195356	0.000129	0.000652
BCAR3	0.195374	0.000129	0.000652
SHPK	0.195443	0.000129	0.000649
KIAA0664	0.195545	0.000127	0.000645
OTC	0.195572	0.000127	0.000643
TNFRSF21	0.19562	0.000127	0.000641
TMEM63B	0.195628	0.000127	0.000641
TMSL3	0.195695	0.000126	0.000638
GAL3ST2	0.195698	0.000126	0.000638
TOMM22	0.195757	0.000125	0.000635
C16orf75	0.19576	0.000125	0.000635
CISD1	0.1958	0.000125	0.000633
HEATR5A	0.195889	0.000124	0.000629
C8orf41	0.195931	0.000124	0.000628
SLAMF7	0.195971	0.000123	0.000626
TLE6	0.196008	0.000123	0.000625
NDUFS4	0.19602	0.000123	0.000624
APOO	0.196025	0.000123	0.000624
MTM1	0.196033	0.000122	0.000624
TAPBPL	0.196082	0.000122	0.000621
DRD5	0.196082	0.000122	0.000621
KCNK6	0.196177	0.000121	0.000617
LRRC1	0.196264	0.00012	0.000613
BFSP2	0.196356	0.000119	0.00061
NEDD9	0.196361	0.000119	0.000609
C12orf48	0.196383	0.000119	0.000609
COX18	0.19639	0.000119	0.000608
NDUFB6	0.1964	0.000119	0.000608
GNA15	0.19643	0.000119	0.000607

GRPEL1	0.196601	0.000117	0.000599
HSD11B2	0.196664	0.000116	0.000597
RPN1	0.196687	0.000116	0.000596
PUSL1	0.196735	0.000116	0.000594
SCNN1A	0.196746	0.000116	0.000594
LOC72908	0.196748	0.000116	0.000594
LRRC8B	0.196787	0.000115	0.000592
AHCYL1	0.196863	0.000114	0.000589
DOCK5	0.196983	0.000113	0.000584
YIPF5	0.196996	0.000113	0.000583
SHCBP1	0.197011	0.000113	0.000583
HMMR	0.197096	0.000112	0.00058
SLC17A4	0.197235	0.000111	0.000574
KRT20	0.197244	0.000111	0.000574
PAQR8	0.197316	0.00011	0.000572
ELOVL1	0.197386	0.00011	0.000569
LIPG	0.197432	0.000109	0.000567
LILRP2	0.197494	0.000109	0.000565
SLCO2A1	0.197524	0.000108	0.000563
HOXD1	0.197536	0.000108	0.000563
C4orf34	0.197575	0.000108	0.000562
JTB	0.197585	0.000108	0.000561
ARPC4	0.197725	0.000107	0.000556
EHHADH	0.197816	0.000106	0.000552
SETD3	0.197869	0.000105	0.00055
ZNF215	0.197899	0.000105	0.000549
CYB5R2	0.197957	0.000105	0.000547
UBXN10	0.198103	0.000103	0.000541
LYPD6B	0.198293	0.000102	0.000533
SIRT6	0.198333	0.000101	0.000532
DMRT2	0.198364	0.000101	0.00053
FRRS1	0.198404	0.000101	0.000529
CDC45	0.198457	0.0001	0.000527
MCCC2	0.198538	9.98E-05	0.000524
METAP1	0.198541	9.97E-05	0.000524
TOMM40L	0.198555	9.96E-05	0.000524
NDUFA1	0.198566	9.95E-05	0.000524
SKA1	0.198573	9.95E-05	0.000523
MORC4	0.198603	9.92E-05	0.000522
USMG5	0.198903	9.68E-05	0.00051
HLA-E	0.19896	9.64E-05	0.000508
CA8	0.198974	9.62E-05	0.000508
LANCL3	0.198976	9.62E-05	0.000508
GIPR	0.19899	9.61E-05	0.000508
CD101	0.199096	9.53E-05	0.000503
ATP5I	0.199119	9.51E-05	0.000503
FAM129B	0.199157	9.48E-05	0.000501
CYP2C19	0.199214	9.43E-05	0.000499
PDSS1	0.199315	9.36E-05	0.000496
PHLDA2	0.199459	9.25E-05	0.000491
LRRC66	0.199599	9.14E-05	0.000486
SLC16A9	0.199696	9.07E-05	0.000483
HS3ST1	0.19977	9.01E-05	0.000481
VPS37B	0.199899	8.91E-05	0.000476
LOC72917	0.1999	8.91E-05	0.000476
NDUFA12	0.199984	8.85E-05	0.000473
TTC9B	0.200049	8.8E-05	0.000471
TNFRSF6B	0.200069	8.79E-05	0.00047

KLK3	0.200073	8.79E-05	0.00047
CISH	0.200098	8.77E-05	0.000469
ICMT	0.200298	8.62E-05	0.000463
S1PR4	0.200325	8.6E-05	0.000462
KCNV1	0.200374	8.57E-05	0.00046
NUDT16P1	0.200374	8.57E-05	0.00046
RAB25	0.200397	8.55E-05	0.00046
NEK6	0.200879	8.22E-05	0.000443
TRIM47	0.200918	8.19E-05	0.000442
COX7B	0.201105	8.06E-05	0.000436
C1orf69	0.201175	8.02E-05	0.000434
TMEM93	0.201206	8E-05	0.000433
F12	0.201272	7.95E-05	0.000431
DLGAP5	0.201344	7.9E-05	0.000429
HMOX2	0.201353	7.9E-05	0.000429
DHRS13	0.201419	7.85E-05	0.000426
SIPA1L2	0.201475	7.82E-05	0.000425
METTL12	0.20148	7.81E-05	0.000425
ANXA11	0.201516	7.79E-05	0.000423
CKS2	0.201532	7.78E-05	0.000423
OIP5	0.201561	7.76E-05	0.000422
GSPT1	0.201591	7.74E-05	0.000421
FEN1	0.201646	7.71E-05	0.00042
KIAA1161	0.201686	7.68E-05	0.000419
FAM23A	0.201763	7.63E-05	0.000416
SPCS1	0.201799	7.61E-05	0.000415
RNH1	0.201878	7.56E-05	0.000413
C5orf30	0.201893	7.55E-05	0.000412
P2RY10	0.201909	7.54E-05	0.000412
GLOD4	0.201914	7.54E-05	0.000412
LEFTY1	0.201931	7.52E-05	0.000411
NPHS2	0.201974	7.5E-05	0.00041
PKP3	0.201991	7.49E-05	0.00041
C16orf93	0.202104	7.42E-05	0.000406
RG9MTD2	0.202347	7.27E-05	0.000398
LOC10018	0.202347	7.27E-05	0.000398
C17orf97	0.202353	7.26E-05	0.000398
TUBGCP2	0.202373	7.25E-05	0.000398
GPI	0.202499	7.17E-05	0.000395
EDEM1	0.202516	7.16E-05	0.000394
SCLT1	0.202638	7.09E-05	0.000391
CD74	0.202678	7.07E-05	0.00039
ETF1	0.20282	6.98E-05	0.000386
KIF19	0.20285	6.97E-05	0.000385
NCF4	0.20286	6.96E-05	0.000385
LOC64300	0.203017	6.87E-05	0.00038
NDUFV3	0.20302	6.87E-05	0.00038
TPD52	0.203064	6.84E-05	0.000379
ZNF440	0.203066	6.84E-05	0.000379
LLPH	0.203203	6.76E-05	0.000375
ATRIP	0.203208	6.76E-05	0.000375
IBTK	0.203237	6.74E-05	0.000375
BEND3	0.203371	6.67E-05	0.000371
PHKA1	0.203519	6.58E-05	0.000367
C1orf93	0.20352	6.58E-05	0.000367
NUDCD2	0.203539	6.57E-05	0.000366
MED18	0.203598	6.54E-05	0.000365
B9D2	0.203631	6.52E-05	0.000364

TLN2	0.203868	6.39E-05	0.000357
RASGRP1	0.203938	6.35E-05	0.000356
RCC1	0.203972	6.34E-05	0.000355
TIFA	0.203977	6.33E-05	0.000355
NAGA	0.204131	6.25E-05	0.000351
NT5E	0.204328	6.15E-05	0.000346
PNOC	0.204365	6.13E-05	0.000345
ABCB8	0.204393	6.11E-05	0.000345
DHRS7C	0.204394	6.11E-05	0.000345
PCNA	0.204448	6.09E-05	0.000343
ASNA1	0.20452	6.05E-05	0.000341
NEURL	0.204628	5.99E-05	0.000338
SLCO4C1	0.204651	5.98E-05	0.000338
CHAF1A	0.204683	5.97E-05	0.000337
S100A16	0.204748	5.93E-05	0.000336
NDUFB2	0.204779	5.92E-05	0.000335
CBARA1	0.204791	5.91E-05	0.000334
CMC1	0.205022	5.8E-05	0.000329
CLRN3	0.205058	5.78E-05	0.000328
C18orf19	0.205075	5.77E-05	0.000327
UQCR11	0.205082	5.77E-05	0.000327
MYL12B	0.205122	5.75E-05	0.000326
CRADD	0.205226	5.7E-05	0.000324
PSMA4	0.205273	5.67E-05	0.000323
CENPK	0.205309	5.66E-05	0.000322
POLA2	0.205467	5.58E-05	0.000318
ZC3H12D	0.205494	5.57E-05	0.000317
CEACAM3	0.205525	5.55E-05	0.000317
44623	0.205545	5.54E-05	0.000316
CT62	0.20558	5.53E-05	0.000315
PLCB3	0.205697	5.47E-05	0.000313
CLPP	0.205834	5.41E-05	0.000309
LOC64662	0.205981	5.34E-05	0.000306
GMNN	0.206134	5.27E-05	0.000302
CASP6	0.206295	5.2E-05	0.000299
FAM132A	0.206365	5.17E-05	0.000297
TMEM48	0.206399	5.15E-05	0.000297
LOC11323	0.206454	5.13E-05	0.000296
CD55	0.206627	5.05E-05	0.000292
HEMK1	0.206636	5.05E-05	0.000292
ING2	0.206668	5.04E-05	0.000291
MED7	0.206721	5.01E-05	0.00029
TNFRSF13	0.206766	4.99E-05	0.000289
PPCS	0.206797	4.98E-05	0.000289
ISOC2	0.207033	4.88E-05	0.000283
VAMP3	0.207033	4.88E-05	0.000283
L2HGDH	0.207117	4.85E-05	0.000282
FAR2	0.207182	4.82E-05	0.00028
ANPEP	0.207317	4.76E-05	0.000278
GOLPH3L	0.207348	4.75E-05	0.000277
EPS8L3	0.207356	4.75E-05	0.000277
C19orf36	0.207426	4.72E-05	0.000275
SH2D6	0.207514	4.68E-05	0.000274
ALOX5	0.207518	4.68E-05	0.000274
AGBL4	0.207627	4.64E-05	0.000271
HMGCS1	0.207868	4.54E-05	0.000266
INF2	0.207947	4.51E-05	0.000265
SNX20	0.207948	4.51E-05	0.000265

NCR3	0.208052	4.47E-05	0.000263
LENG9	0.208054	4.47E-05	0.000263
CD7	0.208141	4.44E-05	0.000261
FBXO25	0.208195	4.42E-05	0.00026
CABIN1	0.208315	4.37E-05	0.000258
TMEM220	0.208326	4.37E-05	0.000257
CLIC5	0.208363	4.35E-05	0.000257
YES1	0.208382	4.35E-05	0.000256
PPCDC	0.208393	4.34E-05	0.000256
LOC10012	0.208408	4.34E-05	0.000256
NOSTRIN	0.208423	4.33E-05	0.000256
MINK1	0.208444	4.32E-05	0.000256
SRA1	0.208639	4.25E-05	0.000252
VPS53	0.208683	4.23E-05	0.000251
COPS3	0.208683	4.23E-05	0.000251
MED9	0.208761	4.2E-05	0.00025
MPND	0.208773	4.2E-05	0.00025
PI4K2B	0.20879	4.19E-05	0.000249
MXD1	0.208816	4.18E-05	0.000249
TRIM72	0.208888	4.16E-05	0.000248
UBE2J2	0.20889	4.16E-05	0.000248
PGGT1B	0.208924	4.15E-05	0.000247
LRMP	0.209088	4.09E-05	0.000244
NDUFB5	0.209217	4.04E-05	0.000242
CALCOCO	0.209237	4.03E-05	0.000242
C9orf125	0.209264	4.03E-05	0.000241
ADAM28	0.209293	4.02E-05	0.000241
SELENBP1	0.209298	4.01E-05	0.00024
SLC1A1	0.209478	3.95E-05	0.000237
TEX264	0.209493	3.95E-05	0.000237
ZNF672	0.209496	3.94E-05	0.000237
YTHDC2	0.209672	3.89E-05	0.000234
ADM	0.209688	3.88E-05	0.000234
HDAC1	0.209747	3.86E-05	0.000233
ATP5H	0.209838	3.83E-05	0.000231
KIAA1539	0.209874	3.82E-05	0.000231
KLRC1	0.209886	3.81E-05	0.00023
SLC6A10P	0.209891	3.81E-05	0.00023
TGM3	0.209955	3.79E-05	0.000229
SDF2L1	0.209976	3.78E-05	0.000229
SLC16A3	0.209976	3.78E-05	0.000229
UTS2	0.210029	3.77E-05	0.000228
C18orf8	0.210064	3.75E-05	0.000227
MADCAM1	0.210097	3.74E-05	0.000227
C21orf88	0.210132	3.73E-05	0.000226
EBP	0.210197	3.71E-05	0.000225
MUC17	0.210257	3.69E-05	0.000224
KIR2DL4	0.210265	3.69E-05	0.000224
GTF2A2	0.210321	3.67E-05	0.000223
HIATL1	0.210448	3.63E-05	0.000221
GPR44	0.210503	3.61E-05	0.00022
TAT	0.210509	3.61E-05	0.00022
C11orf92	0.210564	3.59E-05	0.000219
MLX	0.210573	3.59E-05	0.000219
NACC1	0.210602	3.58E-05	0.000219
TACO1	0.210622	3.58E-05	0.000219
DYRK4	0.210662	3.56E-05	0.000218
CD2	0.210665	3.56E-05	0.000218

LPCAT1	0.210708	3.55E-05	0.000218
CCNA2	0.210718	3.55E-05	0.000217
HTR3E	0.210854	3.5E-05	0.000215
PCSK9	0.210919	3.48E-05	0.000214
IFRD2	0.210941	3.48E-05	0.000214
FPGT	0.21098	3.47E-05	0.000213
IGFALS	0.211056	3.44E-05	0.000212
EPB41L2	0.211093	3.43E-05	0.000212
SCNN1B	0.211116	3.42E-05	0.000211
DLAT	0.211116	3.42E-05	0.000211
HDHD3	0.211148	3.42E-05	0.000211
CMAS	0.211249	3.39E-05	0.000209
STX10	0.211288	3.37E-05	0.000209
CALM3	0.211354	3.35E-05	0.000208
CSTB	0.211387	3.34E-05	0.000207
RPL36AL	0.211511	3.31E-05	0.000205
MGST2	0.211642	3.27E-05	0.000203
TMEM165	0.211734	3.24E-05	0.000202
BRP44	0.211812	3.22E-05	0.000201
S100A10	0.211898	3.2E-05	0.000199
CASR	0.211911	3.19E-05	0.000199
C9	0.211928	3.19E-05	0.000199
BTN3A3	0.211956	3.18E-05	0.000199
KIF11	0.211988	3.17E-05	0.000198
MAP2K6	0.211997	3.17E-05	0.000198
ACN9	0.21202	3.16E-05	0.000198
C17orf81	0.212027	3.16E-05	0.000198
DAP	0.212141	3.13E-05	0.000196
PPP1CC	0.212189	3.12E-05	0.000195
TBC1D10C	0.21234	3.08E-05	0.000193
PAX9	0.212454	3.04E-05	0.000191
PQLC1	0.21257	3.01E-05	0.00019
CYTIP	0.212609	3E-05	0.000189
HADHB	0.212651	2.99E-05	0.000188
ALDH2	0.212703	2.98E-05	0.000188
UBE2L6	0.212741	2.97E-05	0.000187
CCDC125	0.212878	2.93E-05	0.000185
ADORA2B	0.212897	2.93E-05	0.000185
COX7A2	0.212919	2.92E-05	0.000185
TNFSF9	0.21318	2.86E-05	0.000181
AMPD1	0.213189	2.85E-05	0.000181
C12orf36	0.213203	2.85E-05	0.000181
MRPL50	0.213223	2.84E-05	0.000181
IL1RN	0.213299	2.83E-05	0.00018
KCTD5	0.213308	2.82E-05	0.00018
EIF3K	0.213366	2.81E-05	0.000179
MAP4K1	0.213436	2.79E-05	0.000178
SLC25A37	0.213441	2.79E-05	0.000178
SUCLG1	0.213453	2.79E-05	0.000178
DDT	0.213628	2.74E-05	0.000176
EDN2	0.213692	2.73E-05	0.000175
COMMD4	0.213769	2.71E-05	0.000174
CD6	0.213782	2.71E-05	0.000174
CCNO	0.213822	2.7E-05	0.000173
GJB5	0.213925	2.67E-05	0.000172
HCCS	0.213938	2.67E-05	0.000172
IL17F	0.214227	2.6E-05	0.000168
MALL	0.214327	2.58E-05	0.000166

MYO1A	0.214348	2.57E-05	0.000166
TLR3	0.21464	2.51E-05	0.000162
ACAT2	0.214665	2.5E-05	0.000162
KAT2B	0.214809	2.47E-05	0.00016
CAPN8	0.214941	2.44E-05	0.000158
DPCD	0.215001	2.43E-05	0.000158
PPP1R14D	0.215021	2.43E-05	0.000157
SEMA6D	0.215042	2.42E-05	0.000157
CD320	0.215057	2.42E-05	0.000157
DHRS11	0.215075	2.41E-05	0.000157
ALPPL2	0.21515	2.4E-05	0.000156
UGDH	0.215244	2.38E-05	0.000155
INPP1	0.215268	2.37E-05	0.000155
PRSS16	0.215372	2.35E-05	0.000153
RRM1	0.215373	2.35E-05	0.000153
C3orf52	0.215433	2.34E-05	0.000153
SOD1	0.2155	2.32E-05	0.000152
CCL20	0.215543	2.31E-05	0.000152
IRF1	0.215818	2.26E-05	0.000148
CHRM1	0.215822	2.26E-05	0.000148
DUSP4	0.215845	2.25E-05	0.000148
GYS1	0.216024	2.22E-05	0.000146
PLCXD1	0.21617	2.19E-05	0.000144
DHX32	0.21623	2.18E-05	0.000144
MRPS7	0.216238	2.17E-05	0.000143
DCLRE1B	0.216242	2.17E-05	0.000143
CABP4	0.216368	2.15E-05	0.000142
ALDH3A2	0.21641	2.14E-05	0.000142
CDHR5	0.216503	2.12E-05	0.00014
APAF1	0.216504	2.12E-05	0.00014
HSD3B7	0.216572	2.11E-05	0.00014
RHOF	0.216593	2.11E-05	0.00014
SLC30A10	0.216597	2.11E-05	0.00014
TIMM17A	0.216609	2.1E-05	0.000139
CAMK2D	0.21664	2.1E-05	0.000139
B3GNT5	0.216672	2.09E-05	0.000139
ASF1B	0.216753	2.08E-05	0.000138
FOXRED1	0.216799	2.07E-05	0.000138
APOL1	0.216845	2.06E-05	0.000137
AKR1B15	0.216928	2.04E-05	0.000136
TIMM13	0.21696	2.04E-05	0.000136
FLJ40330	0.217053	2.02E-05	0.000135
MRPS36	0.217112	2.01E-05	0.000134
UBE2J1	0.217275	1.98E-05	0.000133
NPSR1	0.217276	1.98E-05	0.000133
BATF2	0.217304	1.98E-05	0.000133
UBASH3A	0.217373	1.96E-05	0.000132
GBA2	0.21747	1.95E-05	0.000131
SLC44A3	0.21776	1.9E-05	0.000128
HLA-C	0.217817	1.89E-05	0.000127
HLA-DMA	0.21788	1.88E-05	0.000127
AP1B1	0.217885	1.87E-05	0.000127
ARSA	0.217931	1.87E-05	0.000126
DTWD2	0.217948	1.86E-05	0.000126
PRELID1	0.217978	1.86E-05	0.000126
PSMA6	0.217989	1.86E-05	0.000126
HPS6	0.218069	1.84E-05	0.000125
TUFM	0.218174	1.83E-05	0.000124

CLDN12	0.218208	1.82E-05	0.000124
GTF2E2	0.218246	1.81E-05	0.000124
ERP29	0.218262	1.81E-05	0.000123
P2RY2	0.218315	1.8E-05	0.000123
C6orf203	0.218346	1.8E-05	0.000123
C11orf53	0.218381	1.79E-05	0.000122
MGST3	0.218444	1.78E-05	0.000122
MRPL22	0.218479	1.78E-05	0.000122
RAB11A	0.218502	1.77E-05	0.000121
FEM1C	0.218512	1.77E-05	0.000121
ANKS4B	0.218607	1.76E-05	0.00012
TFF3	0.21861	1.76E-05	0.00012
CDCA3	0.218818	1.72E-05	0.000118
MPZL2	0.218826	1.72E-05	0.000118
ABCC3	0.218834	1.72E-05	0.000118
POP5	0.218899	1.71E-05	0.000118
GPR98	0.218927	1.71E-05	0.000117
UCP2	0.218942	1.7E-05	0.000117
MGC8704[illegible]	0.218954	1.7E-05	0.000117
SPRYD4	0.218967	1.7E-05	0.000117
NADSYN1	0.218981	1.7E-05	0.000117
GBP3	0.219032	1.69E-05	0.000117
C3orf10	0.219059	1.69E-05	0.000116
ACAD8	0.219124	1.68E-05	0.000116
GAS2L1	0.219142	1.67E-05	0.000116
LY9	0.219173	1.67E-05	0.000116
MRPS18A	0.219259	1.66E-05	0.000115
GZMM	0.219291	1.65E-05	0.000115
TACC3	0.219454	1.63E-05	0.000113
LDLRAP1	0.219546	1.61E-05	0.000112
RBP3	0.219555	1.61E-05	0.000112
MRPL12	0.219567	1.61E-05	0.000112
RBBP8	0.219657	1.6E-05	0.000111
MCEE	0.219692	1.59E-05	0.000111
SH3BP1	0.219721	1.59E-05	0.000111
UGP2	0.219788	1.58E-05	0.00011
AP1M2	0.219816	1.57E-05	0.00011
TSFM	0.219836	1.57E-05	0.00011
LAX1	0.219931	1.56E-05	0.000109
NR2F6	0.219944	1.56E-05	0.000109
HRCT1	0.220031	1.54E-05	0.000108
PTRH1	0.220167	1.52E-05	0.000107
C18orf10	0.220356	1.5E-05	0.000105
PTP4A2	0.220474	1.48E-05	0.000104
KPNA2	0.220576	1.47E-05	0.000104
DEPDC1	0.220736	1.45E-05	0.000102
RNASE4	0.220792	1.44E-05	0.000102
HINT1	0.220815	1.44E-05	0.000102
C1QBP	0.22088	1.43E-05	0.000101
NUDT18	0.220901	1.42E-05	0.000101
MRPS18C	0.220974	1.42E-05	0.0001
ISG20	0.221077	1.4E-05	9.96E-05
FOS	0.221082	1.4E-05	9.96E-05
FLNB	0.221194	1.39E-05	9.86E-05
C14orf53	0.221421	1.36E-05	9.68E-05
IDH3B	0.221437	1.36E-05	9.67E-05
RPH3AL	0.221446	1.36E-05	9.67E-05
ORAI1	0.221467	1.35E-05	9.66E-05

NDUFAF1	0.221615	1.33E-05	9.55E-05
NPY2R	0.221677	1.33E-05	9.5E-05
AK1	0.221685	1.33E-05	9.49E-05
GAK	0.221821	1.31E-05	9.39E-05
NCLN	0.221847	1.31E-05	9.37E-05
NDUFB1	0.221931	1.3E-05	9.32E-05
ACOT11	0.222059	1.28E-05	9.22E-05
SPTLC3	0.22207	1.28E-05	9.22E-05
GZMA	0.222101	1.28E-05	9.2E-05
MAPK3	0.222145	1.27E-05	9.17E-05
LDHA	0.222219	1.26E-05	9.11E-05
MUC5B	0.222242	1.26E-05	9.1E-05
C11orf90	0.222489	1.23E-05	8.92E-05
PDE12	0.222728	1.2E-05	8.74E-05
GNA14	0.222728	1.2E-05	8.74E-05
TMEM50B	0.222753	1.2E-05	8.73E-05
DTX3L	0.222842	1.19E-05	8.67E-05
IL15RA	0.222884	1.19E-05	8.64E-05
COTL1	0.222911	1.18E-05	8.63E-05
PDK1	0.22293	1.18E-05	8.62E-05
GCH1	0.222998	1.17E-05	8.58E-05
METT5D1	0.223033	1.17E-05	8.56E-05
MLF2	0.223059	1.17E-05	8.54E-05
CORO2A	0.223078	1.17E-05	8.53E-05
PITX1	0.223137	1.16E-05	8.48E-05
HMGCL	0.223227	1.15E-05	8.42E-05
CD40LG	0.223333	1.14E-05	8.34E-05
CXCL2	0.223356	1.14E-05	8.33E-05
CCDC109B	0.223489	1.12E-05	8.24E-05
NDUFA2	0.223541	1.12E-05	8.21E-05
NPDC1	0.223572	1.11E-05	8.19E-05
OAZ3	0.223644	1.11E-05	8.14E-05
ANK1	0.22367	1.1E-05	8.13E-05
AGFG2	0.223774	1.09E-05	8.07E-05
NDUFC1	0.223851	1.09E-05	8.02E-05
TTC19	0.22405	1.07E-05	7.88E-05
BOLA3	0.224093	1.06E-05	7.86E-05
CD3E	0.224204	1.05E-05	7.79E-05
TMEM37	0.224362	1.03E-05	7.7E-05
CCDC51	0.224421	1.03E-05	7.67E-05
AQP8	0.224694	1E-05	7.5E-05
HEPH	0.224719	1E-05	7.49E-05
C9orf89	0.224797	9.94E-06	7.44E-05
RAVER2	0.224835	9.9E-06	7.42E-05
DEFB4A	0.224842	9.89E-06	7.42E-05
DPM3	0.224889	9.85E-06	7.4E-05
C11orf20	0.224947	9.8E-06	7.37E-05
ZNF511	0.224948	9.8E-06	7.37E-05
SAMD5	0.224964	9.78E-06	7.36E-05
ACADM	0.225056	9.7E-06	7.31E-05
DCI	0.225374	9.41E-06	7.12E-05
ATP11B	0.225421	9.37E-06	7.1E-05
SECTM1	0.225499	9.31E-06	7.05E-05
AIM2	0.225507	9.3E-06	7.04E-05
SAA1	0.225524	9.28E-06	7.04E-05
PPP2CA	0.225647	9.18E-06	6.97E-05
FBXW9	0.225664	9.16E-06	6.96E-05
FZD5	0.225728	9.11E-06	6.92E-05

TLE1	0.22573	9.11E-06	6.92E-05
NKX2-2	0.225793	9.05E-06	6.89E-05
MRPS16	0.225837	9.01E-06	6.86E-05
SLC12A2	0.225857	9E-06	6.85E-05
DLD	0.225882	8.98E-06	6.84E-05
ACOT7	0.22591	8.95E-06	6.83E-05
MTFMT	0.226091	8.8E-06	6.72E-05
MMP10	0.226123	8.78E-06	6.7E-05
SCP2	0.226137	8.76E-06	6.7E-05
CMBL	0.22616	8.75E-06	6.69E-05
CD3G	0.226172	8.74E-06	6.68E-05
GSTK1	0.226198	8.71E-06	6.67E-05
PPIL5	0.226291	8.64E-06	6.62E-05
SYNGR2	0.226354	8.59E-06	6.59E-05
FAM160A1	0.22642	8.53E-06	6.56E-05
PIGS	0.226489	8.48E-06	6.52E-05
ANO2	0.22655	8.43E-06	6.49E-05
ABHD6	0.226569	8.42E-06	6.48E-05
C1orf177	0.226571	8.41E-06	6.48E-05
HAPLN1	0.226664	8.34E-06	6.43E-05
NOP10	0.226751	8.27E-06	6.39E-05
STIL	0.226762	8.26E-06	6.39E-05
NDRG2	0.226795	8.24E-06	6.38E-05
SULT1C2	0.226845	8.2E-06	6.35E-05
MRPL41	0.226893	8.16E-06	6.33E-05
TRIM21	0.227017	8.07E-06	6.26E-05
EZR	0.227146	7.97E-06	6.19E-05
PIP5K1B	0.227238	7.9E-06	6.14E-05
HAUS8	0.227333	7.83E-06	6.1E-05
PDE4D	0.227344	7.82E-06	6.09E-05
RRM2	0.227465	7.73E-06	6.03E-05
C3orf33	0.227468	7.73E-06	6.03E-05
OVCA2	0.227531	7.69E-06	6E-05
TRAT1	0.227555	7.67E-06	5.99E-05
ALDH16A1	0.227561	7.66E-06	5.99E-05
PTTG1	0.227591	7.64E-06	5.98E-05
DOK4	0.227591	7.64E-06	5.98E-05
SLC25A22	0.227663	7.59E-06	5.95E-05
SLC17A5	0.227795	7.5E-06	5.89E-05
FAHD1	0.227876	7.44E-06	5.85E-05
PMM2	0.227941	7.39E-06	5.81E-05
CEP78	0.228063	7.31E-06	5.76E-05
CISD3	0.22808	7.3E-06	5.76E-05
MRPS27	0.228107	7.28E-06	5.75E-05
METTL7A	0.228149	7.25E-06	5.73E-05
PFKL	0.228159	7.24E-06	5.73E-05
NDUFS3	0.228301	7.15E-06	5.66E-05
OSBPL7	0.228418	7.07E-06	5.6E-05
CCRL1	0.228483	7.02E-06	5.57E-05
MRPL4	0.228605	6.94E-06	5.52E-05
MIA2	0.228692	6.89E-06	5.49E-05
SSTR1	0.228826	6.8E-06	5.44E-05
MLKL	0.229014	6.68E-06	5.35E-05
UNC13B	0.229221	6.55E-06	5.25E-05
CCDC134	0.229358	6.46E-06	5.19E-05
CXCL11	0.229361	6.46E-06	5.19E-05
ATP5C1	0.229367	6.46E-06	5.19E-05
FOXA1	0.229512	6.37E-06	5.13E-05

CD27	0.229781	6.21E-06	5.01E-05
GEMIN4	0.229784	6.21E-06	5.01E-05
CLMN	0.229843	6.17E-06	4.99E-05
CCL15	0.230249	5.94E-06	4.81E-05
P4HB	0.230276	5.92E-06	4.81E-05
CLTB	0.230282	5.92E-06	4.8E-05
SLC30A7	0.230324	5.89E-06	4.79E-05
PRR13	0.230351	5.88E-06	4.78E-05
DNAJC28	0.230472	5.81E-06	4.74E-05
PWWP2B	0.230484	5.8E-06	4.74E-05
PSMB9	0.23054	5.77E-06	4.72E-05
NOX1	0.230828	5.62E-06	4.61E-05
POLR2L	0.230861	5.6E-06	4.6E-05
SLC24A6	0.230905	5.57E-06	4.59E-05
TUBB2A	0.231044	5.5E-06	4.53E-05
DCTPP1	0.231056	5.49E-06	4.53E-05
SLC22A23	0.23106	5.49E-06	4.53E-05
GPR126	0.231119	5.46E-06	4.51E-05
AFAP1L2	0.231204	5.42E-06	4.48E-05
RNMTL1	0.231249	5.39E-06	4.46E-05
SFXN1	0.231268	5.38E-06	4.46E-05
NIPA2	0.231298	5.37E-06	4.45E-05
STXBP6	0.231486	5.27E-06	4.38E-05
ARF4	0.231499	5.27E-06	4.38E-05
ACAT1	0.231527	5.25E-06	4.37E-05
NDUFA10	0.231584	5.22E-06	4.35E-05
MRRF	0.231688	5.17E-06	4.32E-05
SEMA4G	0.232005	5.02E-06	4.2E-05
GOT2	0.232008	5.01E-06	4.2E-05
GALNT5	0.232025	5.01E-06	4.19E-05
RAB19	0.232057	4.99E-06	4.18E-05
APIP	0.232131	4.95E-06	4.16E-05
COL4A6	0.232181	4.93E-06	4.14E-05
HPS1	0.232343	4.85E-06	4.09E-05
C4orf32	0.232515	4.77E-06	4.03E-05
MRPS2	0.23259	4.74E-06	4E-05
FCRL2	0.232609	4.73E-06	4E-05
LSMD1	0.23261	4.73E-06	4E-05
C19orf10	0.232669	4.7E-06	3.98E-05
CAP1	0.232736	4.67E-06	3.96E-05
CDC42	0.232751	4.67E-06	3.96E-05
KIF9	0.233015	4.55E-06	3.87E-05
CBFA2T3	0.233048	4.53E-06	3.86E-05
LMO4	0.233222	4.46E-06	3.8E-05
EPHB3	0.233251	4.45E-06	3.8E-05
NDUFAB1	0.233507	4.34E-06	3.72E-05
SPTLC2	0.233613	4.29E-06	3.69E-05
MYO5C	0.233625	4.29E-06	3.68E-05
ASL	0.233713	4.25E-06	3.66E-05
BLOC1S1	0.233757	4.23E-06	3.65E-05
EFHD2	0.233857	4.19E-06	3.62E-05
DND1	0.233882	4.18E-06	3.61E-05
COX7C	0.233886	4.18E-06	3.61E-05
FABP1	0.233955	4.15E-06	3.6E-05
B3GNT4	0.234125	4.08E-06	3.55E-05
GDI2	0.23413	4.08E-06	3.55E-05
MRPL20	0.234132	4.08E-06	3.55E-05
GALNT12	0.234147	4.08E-06	3.55E-05

ORC1L	0.234188	4.06E-06	3.53E-05
PPPDE2	0.23443	3.96E-06	3.46E-05
ITPK1	0.234449	3.96E-06	3.46E-05
TMBIM6	0.234492	3.94E-06	3.45E-05
LRRC40	0.234835	3.81E-06	3.35E-05
SNPH	0.234851	3.8E-06	3.34E-05
C14orf34	0.234868	3.8E-06	3.34E-05
MBOAT1	0.234952	3.77E-06	3.31E-05
SF3B5	0.234966	3.76E-06	3.31E-05
SH3RF1	0.235132	3.7E-06	3.27E-05
VANGL1	0.235262	3.66E-06	3.23E-05
FAM84A	0.235524	3.56E-06	3.16E-05
SDF4	0.235588	3.54E-06	3.14E-05
MYD88	0.235661	3.52E-06	3.12E-05
PRR5L	0.235776	3.48E-06	3.09E-05
MRPL37	0.2358	3.47E-06	3.08E-05
PCSK6	0.235823	3.46E-06	3.08E-05
PFKFB2	0.235828	3.46E-06	3.08E-05
HES5	0.23583	3.46E-06	3.08E-05
LYZ	0.235923	3.43E-06	3.05E-05
SEMG2	0.235986	3.41E-06	3.04E-05
LYN	0.23603	3.39E-06	3.03E-05
IL1B	0.23616	3.35E-06	2.99E-05
MIER1	0.236314	3.3E-06	2.96E-05
TRAF3IP2	0.236319	3.3E-06	2.96E-05
SLC27A2	0.236361	3.28E-06	2.95E-05
ST5	0.236374	3.28E-06	2.94E-05
C12orf72	0.23648	3.24E-06	2.92E-05
CLDN2	0.236481	3.24E-06	2.92E-05
MRPL16	0.236586	3.21E-06	2.89E-05
CDC42BPG	0.236672	3.18E-06	2.87E-05
FAM108C1	0.236864	3.12E-06	2.82E-05
CLCA2	0.237051	3.07E-06	2.78E-05
TTC39A	0.237175	3.03E-06	2.75E-05
ABCG5	0.237215	3.02E-06	2.75E-05
SPINK5	0.237329	2.98E-06	2.72E-05
KIAA0391	0.237574	2.91E-06	2.66E-05
ASPG	0.237646	2.89E-06	2.65E-05
FAM5C	0.23773	2.87E-06	2.63E-05
MRPL44	0.237742	2.86E-06	2.62E-05
TMEM72	0.237853	2.83E-06	2.6E-05
CTAGE5	0.237954	2.8E-06	2.57E-05
CBR1	0.237968	2.8E-06	2.57E-05
XRCC6BP1	0.238024	2.79E-06	2.56E-05
PLLP	0.238028	2.78E-06	2.56E-05
CDCA2	0.238054	2.78E-06	2.56E-05
EIF4E	0.238068	2.77E-06	2.55E-05
PKIB	0.238136	2.75E-06	2.54E-05
NECAP1	0.238143	2.75E-06	2.54E-05
KRT2	0.238198	2.74E-06	2.53E-05
ARFIP2	0.2383	2.71E-06	2.51E-05
SYTL1	0.238369	2.69E-06	2.5E-05
CCDC56	0.238438	2.67E-06	2.48E-05
LRIG3	0.238475	2.66E-06	2.47E-05
IL17C	0.238562	2.64E-06	2.46E-05
RPS27L	0.238697	2.61E-06	2.43E-05
STAP1	0.238712	2.6E-06	2.43E-05
GSG2	0.238718	2.6E-06	2.42E-05

FAM45B	0.238725	2.6E-06	2.42E-05
PLIN5	0.238751	2.59E-06	2.42E-05
C14orf115	0.238843	2.57E-06	2.4E-05
C22orf28	0.238898	2.55E-06	2.39E-05
NIPAL1	0.23921	2.48E-06	2.32E-05
SPPL2A	0.239272	2.46E-06	2.31E-05
NOXA1	0.239329	2.45E-06	2.3E-05
AKAP7	0.239455	2.42E-06	2.27E-05
C10orf81	0.239674	2.36E-06	2.23E-05
MS4A1	0.239731	2.35E-06	2.22E-05
C14orf147	0.239753	2.35E-06	2.21E-05
VRK1	0.239839	2.33E-06	2.2E-05
MMAB	0.239898	2.31E-06	2.19E-05
ZDHHC16	0.2401	2.27E-06	2.15E-05
SULT1A2	0.240131	2.26E-06	2.14E-05
GALK2	0.240143	2.26E-06	2.14E-05
KIAA0101	0.240176	2.25E-06	2.13E-05
LOC38933	0.240186	2.25E-06	2.13E-05
KBTBD8	0.240313	2.22E-06	2.11E-05
TMEM82	0.240444	2.19E-06	2.08E-05
RPS6KA5	0.240494	2.18E-06	2.08E-05
TNFRSF13	0.240633	2.15E-06	2.05E-05
TFG	0.240658	2.14E-06	2.05E-05
MRPS11	0.240708	2.13E-06	2.04E-05
DLG3	0.24106	2.06E-06	1.97E-05
PSMB8	0.241105	2.05E-06	1.97E-05
SIRPG	0.241136	2.04E-06	1.96E-05
CD247	0.241288	2.01E-06	1.93E-05
RTP4	0.241454	1.98E-06	1.91E-05
MANF	0.241488	1.97E-06	1.9E-05
IVD	0.241521	1.96E-06	1.9E-05
SAMM50	0.241531	1.96E-06	1.9E-05
TUBG1	0.241573	1.95E-06	1.89E-05
AGPAT9	0.241594	1.95E-06	1.89E-05
RND1	0.241723	1.93E-06	1.87E-05
SMPDL3B	0.241746	1.92E-06	1.86E-05
CD244	0.24195	1.88E-06	1.83E-05
NADK	0.242025	1.87E-06	1.82E-05
TMEM9B	0.242085	1.86E-06	1.81E-05
IL12RB1	0.242103	1.85E-06	1.81E-05
FDXR	0.242264	1.82E-06	1.78E-05
JAK2	0.242275	1.82E-06	1.78E-05
CIDEC	0.24247	1.79E-06	1.75E-05
ALDOA	0.242481	1.78E-06	1.75E-05
PARP3	0.242511	1.78E-06	1.74E-05
C9orf82	0.242609	1.76E-06	1.73E-05
TNIP1	0.242664	1.75E-06	1.72E-05
GSTZ1	0.242762	1.73E-06	1.71E-05
MCM5	0.242932	1.7E-06	1.68E-05
SHQ1	0.242945	1.7E-06	1.68E-05
QSOX1	0.243063	1.68E-06	1.67E-05
TNK1	0.243068	1.68E-06	1.67E-05
SAMD9L	0.243104	1.68E-06	1.66E-05
PIM3	0.243105	1.67E-06	1.66E-05
TMEM156	0.243253	1.65E-06	1.64E-05
PROM2	0.243274	1.65E-06	1.64E-05
BAD	0.243332	1.64E-06	1.63E-05
BCAT2	0.243414	1.62E-06	1.62E-05

FAM195A	0.243424	1.62E-06	1.62E-05
HAS3	0.243436	1.62E-06	1.62E-05
GNA11	0.243529	1.6E-06	1.6E-05
SLC17A8	0.243643	1.59E-06	1.59E-05
ENKUR	0.244038	1.52E-06	1.53E-05
TMEM30B	0.244151	1.51E-06	1.52E-05
SMS	0.244177	1.5E-06	1.51E-05
LRRC31	0.244289	1.49E-06	1.5E-05
XBP1	0.2444	1.47E-06	1.48E-05
PIM2	0.244498	1.45E-06	1.47E-05
ATPAF2	0.244633	1.43E-06	1.45E-05
SPA17	0.244657	1.43E-06	1.45E-05
RAC2	0.24478	1.41E-06	1.43E-05
ENDOD1	0.244898	1.4E-06	1.42E-05
YIF1B	0.244958	1.39E-06	1.41E-05
KCNA6	0.245006	1.38E-06	1.41E-05
PSME1	0.24506	1.37E-06	1.4E-05
SERPINB7	0.2451	1.37E-06	1.4E-05
SDHD	0.245143	1.36E-06	1.39E-05
HIGD2A	0.245208	1.35E-06	1.38E-05
OR2W3	0.245238	1.35E-06	1.38E-05
ITGB7	0.2454	1.33E-06	1.36E-05
UGT2B7	0.245555	1.31E-06	1.34E-05
ZWINT	0.245627	1.3E-06	1.33E-05
IKBKE	0.245864	1.26E-06	1.31E-05
CDCA8	0.24633	1.21E-06	1.25E-05
UQCRQ	0.246625	1.17E-06	1.22E-05
MTHFR	0.246715	1.16E-06	1.21E-05
CHCHD4	0.246724	1.16E-06	1.21E-05
LSM12	0.246889	1.14E-06	1.19E-05
SORD	0.246943	1.13E-06	1.19E-05
PGP	0.247185	1.1E-06	1.16E-05
HOXB13	0.24722	1.1E-06	1.16E-05
NBL1	0.247426	1.08E-06	1.13E-05
C12orf75	0.24747	1.07E-06	1.13E-05
SEC24A	0.24747	1.07E-06	1.13E-05
AAGAB	0.247583	1.06E-06	1.12E-05
PLCL2	0.247669	1.05E-06	1.11E-05
CCNB1	0.247685	1.05E-06	1.11E-05
OTOP2	0.247791	1.04E-06	1.1E-05
ALDOB	0.247845	1.03E-06	1.09E-05
RAP1B	0.247884	1.03E-06	1.09E-05
GRAP2	0.248017	1.01E-06	1.08E-05
SNRNP25	0.248066	1.01E-06	1.07E-05
CDC20	0.248301	9.84E-07	1.05E-05
SH2D1B	0.248356	9.78E-07	1.05E-05
SGPP1	0.248434	9.71E-07	1.04E-05
RGS13	0.248661	9.48E-07	1.02E-05
COX6A1	0.248677	9.46E-07	1.02E-05
C2orf79	0.248857	9.29E-07	9.99E-06
SEMA4B	0.249118	9.04E-07	9.75E-06
IFI27L1	0.249261	8.91E-07	9.64E-06
C14orf142	0.249262	8.91E-07	9.64E-06
DLST	0.249419	8.76E-07	9.52E-06
SH2D4A	0.249467	8.72E-07	9.48E-06
AK2	0.24958	8.62E-07	9.37E-06
LITAF	0.249591	8.61E-07	9.37E-06
TUBAL3	0.249709	8.5E-07	9.26E-06

BLK	0.249725	8.49E-07	9.26E-06
ALDH6A1	0.249733	8.48E-07	9.25E-06
TMEM129	0.249793	8.43E-07	9.21E-06
PTPRCAP	0.249795	8.43E-07	9.21E-06
VPS29	0.249849	8.38E-07	9.16E-06
CD19	0.249924	8.31E-07	9.11E-06
GDPD3	0.250071	8.19E-07	8.97E-06
POLD3	0.250075	8.18E-07	8.97E-06
RORC	0.250089	8.17E-07	8.97E-06
VDAC2	0.250139	8.13E-07	8.94E-06
MS4A8B	0.250213	8.07E-07	8.88E-06
SLC25A11	0.25025	8.04E-07	8.86E-06
CYCS	0.250292	8E-07	8.83E-06
SMPDL3A	0.250469	7.85E-07	8.68E-06
ARHGDIB	0.250974	7.45E-07	8.29E-06
CAST	0.251115	7.34E-07	8.18E-06
ACY1	0.251247	7.24E-07	8.08E-06
ATP6V0D1	0.251452	7.09E-07	7.93E-06
CYP4X1	0.251483	7.06E-07	7.91E-06
DIAPH1	0.251489	7.06E-07	7.91E-06
TMC8	0.25166	6.93E-07	7.78E-06
GFPT1	0.251695	6.91E-07	7.76E-06
ENO1	0.251731	6.88E-07	7.73E-06
CCR6	0.251763	6.86E-07	7.71E-06
RARS	0.251799	6.83E-07	7.69E-06
DYNLT1	0.251874	6.78E-07	7.63E-06
LOC64543	0.251912	6.75E-07	7.6E-06
WDR34	0.251936	6.74E-07	7.59E-06
SREBF1	0.25194	6.73E-07	7.59E-06
CYP2C9	0.25201	6.68E-07	7.55E-06
BNIP1	0.252081	6.63E-07	7.5E-06
PBK	0.252131	6.6E-07	7.47E-06
PGD	0.252217	6.54E-07	7.41E-06
FBXW5	0.252402	6.41E-07	7.29E-06
FAM46A	0.252513	6.34E-07	7.21E-06
PRRG4	0.252513	6.34E-07	7.21E-06
C14orf73	0.252522	6.33E-07	7.21E-06
MEI1	0.252571	6.3E-07	7.19E-06
MAP3K5	0.252606	6.28E-07	7.17E-06
SPINK2	0.252641	6.25E-07	7.15E-06
HSD3B2	0.252683	6.23E-07	7.12E-06
PTPN7	0.25275	6.18E-07	7.08E-06
LPIN1	0.252761	6.18E-07	7.08E-06
GPR55	0.252919	6.07E-07	6.98E-06
RAD51	0.252932	6.06E-07	6.98E-06
SLC6A8	0.253011	6.01E-07	6.92E-06
C14orf64	0.253139	5.93E-07	6.85E-06
ATP5O	0.253293	5.84E-07	6.75E-06
PADI3	0.253357	5.8E-07	6.71E-06
C18orf55	0.253383	5.78E-07	6.69E-06
TEX101	0.253395	5.77E-07	6.69E-06
DOLPP1	0.253401	5.77E-07	6.69E-06
GDPD2	0.253604	5.65E-07	6.57E-06
NCRNA00	0.253711	5.59E-07	6.5E-06
COQ3	0.253907	5.47E-07	6.37E-06
EIF5AL1	0.25391	5.47E-07	6.37E-06
TMX2	0.253993	5.42E-07	6.32E-06
ATP8A1	0.254058	5.38E-07	6.28E-06

AKR1A1	0.254142	5.34E-07	6.23E-06
ACER3	0.254205	5.3E-07	6.2E-06
DDX60	0.254267	5.27E-07	6.16E-06
MTUS1	0.254275	5.26E-07	6.16E-06
ARF6	0.25441	5.19E-07	6.08E-06
CTH	0.254464	5.16E-07	6.06E-06
IDI1	0.254677	5.04E-07	5.95E-06
C4orf33	0.254695	5.03E-07	5.94E-06
ALG6	0.254696	5.03E-07	5.94E-06
COX15	0.254697	5.03E-07	5.94E-06
PDE4C	0.254825	4.96E-07	5.88E-06
TPMT	0.254898	4.92E-07	5.84E-06
PHB2	0.255073	4.83E-07	5.75E-06
TMEM141	0.255097	4.82E-07	5.74E-06
GRSF1	0.25512	4.81E-07	5.73E-06
ERI2	0.255155	4.79E-07	5.71E-06
REEP4	0.255371	4.68E-07	5.6E-06
DUS2L	0.255388	4.67E-07	5.59E-06
C6orf108	0.255472	4.63E-07	5.55E-06
SLC4A10	0.255489	4.62E-07	5.55E-06
INSL5	0.255501	4.62E-07	5.54E-06
FAR1	0.255778	4.48E-07	5.4E-06
MRPL39	0.25597	4.39E-07	5.3E-06
HINT2	0.256012	4.37E-07	5.29E-06
MPI	0.25624	4.27E-07	5.17E-06
MCAT	0.256241	4.27E-07	5.17E-06
SLCO1B3	0.25627	4.25E-07	5.16E-06
USP43	0.256293	4.24E-07	5.15E-06
OSTBETA	0.256365	4.21E-07	5.12E-06
DHRS1	0.256375	4.21E-07	5.12E-06
FAM83E	0.256558	4.12E-07	5.03E-06
CLDN8	0.256564	4.12E-07	5.03E-06
DPF3	0.256605	4.1E-07	5.01E-06
BSPRY	0.256615	4.1E-07	5.01E-06
C19orf33	0.256901	3.98E-07	4.87E-06
IPPK	0.256994	3.94E-07	4.83E-06
SLC35D1	0.257015	3.93E-07	4.82E-06
WDR76	0.257028	3.92E-07	4.82E-06
PFN1	0.257091	3.89E-07	4.79E-06
MAEA	0.257121	3.88E-07	4.78E-06
LGALS9B	0.257382	3.77E-07	4.67E-06
PTGES2	0.257458	3.74E-07	4.64E-06
SDR16C5	0.257538	3.71E-07	4.6E-06
ARHGAP8	0.257604	3.69E-07	4.57E-06
HLA-F	0.257641	3.67E-07	4.56E-06
CHCHD1	0.257659	3.66E-07	4.55E-06
C8orf75	0.25774	3.63E-07	4.52E-06
CIITA	0.257838	3.59E-07	4.49E-06
MDM2	0.258102	3.49E-07	4.37E-06
PVRL3	0.258153	3.47E-07	4.35E-06
CDKN2BAS	0.258301	3.42E-07	4.28E-06
ABCG8	0.258319	3.41E-07	4.27E-06
C9orf100	0.25837	3.39E-07	4.25E-06
NMNAT1	0.258378	3.39E-07	4.25E-06
C10orf57	0.258382	3.39E-07	4.25E-06
GALNT3	0.258415	3.38E-07	4.24E-06
FAM107B	0.258436	3.37E-07	4.23E-06
APOBEC1	0.258493	3.35E-07	4.21E-06

RDH13	0.258661	3.29E-07	4.15E-06
ANKRD56	0.258669	3.29E-07	4.14E-06
RETNLB	0.258802	3.24E-07	4.09E-06
NLRC5	0.258992	3.17E-07	4.02E-06
TMEM53	0.259265	3.08E-07	3.91E-06
TAP1	0.259409	3.03E-07	3.86E-06
SYT13	0.259452	3.02E-07	3.85E-06
EXOC6	0.259576	2.98E-07	3.8E-06
GRK6	0.259746	2.92E-07	3.74E-06
CD3D	0.2599	2.88E-07	3.69E-06
BLVRB	0.25996	2.86E-07	3.67E-06
FBXO6	0.259963	2.86E-07	3.67E-06
AP2B1	0.26008	2.82E-07	3.63E-06
C8orf80	0.260537	2.68E-07	3.48E-06
NMUR2	0.260539	2.68E-07	3.48E-06
TNFSF10	0.260622	2.66E-07	3.46E-06
CDKL1	0.260762	2.62E-07	3.42E-06
TRPA1	0.260844	2.59E-07	3.39E-06
MT1E	0.260922	2.57E-07	3.37E-06
APOL6	0.260964	2.56E-07	3.36E-06
HGD	0.261033	2.54E-07	3.34E-06
C14orf2	0.261083	2.53E-07	3.33E-06
SEC13	0.261138	2.51E-07	3.31E-06
LACTB	0.261346	2.46E-07	3.24E-06
TRABD	0.261448	2.43E-07	3.21E-06
SLC40A1	0.261789	2.34E-07	3.1E-06
GDA	0.261991	2.29E-07	3.05E-06
ABHD14B	0.262035	2.28E-07	3.03E-06
TREX1	0.262131	2.25E-07	3.01E-06
PHF7	0.26223	2.23E-07	2.98E-06
MID1IP1	0.262271	2.22E-07	2.97E-06
ERLIN1	0.262329	2.21E-07	2.95E-06
TSPAN3	0.262355	2.2E-07	2.95E-06
AKR7A2	0.262539	2.16E-07	2.89E-06
RAET1E	0.262616	2.14E-07	2.88E-06
NUDT2	0.262752	2.11E-07	2.84E-06
ARSJ	0.262976	2.05E-07	2.78E-06
UNC13D	0.263346	1.97E-07	2.67E-06
C8G	0.263486	1.94E-07	2.64E-06
IL17REL	0.263697	1.9E-07	2.58E-06
TBC1D2	0.263703	1.9E-07	2.58E-06
IL22RA2	0.263773	1.88E-07	2.57E-06
UQCRHL	0.263838	1.87E-07	2.55E-06
MVK	0.264017	1.83E-07	2.51E-06
EIF5A	0.264127	1.81E-07	2.48E-06
SH2D7	0.264205	1.79E-07	2.46E-06
PIK3R3	0.26421	1.79E-07	2.46E-06
WIBG	0.264233	1.79E-07	2.46E-06
AMN	0.264315	1.77E-07	2.44E-06
CS	0.264404	1.75E-07	2.43E-06
MRPL27	0.264426	1.75E-07	2.42E-06
SMAGP	0.264523	1.73E-07	2.4E-06
NXF3	0.264659	1.71E-07	2.37E-06
C11orf24	0.264821	1.68E-07	2.33E-06
CC2D1A	0.264989	1.64E-07	2.29E-06
GPR128	0.265077	1.63E-07	2.27E-06
NDUFV2	0.265229	1.6E-07	2.24E-06
JOSD1	0.265328	1.58E-07	2.23E-06

SAA4	0.265346	1.58E-07	2.22E-06
AURKAIP1	0.265466	1.56E-07	2.2E-06
DENND2A	0.265511	1.55E-07	2.19E-06
IRF4	0.265542	1.55E-07	2.19E-06
C2CD4B	0.26571	1.52E-07	2.15E-06
FFAR2	0.2659	1.49E-07	2.11E-06
MLEC	0.26641	1.4E-07	2E-06
EPN3	0.26676	1.35E-07	1.93E-06
ARHGEF16	0.266956	1.32E-07	1.9E-06
KIAA0114	0.267012	1.31E-07	1.89E-06
MRPL54	0.267174	1.29E-07	1.86E-06
CDKN1A	0.267392	1.26E-07	1.82E-06
OXSM	0.267451	1.25E-07	1.81E-06
MED11	0.267529	1.24E-07	1.8E-06
ALDH3B2	0.267732	1.21E-07	1.76E-06
KDELR3	0.267762	1.21E-07	1.76E-06
SRD5A3	0.268026	1.17E-07	1.71E-06
SEC1	0.268028	1.17E-07	1.71E-06
DHDDS	0.268089	1.16E-07	1.7E-06
ATP5J	0.268138	1.16E-07	1.7E-06
MT1M	0.268175	1.15E-07	1.69E-06
DUOX1	0.2682	1.15E-07	1.69E-06
VWA5A	0.268207	1.15E-07	1.69E-06
S100A14	0.268513	1.11E-07	1.63E-06
RHOV	0.269048	1.04E-07	1.55E-06
MCTP2	0.269064	1.04E-07	1.54E-06
CNDP2	0.269081	1.04E-07	1.54E-06
ZDHHC12	0.269171	1.03E-07	1.53E-06
PTGER2	0.269347	1.01E-07	1.5E-06
EHD4	0.269446	9.98E-08	1.49E-06
ATP1B1	0.269535	9.88E-08	1.48E-06
ICAM3	0.269712	9.69E-08	1.45E-06
KCNJ4	0.269835	9.55E-08	1.43E-06
SDHB	0.270019	9.35E-08	1.41E-06
ZNF488	0.270105	9.26E-08	1.4E-06
BCMO1	0.27024	9.12E-08	1.38E-06
TP53I11	0.270249	9.11E-08	1.38E-06
FRMD3	0.270358	9E-08	1.36E-06
PLCH1	0.270405	8.95E-08	1.36E-06
LYPD5	0.270599	8.76E-08	1.33E-06
GBA3	0.270711	8.65E-08	1.32E-06
CXCR6	0.270715	8.64E-08	1.32E-06
PSME2	0.270874	8.49E-08	1.3E-06
B3GNT7	0.271151	8.23E-08	1.26E-06
IDH2	0.271223	8.16E-08	1.25E-06
EHF	0.271227	8.16E-08	1.25E-06
RASL11A	0.271228	8.15E-08	1.25E-06
IFNGR1	0.271394	8E-08	1.23E-06
ENOSF1	0.271498	7.91E-08	1.22E-06
VNN1	0.271523	7.89E-08	1.22E-06
GLRX	0.271633	7.79E-08	1.21E-06
UBA7	0.271759	7.68E-08	1.2E-06
FAM57A	0.271787	7.65E-08	1.19E-06
DNAJC22	0.271857	7.59E-08	1.19E-06
LOC96610	0.271922	7.53E-08	1.18E-06
ABCB11	0.272014	7.46E-08	1.17E-06
TSPAN8	0.272356	7.17E-08	1.13E-06
FUT8	0.272427	7.11E-08	1.12E-06

BTBD6	0.272773	6.84E-08	1.08E-06
HNRNPF	0.272796	6.82E-08	1.08E-06
S100A6	0.272874	6.76E-08	1.07E-06
NCRNA00	0.272968	6.68E-08	1.06E-06
ANKRD30I	0.273009	6.65E-08	1.06E-06
ATP10B	0.273015	6.65E-08	1.06E-06
ACADSB	0.27322	6.49E-08	1.04E-06
MYCBP	0.273419	6.35E-08	1.02E-06
GNG5	0.273446	6.33E-08	1.02E-06
CASP3	0.273484	6.3E-08	1.01E-06
FUT6	0.273636	6.19E-08	9.98E-07
EPHA10	0.273745	6.11E-08	9.87E-07
IL18	0.273844	6.04E-08	9.77E-07
TTC38	0.273955	5.97E-08	9.65E-07
KIAA0513	0.273969	5.96E-08	9.65E-07
ZNF552	0.274359	5.7E-08	9.25E-07
ATP5D	0.274564	5.56E-08	9.06E-07
ST6GALNA	0.274707	5.47E-08	8.94E-07
ESCO2	0.274752	5.44E-08	8.91E-07
DGKA	0.27489	5.36E-08	8.8E-07
TUBA1C	0.274937	5.33E-08	8.76E-07
SLC6A7	0.275112	5.22E-08	8.61E-07
ERI1	0.275357	5.08E-08	8.41E-07
FCRL4	0.275731	4.86E-08	8.08E-07
C21orf33	0.275961	4.73E-08	7.87E-07
MMAA	0.276025	4.7E-08	7.84E-07
TCF7L2	0.276273	4.56E-08	7.63E-07
ZG16B	0.276622	4.38E-08	7.37E-07
ENDOG	0.276633	4.38E-08	7.37E-07
GLTP	0.276998	4.2E-08	7.1E-07
NDUFS7	0.27704	4.18E-08	7.08E-07
DIRC2	0.277255	4.07E-08	6.91E-07
CEP55	0.277352	4.03E-08	6.84E-07
TNFAIP8	0.277535	3.94E-08	6.73E-07
GNAQ	0.277609	3.91E-08	6.68E-07
C1orf212	0.277656	3.89E-08	6.65E-07
KCNK1	0.277956	3.75E-08	6.44E-07
MMP15	0.278046	3.71E-08	6.38E-07
AKR7A3	0.27821	3.64E-08	6.27E-07
AACS	0.278344	3.59E-08	6.19E-07
RPL10L	0.278441	3.54E-08	6.13E-07
TMEM173	0.278524	3.51E-08	6.08E-07
PTPN18	0.278622	3.47E-08	6.02E-07
FXN	0.278679	3.45E-08	5.99E-07
TMEM106	0.278731	3.43E-08	5.95E-07
ATP13A4	0.278746	3.42E-08	5.95E-07
LIPH	0.278858	3.38E-08	5.88E-07
IL26	0.278875	3.37E-08	5.87E-07
KIAA1324	0.278905	3.36E-08	5.85E-07
STEAP1	0.279574	3.1E-08	5.45E-07
FUT4	0.27982	3.01E-08	5.3E-07
ADH6	0.280249	2.87E-08	5.05E-07
PGAM4	0.280376	2.82E-08	4.98E-07
LONRF3	0.280447	2.8E-08	4.94E-07
MOCOS	0.280455	2.8E-08	4.94E-07
PARM1	0.280463	2.79E-08	4.94E-07
USP18	0.280588	2.75E-08	4.87E-07
MT1H	0.281432	2.49E-08	4.45E-07

CRB3	0.281498	2.47E-08	4.42E-07
CYP2J2	0.281521	2.47E-08	4.42E-07
PSMA5	0.281708	2.41E-08	4.33E-07
ORMDL2	0.281835	2.38E-08	4.27E-07
ATP2A3	0.282012	2.33E-08	4.21E-07
KIAA1522	0.282292	2.25E-08	4.08E-07
AP4B1	0.282376	2.23E-08	4.04E-07
RHOA	0.282901	2.09E-08	3.81E-07
RARRES3	0.282945	2.08E-08	3.79E-07
ELL3	0.283007	2.07E-08	3.77E-07
GPR172B	0.28301	2.07E-08	3.77E-07
FBXO16	0.283031	2.06E-08	3.77E-07
SOCS1	0.283191	2.02E-08	3.7E-07
PEX11A	0.28321	2.02E-08	3.7E-07
TUBA1B	0.283342	1.98E-08	3.65E-07
ART3	0.283375	1.98E-08	3.64E-07
NUDT16	0.283377	1.98E-08	3.64E-07
FDPS	0.283441	1.96E-08	3.62E-07
MTP18	0.283498	1.95E-08	3.6E-07
KCNJ16	0.283505	1.95E-08	3.6E-07
NUDT22	0.283544	1.94E-08	3.59E-07
C3orf55	0.283547	1.94E-08	3.59E-07
PLEK2	0.283799	1.88E-08	3.5E-07
FAM134B	0.283935	1.85E-08	3.45E-07
FAM55B	0.28399	1.84E-08	3.43E-07
CDC42EP2	0.284299	1.77E-08	3.32E-07
TCEA3	0.284358	1.76E-08	3.3E-07
TPK1	0.284456	1.74E-08	3.27E-07
SHMT1	0.284462	1.74E-08	3.27E-07
TRIM10	0.284624	1.7E-08	3.21E-07
FHL2	0.284682	1.69E-08	3.19E-07
CDX1	0.284833	1.66E-08	3.14E-07
CXCL1	0.284899	1.65E-08	3.13E-07
C6orf192	0.285097	1.61E-08	3.06E-07
NR1H4	0.285153	1.6E-08	3.04E-07
S100P	0.285279	1.57E-08	3E-07
UQCRC2	0.28531	1.57E-08	2.99E-07
GPR25	0.285457	1.54E-08	2.95E-07
SLC35A1	0.28569	1.5E-08	2.87E-07
CYTSB	0.285719	1.49E-08	2.87E-07
CMPK1	0.285727	1.49E-08	2.87E-07
CD79A	0.28578	1.48E-08	2.85E-07
MFSD4	0.285887	1.46E-08	2.82E-07
SLC25A24	0.285947	1.45E-08	2.81E-07
C14orf184	0.28653	1.35E-08	2.64E-07
C15orf21	0.286825	1.31E-08	2.56E-07
RIMS3	0.286892	1.3E-08	2.54E-07
COQ9	0.287039	1.27E-08	2.5E-07
REG3A	0.287122	1.26E-08	2.48E-07
ACY3	0.287346	1.23E-08	2.42E-07
NDUFV1	0.287567	1.19E-08	2.36E-07
SHROOM3	0.287588	1.19E-08	2.36E-07
GLRX5	0.287724	1.17E-08	2.32E-07
CCDC60	0.287994	1.13E-08	2.26E-07
FAM81A	0.288128	1.11E-08	2.22E-07
TTLL12	0.28832	1.09E-08	2.18E-07
SLC31A1	0.288564	1.06E-08	2.12E-07
AK7	0.288975	1.01E-08	2.03E-07

SPIB	0.289124	9.87E-09	1.99E-07
LOC11511	0.289157	9.83E-09	1.99E-07
CA9	0.289242	9.73E-09	1.97E-07
GAPDH	0.289286	9.68E-09	1.96E-07
BRI3BP	0.28968	9.22E-09	1.88E-07
RER1	0.289969	8.9E-09	1.82E-07
COX10	0.290656	8.18E-09	1.69E-07
SCIN	0.290849	7.99E-09	1.65E-07
UQCRH	0.290885	7.95E-09	1.65E-07
CDC42SE2	0.291162	7.69E-09	1.6E-07
PPARGC1E	0.291216	7.64E-09	1.6E-07
SEMG1	0.29146	7.41E-09	1.55E-07
CTSS	0.291511	7.36E-09	1.54E-07
XK	0.291577	7.3E-09	1.54E-07
MT1G	0.291686	7.21E-09	1.52E-07
FAM120A	0.291698	7.19E-09	1.52E-07
CHGA	0.291747	7.15E-09	1.51E-07
RHOC	0.291772	7.13E-09	1.51E-07
SLC9A1	0.291852	7.06E-09	1.49E-07
RNPEP	0.291877	7.04E-09	1.49E-07
C1orf151	0.291913	7.01E-09	1.49E-07
IL1A	0.291922	7E-09	1.49E-07
NDUFS2	0.291951	6.97E-09	1.48E-07
MFSD6L	0.292011	6.92E-09	1.48E-07
TBC1D10A	0.292243	6.73E-09	1.44E-07
MOSC1	0.292265	6.71E-09	1.43E-07
BACE2	0.292678	6.37E-09	1.37E-07
C2CD4A	0.292794	6.28E-09	1.35E-07
LSS	0.293352	5.86E-09	1.27E-07
NPY6R	0.293511	5.75E-09	1.25E-07
ERGIC1	0.293814	5.53E-09	1.2E-07
DHFR	0.294631	5E-09	1.1E-07
ATPIF1	0.29476	4.92E-09	1.08E-07
C14orf80	0.294934	4.81E-09	1.06E-07
CXCL3	0.295067	4.73E-09	1.05E-07
TYMS	0.295076	4.73E-09	1.05E-07
H2AFZ	0.29509	4.72E-09	1.05E-07
STX18	0.295148	4.69E-09	1.04E-07
SGMS1	0.29521	4.65E-09	1.04E-07
SFXN2	0.295321	4.59E-09	1.02E-07
GHITM	0.295477	4.5E-09	1.01E-07
TRIM16	0.295666	4.39E-09	9.86E-08
GALNT7	0.295709	4.37E-09	9.83E-08
CPNE5	0.295763	4.34E-09	9.78E-08
TMEM92	0.296051	4.19E-09	9.47E-08
OMA1	0.296078	4.17E-09	9.46E-08
LRRIQ4	0.296187	4.11E-09	9.35E-08
TMEM102	0.296247	4.08E-09	9.3E-08
IMPA2	0.296591	3.91E-09	8.98E-08
TMIGD2	0.296804	3.81E-09	8.77E-08
PADI2	0.29699	3.72E-09	8.57E-08
TXNDC17	0.297123	3.66E-09	8.44E-08
HSD17B2	0.297176	3.63E-09	8.4E-08
RAG1AP1	0.2972	3.62E-09	8.39E-08
RUSC1	0.297273	3.59E-09	8.32E-08
HECTD3	0.297548	3.47E-09	8.08E-08
GALE	0.297693	3.4E-09	7.94E-08
GIPC1	0.297739	3.38E-09	7.92E-08

PFKP	0.297983	3.28E-09	7.73E-08
CCDC85C	0.298104	3.23E-09	7.62E-08
TRIM7	0.29812	3.22E-09	7.61E-08
PDXDC1	0.298217	3.18E-09	7.54E-08
CENPM	0.298429	3.1E-09	7.35E-08
KIAA0125	0.298508	3.07E-09	7.29E-08
CKMT1A	0.298741	2.98E-09	7.11E-08
VSIG1	0.299376	2.75E-09	6.64E-08
REG1B	0.299542	2.69E-09	6.51E-08
IL17A	0.299966	2.55E-09	6.2E-08
UBAC1	0.299972	2.55E-09	6.2E-08
MARVELD:	0.299992	2.54E-09	6.19E-08
EMP2	0.300013	2.54E-09	6.18E-08
KCTD9	0.300419	2.41E-09	5.89E-08
MGST1	0.300797	2.29E-09	5.65E-08
SAA2	0.300835	2.28E-09	5.63E-08
SFN	0.300994	2.24E-09	5.54E-08
C15orf58	0.301162	2.19E-09	5.43E-08
LOC28457	0.301166	2.19E-09	5.43E-08
TMIGD1	0.301217	2.17E-09	5.41E-08
JSRP1	0.30133	2.14E-09	5.34E-08
PDLIM1	0.301387	2.13E-09	5.31E-08
STARD4	0.301493	2.1E-09	5.25E-08
SLITRK6	0.301537	2.09E-09	5.22E-08
CAPN9	0.301604	2.07E-09	5.2E-08
LETM1	0.301691	2.05E-09	5.15E-08
ADAM15	0.301764	2.03E-09	5.11E-08
ASPHD2	0.30184	2.01E-09	5.08E-08
KLK15	0.302121	1.94E-09	4.91E-08
CALML4	0.302285	1.9E-09	4.82E-08
RETSAT	0.302642	1.81E-09	4.63E-08
HMGCR	0.302917	1.75E-09	4.49E-08
KITLG	0.303078	1.71E-09	4.41E-08
CLN3	0.303159	1.69E-09	4.37E-08
SFTPA2	0.303184	1.69E-09	4.36E-08
C11orf75	0.303225	1.68E-09	4.34E-08
TOR1B	0.303248	1.67E-09	4.34E-08
FCRL5	0.303537	1.61E-09	4.21E-08
GP9	0.303963	1.53E-09	4E-08
GSTM4	0.30419	1.48E-09	3.9E-08
TRIM36	0.304559	1.41E-09	3.74E-08
CHST4	0.304638	1.4E-09	3.7E-08
MUDENG	0.304699	1.39E-09	3.68E-08
ATP5A1	0.304724	1.38E-09	3.68E-08
SLAMF6	0.304752	1.38E-09	3.67E-08
SERPINA1	0.304905	1.35E-09	3.61E-08
MAOA	0.304944	1.34E-09	3.6E-08
PKP2	0.3051	1.32E-09	3.54E-08
DNAJC1	0.305207	1.3E-09	3.51E-08
MRPL35	0.305264	1.29E-09	3.49E-08
MMP28	0.305344	1.28E-09	3.46E-08
HIGD1A	0.305654	1.23E-09	3.35E-08
LRRC19	0.305718	1.22E-09	3.33E-08
SLC28A3	0.305822	1.2E-09	3.29E-08
COQ4	0.305824	1.2E-09	3.29E-08
C9orf167	0.306304	1.13E-09	3.11E-08
ANG	0.306457	1.1E-09	3.07E-08
PSMD9	0.306648	1.08E-09	3E-08

CREB3L1	0.306883	1.04E-09	2.92E-08
GALNTL6	0.306992	1.03E-09	2.89E-08
PNP	0.307097	1.02E-09	2.86E-08
SLC25A10	0.307134	1.01E-09	2.85E-08
MLPH	0.307215	1E-09	2.82E-08
TTC39B	0.307249	9.95E-10	2.81E-08
CBR3	0.307311	9.87E-10	2.79E-08
UGT1A8	0.307447	9.7E-10	2.75E-08
P2RY1	0.307453	9.69E-10	2.75E-08
ABCD3	0.307621	9.48E-10	2.7E-08
DNAJC11	0.308089	8.91E-10	2.55E-08
CWH43	0.3081	8.9E-10	2.55E-08
TMEM125	0.30815	8.84E-10	2.54E-08
SH3BGRL3	0.30825	8.73E-10	2.51E-08
PYY	0.308448	8.5E-10	2.45E-08
TAGLN2	0.308452	8.5E-10	2.45E-08
ST3GAL4	0.308465	8.48E-10	2.45E-08
PLS1	0.308643	8.29E-10	2.41E-08
ANO7	0.308806	8.11E-10	2.37E-08
CAPN5	0.308947	7.96E-10	2.33E-08
TMEM171	0.309225	7.68E-10	2.25E-08
ADH1C	0.30926	7.64E-10	2.25E-08
CXCL17	0.30983	7.09E-10	2.1E-08
FUCA1	0.309949	6.98E-10	2.07E-08
CA7	0.309952	6.97E-10	2.07E-08
ALPI	0.310392	6.58E-10	1.96E-08
TRIM14	0.310505	6.48E-10	1.94E-08
UGT1A10	0.310657	6.35E-10	1.9E-08
ZBTB7C	0.310768	6.26E-10	1.87E-08
SCO1	0.311224	5.89E-10	1.77E-08
LGALS3	0.3117	5.53E-10	1.66E-08
ACVRL1	0.311723	5.51E-10	1.66E-08
ANKRD58	0.311793	5.46E-10	1.65E-08
GCG	0.311915	5.37E-10	1.63E-08
GPD1L	0.312663	4.86E-10	1.48E-08
BIRC3	0.312918	4.7E-10	1.44E-08
TEX11	0.312977	4.66E-10	1.43E-08
SGMS2	0.312978	4.66E-10	1.43E-08
GGT6	0.313025	4.63E-10	1.43E-08
ATP5G3	0.313164	4.55E-10	1.41E-08
COMTD1	0.313257	4.49E-10	1.39E-08
UAP1	0.314128	3.99E-10	1.25E-08
DHRS4L2	0.314498	3.8E-10	1.2E-08
PGM1	0.314541	3.78E-10	1.19E-08
C14orf176	0.314728	3.68E-10	1.17E-08
BDH1	0.314778	3.66E-10	1.16E-08
FAM83F	0.314799	3.65E-10	1.16E-08
C1orf210	0.314921	3.59E-10	1.14E-08
GNE	0.315255	3.43E-10	1.09E-08
FAM83A	0.315468	3.33E-10	1.07E-08
TRPM4	0.315811	3.18E-10	1.03E-08
KLRB1	0.315891	3.15E-10	1.02E-08
LOC72860	0.316053	3.08E-10	9.97E-09
CDC42EP5	0.316118	3.05E-10	9.9E-09
ATP5G1	0.316642	2.84E-10	9.28E-09
PSMB10	0.31679	2.79E-10	9.13E-09
TPM3	0.316888	2.75E-10	9.03E-09
FABP2	0.317394	2.57E-10	8.52E-09

C14orf159	0.31741	2.56E-10	8.51E-09
FDFT1	0.31747	2.54E-10	8.47E-09
PCCB	0.317482	2.54E-10	8.47E-09
RAB27A	0.317504	2.53E-10	8.46E-09
TMEM229I	0.31791	2.39E-10	8.03E-09
AKAP5	0.318082	2.34E-10	7.86E-09
SESN2	0.318089	2.34E-10	7.86E-09
CA1	0.318137	2.32E-10	7.84E-09
ACSM3	0.318245	2.29E-10	7.74E-09
CCDC68	0.318383	2.24E-10	7.63E-09
CDCP1	0.318978	2.07E-10	7.1E-09
GABRP	0.319209	2E-10	6.9E-09
BPNT1	0.319376	1.96E-10	6.77E-09
FAM45A	0.319784	1.85E-10	6.44E-09
NUDT6	0.3199	1.82E-10	6.35E-09
TSPO	0.319953	1.81E-10	6.32E-09
AFG3L2	0.32	1.8E-10	6.29E-09
DHRS4	0.32004	1.79E-10	6.27E-09
DENND2D	0.320342	1.72E-10	6.04E-09
NRAP	0.320395	1.7E-10	6.01E-09
PLA2G10	0.320478	1.68E-10	5.95E-09
TOR2A	0.320618	1.65E-10	5.85E-09
GFI1	0.320678	1.64E-10	5.81E-09
ECHS1	0.320772	1.62E-10	5.75E-09
PI3	0.320825	1.61E-10	5.73E-09
TXN2	0.320839	1.6E-10	5.73E-09
NDUFA6	0.321232	1.52E-10	5.45E-09
WDR55	0.321262	1.51E-10	5.44E-09
NAT2	0.321577	1.45E-10	5.24E-09
UQCR10	0.321729	1.42E-10	5.14E-09
BCL3	0.321923	1.38E-10	5.03E-09
COX8A	0.322646	1.25E-10	4.59E-09
CIB1	0.32273	1.23E-10	4.56E-09
DEGS2	0.322785	1.22E-10	4.53E-09
SULT1B1	0.323031	1.18E-10	4.39E-09
DERL3	0.323545	1.1E-10	4.15E-09
FER1L6	0.323697	1.08E-10	4.09E-09
SLC25A3	0.324096	1.02E-10	3.89E-09
TFF1	0.325158	8.8E-11	3.38E-09
TST	0.326254	7.54E-11	2.94E-09
PECI	0.326304	7.49E-11	2.93E-09
EGF	0.326782	7E-11	2.74E-09
ATOH1	0.32714	6.66E-11	2.62E-09
CKAP4	0.327213	6.59E-11	2.61E-09
ABHD5	0.327952	5.94E-11	2.36E-09
SLC35A3	0.328621	5.4E-11	2.16E-09
SCAMP2	0.328782	5.28E-11	2.12E-09
TSPAN13	0.328824	5.25E-11	2.11E-09
FAM55D	0.32884	5.23E-11	2.11E-09
MECOM	0.328884	5.2E-11	2.1E-09
SDCBP2	0.328912	5.18E-11	2.1E-09
ATP8B1	0.328941	5.16E-11	2.1E-09
PRELID2	0.328998	5.12E-11	2.09E-09
HPSE	0.329413	4.82E-11	1.99E-09
TPSG1	0.329434	4.81E-11	1.99E-09
C1orf125	0.329483	4.78E-11	1.98E-09
MST1R	0.32949	4.77E-11	1.98E-09
FUT3	0.329594	4.7E-11	1.97E-09

MTNR1A	0.329675	4.65E-11	1.95E-09
VILL	0.329955	4.47E-11	1.88E-09
LGALS2	0.330044	4.41E-11	1.86E-09
DNM2	0.330791	3.96E-11	1.69E-09
ENTPD8	0.330953	3.87E-11	1.65E-09
SLC22A18A	0.331157	3.76E-11	1.62E-09
DHCR24	0.331932	3.37E-11	1.46E-09
IDH3A	0.331964	3.35E-11	1.46E-09
KLK1	0.332312	3.19E-11	1.4E-09
BCL2L14	0.332429	3.13E-11	1.38E-09
TPI1	0.332505	3.1E-11	1.37E-09
AVPI1	0.33299	2.89E-11	1.29E-09
C9orf152	0.333037	2.87E-11	1.29E-09
TMC5	0.333132	2.83E-11	1.27E-09
GNPNAT1	0.333646	2.63E-11	1.2E-09
HNRNPAB	0.334309	2.39E-11	1.09E-09
GSR	0.334661	2.27E-11	1.04E-09
FUT2	0.334952	2.18E-11	1E-09
B2M	0.334997	2.16E-11	9.97E-10
SLC25A5	0.335153	2.11E-11	9.8E-10
C6orf136	0.335186	2.1E-11	9.77E-10
ATG4A	0.335294	2.07E-11	9.67E-10
CRELD2	0.335419	2.03E-11	9.51E-10
FXYD3	0.335484	2.02E-11	9.45E-10
PANK3	0.335619	1.98E-11	9.29E-10
RNF19B	0.335683	1.96E-11	9.22E-10
BTNL3	0.335926	1.89E-11	8.94E-10
ATP5F1	0.33593	1.89E-11	8.94E-10
OASL	0.336078	1.85E-11	8.79E-10
CANT1	0.336295	1.79E-11	8.58E-10
LPCAT4	0.336424	1.76E-11	8.46E-10
TUBB2C	0.336462	1.75E-11	8.43E-10
BIK	0.338112	1.37E-11	6.74E-10
ETFDH	0.338396	1.32E-11	6.5E-10
HRASLS2	0.338458	1.3E-11	6.47E-10
L1TD1	0.338693	1.26E-11	6.28E-10
MPDU1	0.338848	1.23E-11	6.19E-10
TK1	0.339114	1.18E-11	5.96E-10
NUBP1	0.339202	1.17E-11	5.9E-10
PTGDR	0.339246	1.16E-11	5.88E-10
CLCA3P	0.340176	1.01E-11	5.15E-10
EGLN3	0.3402	1.01E-11	5.15E-10
TP53I3	0.340214	1.01E-11	5.15E-10
OXNAD1	0.340544	9.59E-12	4.93E-10
APEH	0.340864	9.15E-12	4.74E-10
REG1A	0.340985	8.99E-12	4.66E-10
LGALS9C	0.341927	7.81E-12	4.11E-10
C2orf72	0.342535	7.14E-12	3.76E-10
ETFA	0.344193	5.57E-12	2.99E-10
CLDN7	0.344557	5.27E-12	2.84E-10
LGALS3BP	0.345535	4.55E-12	2.48E-10
RAB27B	0.345599	4.51E-12	2.46E-10
RNF186	0.345928	4.29E-12	2.36E-10
MGC29506	0.346695	3.82E-12	2.12E-10
SLC27A4	0.347269	3.5E-12	1.96E-10
GJA9	0.347381	3.44E-12	1.93E-10
LGALS4	0.347858	3.2E-12	1.81E-10
UGT8	0.347986	3.14E-12	1.78E-10

TCN1	0.348123	3.08E-12	1.75E-10
TNIP3	0.348669	2.83E-12	1.62E-10
ADAM6	0.348949	2.71E-12	1.56E-10
CCRL2	0.348979	2.7E-12	1.56E-10
C19orf57	0.34898	2.7E-12	1.56E-10
SPATA18	0.349016	2.69E-12	1.56E-10
SUCLG2	0.349021	2.68E-12	1.56E-10
ZDHHC3	0.349462	2.51E-12	1.47E-10
REP15	0.34967	2.43E-12	1.43E-10
INSC	0.350243	2.23E-12	1.32E-10
MYEOV	0.351496	1.84E-12	1.09E-10
SAR1B	0.3515	1.84E-12	1.09E-10
NFKBIZ	0.351826	1.75E-12	1.05E-10
PLCE1	0.352108	1.67E-12	1.01E-10
TJP3	0.352288	1.62E-12	9.82E-11
E2F2	0.35288	1.48E-12	8.99E-11
BCAS1	0.353523	1.34E-12	8.16E-11
ACO2	0.353932	1.26E-12	7.72E-11
MOGAT2	0.354071	1.23E-12	7.59E-11
C2orf88	0.354291	1.19E-12	7.36E-11
DNASE1L3	0.354374	1.18E-12	7.29E-11
CDC42EP1	0.354873	1.09E-12	6.77E-11
RAB26	0.355212	1.03E-12	6.44E-11
SERPINB1	0.35559	9.73E-13	6.13E-11
TRIM15	0.356453	8.5E-13	5.44E-11
F3	0.356469	8.48E-13	5.44E-11
CNNM4	0.356571	8.35E-13	5.37E-11
FOXA3	0.356653	8.24E-13	5.32E-11
ZC3H12C	0.357727	6.96E-13	4.59E-11
PAFAH2	0.357905	6.77E-13	4.5E-11
STARD5	0.359552	5.22E-13	3.56E-11
C5orf32	0.359693	5.1E-13	3.49E-11
PGAM1	0.359926	4.92E-13	3.39E-11
POC1A	0.360151	4.75E-13	3.28E-11
SGPP2	0.360608	4.41E-13	3.06E-11
TSPAN15	0.361119	4.07E-13	2.83E-11
CRYM	0.361437	3.87E-13	2.71E-11
ACO1	0.36146	3.85E-13	2.71E-11
PTPRH	0.361468	3.85E-13	2.71E-11
FGFBP1	0.36192	3.58E-13	2.54E-11
SGSM3	0.362043	3.51E-13	2.5E-11
SCO2	0.362519	3.25E-13	2.32E-11
PPA2	0.363342	2.85E-13	2.05E-11
NDUFA9	0.363552	2.76E-13	1.99E-11
EIF4E3	0.364111	2.52E-13	1.85E-11
C9orf46	0.364964	2.2E-13	1.63E-11
SLC35C1	0.365052	2.17E-13	1.62E-11
XPNPEP1	0.36554	2E-13	1.5E-11
ARL14	0.365964	1.87E-13	1.43E-11
CD177	0.366087	1.83E-13	1.42E-11
POU2AF1	0.366342	1.76E-13	1.37E-11
TMPRSS2	0.366585	1.69E-13	1.33E-11
GALM	0.367053	1.57E-13	1.24E-11
LGR4	0.367185	1.53E-13	1.22E-11
DUOXA1	0.367848	1.38E-13	1.1E-11
UGT2B15	0.368174	1.31E-13	1.05E-11
CEP70	0.369748	1.01E-13	8.3E-12
IFI27	0.369935	9.78E-14	8.08E-12

VWA3B	0.371038	8.16E-14	6.86E-12
HN1	0.371404	7.68E-14	6.48E-12
COX5A	0.371685	7.33E-14	6.21E-12
COL9A2	0.371886	7.09E-14	6.06E-12
OLFM4	0.371919	7.06E-14	6.06E-12
ATP5B	0.371989	6.97E-14	6.01E-12
TNFRSF17	0.372819	6.08E-14	5.33E-12
FAM46C	0.372845	6.05E-14	5.33E-12
CATSPERB	0.373068	5.83E-14	5.19E-12
RILP	0.373282	5.63E-14	5.05E-12
F2RL1	0.37433	4.73E-14	4.28E-12
LRRC59	0.374372	4.7E-14	4.27E-12
ZC3H12A	0.376378	3.36E-14	3.07E-12
C18orf56	0.376474	3.3E-14	3.03E-12
ACSM1	0.377386	2.83E-14	2.65E-12
IQGAP2	0.377495	2.78E-14	2.61E-12
NRARP	0.377955	2.57E-14	2.45E-12
GOT1	0.378073	2.52E-14	2.42E-12
ERN2	0.378953	2.17E-14	2.11E-12
GUCA2B	0.379041	2.14E-14	2.09E-12
GALNT8	0.379956	1.83E-14	1.81E-12
GOLM1	0.380064	1.8E-14	1.79E-12
LRRC26	0.380408	1.7E-14	1.69E-12
PTGER4	0.380945	1.55E-14	1.56E-12
B3GALT1	0.381023	1.53E-14	1.55E-12
CA12	0.381487	1.41E-14	1.44E-12
PLA2G2A	0.382269	1.24E-14	1.27E-12
GCNT3	0.382269	1.24E-14	1.27E-12
APOB48R	0.382317	1.23E-14	1.27E-12
ASS1	0.383593	9.85E-15	1.03E-12
FCAMR	0.385641	6.91E-15	7.37E-13
SLC39A8	0.386841	5.61E-15	6.02E-13
ALAS1	0.387442	5.06E-15	5.45E-13
PDZK1IP1	0.387587	4.93E-15	5.34E-13
ABCA12	0.388973	3.87E-15	4.21E-13
AIM1	0.389298	3.65E-15	4E-13
UQCRFS1	0.389383	3.6E-15	3.97E-13
HADH	0.389593	3.47E-15	3.84E-13
SLC4A4	0.389641	3.44E-15	3.83E-13
CLINT1	0.389937	3.27E-15	3.66E-13
CHAC2	0.390185	3.13E-15	3.52E-13
FAM3D	0.390327	3.05E-15	3.46E-13
TTLL6	0.391159	2.63E-15	3.01E-13
STS	0.391195	2.62E-15	3.01E-13
FAM177B	0.391691	2.4E-15	2.8E-13
SI	0.391916	2.3E-15	2.7E-13
LOC15332	0.392017	2.26E-15	2.67E-13
SLC6A14	0.392425	2.1E-15	2.51E-13
CA4	0.393445	1.76E-15	2.14E-13
ELOVL6	0.393559	1.72E-15	2.11E-13
RSPH1	0.393695	1.68E-15	2.07E-13
BEST2	0.393736	1.67E-15	2.07E-13
CPT2	0.394	1.59E-15	1.98E-13
FAM162A	0.394357	1.49E-15	1.87E-13
FAM118B	0.394672	1.41E-15	1.78E-13
LGALS9	0.394865	1.36E-15	1.73E-13
UQCRC1	0.395444	1.23E-15	1.57E-13
AGR2	0.396123	1.09E-15	1.4E-13

GPR15	0.396466	1.02E-15	1.33E-13
C2orf7	0.396598	9.99E-16	1.3E-13
RPS6KA1	0.396856	9.54E-16	1.25E-13
SLC41A2	0.397704	8.18E-16	1.08E-13
BARX2	0.397715	8.17E-16	1.08E-13
BRP44L	0.398188	7.5E-16	1.01E-13
ACPP	0.398877	6.62E-16	9.11E-14
PCSK7	0.399278	6.16E-16	8.54E-14
RNF145	0.400258	5.15E-16	7.24E-14
FH	0.400786	4.68E-16	6.67E-14
HHLA2	0.401176	4.36E-16	6.26E-14
PLA2G3	0.401748	3.92E-16	5.68E-14
ITM2C	0.402081	3.69E-16	5.38E-14
GPR110	0.403219	3E-16	4.4E-14
MS4A12	0.403239	2.98E-16	4.4E-14
MYPN	0.403694	2.75E-16	4.12E-14
CHP	0.404453	2.39E-16	3.64E-14
B4GALNT2	0.405099	2.12E-16	3.28E-14
IL1R2	0.406033	1.78E-16	2.78E-14
CEACAM7	0.406064	1.77E-16	2.78E-14
BCL10	0.407547	1.34E-16	2.13E-14
GSDMB	0.409502	9.32E-17	1.5E-14
TMEM61	0.409683	9.01E-17	1.46E-14
HEPACAM	0.409712	8.96E-17	1.46E-14
RBM47	0.410647	7.51E-17	1.25E-14
DMBT1	0.410894	7.17E-17	1.2E-14
RNF183	0.411405	6.51E-17	1.1E-14
IGJ	0.411742	6.11E-17	1.04E-14
CDS1	0.413174	4.65E-17	8.1E-15
NUDT8	0.413704	4.21E-17	7.38E-15
C4BPA	0.414492	3.62E-17	6.47E-15
NAT1	0.415671	2.89E-17	5.21E-15
MB	0.416629	2.4E-17	4.41E-15
C15orf48	0.41692	2.27E-17	4.21E-15
ACADS	0.417743	1.94E-17	3.63E-15
SH3RF2	0.418292	1.74E-17	3.29E-15
XDH	0.418555	1.66E-17	3.19E-15
SDHA	0.419293	1.44E-17	2.79E-15
ASRGL1	0.41949	1.38E-17	2.71E-15
RHBDL2	0.41987	1.28E-17	2.55E-15
CYB561D2	0.420941	1.04E-17	2.09E-15
CKMT1B	0.421003	1.03E-17	2.08E-15
ETHE1	0.422452	7.75E-18	1.6E-15
FA2H	0.422482	7.71E-18	1.6E-15
FAM55A	0.422947	7.04E-18	1.5E-15
GPT	0.423433	6.39E-18	1.38E-15
TNFRSF11A	0.425065	4.63E-18	1.02E-15
SLC37A1	0.426023	3.83E-18	8.64E-16
SLC35A4	0.426454	3.52E-18	8.02E-16
ABHD3	0.426473	3.5E-18	8.02E-16
GMDS	0.42674	3.32E-18	7.75E-16
BLNK	0.426861	3.24E-18	7.66E-16
MFSD2A	0.426904	3.22E-18	7.66E-16
BCL2L15	0.427931	2.62E-18	6.34E-16
PXMP2	0.427932	2.62E-18	6.34E-16
TNFSF13	0.428078	2.55E-18	6.31E-16
SCGB2A1	0.429791	1.81E-18	4.53E-16
STYK1	0.430456	1.58E-18	4.02E-16

CHCHD10	0.430515	1.56E-18	4.02E-16
BTNL8	0.431004	1.41E-18	3.69E-16
SLC44A4	0.431048	1.4E-18	3.69E-16
SPINK4	0.431652	1.24E-18	3.32E-16
ALDH1L1	0.432337	1.08E-18	2.97E-16
C11orf86	0.432534	1.04E-18	2.9E-16
VSIG2	0.432862	9.71E-19	2.79E-16
MRAP2	0.433176	9.11E-19	2.66E-16
C4BPB	0.43369	8.2E-19	2.43E-16
GPA33	0.433984	7.73E-19	2.32E-16
C14orf129	0.43403	7.66E-19	2.32E-16
HK2	0.434998	6.28E-19	1.98E-16
GMPPB	0.435684	5.46E-19	1.75E-16
AHCYL2	0.441268	1.72E-19	5.59E-17
FAS	0.442728	1.27E-19	4.19E-17
CASP10	0.444439	8.84E-20	2.97E-17
ABCC13	0.444444	8.83E-20	2.97E-17
TMEM54	0.445664	6.82E-20	2.37E-17
LDHD	0.447057	5.07E-20	1.8E-17
ZG16	0.4478	4.33E-20	1.56E-17
AGR3	0.449845	2.8E-20	1.03E-17
CCDC109A	0.450086	2.66E-20	9.93E-18
HYAL1	0.45014	2.62E-20	9.93E-18
TC2N	0.450819	2.27E-20	8.82E-18
KLF4	0.454192	1.09E-20	4.33E-18
CARD16	0.456201	7.04E-21	2.85E-18
BMP2	0.45623	6.99E-21	2.85E-18
SLC28A2	0.456542	6.53E-21	2.75E-18
BAK1	0.45687	6.08E-21	2.62E-18
TRIM40	0.45761	5.16E-21	2.27E-18
B3GNT6	0.468102	4.91E-22	2.21E-19
B3GALT5	0.470841	2.62E-22	1.21E-19
NOS2	0.471962	2.03E-22	9.56E-20
SIAE	0.472275	1.88E-22	9.11E-20
NR3C2	0.474317	1.17E-22	5.82E-20
C4orf19	0.480838	2.53E-23	1.29E-20
CLCA1	0.482589	1.67E-23	8.69E-21
AKR1B10	0.482904	1.54E-23	8.28E-21
PLAC8	0.483891	1.22E-23	6.72E-21
PAPSS2	0.484042	1.18E-23	6.66E-21
LCN2	0.485743	7.8E-24	4.55E-21
CASP5	0.486729	6.15E-24	3.69E-21
CTSE	0.48676	6.1E-24	3.69E-21
CASP7	0.486807	6.03E-24	3.69E-21
MUC1	0.488301	4.19E-24	2.77E-21
CYP2C18	0.489333	3.26E-24	2.23E-21
SPDEF	0.489995	2.77E-24	1.96E-21
MUC4	0.490465	2.47E-24	1.81E-21
DAPP1	0.490683	2.34E-24	1.79E-21
CHST5	0.493201	1.26E-24	9.98E-22
REG4	0.493311	1.22E-24	9.98E-22
SLC9A2	0.494964	8.12E-25	7E-22
CASP1	0.497239	4.6E-25	4.14E-22
ITLN1	0.499232	2.78E-25	2.63E-22
CLCA4	0.50226	1.29E-25	1.28E-22
MOBKL2B	0.506805	4.01E-26	4.18E-23
NANS	0.507596	3.27E-26	3.6E-23
MUC2	0.5091	2.21E-26	2.58E-23

LIMA1	0.515564	4.01E-27	4.97E-24
COQ2	0.519878	1.26E-27	1.67E-24
GPR120	0.520107	1.18E-27	1.67E-24
CA2	0.528385	1.22E-28	1.86E-25
DHRS9	0.530903	6.04E-29	9.99E-26
SQRDL	0.535128	1.83E-29	3.3E-26
FCGBP	0.537557	9.15E-30	1.81E-26
C6orf105	0.537825	8.47E-30	1.81E-26
DUOX2	0.550048	2.36E-31	5.84E-28
DUOXA2	0.550409	2.12E-31	5.84E-28
ST6GALNA	0.550991	1.78E-31	5.84E-28
TSPAN1	0.560988	8.41E-33	3.33E-29
CCL28	0.573959	1.37E-34	6.8E-31
LRG1	0.576107	6.81E-35	4.5E-31
CES3	0.588525	1.08E-36	1.07E-32
PIGR	1	1E-36	1E-32

ıip with PIGR.
