# Supplementary material for: The Underlying Roles of Exosome-Associated PIGR in Fatty Acid Metabolism and Immune Signaling in Colorectal Cancer
Source: J Oncol. 2022 Sep 15;2022:4675683. doi: 10.1155/2022/4675683 (PMC9499750; doi:10.1155/2022/4675683)
Supplement: Supplementary Materials — Supplementary Table 1. The upregulated genes and downregulated genes in the three GEO datasets. Supplementary Table 2. The coexpressed genes possess a positive and negative relationships with PIGR. Supplementary Table 3. The top 20 genes positively correlated with PIGR in colorectal cancer. Supplementary Table 4. The top 20 genes negatively correlated with PIGR in colorectal cancer. [file 4675683.f1.zip › 4675683.f1/Supplementary Table 4.docx]

Supplementary Table 4. The top 20 genes negatively correlated with PIGR in colorectal cancer.

| GDPD5 | PCMTD2 | PAN3 | ASXL1 | CHD6 |
| --- | --- | --- | --- | --- |
| C11orf95 | KBTBD6 | PLCG1 | ETNK2 | SLC2A12 |
| BCAM | PCIF1 | ZNF251 | WBP4 | GNAS |
| SORBS1 | PHLDB3 | ZMYM5 | HSDL1 | PRPF6 |
